# KIF21B Expression in Osteosarcoma and Its Regulatory Effect on Osteosarcoma Cell Proliferation and Apoptosis Through the PI3K/AKT Pathway

**DOI:** 10.3389/fonc.2020.606765

**Published:** 2021-01-28

**Authors:** Songjia Ni, Jianjun Li, Sujun Qiu, Yingming Xie, Kaiqin Gong, Yang Duan

**Affiliations:** Department of Orthopedics, Zhujiang Hospital, Southern Medical University, Guangzhou, China

**Keywords:** KIF21B, osteosarcoma, bioinformatics, proliferation, apoptosis, PI3K/AKT pathway

## Abstract

Osteosarcoma (OS) is the most common malignancy that occurs mainly during childhood and adolescence; however, no clear molecular or biological mechanism has been identified. In this study, we aimed to explore new biomarkers for the early diagnosis, targeted treatment, and prognostic determination of osteosarcoma. We first used bioinformatics analysis to show that KIF21B can be used as a biomarker for the diagnosis and prognosis of osteosarcoma. We then examined the expression of KIF21B in human osteosarcoma tissues and cell lines using immunohistochemistry, western blotting, and qRT-PCR. It was found that KIF21B expression was significantly upregulated in osteosarcoma tissues and cell lines. After knocking down the expression of KIF21B in the osteosarcoma cell lines 143B and U2-OS, we used cell fluorescence counting, CCK-8 assays, flow cytometry, and TUNEL staining to examine the effects of KIF21B on osteosarcoma cell proliferation and apoptosis. The results demonstrated that knocking down KIF21B in 143B and U2-OS cells could increase cell apoptosis, inhibit cell proliferation, and reduce tumor formation in nude mice. Subsequently, we used gene chips and bioinformatics to analyze the differential gene expression caused by knocking down KIF21B. The results showed that KIF21B may regulate OS cell proliferation and apoptosis by targeting the PI3K/AKT pathway. We then examined the expression of PI3K/AKT- and apoptosis-related proteins using western blotting. KIF21B knockdown inhibited the PI3K pathway, downregulated Bcl-2, and upregulated Bax. Moreover, the use of PI3K/AKT pathway agonists reversed the regulatory effect of KIF21B on the apoptosis and proliferation of 143B and U2-OS cells. In conclusion, our results indicated that KIF21B plays a key role in osteosarcoma. Low KIF21B expression might indirectly increase the apoptosis and inhibit the proliferation of osteosarcoma cells through the PI3K/AKT pathway.

## Introduction

Osteosarcoma (OS) is the most common primary malignant tumor in children and adolescents (1) and occurs in long bones, such as the proximal humerus and distal femur (2). OS is prone to metastasis and relapse, and 20–25% of patients present with lung metastases at the time of diagnosis (3). The current treatment for OS involves surgery and adjuvant chemotherapy, and the prognosis is poor, with 5-year survival rates below 20% (4).

KIF21B belongs to the kinesin superfamily of proteins (5). Human kinesins are divided into 14 families that consist of 45 different kinesin proteins and are involved in a series of cellular processes, such as mitosis, motility, organelle transport, and tumor development (6). Studies have confirmed that KIF21B is expressed in many types of cells, including neurons (7, 8). However, there are few studies on the relationship between the KIF21B protein and human tumors. When preparing this manuscript, we identified one study that first observed a relationship between KIF21B and hepatocellular carcinoma (9). The function and mechanisms of KIF21B in cancer, particularly in OS, and its effect on prognosis have not yet been widely investigated and remain unknown.

The PI3K/Akt signaling pathway is composed of a conserved family of signal transducing enzymes that are involved in a wide range of physiological processes (10, 11). The PI3K/AKT signaling pathway is also one of the most commonly dysregulated pathways in cancer (12). Therefore, the PI3K/AKT pathway has become the main focus of drug development for the treatment of cancer (13). Moreover, the PI3K/AKT signaling pathway also plays an important role in the regulation of various kinesin proteins (14, 15).

In this study, we first identified KIF21B as a potential new biomarker of OS through bioinformatics analysis. To explore the impact of KIF21B on OS, we examined the expression of KIF21B in osteosarcoma tissues and cell lines. Then, we evaluated the effects of KIF21B on the proliferation and apoptosis of 143B and U2-OS osteosarcoma cells. We further analyzed the differential gene expression caused by knocking down KIF21B and predicted and verified the potential downstream target genes of KIF21B. The purpose of this study was to investigate the role of KIF21B in the progression of OS and to provide ideas for new treatment options for OS.

## Materials and Methods

### Data Processing

The OS-related chip data were downloaded from the GEO database (http://www.ncbi.nlm.nih.gov/geo), including GSE12865 (12 osteosarcoma cells and 2 normal human osteoblastic cells). The differentially expressed mRNAs were analyzed with an adjusted P value <0.05 and |Log2FC| >0.25 as the threshold using the limma package in R software. The dysregulated mRNAs were used for future analysis, and the expression levels of the mRNAs were visualized by volcano plots using the ggplot2 package in R. In addition, the RNA-seq data and clinical features of OS patients were collected from the TCGA database (TARGET-OS, N = 85). The characteristics of the patients included in this study are summarized in **Table 1**.

**Table 1:** The information of clinical features.

Characteristics	Number of cases	Percentages
Age		
<20	74	87.06
>20	11	12.94
Gender		
Female	37	43.53
Male	48	56.47
Race		
Asian	6	7.06
Black or African American	7	8.24
White	51	60.00
Others	21	24.70

### Protein-Protein Interaction Network Analysis

The interactive relationships among the dysregulated genes were analyzed using a protein-protein interaction (PPI) network. The gene information was retrieved from the Search Tool for the Retrieval of Interacting Genes (STRING) database (version 11.0, https://string-db.org/). The PPI network was constructed and visualized by using Cytoscape 3.7.2 software. The ClusterONE algorithm was used to mine the hub modules in the PPI network. The threshold parameters were set to minimum size = 50 and density threshold = 0.5. The nodes with a high degree of connectivity (top 15, degree ≥9) were considered hub genes involved in important biological functions.

### Functional Enrichment and GO Semantic Similarity

Enrichment analysis of the dysregulated genes was used to investigate the biochemical pathways involved in carcinogenesis and tumor progression. The GO functional enrichment analysis and KEGG pathway analysis were performed using the clusterProfiler package in R. We set the criterion that the P value of the GO terms and KEGG analysis was less than 0.05. The top 15 enriched GO terms and KEGG pathways were visualized using the ggplot2 package in R. The functional similarity between genes was measured by semantic similarity with GO terms [17344234]. The semantic similarity computation among genes was performed using the GOSemSim R package.

### Receiver Operating Characteristic Curve Analysis

Receiver operating characteristic (ROC) curve analysis was used to evaluate the diagnostic value of the target mRNAs in patients with OS by calculating the sensitivity and specificity. The area under the curve (AUC) of the ROC curve was used to assess the discrimination performance. An AUC above 0.7 was considered satisfactory diagnostic accuracy [29901123].

### Survival, Univariable, and Multivariable Analyses

Kaplan-Meier survival analysis was performed by using the survival R package to predict the overall survival of the OS patients. The stratified log-rank test was used to compare the differences in overall survival time between the two groups. The Cox proportional hazards regression model was used to explore the univariable and multivariable hazard ratios (HRs) and screen the OS prognostic factors [31216997]. The univariable Cox proportional hazards regression model was used to select the independent clinicopathological factors. Subsequently, the multivariable Cox proportional hazards regression model was implemented to explore the potential prognostic biomarkers of OS. The patients with OS were divided into high-risk and low-risk groups according to the risk score in the hazards model. The time-dependent ROC curve was used to assess the risk prediction rate of the target genes between the high-risk and low-risk groups. All the statistical analyses were performed using R and SPSS 22.0 software. A P value less than 0.05 was considered statistically significant.

### Specimens

A total of 17 pairs of OS tissues and corresponding adjacent tissues were collected from OS patients in the Orthopedics Department of Zhujiang Hospital of Southern Medical University. All the pathological specimens were diagnosed by postoperative pathology and derived from newly formed cancerous tissues with complete clinical data. The patients did not receive preoperative chemoradiotherapy. The patients’ informed consent was acquired for all the procedures related to their tissues, and the study was approved by the Ethics Committee of the Zhujiang Hospital of Southern Medical University.

### Cell Culture

The human osteoblast cell line hFOB1.19 and the human OS cell lines 143B, U2-OS and MG63 were purchased from the American Type Culture Collection (ATCC, Rockville, MD, USA). The cells were cultured in RPMI 1640 medium (HyClone Technologies, Logan, USA) containing 10% fetal bovine serum (FBS, Thermo Fisher Scientific, MA, USA) and 100 U/ml anti-bis (Thermo Fisher Scientific) in 5% CO2 at 37°C.

### Immunohistochemistry

Human tissues were fixed in 10% formalin (Solarbio, Beijing, China) and embedded in paraffin. Five-micrometer sections were dewaxed with dimethylbenzene (Macklin, Shanghai, China) and dehydrated in a gradient ethanol series, followed by incubation with citrate buffer for antigen recovery. Hydrogen peroxide (Macklin, 3%) was used to block the endogenous peroxidase activity. The sections were incubated with primary antibodies against KIF21B (1:500; Thermo Fisher) overnight at 4°C. The sections were then stained using biotinylated secondary antibodies at 37°C for 20 min and exposed using DAB. The images were captured by microscopy (Leica, Wetzlar, Hesse-Darmstadt, Germany). Three fields were randomly selected, and the average percentage of brown or dark yellow particles in the cytoplasm was counted.

### Western Blotting

The total protein was extracted using a whole cell lysis assay (KeyGEN Biotech, Nanjing, Jiangsu Province, China). Eighty micrograms of sample protein was subjected to SDS-PAGE (KeyGEN Biotech) and transferred to PVDF membranes (Millipore, MA, USA). The membranes were blocked and probed with the indicated primary antibodies at 4°C for 12 h. The membranes were then incubated with the indicated HRP-conjugated secondary antibodies at room temperature for 2 h, and the expression of the target proteins was detected by ECL (KeyGEN Biotech). The following antibodies were used: KIF21B (1:3,000; Thermo Fisher), PI3K (1:3,000; Thermo Fisher), p-PI3K (1:1,000; Thermo Fisher), AKT (1:3,000; Thermo Fisher), p-AKT (1:1,000; Thermo Fisher), Bcl-2 (1:2,000; Cell Signaling Technology, Boston, MA, USA), BAX (1:1,000; Cell Signaling Technology), and Actin (1:1,000; Beyotime Biotechnology, Shanghai, China).

### qRT-PCR

The total RNA was extracted and synthesized into cDNA according to the manufacturer’s protocol (TaKaRa Bio, Dalian, Liaoning Province, China). qRT-PCR was performed on a LightCycler 96 (Roche Life Sciences, Switzerland, Basel) using Real-Time PCR Mix (Vazyme Biotech, Nanjing, Jiangsu Province, China). Gene expression relative to GAPDH expression was assessed using the 2-ΔΔCt method. Independent experiments were conducted in triplicate (KIF21B upstream sequence: 5’-GGATGCCACAGATGAGTT-3’, downstream sequence: 5’-TGTCCCGTAACCAAGTTC-3. GAPDH upstream sequence: 5′-ATAGCACAGCCTGGATAGCAACGTAC-3′, downstream sequence: 5′-CACCTTCTACAATGAGCTGCGTGTG-3′.)

### Lentivirus Infection

A short hairpin RNA (shRNA) sequence specific for human KIF21B (GGAGCTGATGGAGTATAAG) and a negative control sequence (TTCTCCGAACGTGTCACGT) were constructed and confirmed. Lentivirus with and without fluorescence were provided and titered by GeneChem (Shanghai, China). The cells in the shKIF21B group were transduced with shRNA lentivirus with a multiplicity of infection (MOI) of 20. The shCtrl group was transduced with a negative control sequence, and the blank control group was not treated. Seventy-two hours after transduction, the cells were continuously cultured for 1 week using complete medium containing puromycin (2 µg/ml, Beyotime Biotechnology).

### Cell Growth Assessments

Virus-infected cells exhibiting green fluorescence were counted on a Celigo instrument (Nexcelom, Lawrence, MA, USA). After 3–5 days of continuous measurements, cell growth curves were plotted to reflect cell growth.

### Cell Counting Kit-8

Cells from each group were seeded into a 96-well plate at a density of 2,000 cells/well, and 10 μl CCK-8 solution (Dojindo, Kumamoto, Japan) was added to each well every 24 h. After incubation at 37°C for 2 h, the absorbance value at 450 nm was detected using a microplate reader (Bio-Rad, Hercules, USA). Triplicate wells were used for all the specimens in each test.

### Cell Apoptosis Analysis by Flow Cytometry

The Annexin FITC/PI Apoptosis Detection Kit (KeyGEN Biotech) was used following the manufacturer’s protocols. A total of 1 × 106 cells were resuspended in a mixture of 100 μl of binding buffer, 10 μl of FITC Annexin V, and 10 μl of PI solution and then incubated in the dark for 15 min at room temperature. Then, cell apoptosis was measured using a flow cytometer (BD Biosciences, NJ, USA). The results were analyzed using FlowJo software. The experiment was repeated in triplicate.

### TUNEL Assay Kit

Adherent cells were sequentially fixed with 4% paraformaldehyde (Beyotime Biotechnology) for 30 min and incubated with Triton X-100 (Beyotime Biotechnology) for 5 min at room temperature. Then, the TUNEL test solution was prepared according to the operating instructions (Beyotime Biotechnology). The cells were added to 50 μl TUNEL detection solution and incubated at 37°C for 60 min in the dark, and the nuclei were stained with DAPI (Beyotime Biotechnology). The nuclei and TUNEL-positive cells were then observed under a fluorescence microscope (Nikon, Tokyo, Japan), and the positive rate (TUNEL/DAPI) of five random fields was calculated.

### Grouping and Treatment of Nude Mice

Nine BALB/c nude mice (6 weeks, approximately 18 g, Animal Center of Southern Medical University) were used for the tumor formation experiments. A total of 3 × 106 143B cells were subcutaneously injected into the right forelegs of the nude mice. A Vernier caliper was used to measure the longest and shortest diameters of the transplanted tumor every 5 days, the volume of the subcutaneous transplanted tumor was calculated (V = L × W2 × 0.5), and the tumor growth curve was drawn. One month after the injection, the mice were anesthetized by using carbon dioxide. The tumor tissue was then removed, weighed, and imaged. The animal experiment was performed in strict accordance with the National Institutes of Health Guide for the Care and Use of Laboratory Animals.

### Gene Microarray Hybridization, Scanning, and Analysis

Hybridization of nucleic acid probes and gene expression profiling chips were performed with a gene chip hybridization kit (Thermo Fisher) for 16 h. Elution and staining were performed using Onfly Fluid Workstation 450. A Gene Chip Scanner 3000 7G scan (Affymetrix, Thermo Fisher) was used to generate images. Chip scan image 5.0 software was used for digital processing and analysis.

### Statistical Analysis

All the data were analyzed using SPSS 22.0 software. The qualitative data are presented as counts (%), and the quantitative data are expressed as the mean ± standard deviation. The measurement data that conformed to a normal distribution were assessed *via* T-tests. Mann-Whitney rank sum tests were used to analyze the data that did not conform to a normal distribution. Three groups of data were analyzed through one-way analysis of variance. Differences were statistically significant at the P < 0.05 level.

## Results

### Comprehensive Analysis Identified KIF21B as a Potential Diagnostic and Prognostic Gene in OS

**Figure 1:**
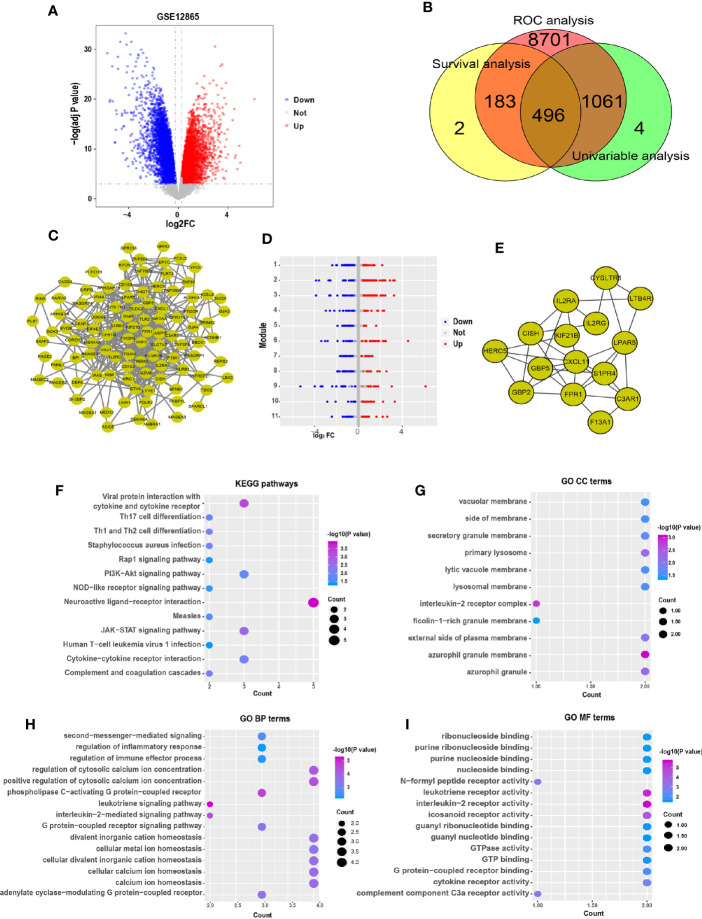
Comprehensive expression analysis for the identification of hub genes associated with the progression of osteosarcoma. **(A)** The volcano plot of significant differentially expressed genes. The red dots represent the upregulated genes, and the blue dots represent the downregulated genes. **(B)** Venn diagram representing the number of dysregulated genes in the ROC, survival and univariable analyses. **(C)** A PPI network of the dysregulated genes was constructed. **(D)** The modules were identified from the PPI network using the ClusterONE algorithm, containing 11 modules. The red dots represent the upregulated genes, the blue dots represent the downregulated genes, and the gray dots represent the genes with no significant difference in expression. **(E)** Interaction network of hub genes. **(F)** Significant pathways in the KEGG pathway analysis with P < 0.05. **(G)** Eleven GO terms were enriched for cellular components. **(H)** The top 15 enriched biological process GO terms of the dysregulated genes. **(I)** The top 15 enriched molecular functions GO terms.

First, principal component analysis (PCA) was performed to identify abnormal samples (**Supplementary Figure 1**). Then, the differentially expressed genes were analyzed by using the limma package in R software. A total of 10,446 differentially expressed genes (DEGs) were identified between the osteosarcoma cells and normal human osteoblastic cells with an adjusted P value <0.05 and |Log2FC| > 0.25 as the threshold. There were 4,875 upregulated and 5,571 downregulated mRNAs (**Figure 1A**). To screen and predict the possible diagnostic mRNAs in OS, the diagnostic values were analyzed using ROC curves. A total of 10,441 DEGs were used in the subsequent studies with AUC > 0.7 as the cutoff criterion (**Supplementary Table 1**). To select novel genes for predicting the overall survival of patients with OS, survival analysis was performed by the Kaplan-Meier method with the log-rank test. The results showed that 1,561 novel genes were associated with the overall survival of OS patients, with a P value <0.05 (**Supplementary Table 2**). To identify the potential prognostic indicators of OS, univariable Cox regression analysis was performed to identify independent risk factors for the prognosis of OS. Here, we identified 681 DEGs that were significantly associated with cancer-specific survival with a P value <0.01 as the threshold (**Supplementary Table 3**). Subsequently, 496 potential target genes were obtained as effective diagnostic and prognostic genes (**Figure 1B**).

To identify the interactions between these 496 target genes, a PPI network was constructed by using the STRING database, with scores >950 as the threshold. The results showed that 100 genes were related to each other in the PPI network (**Figure 1C**). Moreover, the ClusterONE algorithm was particularly useful for detecting protein complexes in the PPI network. A total of 11 PPI modules were identified, with a P value <0.05 (**Figure 1D**). The hub genes were considered to play important roles in the highly intersected network. Fifteen hub genes were identified by network clustering analysis with degree ≥9 as the cutoff criteria (**Figure 1E**).

To explore the biological functions of these hub genes, KEGG pathway and GO term enrichment analyses were performed using the clusterProfiler package in R. The results of the KEGG pathway analysis showed that these dysregulated genes were enriched in cancer-associated pathways, including the JAK-STAT, PI3K-Akt, NOD-like receptor, and Rap1 signaling pathways (**Figure 1F**). In addition, our results showed that 11 GO terms were enriched for cellular components (CC), 125 GO terms were enriched for biological processes (BP), and 31 GO terms were enriched for molecular functions (MF), with P < 0.05 as the threshold. The CC GO terms included those associated with secretory granule membrane and vacuolar membrane (**Figure 1G**), and the BP GO terms mainly included those associated with calcium ion homeostasis, second messenger and regulation of immune effector process (**Figure 1H**). The MF GO terms were associated with G protein-coupled receptor binding and nucleoside binding (**Figure 1I**).

**Figure 2:**
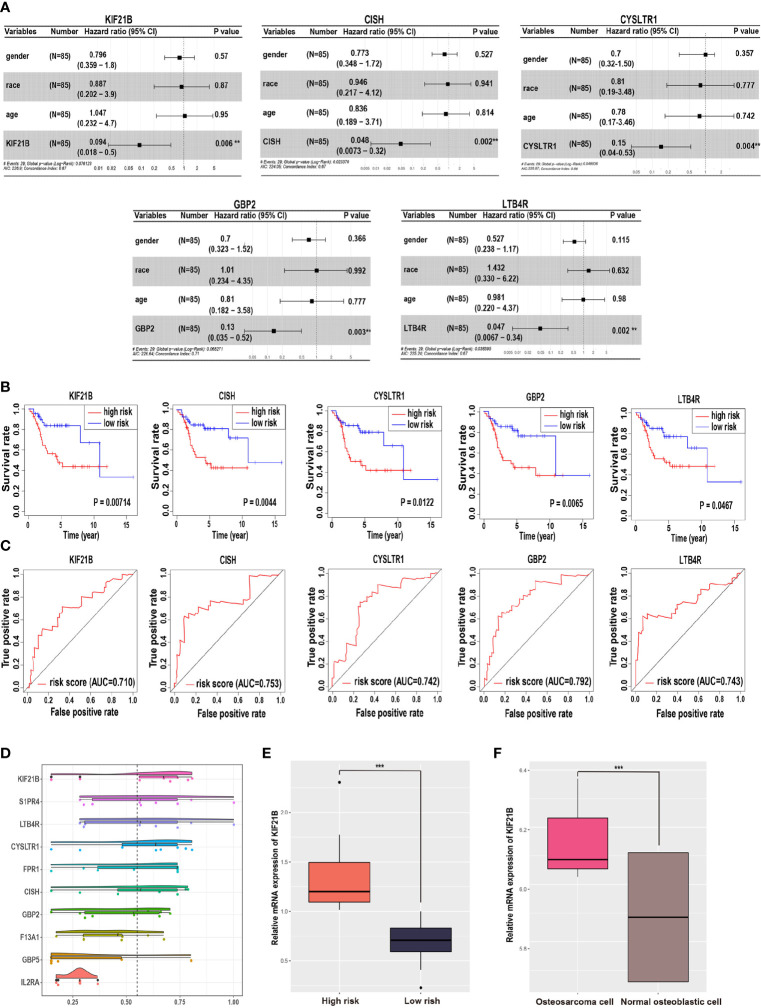
Identification of potential prognostic biomarkers for predicting survival in patients with osteosarcoma. **(A)** Forest plot indicating the hazard ratios for overall survival using a multivariable Cox regression model. **(B)** Kaplan-Meier curves of overall survival in patients with osteosarcoma. The red lines represent high-risk group samples, and the blue lines represent low-risk group samples. **(C)** The time-dependent receiver operating characteristic curve of the prognostic signature in osteosarcoma. **(D)** Functional semantic similarity between 10 hub genes. Boxplot of KIF21B mRNA expression in the high- and low-risk groups **(E)** and in osteosarcoma cells and normal human osteoblastic cells **(F)**. *** means P < 0.05.

In addition, multivariable Cox hazard regression analysis was performed to examine the independent risk factors. We constructed 15 Cox models using the hub genes and clinical features, including age, sex, and race. The results showed that 10 Cox models were identified as independent factors influencing survival time (P value < 0.01; Wald test), including KIF21B (HR = 0.094, 95% CI: 0.018–0.501, P = 0.006), LTB4R (HR = 0.047, 95% CI: 0.007–0.336, P = 0.002), CISH (HR = 0.048, 95% CI: 0.007–0.316, P = 0.002), GBP2 (HR = 0.135, 95% CI: 0.035–0.517, P = 0.003), CYSLTR1 (HR = 0.147, 95% CI: 0.040–0.533, P = 0.004), F13A1 (HR = 0.176, 95% CI: 0.070–0.445, P < 0.0001), GBP5 (HR = 0.296, 95% CI: 0.136–0.642, P = 0.002), FPR1 (HR = 0.358, 95% CI: 0.182–0.701, P < 0.003), IL2RA (HR = 0.474, 95% CI: 0.294–0.767, P = 0.002), and S1PR4 (HR = 0.505, 95% CI: 0.308–0.827, P = 0.007). Visualizations of the top five results are shown in **Figure 2A**. Next, the key gene expression‐based survival risk score of the 85 patients was calculated. All the samples were divided into two groups, the high- and low-risk groups, according to the median risk score. The results showed that the high-risk score was associated with poorer overall survival (**Figure 2B**). Furthermore, a time-dependent ROC curve was used to assess the power of the prognostic factors. The AUC values for the prognostic models ranged from 0.710 to 0.792 at 3 years of overall survival (**Figure 2C**). Moreover, to identify novel biomarkers for further study, GO semantic similarity was used to quantify the functional similarity between 10 key genes. The results showed that KIF21B was considered to be protein with the most significantly strengthened relationship with its partners (**Figure 2D**). The expression of KIF21B was analyzed in the high- and low-risk patients with OS. The results showed that KIF21B was upregulated in the high-risk patients (**Figure 2E**). Taken together, these findings provide evidence that KIF21B is a diagnostic and prognostic biomarker and potential therapeutic target in patients with OS.

### Expression of KIF21B in Human OS Tissues and Cell Lines

**Figure 3:**
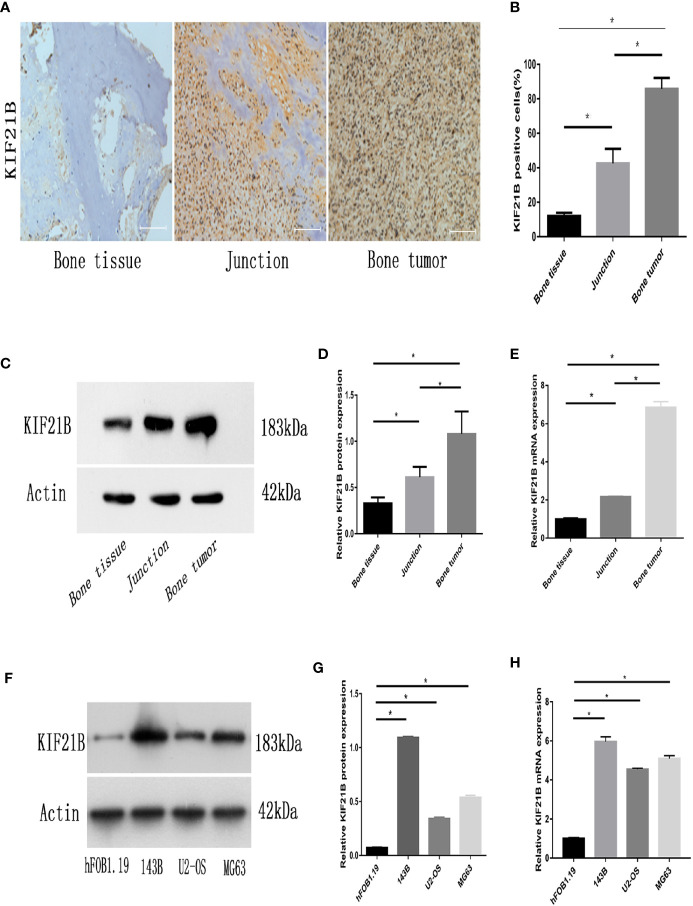
Expression of KIF21B in human OS tissues and OS cell lines. **(A, B)** Representative immunohistochemical images (magnification, 200×) of KIF21B-positive cells show the expression of KIF21B in human osteosarcoma tissue. **(C, D)** The protein expression levels of KIF21B in the human osteosarcoma samples were detected by western blot analysis. **(E)** qRT-PCR was used to measure the mRNA expression levels of KIF21B in the human osteosarcoma samples. **(F, G)** The protein expression levels of KIF21B in human osteoblast cells hFOB1.19 and human OS cell lines 143B, U2-OS, and MG63 were detected by western blot analysis. **(H)** qRT-PCR was used to measure the mRNA expression levels of KIF21B in the hFOB1.19 and OS cell lines. Scale bar = 200 μm, *P < 0.05.

To determine and assess the expression level of KIF21B in OS, the expression of KIF21B in osteosarcoma cells and normal human osteoblastic cells from the GEO database (GSE12865) was analyzed (**Figure 2F**). The results showed that KIF21B was expressed at high levels in patients with OS (adjusted P value = 0.0448). To explore the effect of KIF21B on the development of OS, we then compared the expression levels of KIF21B between tumor tissue, junction tissue and bone tissue. Immunohistochemical staining was performed to detect KIF21B expression. The results showed that the expression of KIF21B in the tumor tissues was significantly higher than that in the junction and bone tissues (**Figures 3A, B**). High KIF21B expression at the tumor site was also confirmed by western blotting analysis (**Figures 3C, D**) and qRT-PCR (**Figure 3E**). Then, qRT-PCR and western blotting were used to explore the expression of KIF21B in OS cell lines. The results demonstrated that the expression of KIF21B in the OS cell lines U2-OS, 143B, and MG63 was higher than that in the Hfob1.19 cell line (**Figures 3F–H**). These results demonstrated that KIF21B was typically overexpressed in OS, suggesting that it might be considered an oncogene.

### Effects of KIF21B Silencing on OS Cell Proliferation and Apoptosis

**Figure 4:**
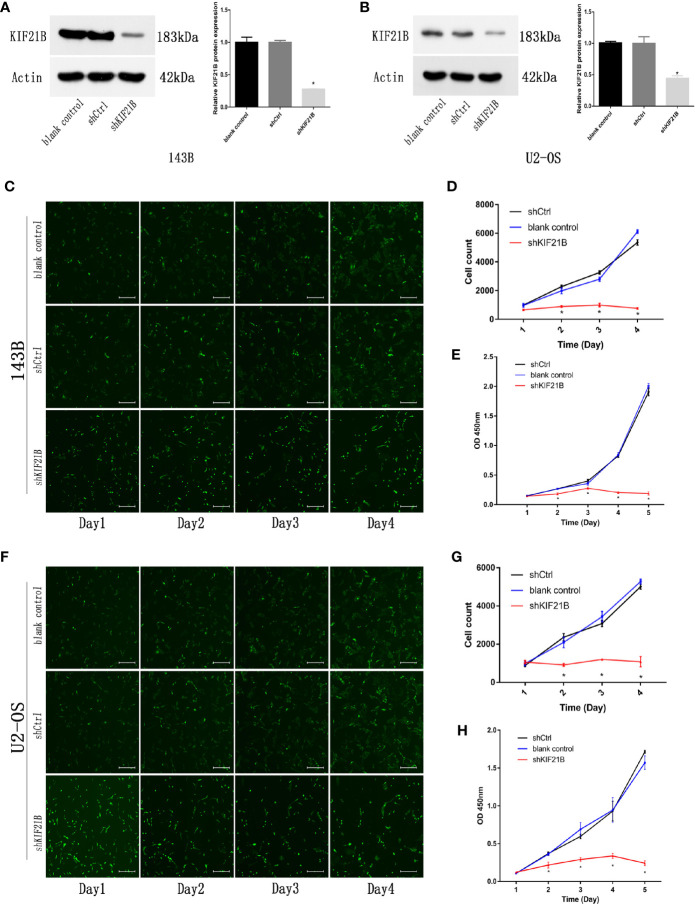
Effects of KIF21B silencing on cell proliferation. **(A, B)** Western blotting was used to measure the KIF21B protein expression in 143B and U2-OS cells following the transfection of KIF21B shRNA. The Celigo cell count and CCK-8 results showing the proliferation of 143B **(C–E)** and U2-OS **(F–H)** cells in each group. Scale bar = 200 μm, *P < 0.05.

**Figure 5:**
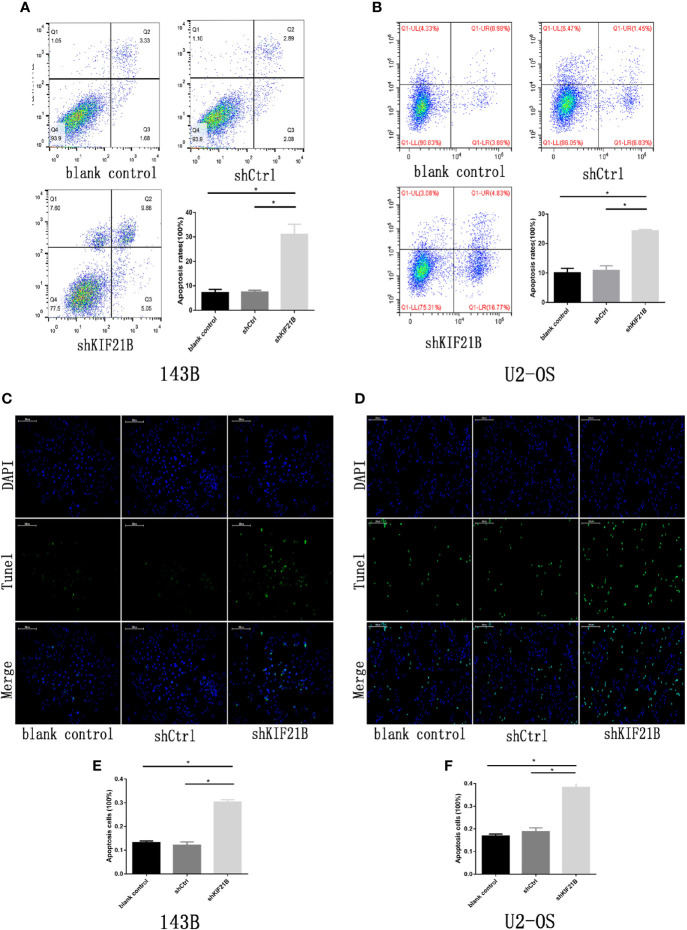
Effects of KIF21B silencing on cell apoptosis. **(A, B)** Flow cytometry results showing the apoptosis rate of 143B and U2-OS cells in each group. **(C–F)** TUNEL assays results showing the apoptosis rate of 143B and U2-OS cells in each group. Scale bar = 200 μm, *P < 0.05.

**Figure 6:**
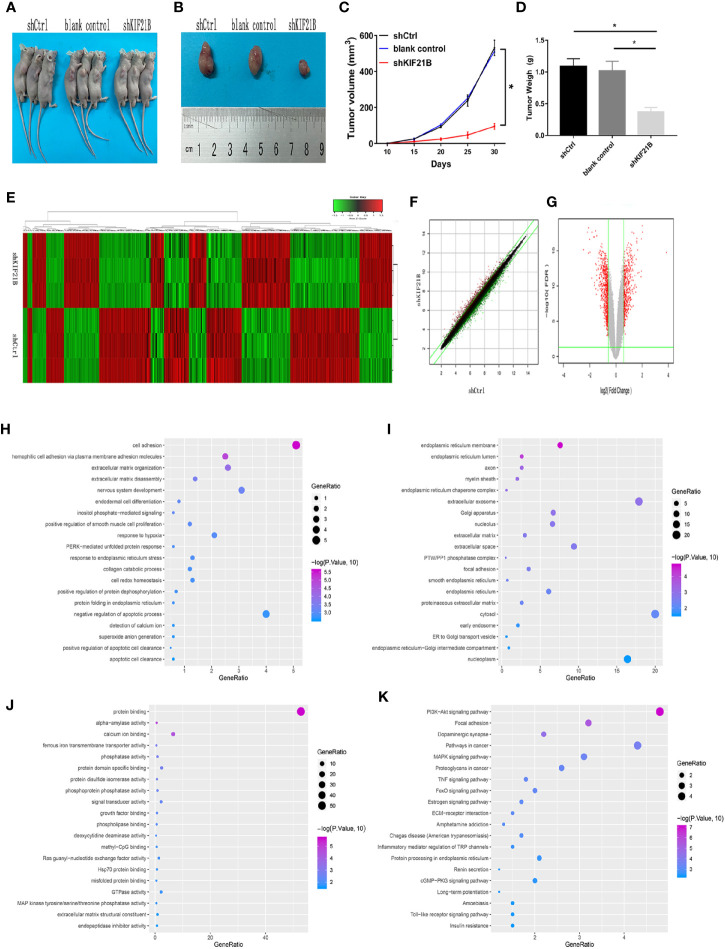
Effects of KIF21B silencing on tumor formation in nude mice and identification of differentially expressed genes. **(A, B)** Representative photo of nude mouse tumors. The tumor size **(C)** and weight **(D)** were measured after different treatments. **(E)** Heatmap of differentially expressed genes between the shCtrl group and shKIF21B group; The scatter plot **(F)** and volcanic map **(G)** of differentially expressed genes. **(H–J)** GO annotation showed the top 20 results of biological process (BP), cellular component (CC), and molecular function (MF) that were highly enriched GO annotations. **(K)** KEGG pathway analysis showed the top 20 pathways that were highly enriched. *P < 0.05.

To investigate the potential role of KIF21B in OS cell function, the expression of KIF21B was assessed 48 h after the transduction of 143B and U2-OS cells with shRNA specific for KIF21B. The KIF21B expression level in the shKIF21B group was significantly downregulated compared with that in the shCtrl group and blank control group (**Figures 4A, B**). Following transduction with the KIF21B lentivirus, the cells were assessed for 4 days, and the growth curves were plotted. The proliferation rate of the 143B cells in the shKIF21B group was significantly reduced compared with that in the control groups (**Figures 4C, D**). The CCK-8 results also demonstrated a significantly reduced absorbance value in the shKIF21B group (**Figure 4E**). In addition, a similar trend was also observed in U2-OS cells as well (**Figures 4F–H**). Moreover, the flow cytometry (**Figures 5A, B**) and TUNEL staining (**Figures 5C–F**) results showed an increased apoptosis rate in the cells transduced with shKIF21B. To confirm these findings *in vivo*, 143B cells were implanted into nude mice (**Figures 6A, B**), and the effects of KIF21B were assessed after 30 days of treatment. We measured the tumor size and weight (**Figures 6C, D**). Compared with the control, KIF21B reduced the tumor size and weight. These results indicated that low KIF21B expression could enhance the apoptosis and suppress the proliferation of OS cells.

### Screening of Differentially Expressed Genes and Analysis of Bioinformatics

To analyze the possible downstream targets of KIF21B, we used a human genome-wide expression chip, which contains 49,395 sets of probes, for further research. RNA probes from each treatment group were hybridized with the chip to analyze the changes in the gene expression profiles of osteosarcoma cells before and after KIF21B knockdown. The differentially expressed RNAs between the KIF21B-silenced OS cells and normal OS cells were analyzed to obtain heatmaps, scatter plots, and volcano plots (**Figures 6E–G**), the dots of the scatter plot in the parallel green solid lines represent the genes that are not significantly different, and the red and green dots represent the genes that are relatively upregulated or downregulated in the shKIF21B group, respectively. The volcanic map was plotted by fold change and FDR between the two groups of samples. The red points are the significantly differentially expressed genes screened with | fold change | ≥1.5 and FDR <0.05 as the standard, and the gray points are the other genes with no significant differences. The results showed that compared with the control, KIF21B gene silencing resulted in a decrease in the expression of 467 genes and an increase in the expression of 382 genes. The top 50 most significant differentially expressed genes are shown in **Supplementary Tables 4**, **5**. Then, the R software clusterProfiler package was used to analyze 849 differentially expressed RNAs. GO pathway analysis (**Figures 6H–J**) showed the top 20 results that were highly enriched. We found that the most enriched BP, CC, and MF terms were cell adhesion, endoplasmic reticulum membrane and protein binding, respectively. KEGG pathway analysis (**Figure 6K**) showed the top 20 pathways that were highly enriched. These pathways were mainly involved in the PI3K/Akt signaling pathway, focal adhesion, dopaminergic synapses, pathways in cancer, and MAPK signaling pathway.

### Silencing of KIF21B Affects Osteosarcoma Cell Function Through the PI3K/AKT Pathway

**Figure 7:**
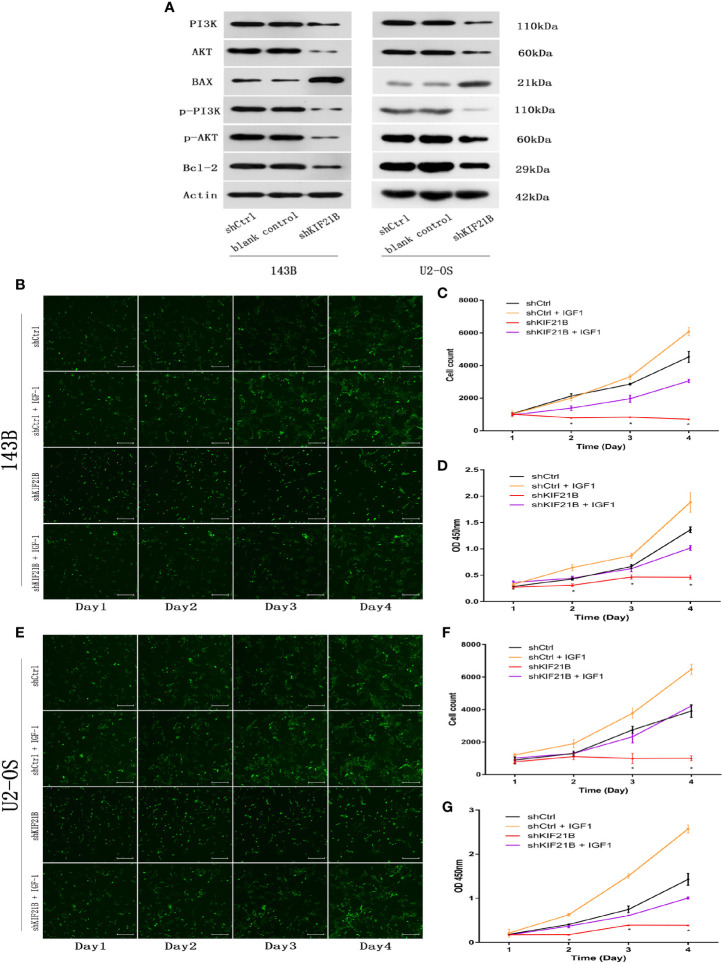
Validation of downstream target pathways and effects of IGF-1 on cell proliferation and apoptosis after silencing KIF21B. **(A)** The western blots show the PI3K/AKT, Bax, and Bcl2 results in the shKIF21B group and negative control groups. The Celigo cell count and CCK-8 results showing the proliferation of the 143B **(B–D)** and U2-OS **(E–G)** cells in the shKIF21B group and negative control groups after treatment with IGF-1 (200 μg/L) for 24 h. Scale bar = 200 μm, *P < 0.05.

**Figure 8:**
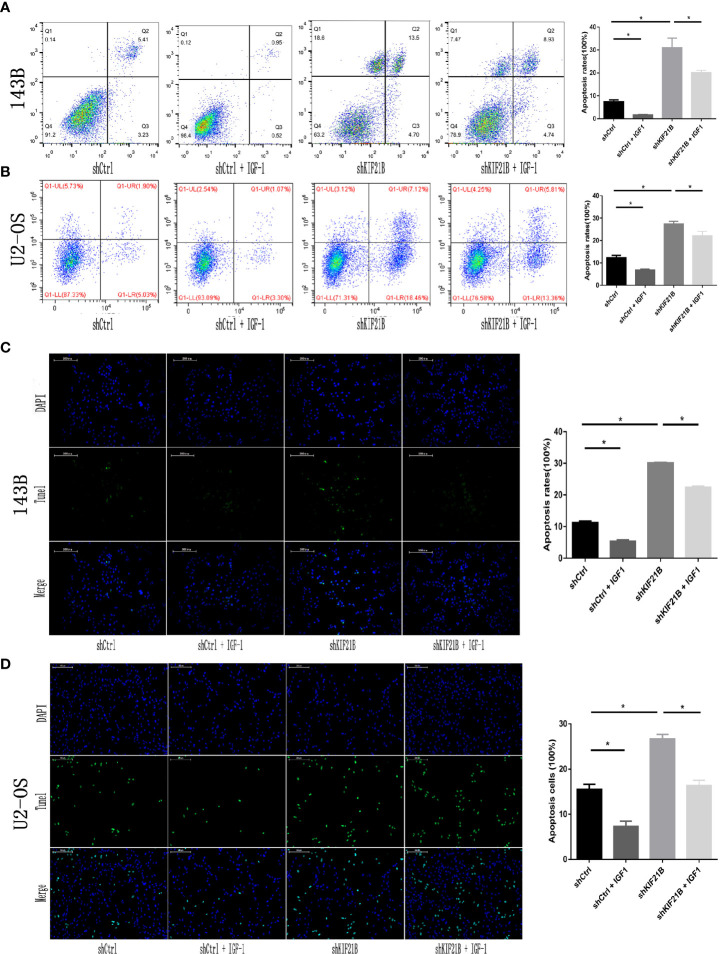
Effects of IGF-1 on cell apoptosis after silencing KIF21B. Flow cytometry **(A, B)** and TUNEL assays **(C, D)** results showing the apoptosis rate in each group after treatment with IGF-1 (200 μg/L) for 24 h. Scale bar = 200 μm, *P < 0.05.

Functional enrichment analysis of the differentially expressed genes suggested that the PI3K/AKT signaling pathway might play an important role in the KIF21B-mediated regulation of OS cells. Since the PI3K/AKT pathway is widely involved in tumor development, we further studied the changes in the key proteins of the PI3K/AKT pathway after silencing KIF21B. The western blot analysis results suggested that the expression levels of PI3K, p-PI3K, AKT, and p-AKT were significantly downregulated after silencing KIF21B. The expression levels of Bax were upregulated, and the expression levels of Bcl-2 were downregulated (**Figure 7A**). Thus, our study demonstrated that factors involved in the PI3K/ATK signaling pathway were significantly downregulated in the shKIF21B group. In addition, based on the results of silencing KIF21B, we treated the cells with the PI3K/AKT signaling pathway agonist IGF-1, and the results showed that the inhibition of proliferation and increase in apoptosis caused by silencing KIF21B were reversed by IGF-1 (**Figures 7** and **8**). These results confirmed that the regulatory effect of KIF21B on 143B and U2-OS cell proliferation and apoptosis might be associated with the PI3K/ATK signaling pathway.

## Discussion

OS is the most common primary malignant bone tumor, with a particular prevalence in adolescents and children (annual incidence of ~3.1 million) (16, 17). Current treatments include a combination of neoadjuvant chemotherapy and physical rescue. Although this method preserves joint function in the extremities, OS metastasis and recurrence remain common. OS cells are highly genetically unstable and histologically heterogeneous, and their signaling is complex (18). Exploring the molecular details of OS development can improve OS diagnostics and therapeutics.

In this study, we first conducted a series of bioinformatics analyses, and the comprehensive analysis determined that KIF21B is a potential new biomarker of OS. Polymorphisms in the KIF21B gene are associated with multiple sclerosis, ankylosing spondylitis, Crohn’s disease, and ulcerative colitis (19–21). Increased expression of KIF21B accelerates the progression of neurodegenerative diseases, such as Alzheimer’s disease and multiple sclerosis (22). In addition, microrepetitions carrying the KIF21B locus are associated with neurodevelopmental and psychological abnormalities (23). Basically no research has been conducted on the role of KIF21B in human tumors. On the other hand, the abnormal expression of multiple kinesin proteins in tumor cells has been observed (24). High expression of KIF5B is observed in breast (25) and skin cancer (26), and the overexpression of KIF14 promotes the development of retinoblastoma (27), lung cancer (28), and breast cancer (29). KIF7 and Eg5/KIF11 are associated with the occurrence and metastasis of various tumors (30). The expression of KIF21B in bone tumors and its related mechanisms have not been reported. Therefore, we wanted to determine whether KIF21B plays a role in the development of osteosarcoma. Combined with the results of the bioinformatics analysis, we detected the expression of KIF21B in OS tissues and cell lines. The results demonstrated that the expression of KIF21B is significantly upregulated. We then selected the OS cell lines 143B and U2-OS. Following KIF21B silencing, we found that cell proliferation was inhibited and cell apoptosis was increased, which indicate that KIF21B may act as an oncogene in osteosarcoma.

Under normal conditions, kinesin forms complexes with dynein molecules, which are involved in the transport of intracellular vesicles and organelles. However, the overexpression of kinesin proteins can generate additional external forces, leading to spindle collapse and monopolar spindle formation, which in turn lead to an uneven distribution of genetic material during the later stages, ultimately leading to aneuploidy. The genetic material acquired or lost in non-euploid cells is considered to be an factor that initiates the malignant progression of cancer (31). To further study the mechanism by which KIF21B participates in the development of osteosarcoma, we used a gene chip to identify differentially expressed genes before and after KIF21B silencing and performed bioinformatics analysis. According to the results, the differentially expressed genes caused by KIF21B silencing are mainly enriched in the PI3K/AKT pathway but also in the focal adhesion, MAPK, TNF, and ECM processes. We then examined the expression levels of PI3K/AKT-related proteins and apoptosis-related proteins. Knocking down KIF21B results in the downregulation of PI3K/AKT-related proteins and Bcl-2 and the upregulation of Bax. In addition, PI3K/AKT pathway agonists can reverse the regulatory effect of KIF21B on the proliferation and apoptosis of osteosarcoma cells.

Prior to this study, there was no clear evidence showing a relationship between KIF21B and PI3K/AKT. The PI3K/AKT signaling pathway has been shown to play a role in the regulation of kinesin protein (32, 33), but the detailed mechanism by which the PI3K/AKT pathway regulates kinesin is currently rarely studied. On the other hand, according to the results of the gene chip and bioinformatics, cell adhesion and ECM processes were also enriched in KIF21B-silenced cells. Since ECM processes and cell adhesion can have a broad relationship with the PI3K/AKT pathway (34–37), we hypothesize that the mechanism by which KIF21B regulates osteosarcoma cells through the PI3K/AKT pathway is probably related to ECM processes and cell adhesion.

It should be noted that there are still several limitations in this study. First, we strictly selected participants to control for potential heterogeneity, which led to a small number of patients (17 patients). Second, the research on the cellular functions and molecular mechanisms of KIF21B was not comprehensive, and further studies are necessary to verify the role of KIF21B in the development and progression of OS. In summary, this experiment first confirmed that KIF21B, as an oncogene, plays an important role in the occurrence and development of OS. KIF21B silencing can also lead to the inhibition of cell proliferation and the induction of apoptosis. The mechanism by which KIF21B regulates OS might be associated with the persistent activation of the PI3K/AKT signaling pathway. However, the basic and clinical research investigating the role of KIF21B is still in the initial stages, and the molecular mechanism involved in the occurrence and development of OS warrants further study.

## Data Availability Statement

The datasets presented in this study can be found in online repositories. The names of the repository/repositories and accession number(s) can be found in the article/**Supplementary Material**.

## Ethics Statement

The studies involving human participants were reviewed and approved by the Ethics Committee of the Zhujiang Hospital of Southern Medical University. The patients/participants provided their written informed consent to participate in this study. The animal study was reviewed and approved by the Ethics Committee of the Zhujiang Hospital of Southern Medical University. Written informed consent was obtained from the owners for the participation of their animals in this study.

## Author Contributions

YD: Conceptualization, investigation, methodology, project administration, resources. SN and JL: Writing—review and editing, data curation, formal analysis, writing—original draft. SQ, KG, and YX: Formal analysis. All authors contributed to the article and approved the submitted version.

## Conflict of Interest

The authors declare that the research was conducted in the absence of any commercial or financial relationships that could be construed as a potential conflict of interest.

## Supplementary Material

Supplementary Figure 1 I Principal component analysis (PCA) of abnormal samples.
