# Supplementary material for: KIF21B Expression in Osteosarcoma and Its Regulatory Effect on Osteosarcoma Cell Proliferation and Apoptosis Through the PI3K/AKT Pathway
Source: Front Oncol. 2021 Jan 28;10:606765. doi: 10.3389/fonc.2020.606765 (PMC7879035; doi:10.3389/fonc.2020.606765)
Supplement: Supplementary file 2 [file Table_1.docx]

Supplementary Table 1 ROC curve analysis for evaluating the diagnostic value of differentially expressed genes

| Symbol | AUC |
| --- | --- |
| A2M | 1 |
| A3GALT2 | 1 |
| AADACL4 | 1 |
| AAED1 | 1 |
| AAMDC | 1 |
| AAMP | 1 |
| AARD | 1 |
| AARS | 1 |
| AASDHPPT | 1 |
| AATF | 1 |
| ABCA10 | 1 |
| ABCA5 | 1 |
| ABCA6 | 1 |
| ABCB1 | 1 |
| ABCB4 | 1 |
| ABCB5 | 1 |
| ABCB8 | 1 |
| ABCB9 | 1 |
| ABCC1 | 1 |
| ABCC12 | 1 |
| ABCC13 | 1 |
| ABCC5 | 1 |
| ABCC8 | 1 |
| ABCC9 | 1 |
| ABCD4 | 1 |
| ABCE1 | 1 |
| ABCF2 | 1 |
| ABCF3 | 1 |
| ABCG1 | 1 |
| ABCG2 | 1 |
| ABHD10 | 1 |
| ABHD12 | 1 |
| ABHD14B | 1 |
| ABHD15 | 1 |
| ABHD17B | 1 |
| ABHD17C | 1 |
| ABHD2 | 1 |
| ABHD3 | 1 |
| ABHD5 | 1 |
| ABHD6 | 1 |
| ABI3 | 1 |
| ABI3BP | 1 |
| ABL1 | 1 |
| ABL2 | 1 |
| ABLIM3 | 1 |
| ABR | 1 |
| ACAA1 | 1 |
| ACACB | 1 |
| ACAD10 | 1 |
| ACAD9 | 1 |
| ACADVL | 1 |
| ACAN | 1 |
| ACAT1 | 1 |
| ACAT2 | 1 |
| ACD | 1 |
| ACIN1 | 1 |
| ACKR1 | 1 |
| ACKR2 | 1 |
| ACKR3 | 1 |
| ACLY | 1 |
| ACO1 | 1 |
| ACO2 | 1 |
| ACOT13 | 1 |
| ACOT4 | 1 |
| ACOT7 | 1 |
| ACOT8 | 1 |
| ACOT9 | 1 |
| ACOX2 | 1 |
| ACP2 | 1 |
| ACPP | 1 |
| ACPT | 1 |
| ACRBP | 1 |
| ACRV1 | 1 |
| ACSL3 | 1 |
| ACSL4 | 1 |
| ACSL5 | 1 |
| ACSM6 | 1 |
| ACSS1 | 1 |
| ACTA2 | 1 |
| ACTB | 1 |
| ACTG1 | 1 |
| ACTL8 | 1 |
| ACTN1 | 1 |
| ACTN4 | 1 |
| ACTR10 | 1 |
| ACTR1A | 1 |
| ACTR2 | 1 |
| ACTR3 | 1 |
| ACTR3B | 1 |
| ACTR3C | 1 |
| ACTRT3 | 1 |
| ACVR1C | 1 |
| ACVR2B | 1 |
| ACVRL1 | 1 |
| ADAM10 | 1 |
| ADAM19 | 1 |
| ADAM2 | 1 |
| ADAM22 | 1 |
| ADAM28 | 1 |
| ADAM9 | 1 |
| ADAMDEC1 | 1 |
| ADAMTS1 | 1 |
| ADAMTS10 | 1 |
| ADAMTS15 | 1 |
| ADAMTS17 | 1 |
| ADAMTS18 | 1 |
| ADAMTS2 | 1 |
| ADAMTS9 | 1 |
| ADAMTSL1 | 1 |
| ADAMTSL5 | 1 |
| ADAP1 | 1 |
| ADAP2 | 1 |
| ADAR | 1 |
| ADARB1 | 1 |
| ADAT3 | 1 |
| ADCK1 | 1 |
| ADCY1 | 1 |
| ADCY10 | 1 |
| ADCY10P1 | 1 |
| ADCY2 | 1 |
| ADCY5 | 1 |
| ADCY7 | 1 |
| ADCY9 | 1 |
| ADD1 | 1 |
| ADD3 | 1 |
| ADGB | 1 |
| ADH1A | 1 |
| ADH5 | 1 |
| ADH7 | 1 |
| ADHFE1 | 1 |
| ADI1 | 1 |
| ADIRF | 1 |
| ADK | 1 |
| ADM | 1 |
| ADNP | 1 |
| ADORA3 | 1 |
| ADRA1A | 1 |
| ADRA1D | 1 |
| ADRA2A | 1 |
| ADRB2 | 1 |
| ADRBK2 | 1 |
| ADRM1 | 1 |
| ADSL | 1 |
| ADSS | 1 |
| ADTRP | 1 |
| AEN | 1 |
| AES | 1 |
| AFAP1.AS1 | 1 |
| AFAP1L1 | 1 |
| AFAP1L2 | 1 |
| AFF1 | 1 |
| AFF2 | 1 |
| AFG3L1P | 1 |
| AFM | 1 |
| AGAP1 | 1 |
| AGBL3 | 1 |
| AGBL4 | 1 |
| AGFG1 | 1 |
| AGFG2 | 1 |
| AGO3 | 1 |
| AGPAT1 | 1 |
| AGPAT2 | 1 |
| AGPAT3 | 1 |
| AGPAT4.IT1 | 1 |
| AGRN | 1 |
| AGT | 1 |
| AGTPBP1 | 1 |
| AHCYL1 | 1 |
| AHDC1 | 1 |
| AHI1 | 1 |
| AHNAK | 1 |
| AHNAK2 | 1 |
| AHR | 1 |
| AHSA1 | 1 |
| AHSA2 | 1 |
| AIDA | 1 |
| AIF1 | 1 |
| AIFM1 | 1 |
| AIFM2 | 1 |
| AIG1 | 1 |
| AIM2 | 1 |
| AIMP1 | 1 |
| AIP | 1 |
| AIPL1 | 1 |
| AJUBA | 1 |
| AK1 | 1 |
| AK2 | 1 |
| AK3 | 1 |
| AK5 | 1 |
| AK6 | 1 |
| AK7 | 1 |
| AKAP11 | 1 |
| AKAP12 | 1 |
| AKAP4 | 1 |
| AKAP6 | 1 |
| AKIRIN2 | 1 |
| AKNA | 1 |
| AKNAD1 | 1 |
| AKR1A1 | 1 |
| AKR1B1 | 1 |
| AKR1C3 | 1 |
| AKR7A2 | 1 |
| AKT1 | 1 |
| AKT1S1 | 1 |
| AKT2 | 1 |
| ALAS1 | 1 |
| ALCAM | 1 |
| ALDH18A1 | 1 |
| ALDH1A1 | 1 |
| ALDH1B1 | 1 |
| ALDH3B1 | 1 |
| ALDH7A1 | 1 |
| ALDH9A1 | 1 |
| ALG12 | 1 |
| ALG8 | 1 |
| ALG9 | 1 |
| ALKBH3 | 1 |
| ALKBH4 | 1 |
| ALKBH6 | 1 |
| ALKBH7 | 1 |
| ALMS1 | 1 |
| ALOX5 | 1 |
| ALOX5AP | 1 |
| ALPK2 | 1 |
| ALPK3 | 1 |
| ALPL | 1 |
| ALS2CR11 | 1 |
| ALS2CR12 | 1 |
| ALYREF | 1 |
| AMBRA1 | 1 |
| AMD1 | 1 |
| AMDHD2 | 1 |
| AMELX | 1 |
| AMER1 | 1 |
| AMER2 | 1 |
| AMFR | 1 |
| AMHR2 | 1 |
| AMICA1 | 1 |
| AMMECR1 | 1 |
| AMOT | 1 |
| AMOTL1 | 1 |
| AMOTL2 | 1 |
| AMT | 1 |
| AMTN | 1 |
| AMY2B | 1 |
| AMZ2 | 1 |
| ANAPC11 | 1 |
| ANAPC13 | 1 |
| ANAPC15 | 1 |
| ANAPC7 | 1 |
| ANG | 1 |
| ANGEL1 | 1 |
| ANGPT2 | 1 |
| ANGPTL5 | 1 |
| ANKAR | 1 |
| ANKDD1B | 1 |
| ANKFN1 | 1 |
| ANKFY1 | 1 |
| ANKH | 1 |
| ANKIB1 | 1 |
| ANKK1 | 1 |
| ANKLE2 | 1 |
| ANKMY1 | 1 |
| ANKRD1 | 1 |
| ANKRD10 | 1 |
| ANKRD13A | 1 |
| ANKRD18DP | 1 |
| ANKRD19P | 1 |
| ANKRD22 | 1 |
| ANKRD23 | 1 |
| ANKRD28 | 1 |
| ANKRD29 | 1 |
| ANKRD33B | 1 |
| ANKRD36 | 1 |
| ANKRD36B | 1 |
| ANKRD40 | 1 |
| ANKRD44 | 1 |
| ANKRD54 | 1 |
| ANKS3 | 1 |
| ANLN | 1 |
| ANO1 | 1 |
| ANO10 | 1 |
| ANO5 | 1 |
| ANO7 | 1 |
| ANP32A.IT1 | 1 |
| ANP32A | 1 |
| ANPEP | 1 |
| ANXA1 | 1 |
| ANXA11 | 1 |
| ANXA2 | 1 |
| ANXA2P2 | 1 |
| ANXA4 | 1 |
| ANXA5 | 1 |
| ANXA6 | 1 |
| ANXA7 | 1 |
| AOAH | 1 |
| AOC2 | 1 |
| AOC3 | 1 |
| AOX1 | 1 |
| AP1G1 | 1 |
| AP1G2 | 1 |
| AP1M1 | 1 |
| AP1S1 | 1 |
| AP1S3 | 1 |
| AP2A1 | 1 |
| AP2B1 | 1 |
| AP2M1 | 1 |
| AP2S1 | 1 |
| AP3B2 | 1 |
| AP3D1 | 1 |
| AP3M1 | 1 |
| AP4M1 | 1 |
| AP4S1 | 1 |
| AP5S1 | 1 |
| APCDD1 | 1 |
| APCDD1L | 1 |
| APEH | 1 |
| APEX1 | 1 |
| APH1B | 1 |
| APLN | 1 |
| APLNR | 1 |
| APLP2 | 1 |
| APMAP | 1 |
| APOA5 | 1 |
| APOBEC2 | 1 |
| APOBEC4 | 1 |
| APOC1 | 1 |
| APOC2 | 1 |
| APOC4 | 1 |
| APOE | 1 |
| APOL4 | 1 |
| APOLD1 | 1 |
| APOM | 1 |
| APOPT1 | 1 |
| APPBP2 | 1 |
| APPL2 | 1 |
| APRT | 1 |
| AQP10 | 1 |
| AQP11 | 1 |
| ARAFP2 | 1 |
| ARAP3 | 1 |
| ARC | 1 |
| ARCN1 | 1 |
| AREG | 1 |
| ARF3 | 1 |
| ARF4 | 1 |
| ARF5 | 1 |
| ARF6 | 1 |
| ARFGAP2 | 1 |
| ARFGAP3 | 1 |
| ARFIP1 | 1 |
| ARFIP2 | 1 |
| ARG1 | 1 |
| ARG2 | 1 |
| ARHGAP1 | 1 |
| ARHGAP11A | 1 |
| ARHGAP17 | 1 |
| ARHGAP21 | 1 |
| ARHGAP22 | 1 |
| ARHGAP25 | 1 |
| ARHGAP26 | 1 |
| ARHGAP27 | 1 |
| ARHGAP28 | 1 |
| ARHGAP29 | 1 |
| ARHGAP30 | 1 |
| ARHGAP35 | 1 |
| ARHGAP4 | 1 |
| ARHGAP44 | 1 |
| ARHGAP6 | 1 |
| ARHGAP9 | 1 |
| ARHGDIA | 1 |
| ARHGDIB | 1 |
| ARHGEF11 | 1 |
| ARHGEF28 | 1 |
| ARHGEF37 | 1 |
| ARHGEF6 | 1 |
| ARHGEF7 | 1 |
| ARHGEF9 | 1 |
| ARID1A | 1 |
| ARID1B | 1 |
| ARID3B | 1 |
| ARID5A | 1 |
| ARID5B | 1 |
| ARL1 | 1 |
| ARL11 | 1 |
| ARL15 | 1 |
| ARL2 | 1 |
| ARL2BP | 1 |
| ARL6IP1 | 1 |
| ARL6IP4 | 1 |
| ARL6IP5 | 1 |
| ARL8B | 1 |
| ARMC2 | 1 |
| ARMC6 | 1 |
| ARMC9 | 1 |
| ARMCX1 | 1 |
| ARMCX2 | 1 |
| ARMCX6 | 1 |
| ARNTL2 | 1 |
| ARPC1A | 1 |
| ARPC1B | 1 |
| ARPC2 | 1 |
| ARPC3 | 1 |
| ARPC4 | 1 |
| ARPC5 | 1 |
| ARPP19 | 1 |
| ARSB | 1 |
| ARSD | 1 |
| ARSJ | 1 |
| ARTN | 1 |
| ARV1 | 1 |
| ASAP2 | 1 |
| ASB1 | 1 |
| ASB10 | 1 |
| ASB13 | 1 |
| ASB14 | 1 |
| ASB15 | 1 |
| ASB5 | 1 |
| ASB6 | 1 |
| ASCC3 | 1 |
| ASF1B | 1 |
| ASH1L | 1 |
| ASH2L | 1 |
| ASIC3 | 1 |
| ASIC5 | 1 |
| ASL | 1 |
| ASMTL.AS1 | 1 |
| ASNA1 | 1 |
| ASNSD1 | 1 |
| ASPDH | 1 |
| ASPH | 1 |
| ASPHD2 | 1 |
| ASPRV1 | 1 |
| ASRGL1 | 1 |
| ASS1 | 1 |
| ASTN1 | 1 |
| ATAD2B | 1 |
| ATF1 | 1 |
| ATF3 | 1 |
| ATF4 | 1 |
| ATG101 | 1 |
| ATG12 | 1 |
| ATG13 | 1 |
| ATG16L2 | 1 |
| ATG3 | 1 |
| ATG5 | 1 |
| ATG9A | 1 |
| ATHL1 | 1 |
| ATIC | 1 |
| ATL1 | 1 |
| ATL3 | 1 |
| ATOH8 | 1 |
| ATP11A | 1 |
| ATP1B2 | 1 |
| ATP2A2 | 1 |
| ATP2B4 | 1 |
| ATP4B | 1 |
| ATP5A1 | 1 |
| ATP5B | 1 |
| ATP5C1 | 1 |
| ATP5D | 1 |
| ATP5G1 | 1 |
| ATP5H | 1 |
| ATP5J | 1 |
| ATP5J2.PTCD1 | 1 |
| ATP5L2 | 1 |
| ATP5S | 1 |
| ATP5SL | 1 |
| ATP6AP1 | 1 |
| ATP6AP1L | 1 |
| ATP6V0A2 | 1 |
| ATP6V0C | 1 |
| ATP6V0D1 | 1 |
| ATP6V0E1 | 1 |
| ATP6V1B1 | 1 |
| ATP6V1D | 1 |
| ATP6V1G2 | 1 |
| ATP6V1H | 1 |
| ATP8A2 | 1 |
| ATP8B1 | 1 |
| ATP8B3 | 1 |
| ATP8B4 | 1 |
| ATXN10 | 1 |
| ATXN3 | 1 |
| ATXN3L | 1 |
| AURKA | 1 |
| AURKAIP1 | 1 |
| AUTS2 | 1 |
| AVEN | 1 |
| AVIL | 1 |
| AVPI1 | 1 |
| AVPR1A | 1 |
| AWAT2 | 1 |
| AXDND1 | 1 |
| AXL | 1 |
| AZI2 | 1 |
| AZIN1 | 1 |
| AZIN2 | 1 |
| B3GALT5 | 1 |
| B3GAT3 | 1 |
| B3GNT1 | 1 |
| B3GNT5 | 1 |
| B3GNT9 | 1 |
| B3GNTL1 | 1 |
| B4GALNT3 | 1 |
| B4GALT1 | 1 |
| B4GALT2 | 1 |
| B4GALT4 | 1 |
| B4GALT7 | 1 |
| B9D2 | 1 |
| BABAM1 | 1 |
| BACH1 | 1 |
| BACH2 | 1 |
| BAG2 | 1 |
| BAG3 | 1 |
| BAG6 | 1 |
| BAI3 | 1 |
| BAK1 | 1 |
| BANF1 | 1 |
| BANK1 | 1 |
| BAP1 | 1 |
| BARX1 | 1 |
| BAX | 1 |
| BAZ2B | 1 |
| BBS2 | 1 |
| BBS4 | 1 |
| BCAM | 1 |
| BCAP31 | 1 |
| BCAR1 | 1 |
| BCAS2 | 1 |
| BCAT2 | 1 |
| BCCIP | 1 |
| BCDIN3D | 1 |
| BCKDK | 1 |
| BCL10 | 1 |
| BCL2 | 1 |
| BCL2L1 | 1 |
| BCL2L11 | 1 |
| BCL2L14 | 1 |
| BCL2L15 | 1 |
| BCL6B | 1 |
| BCL7B | 1 |
| BCL7C | 1 |
| BCO2 | 1 |
| BCOR | 1 |
| BCORL1 | 1 |
| BDH1 | 1 |
| BDKRB1 | 1 |
| BEND5 | 1 |
| BET1 | 1 |
| BET1L | 1 |
| BEX4 | 1 |
| BFAR | 1 |
| BFSP1 | 1 |
| BGN | 1 |
| BHLHE22 | 1 |
| BHLHE23 | 1 |
| BHLHE41 | 1 |
| BICD2 | 1 |
| BID | 1 |
| BIN2 | 1 |
| BIN3 | 1 |
| BIRC3 | 1 |
| BIRC5 | 1 |
| BIRC6 | 1 |
| BIVM | 1 |
| BLCAP | 1 |
| BLNK | 1 |
| BLOC1S1 | 1 |
| BLOC1S2 | 1 |
| BLOC1S4 | 1 |
| BLOC1S6 | 1 |
| BLVRB | 1 |
| BMF | 1 |
| BMP15 | 1 |
| BMP2 | 1 |
| BMP2K | 1 |
| BMP3 | 1 |
| BMP7 | 1 |
| BMP8A | 1 |
| BMP8B | 1 |
| BMPER | 1 |
| BNC1 | 1 |
| BNIP2 | 1 |
| BNIP3L | 1 |
| BOD1 | 1 |
| BOK | 1 |
| BOLA1 | 1 |
| BOLA3 | 1 |
| BPGM | 1 |
| BPHL | 1 |
| BPIFA4P | 1 |
| BPIFC | 1 |
| BRAP | 1 |
| BRD8 | 1 |
| BRE | 1 |
| BRI3 | 1 |
| BRI3BP | 1 |
| BRINP1 | 1 |
| BRINP2 | 1 |
| BRINP3 | 1 |
| BRIX1 | 1 |
| BRK1 | 1 |
| BROX | 1 |
| BRWD1 | 1 |
| BRWD3 | 1 |
| BSG | 1 |
| BSN | 1 |
| BST1 | 1 |
| BST2 | 1 |
| BTAF1 | 1 |
| BTBD10 | 1 |
| BTBD16 | 1 |
| BTBD2 | 1 |
| BTC | 1 |
| BTD | 1 |
| BTF3 | 1 |
| BTK | 1 |
| BTLA | 1 |
| BTN2A3P | 1 |
| BTNL8 | 1 |
| BUB3 | 1 |
| BUD31 | 1 |
| C10orf107 | 1 |
| C10orf11 | 1 |
| C10orf32 | 1 |
| C10orf35 | 1 |
| C10orf90 | 1 |
| C10orf91 | 1 |
| C11orf1 | 1 |
| C11orf24 | 1 |
| C11orf30 | 1 |
| C11orf42 | 1 |
| C11orf44 | 1 |
| C11orf45 | 1 |
| C11orf58 | 1 |
| C11orf65 | 1 |
| C11orf68 | 1 |
| C11orf70 | 1 |
| C11orf71 | 1 |
| C11orf72 | 1 |
| C11orf73 | 1 |
| C11orf74 | 1 |
| C11orf83 | 1 |
| C11orf84 | 1 |
| C11orf85 | 1 |
| C11orf87 | 1 |
| C11orf88 | 1 |
| C12orf10 | 1 |
| C12orf40 | 1 |
| C12orf42 | 1 |
| C12orf43 | 1 |
| C12orf5 | 1 |
| C12orf54 | 1 |
| C12orf65 | 1 |
| C12orf75 | 1 |
| C14orf1 | 1 |
| C14orf119 | 1 |
| C14orf166 | 1 |
| C14orf180 | 1 |
| C14orf183 | 1 |
| C15orf26 | 1 |
| C15orf38.AP3S2 | 1 |
| C15orf48 | 1 |
| C15orf52 | 1 |
| C15orf61 | 1 |
| C16orf13 | 1 |
| C16orf3 | 1 |
| C16orf45 | 1 |
| C16orf54 | 1 |
| C16orf62 | 1 |
| C16orf72 | 1 |
| C16orf80 | 1 |
| C16orf91 | 1 |
| C17orf47 | 1 |
| C17orf49 | 1 |
| C17orf64 | 1 |
| C17orf97 | 1 |
| C19orf10 | 1 |
| C19orf18 | 1 |
| C19orf24 | 1 |
| C19orf25 | 1 |
| C19orf33 | 1 |
| C19orf40 | 1 |
| C19orf43 | 1 |
| C19orf52 | 1 |
| C19orf53 | 1 |
| C19orf60 | 1 |
| C19orf70 | 1 |
| C19orf73 | 1 |
| C1GALT1 | 1 |
| C1GALT1C1 | 1 |
| C1orf101 | 1 |
| C1orf105 | 1 |
| C1orf110 | 1 |
| C1orf111 | 1 |
| C1orf115 | 1 |
| C1orf122 | 1 |
| C1orf123 | 1 |
| C1orf131 | 1 |
| C1orf140 | 1 |
| C1orf146 | 1 |
| C1orf158 | 1 |
| C1orf162 | 1 |
| C1orf180 | 1 |
| C1orf200 | 1 |
| C1orf213 | 1 |
| C1orf216 | 1 |
| C1orf220 | 1 |
| C1orf35 | 1 |
| C1orf43 | 1 |
| C1orf52 | 1 |
| C1orf53 | 1 |
| C1orf68 | 1 |
| C1orf74 | 1 |
| C1orf85 | 1 |
| C1QA | 1 |
| C1QB | 1 |
| C1QBP | 1 |
| C1QC | 1 |
| C1QL2 | 1 |
| C1QTNF2 | 1 |
| C1QTNF3 | 1 |
| C1QTNF7 | 1 |
| C1QTNF9B.AS1 | 1 |
| C1R | 1 |
| C1RL | 1 |
| C1S | 1 |
| C20orf197 | 1 |
| C20orf78 | 1 |
| C20orf96 | 1 |
| C21orf49 | 1 |
| C21orf67 | 1 |
| C21orf91 | 1 |
| C22orf15 | 1 |
| C22orf23 | 1 |
| C22orf29 | 1 |
| C22orf31 | 1 |
| C2CD2L | 1 |
| C2CD3 | 1 |
| C2orf27A | 1 |
| C2orf44 | 1 |
| C2orf48 | 1 |
| C2orf50 | 1 |
| C2orf66 | 1 |
| C2orf73 | 1 |
| C2orf78 | 1 |
| C3AR1 | 1 |
| C3orf14 | 1 |
| C3orf18 | 1 |
| C3orf20 | 1 |
| C3orf22 | 1 |
| C3orf35 | 1 |
| C3orf36 | 1 |
| C3orf38 | 1 |
| C3orf55 | 1 |
| C3orf58 | 1 |
| C3orf70 | 1 |
| C4BPB | 1 |
| C4orf22 | 1 |
| C4orf26 | 1 |
| C4orf3 | 1 |
| C4orf45 | 1 |
| C4orf46 | 1 |
| C5AR1 | 1 |
| C5orf24 | 1 |
| C5orf30 | 1 |
| C5orf42 | 1 |
| C6 | 1 |
| C6orf1 | 1 |
| C6orf123 | 1 |
| C6orf141 | 1 |
| C6orf163 | 1 |
| C6orf211 | 1 |
| C6orf25 | 1 |
| C6orf52 | 1 |
| C6orf89 | 1 |
| C7orf26 | 1 |
| C7orf31 | 1 |
| C7orf33 | 1 |
| C7orf34 | 1 |
| C7orf43 | 1 |
| C7orf50 | 1 |
| C7orf60 | 1 |
| C7orf61 | 1 |
| C7orf66 | 1 |
| C7orf69 | 1 |
| C7orf76 | 1 |
| C8B | 1 |
| C8orf31 | 1 |
| C8orf4 | 1 |
| C8orf46 | 1 |
| C8orf48 | 1 |
| C8orf58 | 1 |
| C8orf59 | 1 |
| C9orf114 | 1 |
| C9orf152 | 1 |
| C9orf153 | 1 |
| C9orf24 | 1 |
| C9orf43 | 1 |
| C9orf53 | 1 |
| C9orf57 | 1 |
| C9orf64 | 1 |
| C9orf66 | 1 |
| C9orf78 | 1 |
| C9orf9 | 1 |
| C9orf91 | 1 |
| CA1 | 1 |
| CA10 | 1 |
| CA12 | 1 |
| CA14 | 1 |
| CA6 | 1 |
| CAB39 | 1 |
| CAB39L | 1 |
| CABP4 | 1 |
| CACFD1 | 1 |
| CACNA1D | 1 |
| CACNA1F | 1 |
| CACNA2D1 | 1 |
| CACNA2D3 | 1 |
| CACNB2 | 1 |
| CACNG5 | 1 |
| CACTIN | 1 |
| CACYBP | 1 |
| CADM1 | 1 |
| CADM2 | 1 |
| CAGE1 | 1 |
| CALCR | 1 |
| CALCRL | 1 |
| CALD1 | 1 |
| CALHM2 | 1 |
| CALM1 | 1 |
| CALM3 | 1 |
| CALML4 | 1 |
| CALML5 | 1 |
| CALR | 1 |
| CALU | 1 |
| CALY | 1 |
| CAMKK1 | 1 |
| CAMKMT | 1 |
| CAMTA1 | 1 |
| CAND2 | 1 |
| CANT1 | 1 |
| CANX | 1 |
| CAP1 | 1 |
| CAP2 | 1 |
| CAPG | 1 |
| CAPN1 | 1 |
| CAPN13 | 1 |
| CAPN2 | 1 |
| CAPN3 | 1 |
| CAPN7 | 1 |
| CAPNS1 | 1 |
| CAPRIN1 | 1 |
| CAPZA2 | 1 |
| CAPZB | 1 |
| CARD10 | 1 |
| CARD6 | 1 |
| CARD8 | 1 |
| CARHSP1 | 1 |
| CARKD | 1 |
| CARM1 | 1 |
| CARS | 1 |
| CARS2 | 1 |
| CASC4 | 1 |
| CASKIN1 | 1 |
| CASKIN2 | 1 |
| CASP10 | 1 |
| CASP12 | 1 |
| CASP5 | 1 |
| CASP6 | 1 |
| CASQ2 | 1 |
| CASS4 | 1 |
| CAV1 | 1 |
| CAV2 | 1 |
| CBFA2T2 | 1 |
| CBFB | 1 |
| CBL | 1 |
| CBX3 | 1 |
| CBX4 | 1 |
| CBX5 | 1 |
| CBX6 | 1 |
| CBY1 | 1 |
| CC2D1B | 1 |
| CC2D2A | 1 |
| CCAR2 | 1 |
| CCBE1 | 1 |
| CCBL1 | 1 |
| CCDC102B | 1 |
| CCDC104 | 1 |
| CCDC106 | 1 |
| CCDC107 | 1 |
| CCDC109B | 1 |
| CCDC11 | 1 |
| CCDC110 | 1 |
| CCDC117 | 1 |
| CCDC12 | 1 |
| CCDC124 | 1 |
| CCDC138 | 1 |
| CCDC14 | 1 |
| CCDC141 | 1 |
| CCDC148 | 1 |
| CCDC150 | 1 |
| CCDC169 | 1 |
| CCDC17 | 1 |
| CCDC170 | 1 |
| CCDC177 | 1 |
| CCDC180 | 1 |
| CCDC184 | 1 |
| CCDC22 | 1 |
| CCDC23 | 1 |
| CCDC25 | 1 |
| CCDC26 | 1 |
| CCDC3 | 1 |
| CCDC30 | 1 |
| CCDC37 | 1 |
| CCDC39 | 1 |
| CCDC51 | 1 |
| CCDC53 | 1 |
| CCDC57 | 1 |
| CCDC61 | 1 |
| CCDC67 | 1 |
| CCDC71L | 1 |
| CCDC80 | 1 |
| CCDC84 | 1 |
| CCDC85A | 1 |
| CCDC85B | 1 |
| CCDC87 | 1 |
| CCDC88A | 1 |
| CCDC88C | 1 |
| CCDC89 | 1 |
| CCDC92 | 1 |
| CCER1 | 1 |
| CCK | 1 |
| CCL13 | 1 |
| CCL14 | 1 |
| CCL16 | 1 |
| CCL18 | 1 |
| CCL2 | 1 |
| CCL22 | 1 |
| CCL25 | 1 |
| CCL26 | 1 |
| CCL4 | 1 |
| CCL4L2 | 1 |
| CCL5 | 1 |
| CCL8 | 1 |
| CCNA2 | 1 |
| CCNB1 | 1 |
| CCNB2 | 1 |
| CCNC | 1 |
| CCND1 | 1 |
| CCNDBP1 | 1 |
| CCNF | 1 |
| CCNG1 | 1 |
| CCNG2 | 1 |
| CCNI2 | 1 |
| CCNJ | 1 |
| CCNL2 | 1 |
| CCNT2 | 1 |
| CCNYL1 | 1 |
| CCP110 | 1 |
| CCPG1 | 1 |
| CCR1 | 1 |
| CCR2 | 1 |
| CCR8 | 1 |
| CCRL2 | 1 |
| CCSAP | 1 |
| CCSER1 | 1 |
| CCT2 | 1 |
| CCT4 | 1 |
| CCT5 | 1 |
| CCT6A | 1 |
| CCT7 | 1 |
| CD109 | 1 |
| CD14 | 1 |
| CD151 | 1 |
| CD163 | 1 |
| CD163L1 | 1 |
| CD164 | 1 |
| CD180 | 1 |
| CD1A | 1 |
| CD1E | 1 |
| CD2 | 1 |
| CD200 | 1 |
| CD200R1 | 1 |
| CD28 | 1 |
| CD2BP2 | 1 |
| CD300A | 1 |
| CD300C | 1 |
| CD300LF | 1 |
| CD34 | 1 |
| CD36 | 1 |
| CD37 | 1 |
| CD38 | 1 |
| CD3EAP | 1 |
| CD4 | 1 |
| CD40 | 1 |
| CD44 | 1 |
| CD46 | 1 |
| CD48 | 1 |
| CD52 | 1 |
| CD53 | 1 |
| CD59 | 1 |
| CD63 | 1 |
| CD69 | 1 |
| CD74 | 1 |
| CD80 | 1 |
| CD81 | 1 |
| CD83 | 1 |
| CD84 | 1 |
| CD86 | 1 |
| CD8A | 1 |
| CD9 | 1 |
| CD93 | 1 |
| CD96 | 1 |
| CD97 | 1 |
| CD99 | 1 |
| CD99L2 | 1 |
| CDA | 1 |
| CDC123 | 1 |
| CDC14A | 1 |
| CDC16 | 1 |
| CDC20 | 1 |
| CDC20B | 1 |
| CDC23 | 1 |
| CDC25B | 1 |
| CDC26 | 1 |
| CDC27 | 1 |
| CDC37 | 1 |
| CDC40 | 1 |
| CDC42 | 1 |
| CDC42EP2 | 1 |
| CDC42SE1 | 1 |
| CDC45 | 1 |
| CDC6 | 1 |
| CDC7 | 1 |
| CDCA2 | 1 |
| CDCA3 | 1 |
| CDCA4 | 1 |
| CDCA7L | 1 |
| CDCP1 | 1 |
| CDCP2 | 1 |
| CDH13 | 1 |
| CDH5 | 1 |
| CDH7 | 1 |
| CDHR3 | 1 |
| CDIP1 | 1 |
| CDIPT | 1 |
| CDK15 | 1 |
| CDK18 | 1 |
| CDK2 | 1 |
| CDK2AP1 | 1 |
| CDK3 | 1 |
| CDK5 | 1 |
| CDK6 | 1 |
| CDK7 | 1 |
| CDK8 | 1 |
| CDK9 | 1 |
| CDKN1A | 1 |
| CDKN2AIPNL | 1 |
| CDKN3 | 1 |
| CDON | 1 |
| CDPF1 | 1 |
| CDR1 | 1 |
| CDR2L | 1 |
| CDS1 | 1 |
| CDT1 | 1 |
| CDV3 | 1 |
| CDX1 | 1 |
| CDX2 | 1 |
| CDX4 | 1 |
| CEACAM1 | 1 |
| CEACAM20 | 1 |
| CEACAM8 | 1 |
| CEBPZ | 1 |
| CECR1 | 1 |
| CECR2 | 1 |
| CELF1 | 1 |
| CELF6 | 1 |
| CEMIP | 1 |
| CENPB | 1 |
| CENPBD1 | 1 |
| CENPBD1P1 | 1 |
| CENPC | 1 |
| CENPH | 1 |
| CENPJ | 1 |
| CENPM | 1 |
| CENPN | 1 |
| CENPW | 1 |
| CEP135 | 1 |
| CEP152 | 1 |
| CEP250 | 1 |
| CEP55 | 1 |
| CEP68 | 1 |
| CEP76 | 1 |
| CEP83 | 1 |
| CEP89 | 1 |
| CERCAM | 1 |
| CERS2 | 1 |
| CERS5 | 1 |
| CES2 | 1 |
| CETN2 | 1 |
| CETP | 1 |
| CFB | 1 |
| CFD | 1 |
| CFL1 | 1 |
| CFL2 | 1 |
| CHAC1 | 1 |
| CHAD | 1 |
| CHCHD1 | 1 |
| CHCHD2 | 1 |
| CHCHD3 | 1 |
| CHCHD5 | 1 |
| CHD2 | 1 |
| CHD6 | 1 |
| CHD7 | 1 |
| CHDH | 1 |
| CHEK2 | 1 |
| CHI3L1 | 1 |
| CHIC1 | 1 |
| CHID1 | 1 |
| CHKA | 1 |
| CHMP1A | 1 |
| CHMP2A | 1 |
| CHMP2B | 1 |
| CHMP3 | 1 |
| CHMP4A | 1 |
| CHMP4B | 1 |
| CHMP6 | 1 |
| CHMP7 | 1 |
| CHN2 | 1 |
| CHP1 | 1 |
| CHPF | 1 |
| CHPT1 | 1 |
| CHRDL2 | 1 |
| CHRM2 | 1 |
| CHRM3 | 1 |
| CHRM4 | 1 |
| CHRNA1 | 1 |
| CHRNA6 | 1 |
| CHRNA7 | 1 |
| CHRNB2 | 1 |
| CHRNB3 | 1 |
| CHST3 | 1 |
| CHST6 | 1 |
| CHTF18 | 1 |
| CHTF8 | 1 |
| CIAO1 | 1 |
| CIB1 | 1 |
| CIB2 | 1 |
| CIDEA | 1 |
| CIITA | 1 |
| CINP | 1 |
| CIRBP | 1 |
| CIRH1A | 1 |
| CISD1 | 1 |
| CISH | 1 |
| CIT | 1 |
| CITED2 | 1 |
| CIZ1 | 1 |
| CKAP2 | 1 |
| CKAP2L | 1 |
| CKAP4 | 1 |
| CKMT2 | 1 |
| CKS1B | 1 |
| CKS2 | 1 |
| CLCC1 | 1 |
| CLCF1 | 1 |
| CLCN2 | 1 |
| CLCN6 | 1 |
| CLCN7 | 1 |
| CLCNKA | 1 |
| CLDN11 | 1 |
| CLDN24 | 1 |
| CLDN5 | 1 |
| CLDN8 | 1 |
| CLEC14A | 1 |
| CLEC1A | 1 |
| CLEC2D | 1 |
| CLEC3A | 1 |
| CLEC3B | 1 |
| CLEC4A | 1 |
| CLEC4E | 1 |
| CLEC4G | 1 |
| CLEC5A | 1 |
| CLEC6A | 1 |
| CLEC7A | 1 |
| CLECL1 | 1 |
| CLIC1 | 1 |
| CLIC2 | 1 |
| CLIC3 | 1 |
| CLIC4 | 1 |
| CLIC5 | 1 |
| CLIC6 | 1 |
| CLINT1 | 1 |
| CLIP3 | 1 |
| CLIP4 | 1 |
| CLMN | 1 |
| CLMP | 1 |
| CLN3 | 1 |
| CLN5 | 1 |
| CLNK | 1 |
| CLOCK | 1 |
| CLP1 | 1 |
| CLPP | 1 |
| CLPTM1 | 1 |
| CLPTM1L | 1 |
| CLRN3 | 1 |
| CLSPN | 1 |
| CLTA | 1 |
| CLTB | 1 |
| CLTC.IT1 | 1 |
| CLTC | 1 |
| CLUAP1 | 1 |
| CLUH | 1 |
| CMAS | 1 |
| CMBL | 1 |
| CMC2 | 1 |
| CMC4 | 1 |
| CMKLR1 | 1 |
| CMPK1 | 1 |
| CMPK2 | 1 |
| CMSS1 | 1 |
| CMTM6 | 1 |
| CMTM8 | 1 |
| CMTR1 | 1 |
| CNBP | 1 |
| CNDP1 | 1 |
| CNEP1R1 | 1 |
| CNGA2 | 1 |
| CNIH4 | 1 |
| CNN2 | 1 |
| CNNM2 | 1 |
| CNOT3 | 1 |
| CNOT7 | 1 |
| CNP | 1 |
| CNPPD1 | 1 |
| CNPY3 | 1 |
| CNPY4 | 1 |
| CNST | 1 |
| CNTFR | 1 |
| CNTLN | 1 |
| CNTN2 | 1 |
| CNTN4 | 1 |
| CNTN6 | 1 |
| CNTNAP1 | 1 |
| CNTRL | 1 |
| COA3 | 1 |
| COA6 | 1 |
| COA7 | 1 |
| COASY | 1 |
| COG4 | 1 |
| COG5 | 1 |
| COG6 | 1 |
| COIL | 1 |
| COL10A1 | 1 |
| COL11A2 | 1 |
| COL14A1 | 1 |
| COL15A1 | 1 |
| COL22A1 | 1 |
| COL24A1 | 1 |
| COL28A1 | 1 |
| COL4A2 | 1 |
| COL4A3BP | 1 |
| COL4A4 | 1 |
| COL6A3 | 1 |
| COLEC11 | 1 |
| COLGALT1 | 1 |
| COMMD1 | 1 |
| COMMD2 | 1 |
| COMMD4 | 1 |
| COMMD6 | 1 |
| COMMD7 | 1 |
| COMMD8 | 1 |
| COMMD9 | 1 |
| COMP | 1 |
| COMT | 1 |
| COPA | 1 |
| COPB1 | 1 |
| COPB2 | 1 |
| COPE | 1 |
| COPG1 | 1 |
| COPRS | 1 |
| COPS2 | 1 |
| COPS4 | 1 |
| COPS6 | 1 |
| COPS7A | 1 |
| COPS8 | 1 |
| COPZ1 | 1 |
| COPZ2 | 1 |
| COQ10B | 1 |
| COQ2 | 1 |
| COQ3 | 1 |
| COQ7 | 1 |
| COQ9 | 1 |
| CORO1B | 1 |
| CORO1C | 1 |
| CORO7 | 1 |
| COTL1 | 1 |
| COX15 | 1 |
| COX19 | 1 |
| COX20 | 1 |
| COX6B1 | 1 |
| COX7A2 | 1 |
| COX7A2L | 1 |
| COX7B | 1 |
| COX7C | 1 |
| COX8A | 1 |
| COX8C | 1 |
| CPA4 | 1 |
| CPAMD8 | 1 |
| CPB2 | 1 |
| CPE | 1 |
| CPEB2 | 1 |
| CPEB3 | 1 |
| CPEB4 | 1 |
| CPED1 | 1 |
| CPLX3 | 1 |
| CPLX4 | 1 |
| CPN2 | 1 |
| CPNE3 | 1 |
| CPNE5 | 1 |
| CPOX | 1 |
| CPPED1 | 1 |
| CPS1 | 1 |
| CPSF2 | 1 |
| CPSF3 | 1 |
| CPSF7 | 1 |
| CPT1A | 1 |
| CPVL | 1 |
| CPXM1 | 1 |
| CR1 | 1 |
| CRADD | 1 |
| CRAT | 1 |
| CRCP | 1 |
| CREB3 | 1 |
| CREB3L4 | 1 |
| CREBL2 | 1 |
| CREBZF | 1 |
| CREM | 1 |
| CRIP2 | 1 |
| CRK | 1 |
| CRLF1 | 1 |
| CRLF2 | 1 |
| CRMP1 | 1 |
| CRNKL1 | 1 |
| CRNN | 1 |
| CROCCP2 | 1 |
| CRTAP | 1 |
| CRTC2 | 1 |
| CRY1 | 1 |
| CRYAB | 1 |
| CRYBA1 | 1 |
| CRYBA2 | 1 |
| CRYBA4 | 1 |
| CRYM.AS1 | 1 |
| CRYM | 1 |
| CS | 1 |
| CSAD | 1 |
| CSDE1 | 1 |
| CSE1L | 1 |
| CSF1 | 1 |
| CSF1R | 1 |
| CSF2 | 1 |
| CSF2RA | 1 |
| CSN1S2AP | 1 |
| CSNK1A1 | 1 |
| CSNK1D | 1 |
| CSNK1G2 | 1 |
| CSNK1G3 | 1 |
| CSNK2A2 | 1 |
| CSNK2B | 1 |
| CSPG4P5 | 1 |
| CSPP1 | 1 |
| CSRP1 | 1 |
| CSRP2 | 1 |
| CSRP3 | 1 |
| CST9 | 1 |
| CSTA | 1 |
| CSTB | 1 |
| CSTF1 | 1 |
| CT55 | 1 |
| CT83 | 1 |
| CTAG2 | 1 |
| CTAGE1 | 1 |
| CTAGE10P | 1 |
| CTBP2 | 1 |
| CTC1 | 1 |
| CTF1 | 1 |
| CTGF | 1 |
| CTHRC1 | 1 |
| CTNNA1 | 1 |
| CTNNA3 | 1 |
| CTNNAL1 | 1 |
| CTNS | 1 |
| CTR9 | 1 |
| CTRC | 1 |
| CTRL | 1 |
| CTSA | 1 |
| CTSB | 1 |
| CTSC | 1 |
| CTSG | 1 |
| CTSH | 1 |
| CTSL | 1 |
| CTSW | 1 |
| CTSZ | 1 |
| CTTN | 1 |
| CTTNBP2 | 1 |
| CTTNBP2NL | 1 |
| CTXN3 | 1 |
| CUBN | 1 |
| CUEDC2 | 1 |
| CUL1 | 1 |
| CUL3 | 1 |
| CUL7 | 1 |
| CUTC | 1 |
| CUX1 | 1 |
| CWF19L1 | 1 |
| CWF19L2 | 1 |
| CX3CL1 | 1 |
| CX3CR1 | 1 |
| CXCL1 | 1 |
| CXCL10 | 1 |
| CXCL11 | 1 |
| CXCL12 | 1 |
| CXCL14 | 1 |
| CXCL2 | 1 |
| CXCL3 | 1 |
| CXCL5 | 1 |
| CXCL8 | 1 |
| CXCL9 | 1 |
| CXCR4 | 1 |
| CXCR6 | 1 |
| CXorf21 | 1 |
| CXorf24 | 1 |
| CXorf36 | 1 |
| CXXC4 | 1 |
| CXXC5 | 1 |
| CYB561A3 | 1 |
| CYB561D2 | 1 |
| CYB5B | 1 |
| CYB5D2 | 1 |
| CYB5R1 | 1 |
| CYB5R3 | 1 |
| CYBA | 1 |
| CYBB | 1 |
| CYBRD1 | 1 |
| CYFIP1 | 1 |
| CYLC1 | 1 |
| CYP19A1 | 1 |
| CYP1B1 | 1 |
| CYP24A1 | 1 |
| CYP26A1 | 1 |
| CYP2C18 | 1 |
| CYP2C19 | 1 |
| CYP2C9 | 1 |
| CYP3A43 | 1 |
| CYP3A5 | 1 |
| CYP3A7 | 1 |
| CYP4F2 | 1 |
| CYP51A1 | 1 |
| CYP7B1 | 1 |
| CYR61 | 1 |
| CYSLTR1 | 1 |
| CYSTM1 | 1 |
| CYTH3 | 1 |
| CYTH4 | 1 |
| CYYR1 | 1 |
| DAB1 | 1 |
| DACH1 | 1 |
| DACT2 | 1 |
| DACT3 | 1 |
| DAD1 | 1 |
| DAG1 | 1 |
| DAGLB | 1 |
| DAK | 1 |
| DALRD3 | 1 |
| DAPK2 | 1 |
| DAPK3 | 1 |
| DAPL1 | 1 |
| DARS | 1 |
| DAW1 | 1 |
| DBIL5P2 | 1 |
| DBN1 | 1 |
| DBP | 1 |
| DBR1 | 1 |
| DCAF16 | 1 |
| DCAF4L1 | 1 |
| DCAF4L2 | 1 |
| DCBLD1 | 1 |
| DCBLD2 | 1 |
| DCD | 1 |
| DCHS1 | 1 |
| DCHS2 | 1 |
| DCK | 1 |
| DCLK2 | 1 |
| DCP2 | 1 |
| DCSTAMP | 1 |
| DCTD | 1 |
| DCTN1 | 1 |
| DCTN2 | 1 |
| DCTN3 | 1 |
| DCTN4 | 1 |
| DCTN5 | 1 |
| DCTN6 | 1 |
| DCTPP1 | 1 |
| DCUN1D3 | 1 |
| DCUN1D5 | 1 |
| DDA1 | 1 |
| DDAH1 | 1 |
| DDAH2 | 1 |
| DDB1 | 1 |
| DDB2 | 1 |
| DDI1 | 1 |
| DDOST | 1 |
| DDR2 | 1 |
| DDRGK1 | 1 |
| DDTL | 1 |
| DDX1 | 1 |
| DDX11 | 1 |
| DDX20 | 1 |
| DDX24 | 1 |
| DDX25 | 1 |
| DDX41 | 1 |
| DDX43 | 1 |
| DDX54 | 1 |
| DDX55 | 1 |
| DDX6 | 1 |
| 1-Dec | 1 |
| DECR1 | 1 |
| DECR2 | 1 |
| DEDD | 1 |
| DEDD2 | 1 |
| DEF8 | 1 |
| DEFB1 | 1 |
| DEFB103A | 1 |
| DEFB106B | 1 |
| DEFB112 | 1 |
| DEFB113 | 1 |
| DEFB116 | 1 |
| DEFB121 | 1 |
| DEFB123 | 1 |
| DEFB128 | 1 |
| DEFB132 | 1 |
| DEFT1P | 1 |
| DEGS1 | 1 |
| DEK | 1 |
| DENND2A | 1 |
| DENND4B | 1 |
| DENR | 1 |
| DEPDC1 | 1 |
| DERA | 1 |
| DERL1 | 1 |
| DERL2 | 1 |
| DESI1 | 1 |
| DFNA5 | 1 |
| DFNB31 | 1 |
| DGAT2L6 | 1 |
| DGCR11 | 1 |
| DGCR6L | 1 |
| DGKA | 1 |
| DGKD | 1 |
| DGKE | 1 |
| DGKZ | 1 |
| DHCR7 | 1 |
| DHDDS | 1 |
| DHPS | 1 |
| DHRS1 | 1 |
| DHRS4.AS1 | 1 |
| DHRS7 | 1 |
| DHRS9 | 1 |
| DHX29 | 1 |
| DHX38 | 1 |
| DHX8 | 1 |
| DIAPH1 | 1 |
| DIAPH3 | 1 |
| DICER1.AS1 | 1 |
| DICER1 | 1 |
| DIEXF | 1 |
| DIMT1 | 1 |
| DIO1 | 1 |
| DIO2 | 1 |
| DIP2A | 1 |
| DIRC1 | 1 |
| DIS3L2 | 1 |
| DISP1 | 1 |
| DKFZp434E1119 | 1 |
| DKFZP434F142 | 1 |
| DKK1 | 1 |
| DKKL1 | 1 |
| DLAT | 1 |
| DLD | 1 |
| DLEU1 | 1 |
| DLG1 | 1 |
| DLG4 | 1 |
| DLL1 | 1 |
| DLL4 | 1 |
| DLST | 1 |
| DLX1 | 1 |
| DLX4 | 1 |
| DLX6 | 1 |
| DMD | 1 |
| DMP1 | 1 |
| DMRT2 | 1 |
| DMRTB1 | 1 |
| DMTF1 | 1 |
| DMWD | 1 |
| DNAH1 | 1 |
| DNAH10 | 1 |
| DNAH12 | 1 |
| DNAH5 | 1 |
| DNAH6 | 1 |
| DNAH7 | 1 |
| DNAJA2 | 1 |
| DNAJA3 | 1 |
| DNAJB13 | 1 |
| DNAJB4 | 1 |
| DNAJB5 | 1 |
| DNAJB7 | 1 |
| DNAJB8 | 1 |
| DNAJC10 | 1 |
| DNAJC12 | 1 |
| DNAJC13 | 1 |
| DNAJC14 | 1 |
| DNAJC19 | 1 |
| DNAJC2 | 1 |
| DNAJC24 | 1 |
| DNAJC25.GNG10 | 1 |
| DNAJC30 | 1 |
| DNAJC7 | 1 |
| DNAJC8 | 1 |
| DNASE1L1 | 1 |
| DND1 | 1 |
| DNER | 1 |
| DNHD1 | 1 |
| DNM1L | 1 |
| DNM3 | 1 |
| DNMBP | 1 |
| DNMT1 | 1 |
| DNMT3A | 1 |
| DNTTIP1 | 1 |
| DOCK3 | 1 |
| DOCK4 | 1 |
| DOCK5 | 1 |
| DOCK6 | 1 |
| DOCK7 | 1 |
| DOCK8 | 1 |
| DOCK9 | 1 |
| DOK1 | 1 |
| DOK2 | 1 |
| DOK3 | 1 |
| DOK5 | 1 |
| DOLK | 1 |
| DOLPP1 | 1 |
| DPAGT1 | 1 |
| DPCD | 1 |
| DPF3 | 1 |
| DPH3 | 1 |
| DPH5 | 1 |
| DPM1 | 1 |
| DPP3 | 1 |
| DPP4 | 1 |
| DPP7 | 1 |
| DPP9 | 1 |
| DPPA2 | 1 |
| DPPA3P2 | 1 |
| DPT | 1 |
| DPY19L2P1 | 1 |
| DPY30 | 1 |
| DPYD | 1 |
| DPYSL2 | 1 |
| DPYSL3 | 1 |
| DR1 | 1 |
| DRAM1 | 1 |
| DRAP1 | 1 |
| DRC1 | 1 |
| DRG1 | 1 |
| DSC1 | 1 |
| DSC2 | 1 |
| DSCAM | 1 |
| DSCC1 | 1 |
| DSCR10 | 1 |
| DSCR3 | 1 |
| DSCR4 | 1 |
| DSE | 1 |
| DSEL | 1 |
| DSG3 | 1 |
| DSG4 | 1 |
| DSTN | 1 |
| DSTNP2 | 1 |
| DTD1 | 1 |
| DTL | 1 |
| DTX3 | 1 |
| DTX4 | 1 |
| DUS1L | 1 |
| DUS2 | 1 |
| DUS4L | 1 |
| DUSP10 | 1 |
| DUSP11 | 1 |
| DUSP14 | 1 |
| DUSP16 | 1 |
| DUSP18 | 1 |
| DUSP3 | 1 |
| DUSP4 | 1 |
| DUSP6 | 1 |
| DUT | 1 |
| DVL2 | 1 |
| DYNAP | 1 |
| DYNC1I2 | 1 |
| DYNC1LI1 | 1 |
| DYNC1LI2 | 1 |
| DYNLL1 | 1 |
| DYNLL2 | 1 |
| DYNLRB1 | 1 |
| DYNLT1 | 1 |
| DYRK3 | 1 |
| DYRK4 | 1 |
| DYSF | 1 |
| E4F1 | 1 |
| EAF2 | 1 |
| EAPP | 1 |
| EBF2 | 1 |
| EBF3 | 1 |
| EBLN2 | 1 |
| EBP | 1 |
| EBPL | 1 |
| ECE1 | 1 |
| ECHDC1 | 1 |
| ECHDC2 | 1 |
| ECHS1 | 1 |
| ECI1 | 1 |
| ECI2 | 1 |
| ECM1 | 1 |
| ECT2 | 1 |
| EDA | 1 |
| EDA2R | 1 |
| EDC4 | 1 |
| EDEM2 | 1 |
| EDF1 | 1 |
| EDIL3 | 1 |
| EDN1 | 1 |
| EDNRA | 1 |
| EDNRB | 1 |
| EEA1 | 1 |
| EEF1A1 | 1 |
| EEF1D | 1 |
| EEF2 | 1 |
| EEPD1 | 1 |
| EFCAB10 | 1 |
| EFCAB13 | 1 |
| EFEMP1 | 1 |
| EFEMP2 | 1 |
| EFHD1 | 1 |
| EFHD2 | 1 |
| EFNA1 | 1 |
| EFNA2 | 1 |
| EFNA4 | 1 |
| EFNB1 | 1 |
| EFTUD2 | 1 |
| EGFR | 1 |
| EGLN3 | 1 |
| EGR2 | 1 |
| EHD1 | 1 |
| EHD2 | 1 |
| EHD4 | 1 |
| EHMT1.IT1 | 1 |
| EI24 | 1 |
| EID1 | 1 |
| EIF1 | 1 |
| EIF1AD | 1 |
| EIF1AY | 1 |
| EIF2AK4 | 1 |
| EIF2B1 | 1 |
| EIF2B3 | 1 |
| EIF2B4 | 1 |
| EIF2D | 1 |
| EIF3A | 1 |
| EIF3C | 1 |
| EIF3D | 1 |
| EIF3E | 1 |
| EIF3G | 1 |
| EIF3H | 1 |
| EIF3I | 1 |
| EIF3J.AS1 | 1 |
| EIF3J | 1 |
| EIF3K | 1 |
| EIF3L | 1 |
| EIF3M | 1 |
| EIF4A3 | 1 |
| EIF4E1B | 1 |
| EIF4E2 | 1 |
| EIF4E3 | 1 |
| EIF4G1 | 1 |
| EIF4G2 | 1 |
| EIF5 | 1 |
| EIF6 | 1 |
| ELF2 | 1 |
| ELF5 | 1 |
| ELFN1 | 1 |
| ELK4 | 1 |
| ELMO1 | 1 |
| ELMO2 | 1 |
| ELN | 1 |
| ELOF1 | 1 |
| ELOVL3 | 1 |
| ELOVL6 | 1 |
| ELOVL7 | 1 |
| ELP3 | 1 |
| ELP5 | 1 |
| ELSPBP1 | 1 |
| ELTD1 | 1 |
| EMC10 | 1 |
| EMC3.AS1 | 1 |
| EMC3 | 1 |
| EMC4 | 1 |
| EMC6 | 1 |
| EMC7 | 1 |
| EMC9 | 1 |
| EMCN | 1 |
| EMD | 1 |
| EMILIN2 | 1 |
| EML2 | 1 |
| EML4 | 1 |
| EMP1 | 1 |
| EMP3 | 1 |
| EMR2 | 1 |
| EMR4P | 1 |
| EMX2 | 1 |
| ENAH | 1 |
| ENDOD1 | 1 |
| ENDOV | 1 |
| ENG | 1 |
| ENHO | 1 |
| ENO1 | 1 |
| ENOX1 | 1 |
| ENPEP | 1 |
| ENPP2 | 1 |
| ENPP7 | 1 |
| ENTHD2 | 1 |
| ENTPD1 | 1 |
| ENTPD3 | 1 |
| ENTPD6 | 1 |
| ENY2 | 1 |
| EP300 | 1 |
| EPAS1 | 1 |
| EPB41 | 1 |
| EPB41L1 | 1 |
| EPB41L3 | 1 |
| EPB41L4B | 1 |
| EPB41L5 | 1 |
| EPG5 | 1 |
| EPGN | 1 |
| EPHB1 | 1 |
| EPOR | 1 |
| EPRS | 1 |
| EPS8 | 1 |
| EPS8L2 | 1 |
| EPYC | 1 |
| ERAL1 | 1 |
| ERBB2 | 1 |
| ERBB2IP | 1 |
| ERC2 | 1 |
| ERCC1 | 1 |
| ERCC2 | 1 |
| ERCC4 | 1 |
| ERCC6L | 1 |
| EREG | 1 |
| ERG | 1 |
| ERGIC1 | 1 |
| ERGIC2 | 1 |
| ERGIC3 | 1 |
| ERI1 | 1 |
| ERICH3 | 1 |
| ERLEC1 | 1 |
| ERLIN1 | 1 |
| ERLIN2 | 1 |
| ERMAP | 1 |
| ERN1 | 1 |
| ERN2 | 1 |
| ERO1L | 1 |
| ERO1LB | 1 |
| ERP27 | 1 |
| ERP29 | 1 |
| ERP44 | 1 |
| ERVFRD.1 | 1 |
| ERVFRD.2 | 1 |
| ESAM | 1 |
| ESCO2 | 1 |
| ESD | 1 |
| ESPN | 1 |
| ESR1 | 1 |
| ESRP1 | 1 |
| ESRRA | 1 |
| ESX1 | 1 |
| ESYT1 | 1 |
| ETAA1 | 1 |
| ETF1 | 1 |
| ETFA | 1 |
| ETFB | 1 |
| ETFDH | 1 |
| ETHE1 | 1 |
| ETV1 | 1 |
| ETV3 | 1 |
| ETV5 | 1 |
| EVA1A | 1 |
| EVA1C | 1 |
| EVC2 | 1 |
| EVL | 1 |
| EXD2 | 1 |
| EXO1 | 1 |
| EXOC3L2 | 1 |
| EXOC6B | 1 |
| EXOC7 | 1 |
| EXOC8 | 1 |
| EXOSC1 | 1 |
| EXOSC2 | 1 |
| EXOSC3 | 1 |
| EXOSC4 | 1 |
| EXOSC8 | 1 |
| EXPH5 | 1 |
| EXT1 | 1 |
| EXT2 | 1 |
| EXTL3 | 1 |
| EYA1 | 1 |
| EZR | 1 |
| F13A1 | 1 |
| F2R | 1 |
| F2RL2 | 1 |
| F5 | 1 |
| F9 | 1 |
| FAAH | 1 |
| FAAH2 | 1 |
| FABP3 | 1 |
| FADD | 1 |
| FADS3 | 1 |
| FAHD1 | 1 |
| FAIM | 1 |
| FAM101B | 1 |
| FAM102B | 1 |
| FAM104A | 1 |
| FAM105A | 1 |
| FAM106A | 1 |
| FAM109A | 1 |
| FAM109B | 1 |
| FAM114A1 | 1 |
| FAM117A | 1 |
| FAM117B | 1 |
| FAM118B | 1 |
| FAM120A | 1 |
| FAM120B | 1 |
| FAM122C | 1 |
| FAM124B | 1 |
| FAM127A | 1 |
| FAM127B | 1 |
| FAM127C | 1 |
| FAM129A | 1 |
| FAM129B | 1 |
| FAM134A | 1 |
| FAM135A | 1 |
| FAM136A | 1 |
| FAM13A | 1 |
| FAM13C | 1 |
| FAM149A | 1 |
| FAM149B1 | 1 |
| FAM150B | 1 |
| FAM154B | 1 |
| FAM156B | 1 |
| FAM159A | 1 |
| FAM160B1 | 1 |
| FAM170B | 1 |
| FAM171A1 | 1 |
| FAM171A2 | 1 |
| FAM171B | 1 |
| FAM172A | 1 |
| FAM173A | 1 |
| FAM174B | 1 |
| FAM175A | 1 |
| FAM175B | 1 |
| FAM177B | 1 |
| FAM180A | 1 |
| FAM181A | 1 |
| FAM186B | 1 |
| FAM189A2 | 1 |
| FAM189B | 1 |
| FAM195B | 1 |
| FAM198A | 1 |
| FAM200B | 1 |
| FAM205A | 1 |
| FAM205B | 1 |
| FAM20B | 1 |
| FAM213A | 1 |
| FAM213B | 1 |
| FAM214B | 1 |
| FAM217A | 1 |
| FAM217B | 1 |
| FAM219A | 1 |
| FAM221A | 1 |
| FAM222A.AS1 | 1 |
| FAM227A | 1 |
| FAM229B | 1 |
| FAM26E | 1 |
| FAM26F | 1 |
| FAM32A | 1 |
| FAM46A | 1 |
| FAM46D | 1 |
| FAM49A | 1 |
| FAM49B | 1 |
| FAM50A | 1 |
| FAM50B | 1 |
| FAM53C | 1 |
| FAM57A | 1 |
| FAM60A | 1 |
| FAM64A | 1 |
| FAM65A | 1 |
| FAM65C | 1 |
| FAM69C | 1 |
| FAM71F2 | 1 |
| FAM73B | 1 |
| FAM78B | 1 |
| FAM83B | 1 |
| FAM83D | 1 |
| FAM86A | 1 |
| FAM86C1 | 1 |
| FAM89A | 1 |
| FAM96A | 1 |
| FAM96B | 1 |
| FAM98A | 1 |
| FAM98B | 1 |
| FAM98C | 1 |
| FAM9A | 1 |
| FAM9B | 1 |
| FAN1 | 1 |
| FANCD2OS | 1 |
| FANCF | 1 |
| FANCG | 1 |
| FANCL | 1 |
| FANK1 | 1 |
| FAR2 | 1 |
| FAR2P1 | 1 |
| FARP1 | 1 |
| FARSB | 1 |
| FAS | 1 |
| FASTK | 1 |
| FASTKD2 | 1 |
| FASTKD3 | 1 |
| FAT3 | 1 |
| FBL | 1 |
| FBLN5 | 1 |
| FBN1 | 1 |
| FBRS | 1 |
| FBXL12 | 1 |
| FBXL19.AS1 | 1 |
| FBXL2 | 1 |
| FBXL21 | 1 |
| FBXL3 | 1 |
| FBXL5 | 1 |
| FBXO15 | 1 |
| FBXO16 | 1 |
| FBXO18 | 1 |
| FBXO21 | 1 |
| FBXO27 | 1 |
| FBXO3 | 1 |
| FBXO30 | 1 |
| FBXO39 | 1 |
| FBXO42 | 1 |
| FBXO5 | 1 |
| FBXO7 | 1 |
| FBXO8 | 1 |
| FBXW4 | 1 |
| FBXW4P1 | 1 |
| FBXW5 | 1 |
| FCAR | 1 |
| FCER1A | 1 |
| FCER1G | 1 |
| FCF1 | 1 |
| FCGBP | 1 |
| FCGR1A | 1 |
| FCGR2A | 1 |
| FCGR2B | 1 |
| FCGR3A | 1 |
| FCRLA | 1 |
| FDFT1 | 1 |
| FDX1L | 1 |
| FDXR | 1 |
| FEM1C | 1 |
| FER | 1 |
| FER1L6.AS1 | 1 |
| FERMT2 | 1 |
| FERMT3 | 1 |
| FEZ1 | 1 |
| FEZ2 | 1 |
| FGA | 1 |
| FGD1 | 1 |
| FGD2 | 1 |
| FGD4 | 1 |
| FGD5 | 1 |
| FGD6 | 1 |
| FGF1 | 1 |
| FGF13 | 1 |
| FGF2 | 1 |
| FGF20 | 1 |
| FGF21 | 1 |
| FGF5 | 1 |
| FGF7 | 1 |
| FGF9 | 1 |
| FGFR3 | 1 |
| FGFRL1 | 1 |
| FGL1 | 1 |
| FGR | 1 |
| FH | 1 |
| FHAD1 | 1 |
| FHIT | 1 |
| FHL1 | 1 |
| FHL2 | 1 |
| FHL5 | 1 |
| FHOD3 | 1 |
| FIBP | 1 |
| FIG4 | 1 |
| FIGF | 1 |
| FIGLA | 1 |
| FIGNL2 | 1 |
| FILIP1 | 1 |
| FILIP1L | 1 |
| FIP1L1 | 1 |
| FIS1 | 1 |
| FITM2 | 1 |
| FJX1 | 1 |
| FKBP10 | 1 |
| FKBP14 | 1 |
| FKBP2 | 1 |
| FKBP3 | 1 |
| FKBP4 | 1 |
| FKBP6 | 1 |
| FKBP8 | 1 |
| FKBPL | 1 |
| FLAD1 | 1 |
| FLJ13224 | 1 |
| FLJ16734 | 1 |
| FLJ20712 | 1 |
| FLJ26245 | 1 |
| FLJ32955 | 1 |
| FLJ34503 | 1 |
| FLJ37201 | 1 |
| FLJ37786 | 1 |
| FLJ38576 | 1 |
| FLJ40194 | 1 |
| FLJ42393 | 1 |
| FLJ44124 | 1 |
| FLJ45950 | 1 |
| FLJ46836 | 1 |
| FLNA | 1 |
| FLNB | 1 |
| FLNC | 1 |
| FLOT2 | 1 |
| FLT1 | 1 |
| FLT4 | 1 |
| FLVCR2 | 1 |
| FLYWCH1 | 1 |
| FLYWCH2 | 1 |
| FMN1 | 1 |
| FMN2 | 1 |
| FMNL1 | 1 |
| FMNL3 | 1 |
| FMO1 | 1 |
| FMO6P | 1 |
| FMO9P | 1 |
| FN1 | 1 |
| FN3KRP | 1 |
| FNBP1 | 1 |
| FNBP1L | 1 |
| FNBP4 | 1 |
| FNDC4 | 1 |
| FNTA | 1 |
| FOCAD | 1 |
| FOLH1 | 1 |
| FOLR2 | 1 |
| FOPNL | 1 |
| FOS | 1 |
| FOSL1 | 1 |
| FOXD1 | 1 |
| FOXE3 | 1 |
| FOXF2 | 1 |
| FOXJ2 | 1 |
| FOXJ3 | 1 |
| FOXL1 | 1 |
| FOXM1 | 1 |
| FOXN3.AS2 | 1 |
| FOXN4 | 1 |
| FOXO1 | 1 |
| FOXP4 | 1 |
| FPGS | 1 |
| FPGT.TNNI3K | 1 |
| FPR2 | 1 |
| FPR3 | 1 |
| FRAS1 | 1 |
| FRK | 1 |
| FRMD3 | 1 |
| FRMD5 | 1 |
| FRMD8 | 1 |
| FRMPD1 | 1 |
| FRMPD3 | 1 |
| FRMPD4 | 1 |
| FSCN1 | 1 |
| FSIP1 | 1 |
| FST | 1 |
| FSTL1 | 1 |
| FSTL3 | 1 |
| FSTL5 | 1 |
| FTH1P3 | 1 |
| FTO | 1 |
| FTSJ1 | 1 |
| FTSJ2 | 1 |
| FTSJ3 | 1 |
| FUBP3 | 1 |
| FUCA2 | 1 |
| FUS | 1 |
| FUT11 | 1 |
| FUT2 | 1 |
| FUZ | 1 |
| FXR2 | 1 |
| FXYD1 | 1 |
| FXYD6 | 1 |
| FYB | 1 |
| FYCO1 | 1 |
| FYN | 1 |
| FYTTD1 | 1 |
| FZD1 | 1 |
| FZD2 | 1 |
| FZD3 | 1 |
| FZD6 | 1 |
| FZD7 | 1 |
| FZR1 | 1 |
| G3BP1 | 1 |
| G3BP2 | 1 |
| G6PC | 1 |
| G6PC3 | 1 |
| G6PD | 1 |
| GAB2 | 1 |
| GAB3 | 1 |
| GABARAP | 1 |
| GABARAPL2 | 1 |
| GABBR1 | 1 |
| GABPB1 | 1 |
| GABRA3 | 1 |
| GABRA4 | 1 |
| GABRA6 | 1 |
| GABRB1 | 1 |
| GABRB2 | 1 |
| GABRB3 | 1 |
| GABRD | 1 |
| GABRG3 | 1 |
| GABRR2 | 1 |
| GAD2 | 1 |
| GADD45A | 1 |
| GADD45GIP1 | 1 |
| GADL1 | 1 |
| GAFA2 | 1 |
| GAFA3 | 1 |
| GAL | 1 |
| GALE | 1 |
| GALNT1 | 1 |
| GALNT10 | 1 |
| GALNT14 | 1 |
| GALNT2 | 1 |
| GALNT5 | 1 |
| GALNT6 | 1 |
| GALNT7 | 1 |
| GALNT8 | 1 |
| GALNTL5 | 1 |
| GAN | 1 |
| GANAB | 1 |
| GAPDH | 1 |
| GAPT | 1 |
| GAREML | 1 |
| GARNL3 | 1 |
| GARS | 1 |
| GART | 1 |
| GAS2 | 1 |
| GAS6 | 1 |
| GATA3 | 1 |
| GATA6 | 1 |
| GATC | 1 |
| GATM | 1 |
| GATS | 1 |
| GBA | 1 |
| GBA3 | 1 |
| GBE1 | 1 |
| GBP2 | 1 |
| GBP3 | 1 |
| GBP4 | 1 |
| GBP5 | 1 |
| GBP6 | 1 |
| GCC1 | 1 |
| GCFC2 | 1 |
| GCH1 | 1 |
| GCKR | 1 |
| GCLM | 1 |
| GCN1L1 | 1 |
| GCNT1 | 1 |
| GCNT4 | 1 |
| GCNT7 | 1 |
| GCRG224 | 1 |
| GCSAM | 1 |
| GCSAML | 1 |
| GDE1 | 1 |
| GDF15 | 1 |
| GDF3 | 1 |
| GDF5 | 1 |
| GDF6 | 1 |
| GDI1 | 1 |
| GDI2 | 1 |
| GDPD3 | 1 |
| GDPD4 | 1 |
| GEM | 1 |
| GEMIN4 | 1 |
| GEMIN7 | 1 |
| GFER | 1 |
| GFM1 | 1 |
| GFM2 | 1 |
| GFPT1 | 1 |
| GGACT | 1 |
| GGNBP2 | 1 |
| GGT5 | 1 |
| GGTA1P | 1 |
| GH1 | 1 |
| GH2 | 1 |
| GHITM | 1 |
| GHR | 1 |
| GHRHR | 1 |
| GHRL | 1 |
| GIGYF2 | 1 |
| GIMAP1.GIMAP5 | 1 |
| GIMAP4 | 1 |
| GIMAP7 | 1 |
| GIMAP8 | 1 |
| GIN1 | 1 |
| GINM1 | 1 |
| GINS2 | 1 |
| GINS3 | 1 |
| GINS4 | 1 |
| GIPC1 | 1 |
| GIPC2 | 1 |
| GIT2 | 1 |
| GJA4 | 1 |
| GJA5 | 1 |
| GJA9 | 1 |
| GJB7 | 1 |
| GJC1 | 1 |
| GK2 | 1 |
| GLB1L | 1 |
| GLCCI1 | 1 |
| GLCE | 1 |
| GLDC | 1 |
| GLE1 | 1 |
| GLI2 | 1 |
| GLI3 | 1 |
| GLIPR1 | 1 |
| GLIPR1L1 | 1 |
| GLIPR2 | 1 |
| GLIS3.AS1 | 1 |
| GLOD4 | 1 |
| GLRA3 | 1 |
| GLRB | 1 |
| GLRX | 1 |
| GLRX3 | 1 |
| GLRX5 | 1 |
| GLS | 1 |
| GLT8D1 | 1 |
| GLT8D2 | 1 |
| GLTP | 1 |
| GLTPD2 | 1 |
| GLTSCR1 | 1 |
| GLUD1 | 1 |
| GLYAT | 1 |
| GLYR1 | 1 |
| GM140 | 1 |
| GM2A | 1 |
| GMDS | 1 |
| GMFB | 1 |
| GMFG | 1 |
| GMNN | 1 |
| GMPPA | 1 |
| GMPPB | 1 |
| GMPR | 1 |
| GMPS | 1 |
| GNA11 | 1 |
| GNA15 | 1 |
| GNAI2 | 1 |
| GNAI3 | 1 |
| GNAQ | 1 |
| GNAT1 | 1 |
| GNB1 | 1 |
| GNB2 | 1 |
| GNB2L1 | 1 |
| GNB5 | 1 |
| GNG10 | 1 |
| GNG11 | 1 |
| GNG12 | 1 |
| GNG13 | 1 |
| GNG2 | 1 |
| GNG3 | 1 |
| GNG4 | 1 |
| GNGT1 | 1 |
| GNGT2 | 1 |
| GNLY | 1 |
| GNN | 1 |
| GNPAT | 1 |
| GNPDA2 | 1 |
| GNPNAT1 | 1 |
| GNPTG | 1 |
| GNRH1 | 1 |
| GNRHR | 1 |
| GNS | 1 |
| GOLGA2 | 1 |
| GOLGA3 | 1 |
| GOLGA5 | 1 |
| GOLGA6L2 | 1 |
| GOLGA7B | 1 |
| GOLGA8M | 1 |
| GOLIM4 | 1 |
| GOLPH3L | 1 |
| GOLT1B | 1 |
| GON4L | 1 |
| GOPC | 1 |
| GORASP1 | 1 |
| GORASP2 | 1 |
| GOSR1 | 1 |
| GOT1 | 1 |
| GOT2 | 1 |
| GP6 | 1 |
| GPA33 | 1 |
| GPAA1 | 1 |
| GPALPP1 | 1 |
| GPAT2 | 1 |
| GPATCH3 | 1 |
| GPATCH4 | 1 |
| GPBP1 | 1 |
| GPC4 | 1 |
| GPC6 | 1 |
| GPCPD1 | 1 |
| GPD1L | 1 |
| GPD2 | 1 |
| GPER1 | 1 |
| GPI | 1 |
| GPM6B | 1 |
| GPN1 | 1 |
| GPN2 | 1 |
| GPN3 | 1 |
| GPNMB | 1 |
| GPR1 | 1 |
| GPR107 | 1 |
| GPR111 | 1 |
| GPR114 | 1 |
| GPR116 | 1 |
| GPR133 | 1 |
| GPR135 | 1 |
| GPR141 | 1 |
| GPR151 | 1 |
| GPR153 | 1 |
| GPR156 | 1 |
| GPR17 | 1 |
| GPR176 | 1 |
| GPR18 | 1 |
| GPR183 | 1 |
| GPR21 | 1 |
| GPR34 | 1 |
| GPR37 | 1 |
| GPR39 | 1 |
| GPR4 | 1 |
| GPR50 | 1 |
| GPR52 | 1 |
| GPR55 | 1 |
| GPR65 | 1 |
| GPR68 | 1 |
| GPR75 | 1 |
| GPR85 | 1 |
| GPR89A | 1 |
| GPR98 | 1 |
| GPRC5B | 1 |
| GPRC5C | 1 |
| GPRC5D | 1 |
| GPRIN1 | 1 |
| GPRIN3 | 1 |
| GPS1 | 1 |
| GPSM1 | 1 |
| GPT2 | 1 |
| GPX1 | 1 |
| GPX4 | 1 |
| GPX5 | 1 |
| GPX8 | 1 |
| GRAMD1B | 1 |
| GRAMD2 | 1 |
| GRAMD3 | 1 |
| GRAP | 1 |
| GRAP2 | 1 |
| GRAPL | 1 |
| GRASP | 1 |
| GRB14 | 1 |
| GRB2 | 1 |
| GREB1 | 1 |
| GREM1 | 1 |
| GREM2 | 1 |
| GRHL3 | 1 |
| GRHPR | 1 |
| GRIK1.AS1 | 1 |
| GRIK2 | 1 |
| GRK5 | 1 |
| GRM3 | 1 |
| GRM4 | 1 |
| GRM8 | 1 |
| GRPEL1 | 1 |
| GRPEL2 | 1 |
| GRSF1 | 1 |
| GRWD1 | 1 |
| GS52 | 1 |
| GSC | 1 |
| GSDMC | 1 |
| GSG2 | 1 |
| GSKIP | 1 |
| GSN.AS1 | 1 |
| GSPT1 | 1 |
| GSS | 1 |
| GSTA1 | 1 |
| GSTA2 | 1 |
| GSTCD | 1 |
| GSTM2P1 | 1 |
| GSTM3 | 1 |
| GSTM5 | 1 |
| GSTO1 | 1 |
| GSTO2 | 1 |
| GSTP1 | 1 |
| GSTT1 | 1 |
| GSTT2 | 1 |
| GSTZ1 | 1 |
| GTDC1 | 1 |
| GTF2A1L | 1 |
| GTF2A2 | 1 |
| GTF2B | 1 |
| GTF2E2 | 1 |
| GTF2F1 | 1 |
| GTF2F2 | 1 |
| GTF2H1 | 1 |
| GTF2H3 | 1 |
| GTF2H5 | 1 |
| GTF3C4 | 1 |
| GTF3C6 | 1 |
| GTPBP6 | 1 |
| GTSE1 | 1 |
| GTSF1 | 1 |
| GUCA1A | 1 |
| GUCY1A2 | 1 |
| GUCY1A3 | 1 |
| GUCY1B3 | 1 |
| GUCY2C | 1 |
| GUSBP11 | 1 |
| GUSBP5 | 1 |
| GVINP1 | 1 |
| GXYLT2 | 1 |
| GYG1 | 1 |
| GYG2 | 1 |
| GYPA | 1 |
| GYS1 | 1 |
| GZMA | 1 |
| GZMK | 1 |
| H19 | 1 |
| H2AFB1 | 1 |
| H2AFX | 1 |
| H2AFY | 1 |
| H2AFZ | 1 |
| H2BFM | 1 |
| H3F3C | 1 |
| HABP4 | 1 |
| HADHA | 1 |
| HADHB | 1 |
| HAGH | 1 |
| HAL | 1 |
| HAMP | 1 |
| HAO2 | 1 |
| HARBI1 | 1 |
| HAS2 | 1 |
| HAS3 | 1 |
| HAT1 | 1 |
| HAUS2 | 1 |
| HAUS7 | 1 |
| HAUS8 | 1 |
| HAVCR2 | 1 |
| HAX1 | 1 |
| HBA2 | 1 |
| HBG2 | 1 |
| HBS1L | 1 |
| HCCS | 1 |
| HCFC1 | 1 |
| HCG4 | 1 |
| HCG8 | 1 |
| HCK | 1 |
| HCLS1 | 1 |
| HCP5 | 1 |
| HDAC1 | 1 |
| HDAC2 | 1 |
| HDAC8 | 1 |
| HDC | 1 |
| HDDC2 | 1 |
| HDDC3 | 1 |
| HDGF | 1 |
| HDGFRP2 | 1 |
| HDX | 1 |
| HEATR2 | 1 |
| HEATR5B | 1 |
| HEBP1 | 1 |
| HEBP2 | 1 |
| HECTD2 | 1 |
| HECTD3 | 1 |
| HECW1.IT1 | 1 |
| HEG1 | 1 |
| HELT | 1 |
| HENMT1 | 1 |
| HEPACAM | 1 |
| HEPH | 1 |
| HERC4 | 1 |
| HERC5 | 1 |
| HERPUD1 | 1 |
| HES1 | 1 |
| HESX1 | 1 |
| HEXIM1 | 1 |
| HEXIM2 | 1 |
| HEY1 | 1 |
| HEY2 | 1 |
| HEYL | 1 |
| HFE | 1 |
| HFE2 | 1 |
| HFM1 | 1 |
| HGF | 1 |
| HGS | 1 |
| HHATL | 1 |
| HHEX | 1 |
| HHLA1 | 1 |
| HHLA2 | 1 |
| HIATL1 | 1 |
| HIBADH | 1 |
| HIC2 | 1 |
| HIF1A | 1 |
| HIF1AN | 1 |
| HIF3A | 1 |
| HIGD1A | 1 |
| HIGD2A | 1 |
| HINT1 | 1 |
| HINT3 | 1 |
| HIST1H1A | 1 |
| HIST1H1B | 1 |
| HIST1H1D | 1 |
| HIST1H1T | 1 |
| HIST1H2AB | 1 |
| HIST1H2AE | 1 |
| HIST1H2AI | 1 |
| HIST1H2AJ | 1 |
| HIST1H2BB | 1 |
| HIST1H2BC | 1 |
| HIST1H2BD | 1 |
| HIST1H2BE | 1 |
| HIST1H2BH | 1 |
| HIST1H2BL | 1 |
| HIST1H2BM | 1 |
| HIST1H2BO | 1 |
| HIST1H3A | 1 |
| HIST1H3B | 1 |
| HIST1H3E | 1 |
| HIST1H3F | 1 |
| HIST1H3I | 1 |
| HIST1H3J | 1 |
| HIST1H4C | 1 |
| HIST1H4G | 1 |
| HIST1H4H | 1 |
| HIST1H4I | 1 |
| HIST1H4K | 1 |
| HIST1H4L | 1 |
| HIST2H2AA4 | 1 |
| HIST2H2AC | 1 |
| HIST2H2BE | 1 |
| HIVEP1 | 1 |
| HIVEP3 | 1 |
| HJURP | 1 |
| HK1 | 1 |
| HK3 | 1 |
| HKR1 | 1 |
| HLA.DMB | 1 |
| HLA.DOA | 1 |
| HLA.DOB | 1 |
| HLA.DPA1 | 1 |
| HLA.DPB2 | 1 |
| HLA.DRA | 1 |
| HLA.DRB5 | 1 |
| HLX | 1 |
| HM13 | 1 |
| HMBOX1 | 1 |
| HMBS | 1 |
| HMCES | 1 |
| HMG20B | 1 |
| HMGA1 | 1 |
| HMGA2 | 1 |
| HMGB1 | 1 |
| HMGCL | 1 |
| HMGCLL1 | 1 |
| HMGN2 | 1 |
| HMGN2P46 | 1 |
| HMGN3 | 1 |
| HMGN5 | 1 |
| HMGXB4 | 1 |
| HMOX1 | 1 |
| HMOX2 | 1 |
| HMP19 | 1 |
| HN1 | 1 |
| HN1L | 1 |
| HNF1A.AS1 | 1 |
| HNF4G | 1 |
| HNRNPA0 | 1 |
| HNRNPA3P1 | 1 |
| HNRNPAB | 1 |
| HNRNPD | 1 |
| HNRNPF | 1 |
| HNRNPH1 | 1 |
| HNRNPH2 | 1 |
| HNRNPH3 | 1 |
| HNRNPK | 1 |
| HNRNPL | 1 |
| HNRNPLL | 1 |
| HNRNPM | 1 |
| HNRNPU.AS1 | 1 |
| HNRNPUL1 | 1 |
| HOMER3 | 1 |
| HOOK3 | 1 |
| HOXA.AS2 | 1 |
| HOXA2 | 1 |
| HOXA3 | 1 |
| HOXA4 | 1 |
| HOXA6 | 1 |
| HOXA7 | 1 |
| HOXB2 | 1 |
| HOXB3 | 1 |
| HOXB4 | 1 |
| HOXB5 | 1 |
| HOXB6 | 1 |
| HOXB7 | 1 |
| HOXC10 | 1 |
| HOXC8 | 1 |
| HOXD11 | 1 |
| HOXD13 | 1 |
| HOXD3 | 1 |
| HOXD4 | 1 |
| HOXD9 | 1 |
| HP | 1 |
| HPCAL1 | 1 |
| HPD | 1 |
| HPGDS | 1 |
| HPS3 | 1 |
| HPS5 | 1 |
| HPS6 | 1 |
| HPX | 1 |
| HRAS | 1 |
| HRC | 1 |
| HRCT1 | 1 |
| HRG | 1 |
| HRH2 | 1 |
| HRSP12 | 1 |
| HSBP1 | 1 |
| HSCB | 1 |
| HSD11B2 | 1 |
| HSD17B11 | 1 |
| HSD17B12 | 1 |
| HSD17B13 | 1 |
| HSD17B2 | 1 |
| HSD17B3 | 1 |
| HSD17B4 | 1 |
| HSD3B7 | 1 |
| HSDL2 | 1 |
| HSFY1 | 1 |
| HSP90AA1 | 1 |
| HSP90AA2 | 1 |
| HSP90AA4P | 1 |
| HSP90AB1 | 1 |
| HSP90AB2P | 1 |
| HSP90AB4P | 1 |
| HSP90AB6P | 1 |
| HSP90B2P | 1 |
| HSPA12B | 1 |
| HSPA13 | 1 |
| HSPA14 | 1 |
| HSPA4 | 1 |
| HSPA5 | 1 |
| HSPA8 | 1 |
| HSPA9 | 1 |
| HSPB1 | 1 |
| HSPB11 | 1 |
| HSPB7 | 1 |
| HSPBP1 | 1 |
| HSPD1 | 1 |
| HSPE1 | 1 |
| HSPH1 | 1 |
| HTR1E | 1 |
| HTR1F | 1 |
| HTR2B | 1 |
| HTR3B | 1 |
| HTR7P1 | 1 |
| HTRA1 | 1 |
| HTRA2 | 1 |
| HUS1B | 1 |
| HYAL2 | 1 |
| HYKK | 1 |
| HYLS1 | 1 |
| IAH1 | 1 |
| IARS | 1 |
| IBSP | 1 |
| IBTK | 1 |
| ICA1 | 1 |
| ICAM2 | 1 |
| ICE2 | 1 |
| ICMT | 1 |
| ICOS | 1 |
| ICT1 | 1 |
| ID1 | 1 |
| ID2 | 1 |
| ID4 | 1 |
| IDH1 | 1 |
| IDH3A | 1 |
| IDH3B | 1 |
| IDH3G | 1 |
| IDI1 | 1 |
| IDO1 | 1 |
| IDS | 1 |
| IER3IP1 | 1 |
| IER5 | 1 |
| IER5L | 1 |
| IFI27 | 1 |
| IFI27L1 | 1 |
| IFI27L2 | 1 |
| IFI44 | 1 |
| IFI44L | 1 |
| IFIH1 | 1 |
| IFIT1 | 1 |
| IFITM1 | 1 |
| IFITM2 | 1 |
| IFITM5 | 1 |
| IFNA10 | 1 |
| IFNA13 | 1 |
| IFNA16 | 1 |
| IFNA4 | 1 |
| IFNA5 | 1 |
| IFNA6 | 1 |
| IFNAR1 | 1 |
| IFNAR2 | 1 |
| IFNL3 | 1 |
| IFRD2 | 1 |
| IFT140 | 1 |
| IFT22 | 1 |
| IFT46 | 1 |
| IFT57 | 1 |
| IGDCC4 | 1 |
| IGF1 | 1 |
| IGF2BP3 | 1 |
| IGFBP1 | 1 |
| IGFBP3 | 1 |
| IGFBP4 | 1 |
| IGFBP5 | 1 |
| IGFBP6 | 1 |
| IGFBP7 | 1 |
| IGFL3 | 1 |
| IGFL4 | 1 |
| IGHV7.81 | 1 |
| IGSF10 | 1 |
| IGSF11 | 1 |
| IGSF3 | 1 |
| IGSF6 | 1 |
| IK | 1 |
| IKBIP | 1 |
| IKBKE | 1 |
| IKZF1 | 1 |
| IKZF3 | 1 |
| IKZF4 | 1 |
| IL10RA | 1 |
| IL11RA | 1 |
| IL12A | 1 |
| IL13RA1 | 1 |
| IL13RA2 | 1 |
| IL17RE | 1 |
| IL18 | 1 |
| IL18BP | 1 |
| IL18R1 | 1 |
| IL1B | 1 |
| IL1R2 | 1 |
| IL1RAPL2 | 1 |
| IL1RL2 | 1 |
| IL1RN | 1 |
| IL20RA | 1 |
| IL23R | 1 |
| IL24 | 1 |
| IL25 | 1 |
| IL26 | 1 |
| IL2RA | 1 |
| IL2RG | 1 |
| IL3 | 1 |
| IL32 | 1 |
| IL36G | 1 |
| IL3RA | 1 |
| IL5RA | 1 |
| IL6ST | 1 |
| IL9 | 1 |
| ILDR1 | 1 |
| ILDR2 | 1 |
| ILF2 | 1 |
| ILF3 | 1 |
| ILK | 1 |
| ILVBL | 1 |
| IMMP1L | 1 |
| IMMT | 1 |
| IMP3 | 1 |
| IMP4 | 1 |
| IMPAD1 | 1 |
| IMPDH2 | 1 |
| IMPG2 | 1 |
| INCENP | 1 |
| INE1 | 1 |
| INHBB | 1 |
| INIP | 1 |
| INMT | 1 |
| INPP4A | 1 |
| INPP5D | 1 |
| INPP5E | 1 |
| INPPL1 | 1 |
| INSC | 1 |
| INSIG1 | 1 |
| INSL4 | 1 |
| INSL6 | 1 |
| INSR | 1 |
| INTS12 | 1 |
| INTS9 | 1 |
| INTU | 1 |
| INVS | 1 |
| IPCEF1 | 1 |
| IPO11 | 1 |
| IPO5 | 1 |
| IPO7 | 1 |
| IPO9 | 1 |
| IPP | 1 |
| IQCF1 | 1 |
| IQCK | 1 |
| IQGAP1 | 1 |
| IQGAP2 | 1 |
| IQSEC2 | 1 |
| IRF2BP1 | 1 |
| IRF6 | 1 |
| IRF8 | 1 |
| IRS4 | 1 |
| IRX1 | 1 |
| IRX3 | 1 |
| ISLR | 1 |
| ISLR2 | 1 |
| ISOC2 | 1 |
| ISX | 1 |
| ISY1 | 1 |
| ITFG1 | 1 |
| ITFG3 | 1 |
| ITGA11 | 1 |
| ITGA3 | 1 |
| ITGA5 | 1 |
| ITGA6 | 1 |
| ITGA9 | 1 |
| ITGAD | 1 |
| ITGAL | 1 |
| ITGAM | 1 |
| ITGAX | 1 |
| ITGB1 | 1 |
| ITGB1BP1 | 1 |
| ITGB1BP2 | 1 |
| ITGB5 | 1 |
| ITGB7 | 1 |
| ITGBL1 | 1 |
| ITIH4 | 1 |
| ITK | 1 |
| ITLN2 | 1 |
| ITM2A | 1 |
| ITM2C | 1 |
| ITPKB | 1 |
| ITPKC | 1 |
| ITPR2 | 1 |
| ITPR3 | 1 |
| ITPRIP | 1 |
| ITPRIPL2 | 1 |
| IVD | 1 |
| IYD | 1 |
| IZUMO2 | 1 |
| JADE3 | 1 |
| JAG2 | 1 |
| JAGN1 | 1 |
| JAM2 | 1 |
| JAM3 | 1 |
| JARID2 | 1 |
| JDP2 | 1 |
| JMJD1C | 1 |
| JMJD6 | 1 |
| JMJD7 | 1 |
| JMJD8 | 1 |
| JOSD1 | 1 |
| JOSD2 | 1 |
| JPH1 | 1 |
| JRK | 1 |
| JRKL | 1 |
| JUN | 1 |
| JUP | 1 |
| KALRN | 1 |
| KANK2 | 1 |
| KANSL1 | 1 |
| KANSL1L | 1 |
| KANSL3 | 1 |
| KARS | 1 |
| KAT5 | 1 |
| KAT8 | 1 |
| KATNAL1 | 1 |
| KATNB1 | 1 |
| KBTBD4 | 1 |
| KBTBD8 | 1 |
| KCNA3 | 1 |
| KCNAB1 | 1 |
| KCNAB3 | 1 |
| KCNC4 | 1 |
| KCNE1 | 1 |
| KCNE1L | 1 |
| KCNE2 | 1 |
| KCNE3 | 1 |
| KCNG1 | 1 |
| KCNH7 | 1 |
| KCNIP2 | 1 |
| KCNIP4 | 1 |
| KCNJ11 | 1 |
| KCNJ5 | 1 |
| KCNK1 | 1 |
| KCNK15 | 1 |
| KCNK17 | 1 |
| KCNK2 | 1 |
| KCNK3 | 1 |
| KCNK5 | 1 |
| KCNK6 | 1 |
| KCNK9 | 1 |
| KCNMA1 | 1 |
| KCNMB1 | 1 |
| KCNMB3 | 1 |
| KCNMB4 | 1 |
| KCNN2 | 1 |
| KCNN3 | 1 |
| KCNN4 | 1 |
| KCNQ1 | 1 |
| KCNQ4 | 1 |
| KCNQ5 | 1 |
| KCNV2 | 1 |
| KCTD10 | 1 |
| KCTD11 | 1 |
| KCTD12 | 1 |
| KCTD16 | 1 |
| KCTD18 | 1 |
| KCTD2 | 1 |
| KCTD20 | 1 |
| KCTD3 | 1 |
| KCTD4 | 1 |
| KCTD5 | 1 |
| KCTD9 | 1 |
| KDELC1 | 1 |
| KDELC2 | 1 |
| KDELR1 | 1 |
| KDELR3 | 1 |
| KDF1 | 1 |
| KDM1B | 1 |
| KDM2A | 1 |
| KDM3A | 1 |
| KDM7A | 1 |
| KDR | 1 |
| KEAP1 | 1 |
| KEL | 1 |
| KHDC1L | 1 |
| KHDRBS1 | 1 |
| KHDRBS2 | 1 |
| KHDRBS3 | 1 |
| KHSRP | 1 |
| KIAA0100 | 1 |
| KIAA0101 | 1 |
| KIAA0196 | 1 |
| KIAA0247 | 1 |
| KIAA0895 | 1 |
| KIAA0895L | 1 |
| KIAA0907 | 1 |
| KIAA0922 | 1 |
| KIAA1024 | 1 |
| KIAA1147 | 1 |
| KIAA1191 | 1 |
| KIAA1211L | 1 |
| KIAA1257 | 1 |
| KIAA1279 | 1 |
| KIAA1328 | 1 |
| KIAA1429 | 1 |
| KIAA1462 | 1 |
| KIAA1549L | 1 |
| KIAA1551 | 1 |
| KIAA1958 | 1 |
| KIDINS220 | 1 |
| KIF11 | 1 |
| KIF13A | 1 |
| KIF1C | 1 |
| KIF20A | 1 |
| KIF21A | 1 |
| KIF22 | 1 |
| KIF23 | 1 |
| KIF25 | 1 |
| KIF26A | 1 |
| KIF2B | 1 |
| KIF3B | 1 |
| KIF3C | 1 |
| KIF5B | 1 |
| KIF5C | 1 |
| KIF6 | 1 |
| KIN | 1 |
| KIR2DL5A | 1 |
| KIRREL3 | 1 |
| KITLG | 1 |
| KL | 1 |
| KLC1 | 1 |
| KLC2 | 1 |
| KLF11 | 1 |
| KLF16 | 1 |
| KLF2 | 1 |
| KLF3.AS1 | 1 |
| KLF3 | 1 |
| KLF7 | 1 |
| KLF9 | 1 |
| KLHDC10 | 1 |
| KLHDC8B | 1 |
| KLHL11 | 1 |
| KLHL26 | 1 |
| KLHL3 | 1 |
| KLHL31 | 1 |
| KLHL32 | 1 |
| KLHL36 | 1 |
| KLHL6 | 1 |
| KLHL7 | 1 |
| KLHL8 | 1 |
| KLHL9 | 1 |
| KLK11 | 1 |
| KLK12 | 1 |
| KLK14 | 1 |
| KLK3 | 1 |
| KLK4 | 1 |
| KLRB1 | 1 |
| KLRC3 | 1 |
| KLRC4.KLRK1 | 1 |
| KLRC4 | 1 |
| KLRD1 | 1 |
| KLRG1 | 1 |
| KMO | 1 |
| KMT2A | 1 |
| KMT2B | 1 |
| KMT2C | 1 |
| KMT2D | 1 |
| KNSTRN | 1 |
| KPNA1 | 1 |
| KPNA2 | 1 |
| KPNA3 | 1 |
| KPNA4 | 1 |
| KPNA6 | 1 |
| KPNB1 | 1 |
| KPRP | 1 |
| KPTN | 1 |
| KRIT1 | 1 |
| KRT14 | 1 |
| KRT15 | 1 |
| KRT18 | 1 |
| KRT18P55 | 1 |
| KRT19 | 1 |
| KRT25 | 1 |
| KRT27 | 1 |
| KRT28 | 1 |
| KRT33A | 1 |
| KRT36 | 1 |
| KRT6B | 1 |
| KRT7 | 1 |
| KRT83 | 1 |
| KRT8P41 | 1 |
| KRTAP1.1 | 1 |
| KRTAP1.3 | 1 |
| KRTAP1.4 | 1 |
| KRTAP1.5 | 1 |
| KRTAP10.1 | 1 |
| KRTAP10.12 | 1 |
| KRTAP10.2 | 1 |
| KRTAP12.1 | 1 |
| KRTAP13.3 | 1 |
| KRTAP17.1 | 1 |
| KRTAP19.2 | 1 |
| KRTAP19.3 | 1 |
| KRTAP19.4 | 1 |
| KRTAP19.7 | 1 |
| KRTAP2.1 | 1 |
| KRTAP2.3 | 1 |
| KRTAP2.4 | 1 |
| KRTAP21.2 | 1 |
| KRTAP22.1 | 1 |
| KRTAP5.1 | 1 |
| KRTAP5.3 | 1 |
| KRTAP5.4 | 1 |
| KRTAP8.1 | 1 |
| KRTAP9.3 | 1 |
| KRTCAP3 | 1 |
| KSR1 | 1 |
| KTI12 | 1 |
| KTN1.AS1 | 1 |
| KXD1 | 1 |
| KY | 1 |
| L2HGDH | 1 |
| L3MBTL1 | 1 |
| L3MBTL4 | 1 |
| LACTB | 1 |
| LAIR1 | 1 |
| LAMA2 | 1 |
| LAMB1 | 1 |
| LAMB2 | 1 |
| LAMB4 | 1 |
| LAMC1 | 1 |
| LAMP1 | 1 |
| LAMP2 | 1 |
| LAMP3 | 1 |
| LAMTOR2 | 1 |
| LAMTOR3 | 1 |
| LAMTOR4 | 1 |
| LAMTOR5 | 1 |
| LANCL1 | 1 |
| LANCL2 | 1 |
| LAPTM4A | 1 |
| LAPTM5 | 1 |
| LARP1B | 1 |
| LARP4 | 1 |
| LARP6 | 1 |
| LARS | 1 |
| LASP1 | 1 |
| LAT2 | 1 |
| LAYN | 1 |
| LBH | 1 |
| LBX2 | 1 |
| LCAT | 1 |
| LCE3C | 1 |
| LCE4A | 1 |
| LCE5A | 1 |
| LCMT1 | 1 |
| LCMT2 | 1 |
| LCN10 | 1 |
| LCP1 | 1 |
| LCP2 | 1 |
| LDHA | 1 |
| LDHAL6A | 1 |
| LDHB | 1 |
| LDLR | 1 |
| LDLRAD4 | 1 |
| LDOC1L | 1 |
| LEAP2 | 1 |
| LECT2 | 1 |
| LEF1 | 1 |
| LEFTY2 | 1 |
| LEKR1 | 1 |
| LELP1 | 1 |
| LEMD1 | 1 |
| LENEP | 1 |
| LENG8 | 1 |
| LEO1 | 1 |
| LEPR | 1 |
| LEPREL1 | 1 |
| LEPREL2 | 1 |
| LEPREL4 | 1 |
| LEPROTL1 | 1 |
| LETM2 | 1 |
| LGALS1 | 1 |
| LGALS12 | 1 |
| LGALS14 | 1 |
| LGALS3 | 1 |
| LGALS9C | 1 |
| LGALSL | 1 |
| LGI3 | 1 |
| LGSN | 1 |
| LHFP | 1 |
| LHFPL1 | 1 |
| LHFPL2 | 1 |
| LHPP | 1 |
| LHX2 | 1 |
| LHX4 | 1 |
| LHX9 | 1 |
| LIF | 1 |
| LIFR | 1 |
| LILRA1 | 1 |
| LILRA3 | 1 |
| LILRB1 | 1 |
| LILRB2 | 1 |
| LILRB3 | 1 |
| LILRB4 | 1 |
| LIMA1 | 1 |
| LIMD2 | 1 |
| LIMK1 | 1 |
| LIMS1 | 1 |
| LIMS3L | 1 |
| LIN37 | 1 |
| LIN7C | 1 |
| LINC00086 | 1 |
| LINC00094 | 1 |
| LINC00114 | 1 |
| LINC00158 | 1 |
| LINC00174 | 1 |
| LINC00216 | 1 |
| LINC00272 | 1 |
| LINC00305 | 1 |
| LINC00308 | 1 |
| LINC00341 | 1 |
| LINC00469 | 1 |
| LINC00477 | 1 |
| LINC00483 | 1 |
| LINC00487 | 1 |
| LINC00526 | 1 |
| LINC00612 | 1 |
| LINC00656 | 1 |
| LINC00665 | 1 |
| LINC00687 | 1 |
| LINC00852 | 1 |
| LINC00893 | 1 |
| LINC00921 | 1 |
| LINC00965 | 1 |
| LINC00982 | 1 |
| LINC00994 | 1 |
| LINC00999 | 1 |
| LINC01165 | 1 |
| LINC01260 | 1 |
| LINC01341 | 1 |
| LINC01356 | 1 |
| LINGO4 | 1 |
| LIPF | 1 |
| LIPI | 1 |
| LIX1L | 1 |
| LMAN2 | 1 |
| LMAN2L | 1 |
| LMBR1L | 1 |
| LMBRD1 | 1 |
| LMCD1 | 1 |
| LMF1 | 1 |
| LMF2 | 1 |
| LMNA | 1 |
| LMNB1 | 1 |
| LMNB2 | 1 |
| LMNTD1 | 1 |
| LMO7 | 1 |
| LOC100127886 | 1 |
| LOC100128175 | 1 |
| LOC100128185 | 1 |
| LOC100128310 | 1 |
| LOC100128356 | 1 |
| LOC100128364 | 1 |
| LOC100128653 | 1 |
| LOC100128922 | 1 |
| LOC100129312 | 1 |
| LOC100129345 | 1 |
| LOC100129449 | 1 |
| LOC100129572 | 1 |
| LOC100129785 | 1 |
| LOC100129884 | 1 |
| LOC100129924 | 1 |
| LOC100130331 | 1 |
| LOC100130428 | 1 |
| LOC100130691 | 1 |
| LOC100130815 | 1 |
| LOC100130916 | 1 |
| LOC100130924 | 1 |
| LOC100131131 | 1 |
| LOC100131497 | 1 |
| LOC100131508 | 1 |
| LOC100131541 | 1 |
| LOC100131796 | 1 |
| LOC100131826 | 1 |
| LOC100131943 | 1 |
| LOC100132005 | 1 |
| LOC100132099 | 1 |
| LOC100132686 | 1 |
| LOC100132731 | 1 |
| LOC100133131 | 1 |
| LOC100133315 | 1 |
| LOC100287290 | 1 |
| LOC100287852 | 1 |
| LOC100287869 | 1 |
| LOC100288637 | 1 |
| LOC100288814 | 1 |
| LOC100288869 | 1 |
| LOC100288974 | 1 |
| LOC100506730 | 1 |
| LOC100507646 | 1 |
| LOC100652824 | 1 |
| LOC101928068 | 1 |
| LOC101928885 | 1 |
| LOC101929829 | 1 |
| LOC102288414 | 1 |
| LOC151174 | 1 |
| LOC153684 | 1 |
| LOC220077 | 1 |
| LOC283588 | 1 |
| LOC286359 | 1 |
| LOC339059 | 1 |
| LOC392364 | 1 |
| LOC400590 | 1 |
| LOC400661 | 1 |
| LOC400940 | 1 |
| LOC440311 | 1 |
| LOC440313 | 1 |
| LOC441179 | 1 |
| LOC441242 | 1 |
| LOC441601 | 1 |
| LOC494127 | 1 |
| LOC494141 | 1 |
| LOC494150 | 1 |
| LOC613266 | 1 |
| LOC644936 | 1 |
| LOC645010 | 1 |
| LOC645166 | 1 |
| LOC645261 | 1 |
| LOC646214 | 1 |
| LOC646513 | 1 |
| LOC646813 | 1 |
| LOC646903 | 1 |
| LOC647983 | 1 |
| LOC728175 | 1 |
| LOC728554 | 1 |
| LOC729603 | 1 |
| LOC729800 | 1 |
| LOC93622 | 1 |
| LONP1 | 1 |
| LONRF3 | 1 |
| LOR | 1 |
| LOX | 1 |
| LOXL1 | 1 |
| LOXL2 | 1 |
| LOXL3 | 1 |
| LPAR1 | 1 |
| LPAR4 | 1 |
| LPAR5 | 1 |
| LPAR6 | 1 |
| LPCAT3 | 1 |
| LPCAT4 | 1 |
| LPHN1 | 1 |
| LPL | 1 |
| LPPR2 | 1 |
| LPPR3 | 1 |
| LPPR4 | 1 |
| LPXN | 1 |
| LRCH1 | 1 |
| LRCH4 | 1 |
| LRFN2 | 1 |
| LRGUK | 1 |
| LRIG2 | 1 |
| LRMP | 1 |
| LRP1 | 1 |
| LRP10 | 1 |
| LRP11 | 1 |
| LRP1B | 1 |
| LRP2 | 1 |
| LRP4 | 1 |
| LRRC1 | 1 |
| LRRC17 | 1 |
| LRRC2 | 1 |
| LRRC31 | 1 |
| LRRC32 | 1 |
| LRRC36 | 1 |
| LRRC39 | 1 |
| LRRC3DN | 1 |
| LRRC4 | 1 |
| LRRC41 | 1 |
| LRRC42 | 1 |
| LRRC45 | 1 |
| LRRC46 | 1 |
| LRRC4B | 1 |
| LRRC59 | 1 |
| LRRC63 | 1 |
| LRRC70 | 1 |
| LRRC8A | 1 |
| LRRC8B | 1 |
| LRRC8D | 1 |
| LRRIQ1 | 1 |
| LRRN4CL | 1 |
| LSM1 | 1 |
| LSM2 | 1 |
| LSM3 | 1 |
| LSM4 | 1 |
| LSM7 | 1 |
| LSM8 | 1 |
| LSMEM1 | 1 |
| LST1 | 1 |
| LTB4R | 1 |
| LTB4R2 | 1 |
| LTBP2 | 1 |
| LTBP3 | 1 |
| LTK | 1 |
| LTV1 | 1 |
| LUC7L | 1 |
| LUC7L3 | 1 |
| LURAP1L | 1 |
| LUZP6 | 1 |
| LY6G5B | 1 |
| LY6K | 1 |
| LY86.AS1 | 1 |
| LY86 | 1 |
| LYG1 | 1 |
| LYN | 1 |
| LYNX1 | 1 |
| LYPD1 | 1 |
| LYPD5 | 1 |
| LYPD6 | 1 |
| LYPD6B | 1 |
| LYPLA2 | 1 |
| LYRM1 | 1 |
| LYRM2 | 1 |
| LYSMD1 | 1 |
| LYSMD3 | 1 |
| LYVE1 | 1 |
| LYZ | 1 |
| LYZL1 | 1 |
| LZIC | 1 |
| LZTR1 | 1 |
| LZTS1 | 1 |
| LZTS2 | 1 |
| M6PR | 1 |
| MAATS1 | 1 |
| MAB21L3 | 1 |
| MACC1 | 1 |
| MAD2L1 | 1 |
| MAD2L1BP | 1 |
| MADCAM1 | 1 |
| MAEL | 1 |
| MAF | 1 |
| MAFB | 1 |
| MAFK | 1 |
| MAGEA1 | 1 |
| MAGEA9B | 1 |
| MAGEB16 | 1 |
| MAGEB18 | 1 |
| MAGEB3 | 1 |
| MAGEB6 | 1 |
| MAGEC2 | 1 |
| MAGEC3 | 1 |
| MAGEE1 | 1 |
| MAGEF1 | 1 |
| MALSU1 | 1 |
| MAML1 | 1 |
| MAML3 | 1 |
| MAMLD1 | 1 |
| MAMSTR | 1 |
| MAN1A1 | 1 |
| MAN1B1 | 1 |
| MAN2A1 | 1 |
| MAN2B2 | 1 |
| MANBAL | 1 |
| MANEA | 1 |
| MANSC1 | 1 |
| MAP10 | 1 |
| MAP1A | 1 |
| MAP1B | 1 |
| MAP1LC3BP1 | 1 |
| MAP1S | 1 |
| MAP2K2 | 1 |
| MAP2K6 | 1 |
| MAP3K1 | 1 |
| MAP3K7 | 1 |
| MAP4K4 | 1 |
| MAPK1 | 1 |
| MAPK12 | 1 |
| MAPK13 | 1 |
| MAPK14 | 1 |
| MAPK1IP1L | 1 |
| MAPK3 | 1 |
| MAPK6 | 1 |
| MAPK8IP3 | 1 |
| MAPKAP1 | 1 |
| MAPKAPK3 | 1 |
| MAPKAPK5.AS1 | 1 |
| MAPKBP1 | 1 |
| MAPRE1 | 1 |
| MAPRE3 | 1 |
| 1-Mar | 1 |
| 2-Mar | 1 |
| 4-Mar | 1 |
| 5-Mar | 1 |
| 9-Mar | 1 |
| MARCKS | 1 |
| MARCKSL1 | 1 |
| MARK4 | 1 |
| MARS2 | 1 |
| MARVELD1 | 1 |
| MARVELD3 | 1 |
| MAS1 | 1 |
| MASP1 | 1 |
| MAST4 | 1 |
| MAT2A | 1 |
| MATR3 | 1 |
| MAVS | 1 |
| MBD3L5 | 1 |
| MBNL1 | 1 |
| MBOAT7 | 1 |
| MBTD1 | 1 |
| MBTPS1 | 1 |
| MC3R | 1 |
| MCAT | 1 |
| MCF2L2 | 1 |
| MCFD2 | 1 |
| MCL1 | 1 |
| MCM10 | 1 |
| MCM2 | 1 |
| MCM3AP.AS1 | 1 |
| MCM5 | 1 |
| MCM6 | 1 |
| MCM8 | 1 |
| MCMBP | 1 |
| MCOLN2 | 1 |
| MCUR1 | 1 |
| MDFI | 1 |
| MDH1 | 1 |
| MDH1B | 1 |
| MDH2 | 1 |
| MDM2 | 1 |
| MEA1 | 1 |
| MECOM | 1 |
| MECR | 1 |
| MED12 | 1 |
| MED16 | 1 |
| MED19 | 1 |
| MED22 | 1 |
| MED27 | 1 |
| MED28 | 1 |
| MED30 | 1 |
| MED31 | 1 |
| MED4 | 1 |
| MED6 | 1 |
| MEDAG | 1 |
| MEF2B | 1 |
| MEF2C | 1 |
| MEGF6 | 1 |
| MEIOB | 1 |
| MEIS1 | 1 |
| MELK | 1 |
| MEN1 | 1 |
| MEOX2 | 1 |
| MEP1B | 1 |
| MEPCE | 1 |
| MEPE | 1 |
| MERTK | 1 |
| MET | 1 |
| METAP1 | 1 |
| METAP2 | 1 |
| METRNL | 1 |
| METTL10 | 1 |
| METTL13 | 1 |
| METTL15 | 1 |
| METTL17 | 1 |
| METTL20 | 1 |
| METTL21A | 1 |
| METTL23 | 1 |
| METTL24 | 1 |
| METTL2A | 1 |
| METTL5 | 1 |
| METTL9 | 1 |
| MEX3B | 1 |
| MFAP1 | 1 |
| MFAP5 | 1 |
| MFF | 1 |
| MFGE8 | 1 |
| MFSD1 | 1 |
| MFSD10 | 1 |
| MFSD5 | 1 |
| MGA | 1 |
| MGAM | 1 |
| MGARP | 1 |
| MGAT2 | 1 |
| MGAT3 | 1 |
| MGAT4A | 1 |
| MGAT4B | 1 |
| MGC16025 | 1 |
| MGC27345 | 1 |
| MGC32805 | 1 |
| MGC40069 | 1 |
| MGC72080 | 1 |
| MGLL | 1 |
| MGRN1 | 1 |
| MGST1 | 1 |
| MGST2 | 1 |
| MGST3 | 1 |
| MICA | 1 |
| MICAL1 | 1 |
| MICB | 1 |
| MICU2 | 1 |
| MIEF1 | 1 |
| MIEN1 | 1 |
| MIER1 | 1 |
| MIF | 1 |
| MINK1 | 1 |
| MINOS1 | 1 |
| MINPP1 | 1 |
| MIOS | 1 |
| MIP | 1 |
| MIPEP | 1 |
| MIR100 | 1 |
| MIR106B | 1 |
| MIR124.1 | 1 |
| MIR125A | 1 |
| MIR128.1 | 1 |
| MIR129.1 | 1 |
| MIR140 | 1 |
| MIR145 | 1 |
| MIR148B | 1 |
| MIR150 | 1 |
| MIR153.1 | 1 |
| MIR154 | 1 |
| MIR15A | 1 |
| MIR181A1 | 1 |
| MIR181A2 | 1 |
| MIR181B1 | 1 |
| MIR181B2 | 1 |
| MIR182 | 1 |
| MIR185 | 1 |
| MIR188 | 1 |
| MIR191 | 1 |
| MIR193A | 1 |
| MIR194.1 | 1 |
| MIR197 | 1 |
| MIR199A1 | 1 |
| MIR200A | 1 |
| MIR202 | 1 |
| MIR204 | 1 |
| MIR205 | 1 |
| MIR206 | 1 |
| MIR211 | 1 |
| MIR216A | 1 |
| MIR218.1 | 1 |
| MIR218.2 | 1 |
| MIR22HG | 1 |
| MIR26A1 | 1 |
| MIR28 | 1 |
| MIR296 | 1 |
| MIR29A | 1 |
| MIR30C1 | 1 |
| MIR31HG | 1 |
| MIR32 | 1 |
| MIR323B | 1 |
| MIR377 | 1 |
| MIR382 | 1 |
| MIR410 | 1 |
| MIR412 | 1 |
| MIR425 | 1 |
| MIR429 | 1 |
| MIR487A | 1 |
| MIR492 | 1 |
| MIR494 | 1 |
| MIR600HG | 1 |
| MIR646HG | 1 |
| MIR9.1 | 1 |
| MIR99B | 1 |
| MIRLET7A1 | 1 |
| MIRLET7D | 1 |
| MIRLET7F2 | 1 |
| MIS18A | 1 |
| MKL2 | 1 |
| MKX | 1 |
| MLANA | 1 |
| MLC1 | 1 |
| MLEC | 1 |
| MLF2 | 1 |
| MLH1 | 1 |
| MLH3 | 1 |
| MLIP | 1 |
| MLKL | 1 |
| MLLT1 | 1 |
| MLLT4 | 1 |
| MLST8 | 1 |
| MLX | 1 |
| MLXIP | 1 |
| MMADHC | 1 |
| MMGT1 | 1 |
| MMP1 | 1 |
| MMP11 | 1 |
| MMP13 | 1 |
| MMP17 | 1 |
| MMP2 | 1 |
| MMP24 | 1 |
| MMP3 | 1 |
| MMP9 | 1 |
| MMRN2 | 1 |
| MNDA | 1 |
| MOB2 | 1 |
| MOB3A | 1 |
| MOB3B | 1 |
| MOB4 | 1 |
| MOCS2 | 1 |
| MOCS3 | 1 |
| MOGAT1 | 1 |
| MOGS | 1 |
| MOK | 1 |
| MON1A | 1 |
| MON1B | 1 |
| MOP.1 | 1 |
| MORC1 | 1 |
| MORC2 | 1 |
| MORC4 | 1 |
| MORF4L1 | 1 |
| MORF4L2 | 1 |
| MORN1 | 1 |
| MORN2 | 1 |
| MORN4 | 1 |
| MORN5 | 1 |
| MOSPD3 | 1 |
| MOV10 | 1 |
| MOXD1 | 1 |
| MPC1 | 1 |
| MPC2 | 1 |
| MPDU1 | 1 |
| MPEG1 | 1 |
| MPG | 1 |
| MPHOSPH6 | 1 |
| MPHOSPH9 | 1 |
| MPI | 1 |
| MPLKIP | 1 |
| MPP5 | 1 |
| MPPED2 | 1 |
| MPST | 1 |
| MPV17 | 1 |
| MPV17L2 | 1 |
| MPZL1 | 1 |
| MPZL2 | 1 |
| MRAP | 1 |
| MRAP2 | 1 |
| MRC1 | 1 |
| MRFAP1 | 1 |
| MRGPRF | 1 |
| MRGPRG.AS1 | 1 |
| MRGPRG | 1 |
| MRGPRX1 | 1 |
| MRO | 1 |
| MROH2A | 1 |
| MROH7 | 1 |
| MRPL10 | 1 |
| MRPL11 | 1 |
| MRPL14 | 1 |
| MRPL15 | 1 |
| MRPL16 | 1 |
| MRPL17 | 1 |
| MRPL18 | 1 |
| MRPL19 | 1 |
| MRPL22 | 1 |
| MRPL23 | 1 |
| MRPL24 | 1 |
| MRPL27 | 1 |
| MRPL28 | 1 |
| MRPL3 | 1 |
| MRPL30 | 1 |
| MRPL33 | 1 |
| MRPL36 | 1 |
| MRPL37 | 1 |
| MRPL38 | 1 |
| MRPL40 | 1 |
| MRPL41 | 1 |
| MRPL42 | 1 |
| MRPL45 | 1 |
| MRPL46 | 1 |
| MRPL47 | 1 |
| MRPL49 | 1 |
| MRPL51 | 1 |
| MRPL53 | 1 |
| MRPL57 | 1 |
| MRPL9 | 1 |
| MRPS10 | 1 |
| MRPS11 | 1 |
| MRPS15 | 1 |
| MRPS16 | 1 |
| MRPS17 | 1 |
| MRPS18A | 1 |
| MRPS2 | 1 |
| MRPS22 | 1 |
| MRPS23 | 1 |
| MRPS26 | 1 |
| MRPS27 | 1 |
| MRPS28 | 1 |
| MRPS34 | 1 |
| MRPS35 | 1 |
| MRPS36 | 1 |
| MRPS7 | 1 |
| MRVI1.AS1 | 1 |
| MS4A10 | 1 |
| MS4A14 | 1 |
| MS4A3 | 1 |
| MS4A4A | 1 |
| MS4A5 | 1 |
| MS4A6A | 1 |
| MS4A6E | 1 |
| MS4A8 | 1 |
| MSANTD2 | 1 |
| MSANTD3.TMEFF1 | 1 |
| MSH4 | 1 |
| MSH5.SAPCD1 | 1 |
| MSH6 | 1 |
| MSMB | 1 |
| MSN | 1 |
| MSR1 | 1 |
| MSRA | 1 |
| MSRB1 | 1 |
| MSRB2 | 1 |
| MSS51 | 1 |
| MSTN | 1 |
| MT1M | 1 |
| MT1X | 1 |
| MT2A | 1 |
| MTA2 | 1 |
| MTCH2 | 1 |
| MTDH | 1 |
| MTERF2 | 1 |
| MTF2 | 1 |
| MTFR2 | 1 |
| MTG1 | 1 |
| MTG2 | 1 |
| MTHFD2L | 1 |
| MTMR10 | 1 |
| MTMR6 | 1 |
| MTMR9 | 1 |
| MTO1 | 1 |
| MTRF1L | 1 |
| MTTP | 1 |
| MTUS1 | 1 |
| MUC13 | 1 |
| MUCL1 | 1 |
| MUL1 | 1 |
| MVB12A | 1 |
| MVD | 1 |
| MVP | 1 |
| MX1 | 1 |
| MXD3 | 1 |
| MXI1 | 1 |
| MXRA5 | 1 |
| MXRA7 | 1 |
| MXRA8 | 1 |
| MYBBP1A | 1 |
| MYBL1 | 1 |
| MYBPC1 | 1 |
| MYCT1 | 1 |
| MYEOV2 | 1 |
| MYF6 | 1 |
| MYH1 | 1 |
| MYH13 | 1 |
| MYH9 | 1 |
| MYL1 | 1 |
| MYL12A | 1 |
| MYL12B | 1 |
| MYL3 | 1 |
| MYL6 | 1 |
| MYL7 | 1 |
| MYL9 | 1 |
| MYLIP | 1 |
| MYLK4 | 1 |
| MYO1B | 1 |
| MYO1C | 1 |
| MYO1E | 1 |
| MYO1F | 1 |
| MYO3A | 1 |
| MYO3B | 1 |
| MYO5C | 1 |
| MYO7A | 1 |
| MYOC | 1 |
| MYOD1 | 1 |
| MYOF | 1 |
| MYOM2 | 1 |
| MYOT | 1 |
| MYPN | 1 |
| MYRF | 1 |
| MYRFL | 1 |
| MYSM1 | 1 |
| MZT2B | 1 |
| N4BP2 | 1 |
| N6AMT2 | 1 |
| NAA10 | 1 |
| NAA11 | 1 |
| NAA15 | 1 |
| NAA16 | 1 |
| NAA50 | 1 |
| NAAA | 1 |
| NABP1 | 1 |
| NACAP1 | 1 |
| NAE1 | 1 |
| NAGA | 1 |
| NAGK | 1 |
| NAGLU | 1 |
| NAGPA | 1 |
| NAIF1 | 1 |
| NAIP | 1 |
| NANP | 1 |
| NANS | 1 |
| NAP1L3 | 1 |
| NAP1L4 | 1 |
| NAPA | 1 |
| NAT1 | 1 |
| NAT10 | 1 |
| NAT14 | 1 |
| NAT2 | 1 |
| NAT8 | 1 |
| NAT8B | 1 |
| NBEA | 1 |
| NBEAL2 | 1 |
| NBN | 1 |
| NBPF3 | 1 |
| NBR1 | 1 |
| NCALD | 1 |
| NCAM1 | 1 |
| NCAPD2 | 1 |
| NCAPG | 1 |
| NCBP2 | 1 |
| NCEH1 | 1 |
| NCF4 | 1 |
| NCKAP1 | 1 |
| NCKAP1L | 1 |
| NCKAP5 | 1 |
| NCL | 1 |
| NCOA1 | 1 |
| NCOR1 | 1 |
| NCSTN | 1 |
| NDFIP2 | 1 |
| NDOR1 | 1 |
| NDRG2 | 1 |
| NDRG3 | 1 |
| NDRG4 | 1 |
| NDST1 | 1 |
| NDST3 | 1 |
| NDUFA11 | 1 |
| NDUFA12 | 1 |
| NDUFA3 | 1 |
| NDUFA4 | 1 |
| NDUFA6 | 1 |
| NDUFA9 | 1 |
| NDUFAB1 | 1 |
| NDUFAF1 | 1 |
| NDUFAF3 | 1 |
| NDUFAF4 | 1 |
| NDUFAF7 | 1 |
| NDUFB1 | 1 |
| NDUFB10 | 1 |
| NDUFB2 | 1 |
| NDUFB3 | 1 |
| NDUFB5 | 1 |
| NDUFB6 | 1 |
| NDUFB7 | 1 |
| NDUFC2 | 1 |
| NDUFS1 | 1 |
| NDUFS2 | 1 |
| NDUFS3 | 1 |
| NDUFS4 | 1 |
| NDUFS5 | 1 |
| NDUFS6 | 1 |
| NDUFS7 | 1 |
| NDUFV1 | 1 |
| NEB | 1 |
| NECAB3 | 1 |
| NECAP2 | 1 |
| NEDD1 | 1 |
| NEDD4L | 1 |
| NEDD8 | 1 |
| NEGR1 | 1 |
| NEIL1 | 1 |
| NEIL2 | 1 |
| NEIL3 | 1 |
| NEK10 | 1 |
| NEK5 | 1 |
| NEK7 | 1 |
| NELFB | 1 |
| NEO1 | 1 |
| NES | 1 |
| NET1 | 1 |
| NETO1 | 1 |
| NETO2 | 1 |
| NEU3 | 1 |
| NEURL2 | 1 |
| NEURL4 | 1 |
| NEUROD6 | 1 |
| NEUROG2 | 1 |
| NEXN.AS1 | 1 |
| NF2 | 1 |
| NFAM1 | 1 |
| NFASC | 1 |
| NFATC1 | 1 |
| NFATC2 | 1 |
| NFATC2IP | 1 |
| NFE2L1 | 1 |
| NFE2L2 | 1 |
| NFE2L3 | 1 |
| NFIC | 1 |
| NFKB1 | 1 |
| NFKBIA | 1 |
| NFKBIB | 1 |
| NFKBIE | 1 |
| NFKBIZ | 1 |
| NFS1 | 1 |
| NGFRAP1 | 1 |
| NHLH2 | 1 |
| NHP2 | 1 |
| NHP2L1 | 1 |
| NHSL2 | 1 |
| NICN1 | 1 |
| NID1 | 1 |
| NIN | 1 |
| NIP7 | 1 |
| NIPA2 | 1 |
| NIPAL2 | 1 |
| NIPAL3 | 1 |
| NIPSNAP1 | 1 |
| NIPSNAP3A | 1 |
| NIPSNAP3B | 1 |
| NKAIN3 | 1 |
| NKAPP1 | 1 |
| NKIRAS1 | 1 |
| NKX2.3 | 1 |
| NKX2.8 | 1 |
| NKX3.1 | 1 |
| NKX6.2 | 1 |
| NLGN1 | 1 |
| NLGN3 | 1 |
| NLGN4X | 1 |
| NLN | 1 |
| NLRC4 | 1 |
| NLRP10 | 1 |
| NLRP3 | 1 |
| NLRX1 | 1 |
| NMD3 | 1 |
| NME1 | 1 |
| NME4 | 1 |
| NMRAL1 | 1 |
| NMRK1 | 1 |
| NMT1 | 1 |
| NMT2 | 1 |
| NMUR2 | 1 |
| NNT | 1 |
| NOA1 | 1 |
| NOB1 | 1 |
| NOC3L | 1 |
| NOC4L | 1 |
| NOD1 | 1 |
| NODAL | 1 |
| NOL3 | 1 |
| NOL4L | 1 |
| NOL6 | 1 |
| NOL7 | 1 |
| NOL8 | 1 |
| NOP14 | 1 |
| NOP16 | 1 |
| NOS1AP | 1 |
| NOS2 | 1 |
| NOSTRIN | 1 |
| NOTCH4 | 1 |
| NOV | 1 |
| NOVA1 | 1 |
| NOVA2 | 1 |
| NOX1 | 1 |
| NOX3 | 1 |
| NOX4 | 1 |
| NPAP1 | 1 |
| NPAT | 1 |
| NPC1 | 1 |
| NPCDR1 | 1 |
| NPDC1 | 1 |
| NPEPPS | 1 |
| NPFF | 1 |
| NPFFR2 | 1 |
| NPHS1 | 1 |
| NPL | 1 |
| NPLOC4 | 1 |
| NPM1 | 1 |
| NPNT | 1 |
| NPPA | 1 |
| NPPB | 1 |
| NPR1 | 1 |
| NPR2 | 1 |
| NPR3 | 1 |
| NPRL3 | 1 |
| NPTN | 1 |
| NPTX1 | 1 |
| NPTX2 | 1 |
| NPY1R | 1 |
| NPY6R | 1 |
| NQO1 | 1 |
| NR1H2 | 1 |
| NR1I3 | 1 |
| NR2C2AP | 1 |
| NR2F6 | 1 |
| NR3C1 | 1 |
| NR3C2 | 1 |
| NR4A1 | 1 |
| NR4A2 | 1 |
| NR4A3 | 1 |
| NR5A2 | 1 |
| NR6A1 | 1 |
| NRARP | 1 |
| NRAS | 1 |
| NRBF2 | 1 |
| NRBP1 | 1 |
| NRCAM | 1 |
| NRF1 | 1 |
| NRG1 | 1 |
| NRG3 | 1 |
| NRIP1 | 1 |
| NRIP3 | 1 |
| NRN1 | 1 |
| NRP2 | 1 |
| NRSN1 | 1 |
| NRSN2 | 1 |
| NRXN3 | 1 |
| NSDHL | 1 |
| NSFL1C | 1 |
| NSMAF | 1 |
| NSMCE1 | 1 |
| NSUN2 | 1 |
| NSUN5 | 1 |
| NSUN5P1 | 1 |
| NSUN6 | 1 |
| NSUN7 | 1 |
| NT5C3A | 1 |
| NT5DC1 | 1 |
| NT5DC3 | 1 |
| NT5E | 1 |
| NTAN1 | 1 |
| NTF3 | 1 |
| NTF4 | 1 |
| NTHL1 | 1 |
| NTMT1 | 1 |
| NTN4 | 1 |
| NTNG2 | 1 |
| NTPCR | 1 |
| NTS | 1 |
| NUAK2 | 1 |
| NUBP1 | 1 |
| NUCB1 | 1 |
| NUDC | 1 |
| NUDCD2 | 1 |
| NUDT12 | 1 |
| NUDT15 | 1 |
| NUDT16 | 1 |
| NUDT16L1 | 1 |
| NUDT2 | 1 |
| NUDT21 | 1 |
| NUDT3 | 1 |
| NUDT4 | 1 |
| NUDT5 | 1 |
| NUDT6 | 1 |
| NUDT9P1 | 1 |
| NUP188 | 1 |
| NUP210 | 1 |
| NUP210L | 1 |
| NUP35 | 1 |
| NUP37 | 1 |
| NUP43 | 1 |
| NUP50 | 1 |
| NUP54 | 1 |
| NUP88 | 1 |
| NUP93 | 1 |
| NUP98 | 1 |
| NUPL2 | 1 |
| NUPR1 | 1 |
| NUSAP1 | 1 |
| NUTF2 | 1 |
| NUTF2P4 | 1 |
| NWD1 | 1 |
| NXF4 | 1 |
| NXF5 | 1 |
| NXNL2 | 1 |
| NXPE4 | 1 |
| NXPH3 | 1 |
| NYAP2 | 1 |
| OAF | 1 |
| OAS1 | 1 |
| OASL | 1 |
| OAT | 1 |
| OAZ1 | 1 |
| OAZ2 | 1 |
| OAZ3 | 1 |
| OBFC1 | 1 |
| OBP2A | 1 |
| OCA2 | 1 |
| OCEL1 | 1 |
| OCIAD1 | 1 |
| OCLM | 1 |
| OCR1 | 1 |
| OCRL | 1 |
| OCSTAMP | 1 |
| ODC1 | 1 |
| ODF4 | 1 |
| OFD1 | 1 |
| OGDH | 1 |
| OGFOD1 | 1 |
| OGFR | 1 |
| OGT | 1 |
| OIT3 | 1 |
| OLFM1 | 1 |
| OLFM2 | 1 |
| OLFM4 | 1 |
| OLFML1 | 1 |
| OMD | 1 |
| OOSP2 | 1 |
| OPRL1 | 1 |
| OPRM1 | 1 |
| OPTN | 1 |
| OR10AG1 | 1 |
| OR10G3 | 1 |
| OR10G7 | 1 |
| OR10H1 | 1 |
| OR10H3 | 1 |
| OR10H4 | 1 |
| OR10J3 | 1 |
| OR10K2 | 1 |
| OR10T2 | 1 |
| OR10W1 | 1 |
| OR10X1 | 1 |
| OR12D2 | 1 |
| OR12D3 | 1 |
| OR13C3 | 1 |
| OR13F1 | 1 |
| OR14A16 | 1 |
| OR14C36 | 1 |
| OR14I1 | 1 |
| OR1E2 | 1 |
| OR1L4 | 1 |
| OR1Q1 | 1 |
| OR1S2 | 1 |
| OR2A12 | 1 |
| OR2A25 | 1 |
| OR2AK2 | 1 |
| OR2B11 | 1 |
| OR2B3 | 1 |
| OR2B6 | 1 |
| OR2C3 | 1 |
| OR2F1 | 1 |
| OR2L3 | 1 |
| OR2L5 | 1 |
| OR2M2 | 1 |
| OR2M4 | 1 |
| OR2M5 | 1 |
| OR2S2 | 1 |
| OR2T10 | 1 |
| OR2T29 | 1 |
| OR2T34 | 1 |
| OR2T4 | 1 |
| OR2W1 | 1 |
| OR4A16 | 1 |
| OR4A5 | 1 |
| OR4B1 | 1 |
| OR4C13 | 1 |
| OR4C15 | 1 |
| OR4E2 | 1 |
| OR4F4 | 1 |
| OR4N2 | 1 |
| OR4P4 | 1 |
| OR4Q3 | 1 |
| OR4X1 | 1 |
| OR51A4 | 1 |
| OR51F2 | 1 |
| OR51G2 | 1 |
| OR51M1 | 1 |
| OR52A1 | 1 |
| OR52A5 | 1 |
| OR52E4 | 1 |
| OR52I2 | 1 |
| OR52K2 | 1 |
| OR52N5 | 1 |
| OR52R1 | 1 |
| OR56A1 | 1 |
| OR56A4 | 1 |
| OR5A1 | 1 |
| OR5A2 | 1 |
| OR5AC2 | 1 |
| OR5AN1 | 1 |
| OR5B12 | 1 |
| OR5B17 | 1 |
| OR5B2 | 1 |
| OR5B21 | 1 |
| OR5D13 | 1 |
| OR5D14 | 1 |
| OR5D16 | 1 |
| OR5E1P | 1 |
| OR5H6 | 1 |
| OR5J2 | 1 |
| OR5K2 | 1 |
| OR5K3 | 1 |
| OR5K4 | 1 |
| OR5L1 | 1 |
| OR5L2 | 1 |
| OR5P2 | 1 |
| OR5R1 | 1 |
| OR5T2 | 1 |
| OR6C4 | 1 |
| OR6C6 | 1 |
| OR6C65 | 1 |
| OR6C70 | 1 |
| OR6K6 | 1 |
| OR6N1 | 1 |
| OR6S1 | 1 |
| OR6T1 | 1 |
| OR7D2 | 1 |
| OR7G1 | 1 |
| OR7G2 | 1 |
| OR8A1 | 1 |
| OR8B12 | 1 |
| OR8B3 | 1 |
| OR8B4 | 1 |
| OR8D1 | 1 |
| OR8D4 | 1 |
| OR8G5 | 1 |
| OR8J1 | 1 |
| OR8J3 | 1 |
| OR8K1 | 1 |
| OR8K5 | 1 |
| OR8S1 | 1 |
| OR9A2 | 1 |
| OR9G4 | 1 |
| OR9I1 | 1 |
| OR9K2 | 1 |
| OR9Q1 | 1 |
| ORAI2 | 1 |
| ORC6 | 1 |
| ORMDL2 | 1 |
| OSBP2 | 1 |
| OSBPL10 | 1 |
| OSBPL3 | 1 |
| OSBPL6 | 1 |
| OSCAR | 1 |
| OSGIN2 | 1 |
| OSR1 | 1 |
| OSR2 | 1 |
| OSTC | 1 |
| OTOR | 1 |
| OTP | 1 |
| OTUD1 | 1 |
| OTUD3 | 1 |
| OTUD7A | 1 |
| OTUD7B | 1 |
| OTX1 | 1 |
| OVCA2 | 1 |
| OVGP1 | 1 |
| OXLD1 | 1 |
| OXSR1 | 1 |
| P2RX1 | 1 |
| P2RX5 | 1 |
| P2RY1 | 1 |
| P2RY10 | 1 |
| P2RY12 | 1 |
| P2RY13 | 1 |
| P2RY14 | 1 |
| P2RY6 | 1 |
| P2RY8 | 1 |
| P4HTM | 1 |
| PA2G4 | 1 |
| PAAF1 | 1 |
| PABPN1 | 1 |
| PACSIN2 | 1 |
| PACSIN3 | 1 |
| PADI2 | 1 |
| PAEP | 1 |
| PAF1 | 1 |
| PAFAH1B1 | 1 |
| PAFAH1B2 | 1 |
| PAFAH1B3 | 1 |
| PAFAH2 | 1 |
| PAICS | 1 |
| PAIP2 | 1 |
| PAIP2B | 1 |
| PAK1IP1 | 1 |
| PAK4 | 1 |
| PAK6 | 1 |
| PALD1 | 1 |
| PALLD | 1 |
| PALM | 1 |
| PALMD | 1 |
| PAM | 1 |
| PAMR1 | 1 |
| PAN2 | 1 |
| PANK2 | 1 |
| PANX1 | 1 |
| PANX3 | 1 |
| PAPPA | 1 |
| PAPPA2 | 1 |
| PAPSS1 | 1 |
| PAPSS2 | 1 |
| PAQR3 | 1 |
| PAQR4 | 1 |
| PAQR5 | 1 |
| PAQR6 | 1 |
| PARD6G | 1 |
| PARK2 | 1 |
| PARM1 | 1 |
| PARP12 | 1 |
| PARP15 | 1 |
| PARP3 | 1 |
| PARP6 | 1 |
| PART1 | 1 |
| PARVA | 1 |
| PARVB | 1 |
| PARVG | 1 |
| PATE1 | 1 |
| PATE2 | 1 |
| PATL2 | 1 |
| PAWR | 1 |
| PAX1 | 1 |
| PAX6 | 1 |
| PAXBP1 | 1 |
| PAXIP1 | 1 |
| PBDC1 | 1 |
| PBK | 1 |
| PBRM1 | 1 |
| PBX4 | 1 |
| PCBD1 | 1 |
| PCBD2 | 1 |
| PCBP1 | 1 |
| PCDH1 | 1 |
| PCDH10 | 1 |
| PCDH12 | 1 |
| PCDH17 | 1 |
| PCDH19 | 1 |
| PCDH7 | 1 |
| PCDH9 | 1 |
| PCDHB18 | 1 |
| PCDHB4 | 1 |
| PCDP1 | 1 |
| PCED1A | 1 |
| PCGF1 | 1 |
| PCMT1 | 1 |
| PCNA | 1 |
| PCNP | 1 |
| PCSK7 | 1 |
| PCYOX1 | 1 |
| PCYT1A | 1 |
| PCYT1B | 1 |
| PCYT2 | 1 |
| PDAP1 | 1 |
| PDC | 1 |
| PDCD1LG2 | 1 |
| PDCD6IP | 1 |
| PDCL2 | 1 |
| PDDC1 | 1 |
| PDE10A | 1 |
| PDE11A | 1 |
| PDE12 | 1 |
| PDE1A | 1 |
| PDE1C | 1 |
| PDE2A | 1 |
| PDE4D | 1 |
| PDE5A | 1 |
| PDE6C | 1 |
| PDE7A | 1 |
| PDE8A | 1 |
| PDE9A | 1 |
| PDGFB | 1 |
| PDGFD | 1 |
| PDGFRA | 1 |
| PDHB | 1 |
| PDIA3 | 1 |
| PDIA6 | 1 |
| PDK3 | 1 |
| PDK4 | 1 |
| PDLIM2 | 1 |
| PDLIM5 | 1 |
| PDP1 | 1 |
| PDSS2 | 1 |
| PDXDC2P | 1 |
| PDZD2 | 1 |
| PDZD4 | 1 |
| PDZD8 | 1 |
| PDZD9 | 1 |
| PDZRN3 | 1 |
| PEA15 | 1 |
| PEBP1 | 1 |
| PECAM1 | 1 |
| PELI1 | 1 |
| PELI2 | 1 |
| PELP1 | 1 |
| PENK | 1 |
| PEPD | 1 |
| PER2 | 1 |
| PERP | 1 |
| PES1 | 1 |
| PET112 | 1 |
| PEX11G | 1 |
| PEX12 | 1 |
| PEX13 | 1 |
| PEX19 | 1 |
| PEX26 | 1 |
| PEX3 | 1 |
| PEX5L | 1 |
| PEX6 | 1 |
| PEX7 | 1 |
| PF4 | 1 |
| PFDN1 | 1 |
| PFDN5 | 1 |
| PFKFB1 | 1 |
| PFKL | 1 |
| PFKM | 1 |
| PFKP | 1 |
| PFN1 | 1 |
| PGAM5 | 1 |
| PGAP2 | 1 |
| PGBD4 | 1 |
| PGBD5 | 1 |
| PGD | 1 |
| PGF | 1 |
| PGK1 | 1 |
| PGK2 | 1 |
| PGLS | 1 |
| PGLYRP2 | 1 |
| PGLYRP3 | 1 |
| PGM1 | 1 |
| PGP | 1 |
| PGPEP1 | 1 |
| PGRMC1 | 1 |
| PGRMC2 | 1 |
| PHACTR1 | 1 |
| PHACTR2 | 1 |
| PHAX | 1 |
| PHB | 1 |
| PHB2 | 1 |
| PHC3 | 1 |
| PHEX | 1 |
| PHF11 | 1 |
| PHF14 | 1 |
| PHF19 | 1 |
| PHF2 | 1 |
| PHF20 | 1 |
| PHF20L1 | 1 |
| PHF21A | 1 |
| PHF21B | 1 |
| PHF23 | 1 |
| PHF3 | 1 |
| PHF5A | 1 |
| PHF8 | 1 |
| PHKA2 | 1 |
| PHKB | 1 |
| PHKG1 | 1 |
| PHKG2 | 1 |
| PHLDA1 | 1 |
| PHLDA2 | 1 |
| PHLDA3 | 1 |
| PHOSPHO2 | 1 |
| PHOX2B | 1 |
| PHPT1 | 1 |
| PHYH | 1 |
| PHYHD1 | 1 |
| PI4K2A | 1 |
| PI4K2B | 1 |
| PIBF1 | 1 |
| PICALM | 1 |
| PIEZO2 | 1 |
| PIGB | 1 |
| PIGG | 1 |
| PIGK | 1 |
| PIGL | 1 |
| PIGQ | 1 |
| PIGS | 1 |
| PIGU | 1 |
| PIGV | 1 |
| PIGW | 1 |
| PIH1D1 | 1 |
| PIK3AP1 | 1 |
| PIK3C2B | 1 |
| PIK3CG | 1 |
| PIK3R2 | 1 |
| PIK3R3 | 1 |
| PIK3R5 | 1 |
| PIKFYVE | 1 |
| PIN1 | 1 |
| PIN1P1 | 1 |
| PIN4 | 1 |
| PINK1 | 1 |
| PINX1 | 1 |
| PIP4K2B | 1 |
| PIP5K1B | 1 |
| PIP5K1C | 1 |
| PIP5KL1 | 1 |
| PITHD1 | 1 |
| PITPNA | 1 |
| PITPNB | 1 |
| PITRM1 | 1 |
| PITX1 | 1 |
| PITX2 | 1 |
| PIWIL2 | 1 |
| PIWIL4 | 1 |
| PJA2 | 1 |
| PKD1L1 | 1 |
| PKD2 | 1 |
| PKHD1L1 | 1 |
| PKIA | 1 |
| PKM | 1 |
| PKMYT1 | 1 |
| PKN3 | 1 |
| PLA2G15 | 1 |
| PLA2G1B | 1 |
| PLA2G2C | 1 |
| PLA2G4C | 1 |
| PLA2G5 | 1 |
| PLA2G6 | 1 |
| PLA2G7 | 1 |
| PLAA | 1 |
| PLAC8L1 | 1 |
| PLAGL2 | 1 |
| PLAT | 1 |
| PLAUR | 1 |
| PLB1 | 1 |
| PLBD1 | 1 |
| PLBD2 | 1 |
| PLCB2 | 1 |
| PLCB3 | 1 |
| PLCD1 | 1 |
| PLCD3 | 1 |
| PLCG2 | 1 |
| PLCL1 | 1 |
| PLCL2 | 1 |
| PLCXD2 | 1 |
| PLD1 | 1 |
| PLD2 | 1 |
| PLEC | 1 |
| PLEK | 1 |
| PLEKHA4 | 1 |
| PLEKHA5 | 1 |
| PLEKHB2 | 1 |
| PLEKHG1 | 1 |
| PLEKHG5 | 1 |
| PLEKHH1 | 1 |
| PLEKHM3 | 1 |
| PLGLB1 | 1 |
| PLGRKT | 1 |
| PLIN2 | 1 |
| PLIN3 | 1 |
| PLK1 | 1 |
| PLK2 | 1 |
| PLK5 | 1 |
| PLN | 1 |
| PLOD3 | 1 |
| PLP2 | 1 |
| PLRG1 | 1 |
| PLSCR2 | 1 |
| PLSCR3 | 1 |
| PLSCR4 | 1 |
| PLVAP | 1 |
| PLXDC1 | 1 |
| PLXDC2 | 1 |
| PLXNA1 | 1 |
| PLXNA2 | 1 |
| PLXNB1 | 1 |
| PLXNB2 | 1 |
| PLXNC1 | 1 |
| PLXND1 | 1 |
| PM20D2 | 1 |
| PML | 1 |
| PMM1 | 1 |
| PMPCA | 1 |
| PMPCB | 1 |
| PMS2CL | 1 |
| PMS2P3 | 1 |
| PMVK | 1 |
| PNISR | 1 |
| PNLIP | 1 |
| PNMA1 | 1 |
| PNMA2 | 1 |
| PNN | 1 |
| PNP | 1 |
| PNPLA2 | 1 |
| PNPLA3 | 1 |
| PNPLA6 | 1 |
| PNPLA7 | 1 |
| PNPO | 1 |
| PNRC1 | 1 |
| PNRC2 | 1 |
| POC1A | 1 |
| POC1B.GALNT4 | 1 |
| PODN | 1 |
| PODXL2 | 1 |
| POF1B | 1 |
| POFUT1 | 1 |
| POGLUT1 | 1 |
| POGZ | 1 |
| POLD1 | 1 |
| POLD2 | 1 |
| POLD4 | 1 |
| POLDIP2 | 1 |
| POLDIP3 | 1 |
| POLE3 | 1 |
| POLE4 | 1 |
| POLH | 1 |
| POLM | 1 |
| POLR1D | 1 |
| POLR1E | 1 |
| POLR2C | 1 |
| POLR2D | 1 |
| POLR2E | 1 |
| POLR2F | 1 |
| POLR2I | 1 |
| POLR2J | 1 |
| POLR2K | 1 |
| POLR2L | 1 |
| POLR3A | 1 |
| POLR3F | 1 |
| POLR3H | 1 |
| POLR3K | 1 |
| POM121L12 | 1 |
| POM121L2 | 1 |
| POMGNT1 | 1 |
| POMP | 1 |
| POMT1 | 1 |
| PON1 | 1 |
| PON2 | 1 |
| PON3 | 1 |
| POP5 | 1 |
| POPDC2 | 1 |
| POPDC3 | 1 |
| PORCN | 1 |
| POSTN | 1 |
| POTEE | 1 |
| POTEJ | 1 |
| POTEKP | 1 |
| POTEM | 1 |
| POU1F1 | 1 |
| POU2F1 | 1 |
| POU4F3 | 1 |
| POU5F1B | 1 |
| POU5F2 | 1 |
| POU6F1 | 1 |
| PP13 | 1 |
| PP13439 | 1 |
| PP2D1 | 1 |
| PPA1 | 1 |
| PPAP2A | 1 |
| PPAP2B | 1 |
| PPAP2C | 1 |
| PPARGC1B | 1 |
| PPBP | 1 |
| PPBPP2 | 1 |
| PPCDC | 1 |
| PPCS | 1 |
| PPDPF | 1 |
| PPEF1 | 1 |
| PPFIA1 | 1 |
| PPFIA2 | 1 |
| PPFIBP1 | 1 |
| PPHLN1 | 1 |
| PPIB | 1 |
| PPID | 1 |
| PPIF | 1 |
| PPIL3 | 1 |
| PPIL4 | 1 |
| PPIP5K2 | 1 |
| PPL | 1 |
| PPM1E | 1 |
| PPM1K | 1 |
| PPM1L | 1 |
| PPME1 | 1 |
| PPP1CA | 1 |
| PPP1CC | 1 |
| PPP1R12A | 1 |
| PPP1R12B | 1 |
| PPP1R13B | 1 |
| PPP1R14C | 1 |
| PPP1R15A | 1 |
| PPP1R17 | 1 |
| PPP1R2P3 | 1 |
| PPP1R2P9 | 1 |
| PPP1R37 | 1 |
| PPP1R3E | 1 |
| PPP1R7 | 1 |
| PPP1R8 | 1 |
| PPP2CA | 1 |
| PPP2CB | 1 |
| PPP2R1A | 1 |
| PPP2R2A | 1 |
| PPP2R2B | 1 |
| PPP2R4 | 1 |
| PPP2R5B | 1 |
| PPP2R5E | 1 |
| PPP3CA | 1 |
| PPP4C | 1 |
| PPP4R1 | 1 |
| PPP5C | 1 |
| PPP6C | 1 |
| PQBP1 | 1 |
| PQLC1 | 1 |
| PRAF2 | 1 |
| PRAMEF14 | 1 |
| PRAMENP | 1 |
| PRB4 | 1 |
| PRC1 | 1 |
| PRCP | 1 |
| PRDM1 | 1 |
| PRDM13 | 1 |
| PRDM14 | 1 |
| PRDM16 | 1 |
| PRDM2 | 1 |
| PRDM4 | 1 |
| PRDM8 | 1 |
| PRDM9 | 1 |
| PRDX1 | 1 |
| PRDX3 | 1 |
| PRDX5 | 1 |
| PREP | 1 |
| PREPL | 1 |
| PREX2 | 1 |
| PRF1 | 1 |
| PRG2 | 1 |
| PRG4 | 1 |
| PRH2 | 1 |
| PRKAA1 | 1 |
| PRKACG | 1 |
| PRKAG1 | 1 |
| PRKAR1A | 1 |
| PRKAR1B | 1 |
| PRKAR2A | 1 |
| PRKCA | 1 |
| PRKCB | 1 |
| PRKCDBP | 1 |
| PRKCE | 1 |
| PRKCH | 1 |
| PRKCSH | 1 |
| PRKD1 | 1 |
| PRKD3 | 1 |
| PRKRA | 1 |
| PRKRIP1 | 1 |
| PRKX | 1 |
| PRLR | 1 |
| PRNP | 1 |
| PRNT | 1 |
| PRO2012 | 1 |
| PROCR | 1 |
| PRODH2 | 1 |
| PRORSD1P | 1 |
| PRORY | 1 |
| PRPF19 | 1 |
| PRPF3 | 1 |
| PRPF31 | 1 |
| PRPF38B | 1 |
| PRPF39 | 1 |
| PRPF4 | 1 |
| PRPF40A | 1 |
| PRPF6 | 1 |
| PRPF8 | 1 |
| PRPS1 | 1 |
| PRPS1L1 | 1 |
| PRR11 | 1 |
| PRR12 | 1 |
| PRR14 | 1 |
| PRR14L | 1 |
| PRR15L | 1 |
| PRR16 | 1 |
| PRR21 | 1 |
| PRR23A | 1 |
| PRR26 | 1 |
| PRR27 | 1 |
| PRR30 | 1 |
| PRR4 | 1 |
| PRR5L | 1 |
| PRRT3 | 1 |
| PRRX2 | 1 |
| PRSS1 | 1 |
| PRSS12 | 1 |
| PRSS23 | 1 |
| PRSS35 | 1 |
| PRSS37 | 1 |
| PRTG | 1 |
| PRUNE | 1 |
| PSAT1 | 1 |
| PSEN1 | 1 |
| PSENEN | 1 |
| PSG1 | 1 |
| PSG2 | 1 |
| PSG4 | 1 |
| PSG5 | 1 |
| PSG7 | 1 |
| PSMA1 | 1 |
| PSMA4 | 1 |
| PSMA5 | 1 |
| PSMB1 | 1 |
| PSMB2 | 1 |
| PSMB3 | 1 |
| PSMB5 | 1 |
| PSMB6 | 1 |
| PSMB7 | 1 |
| PSMC2 | 1 |
| PSMC3 | 1 |
| PSMC3IP | 1 |
| PSMC4 | 1 |
| PSMC5 | 1 |
| PSMD1 | 1 |
| PSMD10 | 1 |
| PSMD12 | 1 |
| PSMD13 | 1 |
| PSMD14 | 1 |
| PSMD2 | 1 |
| PSMD5 | 1 |
| PSMD6 | 1 |
| PSMD7 | 1 |
| PSMD8 | 1 |
| PSMD9 | 1 |
| PSME3 | 1 |
| PSME4 | 1 |
| PSMF1 | 1 |
| PSMG1 | 1 |
| PSMG2 | 1 |
| PSORS1C3 | 1 |
| PSPC1 | 1 |
| PSRC1 | 1 |
| PSTPIP2 | 1 |
| PTAFR | 1 |
| PTBP2 | 1 |
| PTCH1 | 1 |
| PTCH2 | 1 |
| PTCRA | 1 |
| PTDSS1 | 1 |
| PTF1A | 1 |
| PTGDR | 1 |
| PTGDR2 | 1 |
| PTGDS | 1 |
| PTGER2 | 1 |
| PTGES2 | 1 |
| PTGIS | 1 |
| PTGR1 | 1 |
| PTGS1 | 1 |
| PTH1R | 1 |
| PTH2 | 1 |
| PTH2R | 1 |
| PTK2B | 1 |
| PTOV1 | 1 |
| PTP4A1 | 1 |
| PTP4A2 | 1 |
| PTP4A3 | 1 |
| PTPDC1 | 1 |
| PTPLA | 1 |
| PTPLAD1 | 1 |
| PTPMT1 | 1 |
| PTPN11 | 1 |
| PTPN12 | 1 |
| PTPN13 | 1 |
| PTPN18 | 1 |
| PTPN21 | 1 |
| PTPN22 | 1 |
| PTPN3 | 1 |
| PTPN4 | 1 |
| PTPN6 | 1 |
| PTPRA | 1 |
| PTPRB | 1 |
| PTPRC | 1 |
| PTPRCAP | 1 |
| PTPRD | 1 |
| PTPRE | 1 |
| PTPRF | 1 |
| PTPRK | 1 |
| PTPRO | 1 |
| PTPRR | 1 |
| PTPRU | 1 |
| PTPRZ1 | 1 |
| PTRF | 1 |
| PTRH1 | 1 |
| PTRH2 | 1 |
| PTTG1IP | 1 |
| PTTG2 | 1 |
| PTX3 | 1 |
| PUF60 | 1 |
| PURA | 1 |
| PURG | 1 |
| PUS10 | 1 |
| PUS7L | 1 |
| PUSL1 | 1 |
| PVR | 1 |
| PVRIG | 1 |
| PVRL3 | 1 |
| PWP1 | 1 |
| PWWP2B | 1 |
| PXDC1 | 1 |
| PXDN | 1 |
| PXDNL | 1 |
| PXN | 1 |
| PXYLP1 | 1 |
| PYCARD | 1 |
| PYCRL | 1 |
| PYGB | 1 |
| PYHIN1 | 1 |
| PYROXD1 | 1 |
| PYY2 | 1 |
| PZP | 1 |
| QDPR | 1 |
| QRICH1 | 1 |
| QRSL1 | 1 |
| QSER1 | 1 |
| QSOX1 | 1 |
| RAB10 | 1 |
| RAB11A | 1 |
| RAB11B | 1 |
| RAB11FIP1 | 1 |
| RAB11FIP5 | 1 |
| RAB13 | 1 |
| RAB14 | 1 |
| RAB18 | 1 |
| RAB1A | 1 |
| RAB1B | 1 |
| RAB21 | 1 |
| RAB23 | 1 |
| RAB2A | 1 |
| RAB30 | 1 |
| RAB32 | 1 |
| RAB34 | 1 |
| RAB35 | 1 |
| RAB39A | 1 |
| RAB3B | 1 |
| RAB3GAP1 | 1 |
| RAB44 | 1 |
| RAB5A | 1 |
| RAB5B | 1 |
| RAB5C | 1 |
| RAB6A | 1 |
| RAB7A | 1 |
| RAB8A | 1 |
| RAB8B | 1 |
| RABAC1 | 1 |
| RABEP1 | 1 |
| RABEP2 | 1 |
| RABEPK | 1 |
| RABGAP1 | 1 |
| RABGEF1 | 1 |
| RABGGTB | 1 |
| RABIF | 1 |
| RABL6 | 1 |
| RAC1 | 1 |
| RACGAP1 | 1 |
| RACGAP1P | 1 |
| RAD21 | 1 |
| RAD23B | 1 |
| RAD51 | 1 |
| RAD51C | 1 |
| RAD51D | 1 |
| RAD9A | 1 |
| RAET1E | 1 |
| RAF1 | 1 |
| RAG1 | 1 |
| RAG2 | 1 |
| RAI1 | 1 |
| RALA | 1 |
| RALB | 1 |
| RALBP1 | 1 |
| RALGAPA2 | 1 |
| RALGDS | 1 |
| RALGPS2 | 1 |
| RALY | 1 |
| RALYL | 1 |
| RAMP2.AS1 | 1 |
| RAMP2 | 1 |
| RAN | 1 |
| RANBP1 | 1 |
| RANBP17 | 1 |
| RANBP3 | 1 |
| RANBP3L | 1 |
| RANGAP1 | 1 |
| RAP1B | 1 |
| RAP1GAP2 | 1 |
| RAP1GDS1 | 1 |
| RAPGEF2 | 1 |
| RAPGEF4 | 1 |
| RAPGEF5 | 1 |
| RAPGEFL1 | 1 |
| RARB | 1 |
| RARG | 1 |
| RARRES1 | 1 |
| RARS | 1 |
| RARS2 | 1 |
| RASA1 | 1 |
| RASA3 | 1 |
| RASAL3 | 1 |
| RASGEF1B | 1 |
| RASGRF2 | 1 |
| RASGRP1 | 1 |
| RASGRP2 | 1 |
| RASGRP3 | 1 |
| RASGRP4 | 1 |
| RASIP1 | 1 |
| RASL11A | 1 |
| RASL12 | 1 |
| RASSF2 | 1 |
| RASSF4 | 1 |
| RASSF5 | 1 |
| RBBP6 | 1 |
| RBFOX2 | 1 |
| RBFOX3 | 1 |
| RBKS | 1 |
| RBM11 | 1 |
| RBM12 | 1 |
| RBM14 | 1 |
| RBM15 | 1 |
| RBM15B | 1 |
| RBM33 | 1 |
| RBM42 | 1 |
| RBM44 | 1 |
| RBM47 | 1 |
| RBM7 | 1 |
| RBMS2 | 1 |
| RBP2 | 1 |
| RBP3 | 1 |
| RBP7 | 1 |
| RBPJ | 1 |
| RBPMS | 1 |
| RBX1 | 1 |
| RCC2 | 1 |
| RCN2 | 1 |
| RCN3 | 1 |
| RCOR2 | 1 |
| RCSD1 | 1 |
| RDH11 | 1 |
| RDH14 | 1 |
| RDH5 | 1 |
| RDM1 | 1 |
| RDX | 1 |
| REC8 | 1 |
| RECK | 1 |
| RECQL | 1 |
| RECQL5 | 1 |
| REEP2 | 1 |
| REEP5 | 1 |
| REG3G | 1 |
| REG4 | 1 |
| REL | 1 |
| RELA | 1 |
| REM2 | 1 |
| REPS2 | 1 |
| RER1 | 1 |
| RERE | 1 |
| RERG | 1 |
| RERGL | 1 |
| REST | 1 |
| RETSAT | 1 |
| REV1 | 1 |
| REV3L | 1 |
| REXO1L1P | 1 |
| REXO2 | 1 |
| RFC2 | 1 |
| RFC3 | 1 |
| RFESD | 1 |
| RFFL | 1 |
| RFK | 1 |
| RFPL1 | 1 |
| RFT1 | 1 |
| RFTN1 | 1 |
| RFWD3 | 1 |
| RFXANK | 1 |
| RFXAP | 1 |
| RGAG4 | 1 |
| RGCC | 1 |
| RGL4 | 1 |
| RGMB | 1 |
| RGS1 | 1 |
| RGS13 | 1 |
| RGS14 | 1 |
| RGS18 | 1 |
| RGS2 | 1 |
| RGS20 | 1 |
| RGS22 | 1 |
| RGS4 | 1 |
| RGS5 | 1 |
| RGS7 | 1 |
| RGS7BP | 1 |
| RHBDD1 | 1 |
| RHBDD2 | 1 |
| RHBDF1 | 1 |
| RHBDF2 | 1 |
| RHCE | 1 |
| RHEB | 1 |
| RHNO1 | 1 |
| RHOA | 1 |
| RHOB | 1 |
| RHOBTB2 | 1 |
| RHOC | 1 |
| RHOG | 1 |
| RHOH | 1 |
| RHOJ | 1 |
| RHOXF1 | 1 |
| RIBC1 | 1 |
| RIC8B | 1 |
| RIMS2 | 1 |
| RIN1 | 1 |
| RINL | 1 |
| RIOK1 | 1 |
| RIOK2 | 1 |
| RIOK3 | 1 |
| RIPK1 | 1 |
| RIPK3 | 1 |
| RIPPLY2 | 1 |
| RIT1 | 1 |
| RITA1 | 1 |
| RLF | 1 |
| RLIM | 1 |
| RMDN1 | 1 |
| RMDN2 | 1 |
| RMDN3 | 1 |
| RMI2 | 1 |
| RMND1 | 1 |
| RMND5A | 1 |
| RMRP | 1 |
| RNASE1 | 1 |
| RNASE10 | 1 |
| RNASE12 | 1 |
| RNASE13 | 1 |
| RNASE2 | 1 |
| RNASE6 | 1 |
| RNASE7 | 1 |
| RNASEK.C17orf49 | 1 |
| RNASEK | 1 |
| RND3 | 1 |
| RNF103 | 1 |
| RNF11 | 1 |
| RNF113B | 1 |
| RNF115 | 1 |
| RNF122 | 1 |
| RNF125 | 1 |
| RNF126 | 1 |
| RNF14 | 1 |
| RNF144A | 1 |
| RNF148 | 1 |
| RNF149 | 1 |
| RNF167 | 1 |
| RNF170 | 1 |
| RNF175 | 1 |
| RNF180 | 1 |
| RNF181 | 1 |
| RNF185 | 1 |
| RNF19A | 1 |
| RNF216 | 1 |
| RNF24 | 1 |
| RNF25 | 1 |
| RNF26 | 1 |
| RNF4 | 1 |
| RNF40 | 1 |
| RNF41 | 1 |
| RNF6 | 1 |
| RNH1 | 1 |
| RNMTL1 | 1 |
| RNPC3 | 1 |
| RNPEP | 1 |
| RNU12 | 1 |
| RNU2.1 | 1 |
| RNU4.1 | 1 |
| RNU4.2 | 1 |
| RNU5D.1 | 1 |
| RNVU1.3 | 1 |
| ROBO2 | 1 |
| ROBO4 | 1 |
| ROCK1 | 1 |
| ROCK1P1 | 1 |
| ROMO1 | 1 |
| ROR2 | 1 |
| RORB | 1 |
| RP1L1 | 1 |
| RP9 | 1 |
| RPA1 | 1 |
| RPA3 | 1 |
| RPA4 | 1 |
| RPAP2 | 1 |
| RPE | 1 |
| RPEL1 | 1 |
| RPF2 | 1 |
| RPGRIP1 | 1 |
| RPL10 | 1 |
| RPL10A | 1 |
| RPL12 | 1 |
| RPL13AP20 | 1 |
| RPL13AP3 | 1 |
| RPL13AP5 | 1 |
| RPL13P5 | 1 |
| RPL14 | 1 |
| RPL15 | 1 |
| RPL17 | 1 |
| RPL18 | 1 |
| RPL19 | 1 |
| RPL21P44 | 1 |
| RPL22L1 | 1 |
| RPL23 | 1 |
| RPL23AP64 | 1 |
| RPL26 | 1 |
| RPL26L1 | 1 |
| RPL27 | 1 |
| RPL27A | 1 |
| RPL3 | 1 |
| RPL31 | 1 |
| RPL32P3 | 1 |
| RPL35 | 1 |
| RPL36 | 1 |
| RPL36AL | 1 |
| RPL38 | 1 |
| RPL39L | 1 |
| RPL3L | 1 |
| RPL4 | 1 |
| RPL5 | 1 |
| RPL8 | 1 |
| RPLP0 | 1 |
| RPN2 | 1 |
| RPP25L | 1 |
| RPP30 | 1 |
| RPP40 | 1 |
| RPS10P7 | 1 |
| RPS11 | 1 |
| RPS14P3 | 1 |
| RPS16 | 1 |
| RPS19 | 1 |
| RPS19BP1 | 1 |
| RPS23 | 1 |
| RPS25 | 1 |
| RPS27L | 1 |
| RPS29 | 1 |
| RPS2P45 | 1 |
| RPS3 | 1 |
| RPS3A | 1 |
| RPS4X | 1 |
| RPS4Y1 | 1 |
| RPS6 | 1 |
| RPS6KA1 | 1 |
| RPS6KA2 | 1 |
| RPS6KA4 | 1 |
| RPS6KA5 | 1 |
| RPS6KB2 | 1 |
| RPS6P6 | 1 |
| RPSAP52 | 1 |
| RPSAP58 | 1 |
| RPUSD1 | 1 |
| RPUSD2 | 1 |
| RPUSD3 | 1 |
| RQCD1 | 1 |
| RRAGA | 1 |
| RRAGC | 1 |
| RRAS | 1 |
| RRAS2 | 1 |
| RRM1 | 1 |
| RRM2 | 1 |
| RRM2B | 1 |
| RRNAD1 | 1 |
| RRP1 | 1 |
| RRP15 | 1 |
| RRP36 | 1 |
| RRP7A | 1 |
| RRP9 | 1 |
| RRS1 | 1 |
| RSAD2 | 1 |
| RSC1A1 | 1 |
| RSL1D1 | 1 |
| RSL24D1 | 1 |
| RSPH1 | 1 |
| RSPRY1 | 1 |
| RSU1 | 1 |
| RTCB | 1 |
| RTF1 | 1 |
| RTFDC1 | 1 |
| RTKN | 1 |
| RTN4 | 1 |
| RTN4IP1 | 1 |
| RTP1 | 1 |
| RUFY1 | 1 |
| RUFY4 | 1 |
| RUNDC3B | 1 |
| RUNX1T1 | 1 |
| RUNX2 | 1 |
| RUNX3 | 1 |
| RUSC1.AS1 | 1 |
| RUSC2 | 1 |
| RUVBL1 | 1 |
| RUVBL2 | 1 |
| RWDD1 | 1 |
| RWDD2B | 1 |
| RXRA | 1 |
| RYR2 | 1 |
| S100A10 | 1 |
| S100A11 | 1 |
| S100A14 | 1 |
| S100A16 | 1 |
| S100A2 | 1 |
| S100A3 | 1 |
| S100A4 | 1 |
| S100A6 | 1 |
| S100A7 | 1 |
| S100A8 | 1 |
| S100B | 1 |
| S100G | 1 |
| S1PR1 | 1 |
| S1PR4 | 1 |
| SAA1 | 1 |
| SAC3D1 | 1 |
| SACS | 1 |
| SAE1 | 1 |
| SAFB | 1 |
| SAG | 1 |
| SALL1 | 1 |
| SAMD3 | 1 |
| SAMM50 | 1 |
| SAMSN1 | 1 |
| SAP18 | 1 |
| SAP30BP | 1 |
| SAR1B | 1 |
| SARAF | 1 |
| SARS | 1 |
| SARS2 | 1 |
| SART1 | 1 |
| SART3 | 1 |
| SASH3 | 1 |
| SASS6 | 1 |
| SAT1 | 1 |
| SATB1 | 1 |
| SATB2.AS1 | 1 |
| SATB2 | 1 |
| SAV1 | 1 |
| SBDS | 1 |
| SBF1 | 1 |
| SBNO1 | 1 |
| SBSN | 1 |
| SCAMP2 | 1 |
| SCAMP3 | 1 |
| SCAMP5 | 1 |
| SCAND1 | 1 |
| SCAND2P | 1 |
| SCAP | 1 |
| SCAPER | 1 |
| SCARA3 | 1 |
| SCARB1 | 1 |
| SCARB2 | 1 |
| SCARF1 | 1 |
| SCARNA10 | 1 |
| SCARNA12 | 1 |
| SCARNA13 | 1 |
| SCARNA23 | 1 |
| SCARNA4 | 1 |
| SCARNA8 | 1 |
| SCART1 | 1 |
| SCCPDH | 1 |
| SCFD2 | 1 |
| SCGB2A2 | 1 |
| SCGB2B2 | 1 |
| SCGB3A2 | 1 |
| SCGN | 1 |
| SCIMP | 1 |
| SCML1 | 1 |
| SCN1A | 1 |
| SCN2A | 1 |
| SCN3A | 1 |
| SCN5A | 1 |
| SCNN1B | 1 |
| SCOC.AS1 | 1 |
| SCOC | 1 |
| SCP2 | 1 |
| SCRN1 | 1 |
| SCRN3 | 1 |
| SCUBE1 | 1 |
| SCUBE3 | 1 |
| SCYL1 | 1 |
| SCYL2 | 1 |
| SDC4 | 1 |
| SDCBP | 1 |
| SDCCAG3 | 1 |
| SDF2 | 1 |
| SDF2L1 | 1 |
| SDF4 | 1 |
| SDHA | 1 |
| SDHAF2 | 1 |
| SDHB | 1 |
| SDHD | 1 |
| SDK2 | 1 |
| SDR42E1 | 1 |
| SDR9C7 | 1 |
| SDSL | 1 |
| SEBOX | 1 |
| SEC13 | 1 |
| SEC14L5 | 1 |
| SEC16A | 1 |
| SEC22C | 1 |
| SEC23A | 1 |
| SEC23IP | 1 |
| SEC24D | 1 |
| SEC31A | 1 |
| SEC31B | 1 |
| SEC61A1 | 1 |
| SEC61B | 1 |
| SEC62 | 1 |
| SEC63 | 1 |
| SECISBP2 | 1 |
| SECISBP2L | 1 |
| SECTM1 | 1 |
| SEL1L | 1 |
| SEL1L2 | 1 |
| SEL1L3 | 1 |
| SELK | 1 |
| SELL | 1 |
| SELM | 1 |
| SELP | 1 |
| SELT | 1 |
| SELV | 1 |
| SEMA3C | 1 |
| SEMA4A | 1 |
| SEMA4C | 1 |
| SEMA4D | 1 |
| SEMA4F | 1 |
| SEMA6A | 1 |
| SEMA6B | 1 |
| SEMA6C | 1 |
| SEMA6D | 1 |
| SEMA7A | 1 |
| SEMG2 | 1 |
| SENP3 | 1 |
| SENP7 | 1 |
| 15-Sep | 1 |
| SEPHS2 | 1 |
| 10-Sep | 1 |
| 11-Sep | 1 |
| 14-Sep | 1 |
| 2-Sep | 1 |
| 4-Sep | 1 |
| 9-Sep | 1 |
| SEPW1 | 1 |
| SERAC1 | 1 |
| SERINC1 | 1 |
| SERINC3 | 1 |
| SERINC4 | 1 |
| SERP2 | 1 |
| SERPINA12 | 1 |
| SERPINB1 | 1 |
| SERPINB2 | 1 |
| SERPINB4 | 1 |
| SERPINB6 | 1 |
| SERPINB7 | 1 |
| SERPINB8 | 1 |
| SERPINB9P1 | 1 |
| SERPINE1 | 1 |
| SERPINE2 | 1 |
| SERPING1 | 1 |
| SERPINI2 | 1 |
| SERTAD1 | 1 |
| SESTD1 | 1 |
| SET | 1 |
| SETBP1 | 1 |
| SETD1B | 1 |
| SETD4 | 1 |
| SETD5 | 1 |
| SETD7 | 1 |
| SETDB1 | 1 |
| SETMAR | 1 |
| SF1 | 1 |
| SF3A1 | 1 |
| SF3B2 | 1 |
| SF3B3 | 1 |
| SF3B5 | 1 |
| SF3B6 | 1 |
| SFMBT2 | 1 |
| SFN | 1 |
| SFRP2 | 1 |
| SFSWAP | 1 |
| SFT2D1 | 1 |
| SFT2D3 | 1 |
| SFXN1 | 1 |
| SFXN2 | 1 |
| SFXN3 | 1 |
| SFXN4 | 1 |
| SGCB | 1 |
| SGCG | 1 |
| SGK1 | 1 |
| SGK223 | 1 |
| SGK494 | 1 |
| SGSH | 1 |
| SGTB | 1 |
| SH2B1 | 1 |
| SH2B2 | 1 |
| SH2B3 | 1 |
| SH2D1A | 1 |
| SH2D1B | 1 |
| SH2D2A | 1 |
| SH2D3C | 1 |
| SH2D4A | 1 |
| SH3BP4 | 1 |
| SH3BP5L | 1 |
| SH3D19 | 1 |
| SH3D21 | 1 |
| SH3GL1 | 1 |
| SH3GL3 | 1 |
| SH3GLB1 | 1 |
| SH3PXD2A | 1 |
| SH3PXD2B | 1 |
| SH3RF1 | 1 |
| SH3RF2 | 1 |
| SH3TC2 | 1 |
| SHANK2.AS3 | 1 |
| SHANK2 | 1 |
| SHANK3 | 1 |
| SHC3 | 1 |
| SHCBP1 | 1 |
| SHE | 1 |
| SHF | 1 |
| SHFM1 | 1 |
| SHISA2 | 1 |
| SHISA5 | 1 |
| SHKBP1 | 1 |
| SHMT2 | 1 |
| SHOC2 | 1 |
| SHOX2 | 1 |
| SHPRH | 1 |
| SHROOM1 | 1 |
| SHROOM4 | 1 |
| SIAE | 1 |
| SIAH1 | 1 |
| SIDT1 | 1 |
| SIGLEC17P | 1 |
| SIGLECL1 | 1 |
| SIK1 | 1 |
| SIK3 | 1 |
| SIPA1L1 | 1 |
| SIRPB2 | 1 |
| SIRPG | 1 |
| SIRT1 | 1 |
| SIRT5 | 1 |
| SIRT7 | 1 |
| SIX1 | 1 |
| SIX4 | 1 |
| SKA2 | 1 |
| SKA3 | 1 |
| SKAP1 | 1 |
| SKOR1 | 1 |
| SLA | 1 |
| SLAMF1 | 1 |
| SLAMF6 | 1 |
| SLAMF7 | 1 |
| SLAMF8 | 1 |
| SLBP | 1 |
| SLC10A5 | 1 |
| SLC10A7 | 1 |
| SLC12A4 | 1 |
| SLC12A6 | 1 |
| SLC12A7 | 1 |
| SLC13A1 | 1 |
| SLC13A2 | 1 |
| SLC14A1 | 1 |
| SLC15A2 | 1 |
| SLC15A5 | 1 |
| SLC16A10 | 1 |
| SLC16A2 | 1 |
| SLC16A4 | 1 |
| SLC16A6 | 1 |
| SLC17A2 | 1 |
| SLC17A6 | 1 |
| SLC19A2 | 1 |
| SLC19A3 | 1 |
| SLC1A1 | 1 |
| SLC20A1 | 1 |
| SLC22A10 | 1 |
| SLC22A15 | 1 |
| SLC22A2 | 1 |
| SLC22A3 | 1 |
| SLC22A5 | 1 |
| SLC22A9 | 1 |
| SLC23A3 | 1 |
| SLC25A1 | 1 |
| SLC25A11 | 1 |
| SLC25A14 | 1 |
| SLC25A22 | 1 |
| SLC25A25 | 1 |
| SLC25A26 | 1 |
| SLC25A27 | 1 |
| SLC25A3 | 1 |
| SLC25A30 | 1 |
| SLC25A31 | 1 |
| SLC25A37 | 1 |
| SLC25A38 | 1 |
| SLC25A39 | 1 |
| SLC25A4 | 1 |
| SLC25A46 | 1 |
| SLC25A48 | 1 |
| SLC25A51 | 1 |
| SLC26A4 | 1 |
| SLC26A7 | 1 |
| SLC27A2 | 1 |
| SLC27A4 | 1 |
| SLC2A11 | 1 |
| SLC2A12 | 1 |
| SLC2A14 | 1 |
| SLC2A2 | 1 |
| SLC30A1 | 1 |
| SLC30A4 | 1 |
| SLC30A9 | 1 |
| SLC31A1 | 1 |
| SLC31A2 | 1 |
| SLC34A1 | 1 |
| SLC35A1 | 1 |
| SLC35A4 | 1 |
| SLC35A5 | 1 |
| SLC35B3 | 1 |
| SLC35B4 | 1 |
| SLC35C1 | 1 |
| SLC35D3 | 1 |
| SLC35E1 | 1 |
| SLC35F1 | 1 |
| SLC35F5 | 1 |
| SLC36A2 | 1 |
| SLC37A1 | 1 |
| SLC37A2 | 1 |
| SLC38A1 | 1 |
| SLC38A2 | 1 |
| SLC38A3 | 1 |
| SLC38A5 | 1 |
| SLC38A7 | 1 |
| SLC39A1 | 1 |
| SLC39A10 | 1 |
| SLC39A13 | 1 |
| SLC39A3 | 1 |
| SLC39A7 | 1 |
| SLC39A8 | 1 |
| SLC3A2 | 1 |
| SLC40A1 | 1 |
| SLC41A3 | 1 |
| SLC43A3 | 1 |
| SLC44A3 | 1 |
| SLC44A4 | 1 |
| SLC44A5 | 1 |
| SLC45A2 | 1 |
| SLC45A4 | 1 |
| SLC46A1 | 1 |
| SLC48A1 | 1 |
| SLC4A10 | 1 |
| SLC4A11 | 1 |
| SLC4A1AP | 1 |
| SLC4A5 | 1 |
| SLC4A7 | 1 |
| SLC4A9 | 1 |
| SLC50A1 | 1 |
| SLC51A | 1 |
| SLC5A3 | 1 |
| SLC5A4 | 1 |
| SLC5A6 | 1 |
| SLC5A8 | 1 |
| SLC6A16 | 1 |
| SLC6A2 | 1 |
| SLC6A6 | 1 |
| SLC7A11 | 1 |
| SLC7A2 | 1 |
| SLC7A3 | 1 |
| SLC7A5 | 1 |
| SLC7A7 | 1 |
| SLC8A3 | 1 |
| SLC9A2 | 1 |
| SLC9A3R1 | 1 |
| SLC9A6 | 1 |
| SLC9A7 | 1 |
| SLC9A9 | 1 |
| SLC9C1 | 1 |
| SLC9C2 | 1 |
| SLCO1B7 | 1 |
| SLCO1C1 | 1 |
| SLCO2A1 | 1 |
| SLCO2B1 | 1 |
| SLCO4C1 | 1 |
| SLFN13 | 1 |
| SLIRP | 1 |
| SLITRK5 | 1 |
| SLN | 1 |
| SMAD1 | 1 |
| SMAD2 | 1 |
| SMAD3 | 1 |
| SMAGP | 1 |
| SMARCA2 | 1 |
| SMARCAL1 | 1 |
| SMC1A | 1 |
| SMC4 | 1 |
| SMC5 | 1 |
| SMCO4 | 1 |
| SMDT1 | 1 |
| SMG8 | 1 |
| SMIM14 | 1 |
| SMIM15 | 1 |
| SMIM2 | 1 |
| SMIM20 | 1 |
| SMIM21 | 1 |
| SMIM3 | 1 |
| SMIM4 | 1 |
| SMIM5 | 1 |
| SMIM7 | 1 |
| SMO | 1 |
| SMPD1 | 1 |
| SMPD3 | 1 |
| SMTN | 1 |
| SMURF2 | 1 |
| SMYD1 | 1 |
| SMYD3 | 1 |
| SMYD5 | 1 |
| SNAI2 | 1 |
| SNAP23 | 1 |
| SNAPC2 | 1 |
| SNAPC3 | 1 |
| SNCA | 1 |
| SNCAIP | 1 |
| SND1.IT1 | 1 |
| SNED1 | 1 |
| SNF8 | 1 |
| SNHG11 | 1 |
| SNORA13 | 1 |
| SNORA14A | 1 |
| SNORA15 | 1 |
| SNORA16B | 1 |
| SNORA19 | 1 |
| SNORA20 | 1 |
| SNORA22 | 1 |
| SNORA24 | 1 |
| SNORA2A | 1 |
| SNORA2B | 1 |
| SNORA35 | 1 |
| SNORA36C | 1 |
| SNORA38B | 1 |
| SNORA4 | 1 |
| SNORA45A | 1 |
| SNORA45B | 1 |
| SNORA46 | 1 |
| SNORA48 | 1 |
| SNORA49 | 1 |
| SNORA54 | 1 |
| SNORA55 | 1 |
| SNORA58 | 1 |
| SNORA59B | 1 |
| SNORA5A | 1 |
| SNORA5B | 1 |
| SNORA5C | 1 |
| SNORA60 | 1 |
| SNORA65 | 1 |
| SNORA70 | 1 |
| SNORA70B | 1 |
| SNORA70D | 1 |
| SNORA70G | 1 |
| SNORA71B | 1 |
| SNORA71C | 1 |
| SNORA71D | 1 |
| SNORA75 | 1 |
| SNORA76A | 1 |
| SNORA80E | 1 |
| SNORD103A | 1 |
| SNORD105 | 1 |
| SNORD113.3 | 1 |
| SNORD113.4 | 1 |
| SNORD114.2 | 1 |
| SNORD114.3 | 1 |
| SNORD115.24 | 1 |
| SNORD115.28 | 1 |
| SNORD115.8 | 1 |
| SNORD116.25 | 1 |
| SNORD116.26 | 1 |
| SNORD116.27 | 1 |
| SNORD117 | 1 |
| SNORD13P1 | 1 |
| SNORD13P2 | 1 |
| SNORD15A | 1 |
| SNORD15B | 1 |
| SNORD20 | 1 |
| SNORD21 | 1 |
| SNORD32A | 1 |
| SNORD32B | 1 |
| SNORD33 | 1 |
| SNORD34 | 1 |
| SNORD45A | 1 |
| SNORD46 | 1 |
| SNORD50A | 1 |
| SNORD53 | 1 |
| SNORD56B | 1 |
| SNORD59A | 1 |
| SNORD62A | 1 |
| SNORD8 | 1 |
| SNORD82 | 1 |
| SNORD94 | 1 |
| SNORD96B | 1 |
| SNPH | 1 |
| SNRK | 1 |
| SNRNP25 | 1 |
| SNRNP27 | 1 |
| SNRNP35 | 1 |
| SNRPA1 | 1 |
| SNRPC | 1 |
| SNRPD1 | 1 |
| SNRPD3 | 1 |
| SNRPF | 1 |
| SNRPN | 1 |
| SNTB2 | 1 |
| SNTG1 | 1 |
| SNUPN | 1 |
| SNX1 | 1 |
| SNX10 | 1 |
| SNX11 | 1 |
| SNX12 | 1 |
| SNX14 | 1 |
| SNX15 | 1 |
| SNX16 | 1 |
| SNX3 | 1 |
| SNX32 | 1 |
| SNX33 | 1 |
| SNX6 | 1 |
| SNX9 | 1 |
| SOAT1 | 1 |
| SOCS5 | 1 |
| SOCS6 | 1 |
| SOD1 | 1 |
| SOD2 | 1 |
| SORBS1 | 1 |
| SORCS1 | 1 |
| SORD | 1 |
| SOWAHB | 1 |
| SOX1 | 1 |
| SOX13 | 1 |
| SOX18 | 1 |
| SOX30 | 1 |
| SOX6 | 1 |
| SP3P | 1 |
| SP7 | 1 |
| SPA17 | 1 |
| SPACA1 | 1 |
| SPAG11A | 1 |
| SPAG16 | 1 |
| SPAG5 | 1 |
| SPAG6 | 1 |
| SPAG8 | 1 |
| SPANXA2.OT1 | 1 |
| SPANXN5 | 1 |
| SPARCL1 | 1 |
| SPATA1 | 1 |
| SPATA17 | 1 |
| SPATA18 | 1 |
| SPATA20 | 1 |
| SPATA24 | 1 |
| SPATA25 | 1 |
| SPATA31D1 | 1 |
| SPATA31D4 | 1 |
| SPATA9 | 1 |
| SPC24 | 1 |
| SPC25 | 1 |
| SPCS1 | 1 |
| SPCS3 | 1 |
| SPDYA | 1 |
| SPDYC | 1 |
| SPDYE3 | 1 |
| SPECC1 | 1 |
| SPEF2 | 1 |
| SPEM1 | 1 |
| SPEN | 1 |
| SPERT | 1 |
| SPG20 | 1 |
| SPI1 | 1 |
| SPIB | 1 |
| SPIDR | 1 |
| SPIN1 | 1 |
| SPIN3 | 1 |
| SPINK2 | 1 |
| SPINK9 | 1 |
| SPNS1 | 1 |
| SPNS2 | 1 |
| SPOCD1 | 1 |
| SPOCK1 | 1 |
| SPOCK3 | 1 |
| SPON1 | 1 |
| SPOP | 1 |
| SPP1 | 1 |
| SPP2 | 1 |
| SPR | 1 |
| SPRED2 | 1 |
| SPRR1B | 1 |
| SPRY1 | 1 |
| SPRY2 | 1 |
| SPRYD3 | 1 |
| SPRYD7 | 1 |
| SPSB3 | 1 |
| SPTAN1 | 1 |
| SPTBN1 | 1 |
| SPTLC2 | 1 |
| SPTLC3 | 1 |
| SPTSSB | 1 |
| SPTY2D1 | 1 |
| SQRDL | 1 |
| SQSTM1 | 1 |
| SRC | 1 |
| SRD5A1 | 1 |
| SREBF2 | 1 |
| SRGAP3 | 1 |
| SRGN | 1 |
| SRI | 1 |
| SRP14 | 1 |
| SRP19 | 1 |
| SRP68 | 1 |
| SRPR | 1 |
| SRR | 1 |
| SRRM1 | 1 |
| SRSF1 | 1 |
| SRSF11 | 1 |
| SRSF12 | 1 |
| SRSF3 | 1 |
| SRSF5 | 1 |
| SRSF6 | 1 |
| SRSF9 | 1 |
| SRXN1 | 1 |
| SS18L1 | 1 |
| SS18L2 | 1 |
| SSB | 1 |
| SSBP1 | 1 |
| SSBP2 | 1 |
| SSBP4 | 1 |
| SSFA2 | 1 |
| SSH1 | 1 |
| SSH2 | 1 |
| SSNA1 | 1 |
| SSR1 | 1 |
| SSR3 | 1 |
| SSSCA1 | 1 |
| SSTR1 | 1 |
| SSTR4 | 1 |
| SSX2IP | 1 |
| ST13P4 | 1 |
| ST18 | 1 |
| ST3GAL6 | 1 |
| ST6GALNAC3 | 1 |
| ST6GALNAC4 | 1 |
| ST8SIA4 | 1 |
| ST8SIA5 | 1 |
| ST8SIA6 | 1 |
| STAB1 | 1 |
| STAC | 1 |
| STAC3 | 1 |
| STAG3 | 1 |
| STAM | 1 |
| STAM2 | 1 |
| STAMBPL1 | 1 |
| STAP1 | 1 |
| STAP2 | 1 |
| STARD7 | 1 |
| STARD9 | 1 |
| STAT1 | 1 |
| STAT4 | 1 |
| STBD1 | 1 |
| STC1 | 1 |
| STC2 | 1 |
| STEAP3 | 1 |
| STEAP4 | 1 |
| STIP1 | 1 |
| STK11 | 1 |
| STK16 | 1 |
| STK17A | 1 |
| STK17B | 1 |
| STK25 | 1 |
| STK3 | 1 |
| STK31 | 1 |
| STK33 | 1 |
| STK39 | 1 |
| STK4 | 1 |
| STMN2 | 1 |
| STMN4 | 1 |
| STOM | 1 |
| STOML1 | 1 |
| STOML2 | 1 |
| STON1 | 1 |
| STON2 | 1 |
| STOX1 | 1 |
| STOX2 | 1 |
| STPG1 | 1 |
| STPG2 | 1 |
| STRA13 | 1 |
| STRN4 | 1 |
| STS | 1 |
| STT3B | 1 |
| STUB1 | 1 |
| STX11 | 1 |
| STX12 | 1 |
| STX17 | 1 |
| STX19 | 1 |
| STX1A | 1 |
| STX2 | 1 |
| STX4 | 1 |
| STX6 | 1 |
| STX7 | 1 |
| STXBP5 | 1 |
| STXBP6 | 1 |
| SUB1 | 1 |
| SUCLA2 | 1 |
| SUCLG1 | 1 |
| SUCLG2 | 1 |
| SUCNR1 | 1 |
| SUGP1 | 1 |
| SUGT1 | 1 |
| SULF1 | 1 |
| SULT1A1 | 1 |
| SULT1A2 | 1 |
| SULT1B1 | 1 |
| SULT1C2 | 1 |
| SULT1C3 | 1 |
| SULT1C4 | 1 |
| SUMF1 | 1 |
| SUMO3 | 1 |
| SUMO4 | 1 |
| SUN2 | 1 |
| SUOX | 1 |
| SUPT3H | 1 |
| SUPT4H1 | 1 |
| SUPT5H | 1 |
| SUPT7L | 1 |
| SUPV3L1 | 1 |
| SURF1 | 1 |
| SURF2 | 1 |
| SURF4 | 1 |
| SURF6 | 1 |
| SUSD2 | 1 |
| SUSD4 | 1 |
| SUSD5 | 1 |
| SUV39H2 | 1 |
| SUV420H1 | 1 |
| SVOP | 1 |
| SWAP70 | 1 |
| SWI5 | 1 |
| SYDE1 | 1 |
| SYK | 1 |
| SYMPK | 1 |
| SYN1 | 1 |
| SYN3 | 1 |
| SYNC | 1 |
| SYNCRIP | 1 |
| SYNDIG1L | 1 |
| SYNE1 | 1 |
| SYNE2 | 1 |
| SYNE3 | 1 |
| SYNGR1 | 1 |
| SYNJ2 | 1 |
| SYNJ2BP | 1 |
| SYNM | 1 |
| SYPL1 | 1 |
| SYS1 | 1 |
| SYT10 | 1 |
| SYT11 | 1 |
| SYT15 | 1 |
| SYT17 | 1 |
| SYT8 | 1 |
| SYTL2 | 1 |
| SZRD1 | 1 |
| SZT2 | 1 |
| TAAR2 | 1 |
| TAB2 | 1 |
| TAC3 | 1 |
| TAC4 | 1 |
| TACC2 | 1 |
| TACO1 | 1 |
| TACR2 | 1 |
| TACR3 | 1 |
| TADA3 | 1 |
| TAF1 | 1 |
| TAF10 | 1 |
| TAF11 | 1 |
| TAF13 | 1 |
| TAF15 | 1 |
| TAF1B | 1 |
| TAF4 | 1 |
| TAF4B | 1 |
| TAF6 | 1 |
| TAF6L | 1 |
| TAF7 | 1 |
| TAF8 | 1 |
| TAGAP | 1 |
| TAGLN | 1 |
| TAGLN2 | 1 |
| TALDO1 | 1 |
| TANC1 | 1 |
| TAOK2 | 1 |
| TAPT1.AS1 | 1 |
| TARBP2 | 1 |
| TARDBP | 1 |
| TAS2R1 | 1 |
| TAS2R10 | 1 |
| TAS2R13 | 1 |
| TAS2R14 | 1 |
| TAS2R16 | 1 |
| TAS2R19 | 1 |
| TAS2R20 | 1 |
| TAS2R3 | 1 |
| TAS2R38 | 1 |
| TAS2R4 | 1 |
| TAS2R43 | 1 |
| TAS2R46 | 1 |
| TAS2R5 | 1 |
| TAS2R50 | 1 |
| TAS2R60 | 1 |
| TAS2R8 | 1 |
| TAS2R9 | 1 |
| TATDN1 | 1 |
| TAX1BP3 | 1 |
| TBC1D10A | 1 |
| TBC1D10B | 1 |
| TBC1D13 | 1 |
| TBC1D17 | 1 |
| TBC1D2 | 1 |
| TBC1D21 | 1 |
| TBC1D22B | 1 |
| TBC1D23 | 1 |
| TBC1D30 | 1 |
| TBC1D4 | 1 |
| TBC1D5 | 1 |
| TBC1D9B | 1 |
| TBCB | 1 |
| TBCK | 1 |
| TBL1XR1 | 1 |
| TBL1Y | 1 |
| TBL2 | 1 |
| TBL3 | 1 |
| TBP | 1 |
| TBPL1 | 1 |
| TBR1 | 1 |
| TBX15 | 1 |
| TBX18 | 1 |
| TBX19 | 1 |
| TBX2 | 1 |
| TBX20 | 1 |
| TBX22 | 1 |
| TBX6 | 1 |
| TBXAS1 | 1 |
| TC2N | 1 |
| TCEAL4 | 1 |
| TCEAL6 | 1 |
| TCEAL8 | 1 |
| TCEB1 | 1 |
| TCEB2 | 1 |
| TCEB3 | 1 |
| TCERG1L | 1 |
| TCF19 | 1 |
| TCF20 | 1 |
| TCF3 | 1 |
| TCHP | 1 |
| TCL6 | 1 |
| TCP1 | 1 |
| TCP10L2 | 1 |
| TCP11L1 | 1 |
| TCTA | 1 |
| TCTE3 | 1 |
| TCTEX1D1 | 1 |
| TCTEX1D2 | 1 |
| TCTN2 | 1 |
| TCTN3 | 1 |
| TDG | 1 |
| TDO2 | 1 |
| TDRD5 | 1 |
| TDRD6 | 1 |
| TDRD7 | 1 |
| TDRP | 1 |
| TEAD1 | 1 |
| TEC | 1 |
| TECTA | 1 |
| TEF | 1 |
| TEK | 1 |
| TELO2 | 1 |
| TENM1 | 1 |
| TENM3 | 1 |
| TENM4 | 1 |
| TERF2IP | 1 |
| TES | 1 |
| TESPA1 | 1 |
| TET2 | 1 |
| TET3 | 1 |
| TEX12 | 1 |
| TEX14 | 1 |
| TEX2 | 1 |
| TEX26 | 1 |
| TEX29 | 1 |
| TF | 1 |
| TFAP2B | 1 |
| TFAP2D | 1 |
| TFAP4 | 1 |
| TFB1M | 1 |
| TFCP2L1 | 1 |
| TFDP1 | 1 |
| TFDP2 | 1 |
| TFE3 | 1 |
| TFEC | 1 |
| TFF2 | 1 |
| TFF3 | 1 |
| TFG | 1 |
| TFIP11 | 1 |
| TFPI2 | 1 |
| TFR2 | 1 |
| TG | 1 |
| TGFB1I1 | 1 |
| TGFB3 | 1 |
| TGFBI | 1 |
| TGFBR3 | 1 |
| TGIF2 | 1 |
| TGM2 | 1 |
| TGOLN2 | 1 |
| THAP10 | 1 |
| THAP4 | 1 |
| THAP7 | 1 |
| THAP8 | 1 |
| THBS1 | 1 |
| THBS2 | 1 |
| THEM4 | 1 |
| THEMIS | 1 |
| THEMIS2 | 1 |
| THOC3 | 1 |
| THOC6 | 1 |
| THOC7 | 1 |
| THRAP3 | 1 |
| THRB | 1 |
| THSD1 | 1 |
| THSD7A | 1 |
| THUMPD2 | 1 |
| THUMPD3 | 1 |
| THY1 | 1 |
| TIA1 | 1 |
| TIE1 | 1 |
| TIFAB | 1 |
| TIGD6 | 1 |
| TIGD7 | 1 |
| TIMD4 | 1 |
| TIMM17A | 1 |
| TIMM22 | 1 |
| TIMM23B | 1 |
| TIMM44 | 1 |
| TIMM50 | 1 |
| TIMM8A | 1 |
| TIMM8B | 1 |
| TIMMDC1 | 1 |
| TIMP1 | 1 |
| TIMP3 | 1 |
| TINAG | 1 |
| TINF2 | 1 |
| TIPRL | 1 |
| TIRAP | 1 |
| TJP2 | 1 |
| TK1 | 1 |
| TK2 | 1 |
| TKT | 1 |
| TKTL1 | 1 |
| TLCD1 | 1 |
| TLDC1 | 1 |
| TLDC2 | 1 |
| TLE1 | 1 |
| TLN1 | 1 |
| TLN2 | 1 |
| TLR1 | 1 |
| TLR10 | 1 |
| TLR2 | 1 |
| TLR5 | 1 |
| TLR7 | 1 |
| TLR8 | 1 |
| TM2D2 | 1 |
| TM4SF1 | 1 |
| TM4SF18 | 1 |
| TM4SF4 | 1 |
| TM6SF1 | 1 |
| TM7SF3 | 1 |
| TM9SF1 | 1 |
| TM9SF3 | 1 |
| TM9SF4 | 1 |
| TMBIM4 | 1 |
| TMBIM6 | 1 |
| TMC5 | 1 |
| TMC6 | 1 |
| TMC7 | 1 |
| TMC8 | 1 |
| TMCC3 | 1 |
| TMCO3 | 1 |
| TMCO4 | 1 |
| TMED1 | 1 |
| TMED10 | 1 |
| TMED2 | 1 |
| TMED3 | 1 |
| TMED4 | 1 |
| TMED9 | 1 |
| TMEM100 | 1 |
| TMEM104 | 1 |
| TMEM108 | 1 |
| TMEM109 | 1 |
| TMEM11 | 1 |
| TMEM115 | 1 |
| TMEM120A | 1 |
| TMEM123 | 1 |
| TMEM126A | 1 |
| TMEM127 | 1 |
| TMEM128 | 1 |
| TMEM132A | 1 |
| TMEM133 | 1 |
| TMEM138 | 1 |
| TMEM14B | 1 |
| TMEM14C | 1 |
| TMEM150B | 1 |
| TMEM151B | 1 |
| TMEM154 | 1 |
| TMEM159 | 1 |
| TMEM160 | 1 |
| TMEM161B | 1 |
| TMEM163 | 1 |
| TMEM165 | 1 |
| TMEM167A | 1 |
| TMEM171 | 1 |
| TMEM173 | 1 |
| TMEM174 | 1 |
| TMEM176A | 1 |
| TMEM176B | 1 |
| TMEM177 | 1 |
| TMEM178A | 1 |
| TMEM18 | 1 |
| TMEM184B | 1 |
| TMEM184C | 1 |
| TMEM185A | 1 |
| TMEM185B | 1 |
| TMEM19 | 1 |
| TMEM198B | 1 |
| TMEM2 | 1 |
| TMEM200A | 1 |
| TMEM204 | 1 |
| TMEM212 | 1 |
| TMEM214 | 1 |
| TMEM221 | 1 |
| TMEM222 | 1 |
| TMEM236 | 1 |
| TMEM242 | 1 |
| TMEM246 | 1 |
| TMEM248 | 1 |
| TMEM251 | 1 |
| TMEM254 | 1 |
| TMEM255B | 1 |
| TMEM256 | 1 |
| TMEM258 | 1 |
| TMEM261 | 1 |
| TMEM30A | 1 |
| TMEM30B | 1 |
| TMEM33 | 1 |
| TMEM43 | 1 |
| TMEM47 | 1 |
| TMEM5 | 1 |
| TMEM50A | 1 |
| TMEM51.AS1 | 1 |
| TMEM53 | 1 |
| TMEM54 | 1 |
| TMEM59 | 1 |
| TMEM60 | 1 |
| TMEM62 | 1 |
| TMEM65 | 1 |
| TMEM68 | 1 |
| TMEM69 | 1 |
| TMEM75 | 1 |
| TMEM87A | 1 |
| TMEM88 | 1 |
| TMEM8A | 1 |
| TMEM8C | 1 |
| TMEM95 | 1 |
| TMEM99 | 1 |
| TMEM9B | 1 |
| TMIGD2 | 1 |
| TMOD3 | 1 |
| TMPO | 1 |
| TMPRSS11A | 1 |
| TMPRSS11B | 1 |
| TMPRSS11D | 1 |
| TMPRSS5 | 1 |
| TMSB10 | 1 |
| TMSB15B | 1 |
| TMTC2 | 1 |
| TMTC3 | 1 |
| TMUB1 | 1 |
| TMUB2 | 1 |
| TMX3 | 1 |
| TMX4 | 1 |
| TNFAIP1 | 1 |
| TNFAIP3 | 1 |
| TNFAIP8L1 | 1 |
| TNFAIP8L2 | 1 |
| TNFRSF10A | 1 |
| TNFRSF10B | 1 |
| TNFRSF10D | 1 |
| TNFRSF12A | 1 |
| TNFRSF14 | 1 |
| TNFRSF1A | 1 |
| TNFRSF21 | 1 |
| TNFRSF25 | 1 |
| TNFRSF9 | 1 |
| TNFSF10 | 1 |
| TNFSF11 | 1 |
| TNFSF13B | 1 |
| TNFSF4 | 1 |
| TNFSF8 | 1 |
| TNFSF9 | 1 |
| TNIK | 1 |
| TNIP2 | 1 |
| TNMD | 1 |
| TNNI3 | 1 |
| TNNT1 | 1 |
| TNPO1 | 1 |
| TNPO3 | 1 |
| TNRC6C | 1 |
| TNS3 | 1 |
| TNS4 | 1 |
| TNXB | 1 |
| TOB1 | 1 |
| TOB2 | 1 |
| TOB2P1 | 1 |
| TOE1 | 1 |
| TOLLIP | 1 |
| TOM1 | 1 |
| TOMM22 | 1 |
| TOMM40 | 1 |
| TOMM5 | 1 |
| TOMM70A | 1 |
| TOP2A | 1 |
| TOR1A | 1 |
| TOR1AIP1 | 1 |
| TOR1AIP2 | 1 |
| TOR1B | 1 |
| TOX | 1 |
| TOX3 | 1 |
| TP53 | 1 |
| TP53I11 | 1 |
| TP53I13 | 1 |
| TP53I3 | 1 |
| TP53RK | 1 |
| TP73.AS1 | 1 |
| TPBG | 1 |
| TPD52L1 | 1 |
| TPD52L2 | 1 |
| TPD52L3 | 1 |
| TPGS2 | 1 |
| TPH1 | 1 |
| TPM1 | 1 |
| TPM2 | 1 |
| TPM4 | 1 |
| TPMT | 1 |
| TPRA1 | 1 |
| TPRG1 | 1 |
| TPRG1L | 1 |
| TPRKB | 1 |
| TPTEP1 | 1 |
| TPX2 | 1 |
| TRA2A | 1 |
| TRA2B | 1 |
| TRABD2B | 1 |
| TRADD | 1 |
| TRAF3IP1 | 1 |
| TRAF3IP2 | 1 |
| TRAF5 | 1 |
| TRAF7 | 1 |
| TRAK1 | 1 |
| TRAM1 | 1 |
| TRAM2 | 1 |
| TRAPPC2L | 1 |
| TRAPPC3 | 1 |
| TRAPPC3L | 1 |
| TRAPPC5 | 1 |
| TRAT1 | 1 |
| TRDMT1 | 1 |
| TREM2 | 1 |
| TREML1 | 1 |
| TREML4 | 1 |
| TRIAP1 | 1 |
| TRIM17 | 1 |
| TRIM24 | 1 |
| TRIM28 | 1 |
| TRIM31 | 1 |
| TRIM32 | 1 |
| TRIM33 | 1 |
| TRIM35 | 1 |
| TRIM36 | 1 |
| TRIM37 | 1 |
| TRIM41 | 1 |
| TRIM44 | 1 |
| TRIM45 | 1 |
| TRIM47 | 1 |
| TRIM49B | 1 |
| TRIM49C | 1 |
| TRIM5 | 1 |
| TRIM56 | 1 |
| TRIM59 | 1 |
| TRIM61 | 1 |
| TRIM63 | 1 |
| TRIM66 | 1 |
| TRIM7 | 1 |
| TRIM71 | 1 |
| TRIM74 | 1 |
| TRIOBP | 1 |
| TRIP12 | 1 |
| TRIP13 | 1 |
| TRIP4 | 1 |
| TRIT1 | 1 |
| TRMT112 | 1 |
| TRMT13 | 1 |
| TRO | 1 |
| TRPC4AP | 1 |
| TRPC5 | 1 |
| TRPM4 | 1 |
| TRPV1 | 1 |
| TRPV4 | 1 |
| TRUB1 | 1 |
| TRUB2 | 1 |
| TSACC | 1 |
| TSC2 | 1 |
| TSC22D3 | 1 |
| TSEN34 | 1 |
| TSEN54 | 1 |
| TSFM | 1 |
| TSG101 | 1 |
| TSGA13 | 1 |
| TSHR | 1 |
| TSN | 1 |
| TSPAN1 | 1 |
| TSPAN12 | 1 |
| TSPAN14 | 1 |
| TSPAN15 | 1 |
| TSPAN17 | 1 |
| TSPAN3 | 1 |
| TSPAN31 | 1 |
| TSPAN33 | 1 |
| TSPAN4 | 1 |
| TSPAN5 | 1 |
| TSPAN7 | 1 |
| TSPAN9 | 1 |
| TSPO | 1 |
| TSPO2 | 1 |
| TSPY26P | 1 |
| TSPYL4 | 1 |
| TSPYL6 | 1 |
| TSR1 | 1 |
| TSR2 | 1 |
| TSR3 | 1 |
| TSSC4 | 1 |
| TSSK1B | 1 |
| TSSK4 | 1 |
| TST | 1 |
| TSTA3 | 1 |
| TTBK2 | 1 |
| TTC14 | 1 |
| TTC17 | 1 |
| TTC23 | 1 |
| TTC26 | 1 |
| TTC27 | 1 |
| TTC37 | 1 |
| TTC38 | 1 |
| TTC8 | 1 |
| TTC9 | 1 |
| TTC9C | 1 |
| TTF1 | 1 |
| TTI2 | 1 |
| TTK | 1 |
| TTL | 1 |
| TTLL1 | 1 |
| TTLL11 | 1 |
| TTLL12 | 1 |
| TTLL5 | 1 |
| TTN | 1 |
| TTPAL | 1 |
| TTR | 1 |
| TTTY13 | 1 |
| TTTY6 | 1 |
| TTTY8 | 1 |
| TTYH2 | 1 |
| TUB | 1 |
| TUBA1A | 1 |
| TUBA1C | 1 |
| TUBA4A | 1 |
| TUBA8 | 1 |
| TUBB3 | 1 |
| TUBB4B | 1 |
| TUBB6 | 1 |
| TUBB7P | 1 |
| TUBG1 | 1 |
| TUFM | 1 |
| TUFT1 | 1 |
| TULP3 | 1 |
| TUSC1 | 1 |
| TUSC2 | 1 |
| TVP23C | 1 |
| TWF2 | 1 |
| TWSG1 | 1 |
| TXK | 1 |
| TXLNA | 1 |
| TXLNB | 1 |
| TXLNG | 1 |
| TXN | 1 |
| TXN2 | 1 |
| TXNDC11 | 1 |
| TXNDC12 | 1 |
| TXNDC15 | 1 |
| TXNDC16 | 1 |
| TXNDC17 | 1 |
| TXNDC8 | 1 |
| TXNDC9 | 1 |
| TXNIP | 1 |
| TXNRD1 | 1 |
| TYMS | 1 |
| TYROBP | 1 |
| TYW1 | 1 |
| U2AF1L4 | 1 |
| UAP1 | 1 |
| UBA3 | 1 |
| UBA52 | 1 |
| UBA6 | 1 |
| UBAC1 | 1 |
| UBAC2 | 1 |
| UBAP1 | 1 |
| UBAP2 | 1 |
| UBASH3B | 1 |
| UBC | 1 |
| UBE2A | 1 |
| UBE2B | 1 |
| UBE2C | 1 |
| UBE2D3 | 1 |
| UBE2DNL | 1 |
| UBE2E3 | 1 |
| UBE2G1 | 1 |
| UBE2H | 1 |
| UBE2M | 1 |
| UBE2MP1 | 1 |
| UBE2NL | 1 |
| UBE2Q1 | 1 |
| UBE2Q2 | 1 |
| UBE2Q2P2 | 1 |
| UBE2T | 1 |
| UBE2U | 1 |
| UBE2V2 | 1 |
| UBE2W | 1 |
| UBE2Z | 1 |
| UBE3B | 1 |
| UBE3C | 1 |
| UBFD1 | 1 |
| UBIAD1 | 1 |
| UBL3 | 1 |
| UBL4A | 1 |
| UBL4B | 1 |
| UBL5 | 1 |
| UBL7 | 1 |
| UBN2 | 1 |
| UBQLN3 | 1 |
| UBTD2 | 1 |
| UBTF | 1 |
| UBXN2A | 1 |
| UCHL3 | 1 |
| UCHL5 | 1 |
| UCK1 | 1 |
| UCN2 | 1 |
| UCP1 | 1 |
| UCP2 | 1 |
| UCP3 | 1 |
| UFC1 | 1 |
| UFL1 | 1 |
| UFM1 | 1 |
| UFSP2 | 1 |
| UGCG | 1 |
| UGDH | 1 |
| UGP2 | 1 |
| UGT2B15 | 1 |
| UGT2B28 | 1 |
| UGT2B7 | 1 |
| UGT8 | 1 |
| UHMK1 | 1 |
| UHRF1 | 1 |
| UHRF1BP1 | 1 |
| ULBP3 | 1 |
| ULK3 | 1 |
| ULK4 | 1 |
| UNC119B | 1 |
| UNC45A | 1 |
| UNC45B | 1 |
| UNC5C | 1 |
| UNG | 1 |
| UPF1 | 1 |
| UPF3A | 1 |
| UPF3B | 1 |
| UPK3B | 1 |
| UPRT | 1 |
| UQCC1 | 1 |
| UQCRC1 | 1 |
| UQCRC2 | 1 |
| UQCRFS1 | 1 |
| URGCP.MRPS24 | 1 |
| URM1 | 1 |
| UROD | 1 |
| UROS | 1 |
| USE1 | 1 |
| USH1C | 1 |
| USH1G | 1 |
| USO1 | 1 |
| USP10 | 1 |
| USP14 | 1 |
| USP2 | 1 |
| USP24 | 1 |
| USP31 | 1 |
| USP32 | 1 |
| USP36 | 1 |
| USP39 | 1 |
| USP44 | 1 |
| USP48 | 1 |
| USP49 | 1 |
| USP5 | 1 |
| USP50 | 1 |
| USP53 | 1 |
| USP6 | 1 |
| USP7 | 1 |
| UTP11L | 1 |
| UTP14A | 1 |
| UTP15 | 1 |
| UTP20 | 1 |
| UTP3 | 1 |
| UTP6 | 1 |
| UTS2B | 1 |
| UTS2R | 1 |
| UXS1 | 1 |
| UXT | 1 |
| VAMP1 | 1 |
| VAMP3 | 1 |
| VAMP5 | 1 |
| VAMP7 | 1 |
| VAMP8 | 1 |
| VAPB | 1 |
| VARS | 1 |
| VASH1 | 1 |
| VASN | 1 |
| VASP | 1 |
| VAT1 | 1 |
| VAV1 | 1 |
| VAV3 | 1 |
| VAX2 | 1 |
| VCAN | 1 |
| VCP | 1 |
| VCPIP1 | 1 |
| VCY | 1 |
| VDAC1 | 1 |
| VDAC3 | 1 |
| VEGFB | 1 |
| VEGFC | 1 |
| VEPH1 | 1 |
| VGLL1 | 1 |
| VGLL3 | 1 |
| VHL | 1 |
| VHLL | 1 |
| VIM | 1 |
| VIPAS39 | 1 |
| VKORC1 | 1 |
| VKORC1L1 | 1 |
| VMP1 | 1 |
| VN1R1 | 1 |
| VN1R2 | 1 |
| VN1R3 | 1 |
| VNN2 | 1 |
| VOPP1 | 1 |
| VPREB1 | 1 |
| VPS11 | 1 |
| VPS13B | 1 |
| VPS18 | 1 |
| VPS25 | 1 |
| VPS26A | 1 |
| VPS29 | 1 |
| VPS33A | 1 |
| VPS35 | 1 |
| VPS39 | 1 |
| VPS41 | 1 |
| VPS4A | 1 |
| VPS4B | 1 |
| VPS52 | 1 |
| VSTM2A | 1 |
| VTA1 | 1 |
| VTI1A | 1 |
| VTI1B | 1 |
| VTRNA1.1 | 1 |
| VTRNA1.3 | 1 |
| VWA1 | 1 |
| VWA3B | 1 |
| VWDE | 1 |
| VWF | 1 |
| WARS | 1 |
| WAS | 1 |
| WASF3 | 1 |
| WBP1L | 1 |
| WBP4 | 1 |
| WBP5 | 1 |
| WBSCR16 | 1 |
| WBSCR22 | 1 |
| WDFY1 | 1 |
| WDFY4 | 1 |
| WDPCP | 1 |
| WDR1 | 1 |
| WDR13 | 1 |
| WDR18 | 1 |
| WDR24 | 1 |
| WDR34 | 1 |
| WDR35 | 1 |
| WDR36 | 1 |
| WDR37 | 1 |
| WDR41 | 1 |
| WDR43 | 1 |
| WDR46 | 1 |
| WDR47 | 1 |
| WDR49 | 1 |
| WDR52 | 1 |
| WDR54 | 1 |
| WDR6 | 1 |
| WDR63 | 1 |
| WDR74 | 1 |
| WDR76 | 1 |
| WDR77 | 1 |
| WDR82 | 1 |
| WDR83 | 1 |
| WDR83OS | 1 |
| WDR86 | 1 |
| WDR91 | 1 |
| WDTC1 | 1 |
| WEE2.AS1 | 1 |
| WFDC1 | 1 |
| WFDC10B | 1 |
| WFDC11 | 1 |
| WFDC13 | 1 |
| WFDC6 | 1 |
| WFS1 | 1 |
| WIBG | 1 |
| WIPF3 | 1 |
| WIPI1 | 1 |
| WIPI2 | 1 |
| WISP2 | 1 |
| WNK3 | 1 |
| WNT1 | 1 |
| WNT2 | 1 |
| WNT5A | 1 |
| WNT5B | 1 |
| WNT7B | 1 |
| WNT9A | 1 |
| WNT9B | 1 |
| WRAP53 | 1 |
| WRB | 1 |
| WRNIP1 | 1 |
| WSB2 | 1 |
| WTH3DI | 1 |
| WWC3 | 1 |
| WWP1 | 1 |
| XAB2 | 1 |
| XAF1 | 1 |
| XAGE2 | 1 |
| XCR1 | 1 |
| XG | 1 |
| XIAP | 1 |
| XIRP1 | 1 |
| XKR3 | 1 |
| XKR4 | 1 |
| XKR5 | 1 |
| XKRX | 1 |
| XKRY2 | 1 |
| XPNPEP1 | 1 |
| XPO6 | 1 |
| XRCC1 | 1 |
| XRCC4 | 1 |
| XRCC5 | 1 |
| XRCC6 | 1 |
| XYLT1 | 1 |
| XYLT2 | 1 |
| YAP1 | 1 |
| YARS2 | 1 |
| YBX3 | 1 |
| YDJC | 1 |
| YIF1A | 1 |
| YIPF2 | 1 |
| YIPF3 | 1 |
| YIPF4 | 1 |
| YIPF5 | 1 |
| YIPF7 | 1 |
| YKT6 | 1 |
| YME1L1 | 1 |
| YOD1 | 1 |
| YPEL1 | 1 |
| YPEL3 | 1 |
| YPEL5 | 1 |
| YTHDF2 | 1 |
| YTHDF3 | 1 |
| YWHAB | 1 |
| YWHAE | 1 |
| YWHAG | 1 |
| YWHAH | 1 |
| YWHAQ | 1 |
| YWHAQP8 | 1 |
| YY1 | 1 |
| YY1AP1 | 1 |
| ZAK | 1 |
| ZBBX | 1 |
| ZBED5 | 1 |
| ZBED6CL | 1 |
| ZBTB14 | 1 |
| ZBTB2 | 1 |
| ZBTB25 | 1 |
| ZBTB26 | 1 |
| ZBTB3 | 1 |
| ZBTB34 | 1 |
| ZBTB4 | 1 |
| ZBTB40 | 1 |
| ZBTB42 | 1 |
| ZBTB45 | 1 |
| ZBTB46 | 1 |
| ZBTB47 | 1 |
| ZBTB48 | 1 |
| ZBTB7A | 1 |
| ZBTB7C | 1 |
| ZBTB8OS | 1 |
| ZC2HC1A | 1 |
| ZC3H14 | 1 |
| ZC3H18 | 1 |
| ZC3H4 | 1 |
| ZC3H7A | 1 |
| ZC3H7B | 1 |
| ZC3H8 | 1 |
| ZC3HC1 | 1 |
| ZCCHC10 | 1 |
| ZCCHC11 | 1 |
| ZCCHC17 | 1 |
| ZCCHC18 | 1 |
| ZCCHC9 | 1 |
| ZCRB1 | 1 |
| ZDHHC12 | 1 |
| ZDHHC13 | 1 |
| ZDHHC16 | 1 |
| ZDHHC20 | 1 |
| ZDHHC24 | 1 |
| ZDHHC5 | 1 |
| ZDHHC7 | 1 |
| ZDHHC8P1 | 1 |
| ZDHHC9 | 1 |
| ZEB2 | 1 |
| ZFAND5 | 1 |
| ZFHX3 | 1 |
| ZFHX4 | 1 |
| ZFP36L1 | 1 |
| ZFP36L2 | 1 |
| ZFP42 | 1 |
| ZFPL1 | 1 |
| ZFYVE16 | 1 |
| ZFYVE20 | 1 |
| ZFYVE21 | 1 |
| ZG16 | 1 |
| ZG16B | 1 |
| ZGPAT | 1 |
| ZHX1.C8orf76 | 1 |
| ZHX1 | 1 |
| ZHX3 | 1 |
| ZIC2 | 1 |
| ZIC5 | 1 |
| ZKSCAN7 | 1 |
| ZKSCAN8 | 1 |
| ZMAT1 | 1 |
| ZMAT2 | 1 |
| ZMAT3 | 1 |
| ZMAT4 | 1 |
| ZMIZ2 | 1 |
| ZMPSTE24 | 1 |
| ZNF10 | 1 |
| ZNF101 | 1 |
| ZNF106 | 1 |
| ZNF107 | 1 |
| ZNF117 | 1 |
| ZNF12 | 1 |
| ZNF134 | 1 |
| ZNF138 | 1 |
| ZNF157 | 1 |
| ZNF169 | 1 |
| ZNF177 | 1 |
| ZNF195 | 1 |
| ZNF197 | 1 |
| ZNF202 | 1 |
| ZNF215 | 1 |
| ZNF219 | 1 |
| ZNF230 | 1 |
| ZNF232 | 1 |
| ZNF233 | 1 |
| ZNF235 | 1 |
| ZNF236 | 1 |
| ZNF248 | 1 |
| ZNF253 | 1 |
| ZNF254 | 1 |
| ZNF257 | 1 |
| ZNF267 | 1 |
| ZNF273 | 1 |
| ZNF274 | 1 |
| ZNF275 | 1 |
| ZNF276 | 1 |
| ZNF280A | 1 |
| ZNF280D | 1 |
| ZNF286A | 1 |
| ZNF3 | 1 |
| ZNF318 | 1 |
| ZNF319 | 1 |
| ZNF324 | 1 |
| ZNF329 | 1 |
| ZNF354B | 1 |
| ZNF362 | 1 |
| ZNF366 | 1 |
| ZNF367 | 1 |
| ZNF385A | 1 |
| ZNF385B | 1 |
| ZNF385D | 1 |
| ZNF395 | 1 |
| ZNF407 | 1 |
| ZNF423 | 1 |
| ZNF425 | 1 |
| ZNF429 | 1 |
| ZNF431 | 1 |
| ZNF446 | 1 |
| ZNF451 | 1 |
| ZNF462 | 1 |
| ZNF467 | 1 |
| ZNF468 | 1 |
| ZNF471 | 1 |
| ZNF474 | 1 |
| ZNF48 | 1 |
| ZNF491 | 1 |
| ZNF492 | 1 |
| ZNF493 | 1 |
| ZNF496 | 1 |
| ZNF506 | 1 |
| ZNF514 | 1 |
| ZNF519 | 1 |
| ZNF532 | 1 |
| ZNF540 | 1 |
| ZNF549 | 1 |
| ZNF551 | 1 |
| ZNF561 | 1 |
| ZNF567 | 1 |
| ZNF573 | 1 |
| ZNF574 | 1 |
| ZNF576 | 1 |
| ZNF577 | 1 |
| ZNF581 | 1 |
| ZNF582 | 1 |
| ZNF583 | 1 |
| ZNF585A | 1 |
| ZNF586 | 1 |
| ZNF589 | 1 |
| ZNF605 | 1 |
| ZNF606 | 1 |
| ZNF608 | 1 |
| ZNF609 | 1 |
| ZNF613 | 1 |
| ZNF622 | 1 |
| ZNF624 | 1 |
| ZNF638 | 1 |
| ZNF644 | 1 |
| ZNF649 | 1 |
| ZNF660 | 1 |
| ZNF664.FAM101A | 1 |
| ZNF667.AS1 | 1 |
| ZNF667 | 1 |
| ZNF668 | 1 |
| ZNF669 | 1 |
| ZNF678 | 1 |
| ZNF684 | 1 |
| ZNF69 | 1 |
| ZNF691 | 1 |
| ZNF692 | 1 |
| ZNF699 | 1 |
| ZNF704 | 1 |
| ZNF708 | 1 |
| ZNF709 | 1 |
| ZNF711 | 1 |
| ZNF713 | 1 |
| ZNF716 | 1 |
| ZNF721 | 1 |
| ZNF729 | 1 |
| ZNF732 | 1 |
| ZNF747 | 1 |
| ZNF75D | 1 |
| ZNF767P | 1 |
| ZNF768 | 1 |
| ZNF773 | 1 |
| ZNF778 | 1 |
| ZNF780B | 1 |
| ZNF781 | 1 |
| ZNF782 | 1 |
| ZNF784 | 1 |
| ZNF785 | 1 |
| ZNF789 | 1 |
| ZNF792 | 1 |
| ZNF808 | 1 |
| ZNF816 | 1 |
| ZNF823 | 1 |
| ZNF827 | 1 |
| ZNF835 | 1 |
| ZNF839 | 1 |
| ZNF844 | 1 |
| ZNF85 | 1 |
| ZNF862 | 1 |
| ZNF91 | 1 |
| ZNF99 | 1 |
| ZNHIT3 | 1 |
| ZNRF2P1 | 1 |
| ZRANB2 | 1 |
| ZSCAN12 | 1 |
| ZSCAN29 | 1 |
| ZSCAN31 | 1 |
| ZSCAN4 | 1 |
| ZSWIM2 | 1 |
| ZSWIM5 | 1 |
| ZSWIM6 | 1 |
| ZW10 | 1 |
| ZWILCH | 1 |
| ZYX | 1 |
| ZZZ3 | 1 |
| SV2C | 0.979167 |
| A1BG | 0.958333 |
| A1CF | 0.958333 |
| AADACL3 | 0.958333 |
| AADAT | 0.958333 |
| AATK.AS1 | 0.958333 |
| ABAT | 0.958333 |
| ABCA1 | 0.958333 |
| ABCA7 | 0.958333 |
| ABCC2 | 0.958333 |
| ABCC3 | 0.958333 |
| ABCD2 | 0.958333 |
| ABCD3 | 0.958333 |
| ABCG4 | 0.958333 |
| ABHD1 | 0.958333 |
| ABHD16B | 0.958333 |
| ABI1 | 0.958333 |
| ABO | 0.958333 |
| ACBD3 | 0.958333 |
| ACER1 | 0.958333 |
| ACOT11 | 0.958333 |
| ACSF3 | 0.958333 |
| ACSM3 | 0.958333 |
| ACSS3 | 0.958333 |
| ACTL7A | 0.958333 |
| ADAM23 | 0.958333 |
| ADAM5 | 0.958333 |
| ADAM8 | 0.958333 |
| ADAMTS4 | 0.958333 |
| ADCK4 | 0.958333 |
| ADIPOQ | 0.958333 |
| ADORA1 | 0.958333 |
| ADPRH | 0.958333 |
| AFAP1 | 0.958333 |
| AFP | 0.958333 |
| AGAP2 | 0.958333 |
| AGER | 0.958333 |
| AGO4 | 0.958333 |
| AGPS | 0.958333 |
| AGXT2 | 0.958333 |
| AK8 | 0.958333 |
| AKTIP | 0.958333 |
| ALG2 | 0.958333 |
| ALOX12P2 | 0.958333 |
| AMBN | 0.958333 |
| AMDHD1 | 0.958333 |
| AMPD2 | 0.958333 |
| ANGPTL6 | 0.958333 |
| ANK1 | 0.958333 |
| ANKDD1A | 0.958333 |
| ANKRD18A | 0.958333 |
| ANKRD26P1 | 0.958333 |
| ANKRD36C | 0.958333 |
| ANKRD55 | 0.958333 |
| ANKRD6 | 0.958333 |
| ANKRD61 | 0.958333 |
| ANO4 | 0.958333 |
| ANTXR2 | 0.958333 |
| ANXA2R | 0.958333 |
| APBB1 | 0.958333 |
| API5 | 0.958333 |
| APOA1 | 0.958333 |
| APOC3 | 0.958333 |
| AQP8 | 0.958333 |
| AQPEP | 0.958333 |
| ARHGAP42 | 0.958333 |
| ARHGDIG | 0.958333 |
| ARHGEF15 | 0.958333 |
| ARID4B | 0.958333 |
| ARL13A | 0.958333 |
| ARL6IP6 | 0.958333 |
| ARMCX3 | 0.958333 |
| ARSA | 0.958333 |
| ARSK | 0.958333 |
| ASB18 | 0.958333 |
| ASCL3 | 0.958333 |
| ASF1A | 0.958333 |
| ASTL | 0.958333 |
| ASUN | 0.958333 |
| ATL2 | 0.958333 |
| ATP10B | 0.958333 |
| ATP13A3 | 0.958333 |
| ATP2A1 | 0.958333 |
| ATP6V1E1 | 0.958333 |
| ATP7B | 0.958333 |
| ATP8B5P | 0.958333 |
| AWAT1 | 0.958333 |
| AXIN2 | 0.958333 |
| B3GALNT1 | 0.958333 |
| B3GNT8 | 0.958333 |
| BAD | 0.958333 |
| BAG1 | 0.958333 |
| BAZ1A | 0.958333 |
| BAZ2A | 0.958333 |
| BBOX1 | 0.958333 |
| BBS7 | 0.958333 |
| BCAT1 | 0.958333 |
| BCL9L | 0.958333 |
| BCR | 0.958333 |
| BCS1L | 0.958333 |
| BEGAIN | 0.958333 |
| BEX5 | 0.958333 |
| BNIP1 | 0.958333 |
| BNIPL | 0.958333 |
| BORA | 0.958333 |
| BPIFA2 | 0.958333 |
| BPIFB4 | 0.958333 |
| BPNT1 | 0.958333 |
| BRCC3 | 0.958333 |
| BRS3 | 0.958333 |
| BRSK2 | 0.958333 |
| BSPH1 | 0.958333 |
| BTG4 | 0.958333 |
| BYSL | 0.958333 |
| BZW2 | 0.958333 |
| C11orf31 | 0.958333 |
| C11orf52 | 0.958333 |
| C12orf50 | 0.958333 |
| C14orf79 | 0.958333 |
| C15orf39 | 0.958333 |
| C15orf54 | 0.958333 |
| C16orf74 | 0.958333 |
| C17orf104 | 0.958333 |
| C17orf99 | 0.958333 |
| C18orf12 | 0.958333 |
| C18orf21 | 0.958333 |
| C19orf35 | 0.958333 |
| C19orf45 | 0.958333 |
| C20orf173 | 0.958333 |
| C22orf39 | 0.958333 |
| C2orf88 | 0.958333 |
| C3orf33 | 0.958333 |
| C3orf65 | 0.958333 |
| C5orf47 | 0.958333 |
| C6orf120 | 0.958333 |
| C6orf132 | 0.958333 |
| C6orf136 | 0.958333 |
| C6orf15 | 0.958333 |
| C6orf99 | 0.958333 |
| C7orf25 | 0.958333 |
| C9orf135 | 0.958333 |
| C9orf85 | 0.958333 |
| CA2 | 0.958333 |
| CABP1 | 0.958333 |
| CACNA1E | 0.958333 |
| CACNA2D2 | 0.958333 |
| CACNG1 | 0.958333 |
| CACNG7 | 0.958333 |
| CALHM1 | 0.958333 |
| CALML3 | 0.958333 |
| CAPN5 | 0.958333 |
| CAPS | 0.958333 |
| CARD11 | 0.958333 |
| CARD16 | 0.958333 |
| CASC5 | 0.958333 |
| CASP3 | 0.958333 |
| CAST | 0.958333 |
| CASZ1 | 0.958333 |
| CATIP | 0.958333 |
| CATSPER2P1 | 0.958333 |
| CBX7 | 0.958333 |
| CCDC113 | 0.958333 |
| CCDC115 | 0.958333 |
| CCDC167 | 0.958333 |
| CCDC168 | 0.958333 |
| CCDC174 | 0.958333 |
| CCDC28B | 0.958333 |
| CCDC43 | 0.958333 |
| CCDC73 | 0.958333 |
| CCDC94 | 0.958333 |
| CCL19 | 0.958333 |
| CCL28 | 0.958333 |
| CCM2 | 0.958333 |
| CCNJL | 0.958333 |
| CCR9 | 0.958333 |
| CD200R1L | 0.958333 |
| CD244 | 0.958333 |
| CD300LB | 0.958333 |
| CD33 | 0.958333 |
| CD47 | 0.958333 |
| CD72 | 0.958333 |
| CDC37L1 | 0.958333 |
| CDC42SE2 | 0.958333 |
| CDK1 | 0.958333 |
| CDKL2 | 0.958333 |
| CDKL3 | 0.958333 |
| CDKL4 | 0.958333 |
| CDKL5 | 0.958333 |
| CDRT4 | 0.958333 |
| CDYL | 0.958333 |
| CEBPD | 0.958333 |
| CENPF | 0.958333 |
| CENPK | 0.958333 |
| CENPL | 0.958333 |
| CENPT | 0.958333 |
| CEP41 | 0.958333 |
| CEP85 | 0.958333 |
| CEP95 | 0.958333 |
| CERS3 | 0.958333 |
| CETN1 | 0.958333 |
| CFC1B | 0.958333 |
| CFHR4 | 0.958333 |
| CGA | 0.958333 |
| CHAT | 0.958333 |
| CHDC2 | 0.958333 |
| CHMP5 | 0.958333 |
| CHRNA2 | 0.958333 |
| CHST10 | 0.958333 |
| CHST12 | 0.958333 |
| CHST5 | 0.958333 |
| CHST7 | 0.958333 |
| CILP | 0.958333 |
| CKMT1A | 0.958333 |
| CKMT1B | 0.958333 |
| CLC | 0.958333 |
| CLCA3P | 0.958333 |
| CLDN16 | 0.958333 |
| CLDN22 | 0.958333 |
| CLDN9 | 0.958333 |
| CLEC4M | 0.958333 |
| CNBD1 | 0.958333 |
| CNFN | 0.958333 |
| CNIH3 | 0.958333 |
| CNOT1 | 0.958333 |
| CNPY2 | 0.958333 |
| CNRIP1 | 0.958333 |
| CNTNAP5 | 0.958333 |
| COA1 | 0.958333 |
| COG7 | 0.958333 |
| COL13A1 | 0.958333 |
| COL18A1 | 0.958333 |
| COL5A3 | 0.958333 |
| COL8A1 | 0.958333 |
| COLQ | 0.958333 |
| CORO1A | 0.958333 |
| CPT2 | 0.958333 |
| CPZ | 0.958333 |
| CRAMP1L | 0.958333 |
| CREG2 | 0.958333 |
| CRKL | 0.958333 |
| CRTAC1 | 0.958333 |
| CRYZL1 | 0.958333 |
| CSDC2 | 0.958333 |
| CSNK1A1P1 | 0.958333 |
| CST13P | 0.958333 |
| CST2 | 0.958333 |
| CST8 | 0.958333 |
| CSTL1 | 0.958333 |
| CTCF | 0.958333 |
| CTIF | 0.958333 |
| CTLA4 | 0.958333 |
| CTRB2 | 0.958333 |
| CTSS | 0.958333 |
| CWC22 | 0.958333 |
| CWH43 | 0.958333 |
| CXorf67 | 0.958333 |
| CYB5R2 | 0.958333 |
| CYLD | 0.958333 |
| CYP1A1 | 0.958333 |
| CYP1A2 | 0.958333 |
| CYP1B1.AS1 | 0.958333 |
| CYP20A1 | 0.958333 |
| CYP27C1 | 0.958333 |
| CYP2C8 | 0.958333 |
| CYTIP | 0.958333 |
| DAAM1 | 0.958333 |
| DAP3 | 0.958333 |
| DBI | 0.958333 |
| DBX1 | 0.958333 |
| DCAKD | 0.958333 |
| DCDC2 | 0.958333 |
| DCLRE1C | 0.958333 |
| DDX19A | 0.958333 |
| DDX21 | 0.958333 |
| DDX51 | 0.958333 |
| DDX53 | 0.958333 |
| DDX56 | 0.958333 |
| DEF6 | 0.958333 |
| DEFB133 | 0.958333 |
| DEFB135 | 0.958333 |
| DEPDC4 | 0.958333 |
| DEPDC7 | 0.958333 |
| DGKH | 0.958333 |
| DIO3 | 0.958333 |
| DIP2B | 0.958333 |
| DIRC2 | 0.958333 |
| DLG5.AS1 | 0.958333 |
| DLX5 | 0.958333 |
| DMAP1 | 0.958333 |
| DNAJA1P5 | 0.958333 |
| DNAJB12 | 0.958333 |
| DNAJB6 | 0.958333 |
| DNAJC4 | 0.958333 |
| DNAJC5B | 0.958333 |
| DOC2B | 0.958333 |
| DOK4 | 0.958333 |
| DONSON | 0.958333 |
| DPEP2 | 0.958333 |
| DRICH1 | 0.958333 |
| DSG1 | 0.958333 |
| DSP | 0.958333 |
| DUOXA1 | 0.958333 |
| DUSP27 | 0.958333 |
| DVL3 | 0.958333 |
| E2F7 | 0.958333 |
| ECSIT | 0.958333 |
| EFCAB11 | 0.958333 |
| EFCAB3 | 0.958333 |
| EFCAB5 | 0.958333 |
| EFHC2 | 0.958333 |
| EFNA3 | 0.958333 |
| EFNA5 | 0.958333 |
| EFNB3 | 0.958333 |
| EGFL7 | 0.958333 |
| EHBP1 | 0.958333 |
| EHMT2 | 0.958333 |
| EIF2B2 | 0.958333 |
| EIF5A2 | 0.958333 |
| ELF3 | 0.958333 |
| EMILIN1 | 0.958333 |
| EML1 | 0.958333 |
| ENGASE | 0.958333 |
| ENTHD1 | 0.958333 |
| EPC1 | 0.958333 |
| ERAP2 | 0.958333 |
| ERCC8 | 0.958333 |
| ERI2 | 0.958333 |
| ERI3 | 0.958333 |
| ERMN | 0.958333 |
| ERVK13.1 | 0.958333 |
| ESYT3 | 0.958333 |
| EVI2B | 0.958333 |
| EWSR1 | 0.958333 |
| EYS | 0.958333 |
| F2RL1 | 0.958333 |
| FABP12 | 0.958333 |
| FABP6 | 0.958333 |
| FAF2 | 0.958333 |
| FAIM3 | 0.958333 |
| FAM124A | 0.958333 |
| FAM126A | 0.958333 |
| FAM150A | 0.958333 |
| FAM160A1 | 0.958333 |
| FAM177A1 | 0.958333 |
| FAM192A | 0.958333 |
| FAM193B | 0.958333 |
| FAM19A3 | 0.958333 |
| FAM223A | 0.958333 |
| FAM81B | 0.958333 |
| FAM83A | 0.958333 |
| FAM86B1 | 0.958333 |
| FAM89B | 0.958333 |
| FAM92A1P2 | 0.958333 |
| FASLG | 0.958333 |
| FBLN1 | 0.958333 |
| FBXO34 | 0.958333 |
| FBXO9 | 0.958333 |
| FBXW12 | 0.958333 |
| FCRL5 | 0.958333 |
| FEM1B | 0.958333 |
| FER1L6 | 0.958333 |
| FEV | 0.958333 |
| FFAR4 | 0.958333 |
| FGF14 | 0.958333 |
| FGF8 | 0.958333 |
| FGFBP2 | 0.958333 |
| FGFR1OP | 0.958333 |
| FICD | 0.958333 |
| FIZ1 | 0.958333 |
| FKBP5 | 0.958333 |
| FLJ13744 | 0.958333 |
| FLJ30403 | 0.958333 |
| FLJ30679 | 0.958333 |
| FLJ46120 | 0.958333 |
| FLOT1 | 0.958333 |
| FOLR4 | 0.958333 |
| FOXH1 | 0.958333 |
| FOXN3 | 0.958333 |
| FPR1 | 0.958333 |
| FRMD4B | 0.958333 |
| FRS3 | 0.958333 |
| FSCB | 0.958333 |
| FTMT | 0.958333 |
| FUT1 | 0.958333 |
| FXN | 0.958333 |
| FXYD4 | 0.958333 |
| FZD4 | 0.958333 |
| GABRA1 | 0.958333 |
| GABRP | 0.958333 |
| GABRR1 | 0.958333 |
| GALNT16 | 0.958333 |
| GALR1 | 0.958333 |
| GALT | 0.958333 |
| GAR1 | 0.958333 |
| GAS8 | 0.958333 |
| GBGT1 | 0.958333 |
| GDPD1 | 0.958333 |
| GEMIN5 | 0.958333 |
| GEMIN8P4 | 0.958333 |
| GGA3 | 0.958333 |
| GGCX | 0.958333 |
| GGTLC2 | 0.958333 |
| GHRH | 0.958333 |
| GJB2 | 0.958333 |
| GJD4 | 0.958333 |
| GLB1 | 0.958333 |
| GLDN | 0.958333 |
| GLIPR1L2 | 0.958333 |
| GLP2R | 0.958333 |
| GLRA2 | 0.958333 |
| GLRA4 | 0.958333 |
| GLRX2 | 0.958333 |
| GLT6D1 | 0.958333 |
| GNA12 | 0.958333 |
| GNRH2 | 0.958333 |
| GPANK1 | 0.958333 |
| GPR112 | 0.958333 |
| GPR143 | 0.958333 |
| GPR148 | 0.958333 |
| GPR149 | 0.958333 |
| GPR174 | 0.958333 |
| GPR32 | 0.958333 |
| GPR35 | 0.958333 |
| GRHL1 | 0.958333 |
| GRIPAP1 | 0.958333 |
| GRM2 | 0.958333 |
| GSK3A | 0.958333 |
| GTF2H2C_2 | 0.958333 |
| GTF3C5 | 0.958333 |
| GUK1 | 0.958333 |
| HAND2 | 0.958333 |
| HAPLN3 | 0.958333 |
| HCN3 | 0.958333 |
| HDAC11 | 0.958333 |
| HECTD4 | 0.958333 |
| HIP1R | 0.958333 |
| HIST1H1E | 0.958333 |
| HIST1H2AK | 0.958333 |
| HMG20A | 0.958333 |
| HNRNPU | 0.958333 |
| HOXA10 | 0.958333 |
| HOXA11 | 0.958333 |
| HOXA13 | 0.958333 |
| HSD17B7P2 | 0.958333 |
| HSFX2 | 0.958333 |
| HSPA12A | 0.958333 |
| HSPA6 | 0.958333 |
| HSPB9 | 0.958333 |
| HTATSF1P2 | 0.958333 |
| HTR4 | 0.958333 |
| HYAL1 | 0.958333 |
| HYAL4 | 0.958333 |
| IARS2 | 0.958333 |
| ICAM1 | 0.958333 |
| IFI16 | 0.958333 |
| IFNA2 | 0.958333 |
| IFNE | 0.958333 |
| IFNL1 | 0.958333 |
| IFNW1 | 0.958333 |
| IGFALS | 0.958333 |
| IGFL1 | 0.958333 |
| IGSF9B | 0.958333 |
| IKBKG | 0.958333 |
| IL12RB2 | 0.958333 |
| IL17RD | 0.958333 |
| IL2 | 0.958333 |
| ILKAP | 0.958333 |
| ING2 | 0.958333 |
| INHA | 0.958333 |
| INHBC | 0.958333 |
| INPP1 | 0.958333 |
| INSM2 | 0.958333 |
| IRS1 | 0.958333 |
| ISCU | 0.958333 |
| ITGAE | 0.958333 |
| ITGB4 | 0.958333 |
| ITSN2 | 0.958333 |
| JAK3 | 0.958333 |
| JAKMIP3 | 0.958333 |
| JMJD1C.AS1 | 0.958333 |
| KAAG1 | 0.958333 |
| KATNBL1 | 0.958333 |
| KCMF1 | 0.958333 |
| KCNA2 | 0.958333 |
| KCNC2 | 0.958333 |
| KCND3 | 0.958333 |
| KCNJ10 | 0.958333 |
| KCNV1 | 0.958333 |
| KDELR2 | 0.958333 |
| KIAA0040 | 0.958333 |
| KIAA0319L | 0.958333 |
| KIAA1161 | 0.958333 |
| KIAA1731 | 0.958333 |
| KIAA1875 | 0.958333 |
| KIF13B | 0.958333 |
| KIF17 | 0.958333 |
| KIF4A | 0.958333 |
| KLB | 0.958333 |
| KLF5 | 0.958333 |
| KLF8 | 0.958333 |
| KLHDC1 | 0.958333 |
| KLHDC9 | 0.958333 |
| KLHL10 | 0.958333 |
| KLHL12 | 0.958333 |
| KLHL18 | 0.958333 |
| KLHL30.AS1 | 0.958333 |
| KLK2 | 0.958333 |
| KRT13 | 0.958333 |
| KRT2 | 0.958333 |
| KRT38 | 0.958333 |
| KRT4 | 0.958333 |
| KRT9 | 0.958333 |
| KRTAP11.1 | 0.958333 |
| KRTAP12.3 | 0.958333 |
| LAD1 | 0.958333 |
| LAIR2 | 0.958333 |
| LCE1F | 0.958333 |
| LCK | 0.958333 |
| LCORL | 0.958333 |
| LDHAL6B | 0.958333 |
| LDLRAD2 | 0.958333 |
| LENG1 | 0.958333 |
| LETM1 | 0.958333 |
| LGI4 | 0.958333 |
| LILRP2 | 0.958333 |
| LIM2 | 0.958333 |
| LIME1 | 0.958333 |
| LIN7A | 0.958333 |
| LINC00052 | 0.958333 |
| LINC00087 | 0.958333 |
| LINC00167 | 0.958333 |
| LINC00304 | 0.958333 |
| LINC00312 | 0.958333 |
| LINC00474 | 0.958333 |
| LINC00528 | 0.958333 |
| LINC00661 | 0.958333 |
| LINC00671 | 0.958333 |
| LINC00896 | 0.958333 |
| LINC01089 | 0.958333 |
| LINC01106 | 0.958333 |
| LINC01138 | 0.958333 |
| LINC01366 | 0.958333 |
| LINC01446 | 0.958333 |
| LIX1 | 0.958333 |
| LLGL1 | 0.958333 |
| LLGL2 | 0.958333 |
| LMO2 | 0.958333 |
| LMOD1 | 0.958333 |
| LOC100128398 | 0.958333 |
| LOC100128751 | 0.958333 |
| LOC100128775 | 0.958333 |
| LOC100129534 | 0.958333 |
| LOC100130236 | 0.958333 |
| LOC100131756 | 0.958333 |
| LOC100131860 | 0.958333 |
| LOC100132354 | 0.958333 |
| LOC100133130 | 0.958333 |
| LOC100287896 | 0.958333 |
| LOC100506127 | 0.958333 |
| LOC100507191 | 0.958333 |
| LOC100996455 | 0.958333 |
| LOC151760 | 0.958333 |
| LOC158960 | 0.958333 |
| LOC285696 | 0.958333 |
| LOC388882 | 0.958333 |
| LOC390956 | 0.958333 |
| LOC400692 | 0.958333 |
| LOC441956 | 0.958333 |
| LOC442132 | 0.958333 |
| LOC642757 | 0.958333 |
| LOC642776 | 0.958333 |
| LOC642947 | 0.958333 |
| LOC644172 | 0.958333 |
| LOC646358 | 0.958333 |
| LOC653486 | 0.958333 |
| LOC692247 | 0.958333 |
| LOC731157 | 0.958333 |
| LPCAT1 | 0.958333 |
| LPPR5 | 0.958333 |
| LRIT1 | 0.958333 |
| LRIT2 | 0.958333 |
| LRRC25 | 0.958333 |
| LRRC55 | 0.958333 |
| LRRC73 | 0.958333 |
| LRRC75B | 0.958333 |
| LRSAM1 | 0.958333 |
| LSS | 0.958333 |
| LY9 | 0.958333 |
| LYPD2 | 0.958333 |
| LYRM5 | 0.958333 |
| MAGEB2 | 0.958333 |
| MAGED4 | 0.958333 |
| MAGEL2 | 0.958333 |
| MALAT1 | 0.958333 |
| MAP3K6 | 0.958333 |
| MAP3K9 | 0.958333 |
| MAPT | 0.958333 |
| 3-Mar | 0.958333 |
| 7-Mar | 0.958333 |
| MATN1.AS1 | 0.958333 |
| MBL2 | 0.958333 |
| MBNL2 | 0.958333 |
| MC1R | 0.958333 |
| MCPH1 | 0.958333 |
| MCRS1 | 0.958333 |
| MDFIC | 0.958333 |
| MEFV | 0.958333 |
| METTL14 | 0.958333 |
| METTL22 | 0.958333 |
| MGC45922 | 0.958333 |
| MICAL3 | 0.958333 |
| MIR10B | 0.958333 |
| MIR133B | 0.958333 |
| MIR141 | 0.958333 |
| MIR184 | 0.958333 |
| MIR186 | 0.958333 |
| MIR212 | 0.958333 |
| MIR30B | 0.958333 |
| MIR33A | 0.958333 |
| MIR9.3 | 0.958333 |
| MIR93 | 0.958333 |
| MIR96 | 0.958333 |
| MKI67 | 0.958333 |
| MKKS | 0.958333 |
| MLLT10 | 0.958333 |
| MLLT4.AS1 | 0.958333 |
| MLLT6 | 0.958333 |
| MMACHC | 0.958333 |
| MMRN1 | 0.958333 |
| MORN3 | 0.958333 |
| MOSPD1 | 0.958333 |
| MPL | 0.958333 |
| MPP7 | 0.958333 |
| MPPED1 | 0.958333 |
| MROH6 | 0.958333 |
| MROH8 | 0.958333 |
| MRPL13 | 0.958333 |
| MRPL2 | 0.958333 |
| MRPL34 | 0.958333 |
| MRPL4 | 0.958333 |
| MRPL44 | 0.958333 |
| MRPS18B | 0.958333 |
| MRPS9 | 0.958333 |
| MSANTD1 | 0.958333 |
| MSANTD3 | 0.958333 |
| MTERF1 | 0.958333 |
| MTMR8 | 0.958333 |
| MTOR | 0.958333 |
| MX2 | 0.958333 |
| MXD1 | 0.958333 |
| MYCBPAP | 0.958333 |
| MYO1H | 0.958333 |
| MYO9B | 0.958333 |
| NAA20 | 0.958333 |
| NAB1 | 0.958333 |
| NANOGNB | 0.958333 |
| NARS | 0.958333 |
| NAV2 | 0.958333 |
| NCKAP5L | 0.958333 |
| NCMAP | 0.958333 |
| NCOA5 | 0.958333 |
| NCOR2 | 0.958333 |
| NCR3 | 0.958333 |
| NDE1 | 0.958333 |
| NFU1 | 0.958333 |
| NGB | 0.958333 |
| NGF | 0.958333 |
| NGRN | 0.958333 |
| NHLRC1 | 0.958333 |
| NIFK | 0.958333 |
| NIPBL | 0.958333 |
| NKAIN2 | 0.958333 |
| NKAP | 0.958333 |
| NKD2 | 0.958333 |
| NKG7 | 0.958333 |
| NKX2.6 | 0.958333 |
| NLE1 | 0.958333 |
| NMB | 0.958333 |
| NME6 | 0.958333 |
| NME8 | 0.958333 |
| NMU | 0.958333 |
| NOM1 | 0.958333 |
| NOS3 | 0.958333 |
| NOXO1 | 0.958333 |
| NREP | 0.958333 |
| NRIP2 | 0.958333 |
| NRM | 0.958333 |
| NSL1 | 0.958333 |
| NT5DC4 | 0.958333 |
| NUDT17 | 0.958333 |
| NXF3 | 0.958333 |
| NXNL1 | 0.958333 |
| NXPE3 | 0.958333 |
| OCIAD2 | 0.958333 |
| OGG1 | 0.958333 |
| OLIG2 | 0.958333 |
| OPN3 | 0.958333 |
| OPRK1 | 0.958333 |
| OR10C1 | 0.958333 |
| OR10G2 | 0.958333 |
| OR13C4 | 0.958333 |
| OR13C8 | 0.958333 |
| OR13C9 | 0.958333 |
| OR13H1 | 0.958333 |
| OR1J4 | 0.958333 |
| OR1L3 | 0.958333 |
| OR2AG1 | 0.958333 |
| OR2D3 | 0.958333 |
| OR2G3 | 0.958333 |
| OR2M7 | 0.958333 |
| OR2T35 | 0.958333 |
| OR4K2 | 0.958333 |
| OR4S2 | 0.958333 |
| OR4X2 | 0.958333 |
| OR51B5 | 0.958333 |
| OR51G1 | 0.958333 |
| OR51Q1 | 0.958333 |
| OR52B4 | 0.958333 |
| OR52E2 | 0.958333 |
| OR52J3 | 0.958333 |
| OR52M1 | 0.958333 |
| OR5D18 | 0.958333 |
| OR5M9 | 0.958333 |
| OR6C75 | 0.958333 |
| OR6M1 | 0.958333 |
| OR6W1P | 0.958333 |
| OR7C2 | 0.958333 |
| OR7G3 | 0.958333 |
| OR8B8 | 0.958333 |
| OR8D2 | 0.958333 |
| ORAI1 | 0.958333 |
| OSMR | 0.958333 |
| OSTM1 | 0.958333 |
| OSTN | 0.958333 |
| OTOP2 | 0.958333 |
| OXER1 | 0.958333 |
| OXR1 | 0.958333 |
| P2RX3 | 0.958333 |
| PAGE2 | 0.958333 |
| PAK2 | 0.958333 |
| PANK1 | 0.958333 |
| PANK4 | 0.958333 |
| PAPLN | 0.958333 |
| PARD3 | 0.958333 |
| PARP14 | 0.958333 |
| PARS2 | 0.958333 |
| PCDH15 | 0.958333 |
| PCED1B | 0.958333 |
| PCF11 | 0.958333 |
| PCK1 | 0.958333 |
| PDCL3 | 0.958333 |
| PDGFC | 0.958333 |
| PDLIM7 | 0.958333 |
| PDZD11 | 0.958333 |
| PEX10 | 0.958333 |
| PGAM2 | 0.958333 |
| PHACTR4 | 0.958333 |
| PIAS1 | 0.958333 |
| PIGF | 0.958333 |
| PIM3 | 0.958333 |
| PIP | 0.958333 |
| PITPNC1 | 0.958333 |
| PLA1A | 0.958333 |
| PLA2G12B | 0.958333 |
| PLAC4 | 0.958333 |
| PLD5 | 0.958333 |
| PLEKHA8 | 0.958333 |
| PLEKHB1 | 0.958333 |
| PLEKHF1 | 0.958333 |
| PLEKHG4B | 0.958333 |
| PLG | 0.958333 |
| PLLP | 0.958333 |
| PNCK | 0.958333 |
| PNMAL1 | 0.958333 |
| PODNL1 | 0.958333 |
| POLR2G | 0.958333 |
| POLR2J3 | 0.958333 |
| POM121L8P | 0.958333 |
| POP1 | 0.958333 |
| POU2F2 | 0.958333 |
| POU4F2 | 0.958333 |
| PPEF2 | 0.958333 |
| PPM1N | 0.958333 |
| PPP1R12C | 0.958333 |
| PPP1R3F | 0.958333 |
| PPP4R2 | 0.958333 |
| PPT2 | 0.958333 |
| PQLC3 | 0.958333 |
| PRADC1 | 0.958333 |
| PRH1 | 0.958333 |
| PRKCI | 0.958333 |
| PRL | 0.958333 |
| PRMT2 | 0.958333 |
| PROL1 | 0.958333 |
| PRPF40B | 0.958333 |
| PRR23C | 0.958333 |
| PRR35 | 0.958333 |
| PRR5 | 0.958333 |
| PRRC2C | 0.958333 |
| PRSS3 | 0.958333 |
| PRSS48 | 0.958333 |
| PRSS57 | 0.958333 |
| PSD3 | 0.958333 |
| PSMA8 | 0.958333 |
| PSMD3 | 0.958333 |
| PSMG4 | 0.958333 |
| PTPLAD2 | 0.958333 |
| PTPN7 | 0.958333 |
| PTPN9 | 0.958333 |
| PTPRT | 0.958333 |
| PTTG1 | 0.958333 |
| PYCR1 | 0.958333 |
| PYROXD2 | 0.958333 |
| RAB9BP1 | 0.958333 |
| RABL3 | 0.958333 |
| RAD1 | 0.958333 |
| RAI14 | 0.958333 |
| RALGAPA1 | 0.958333 |
| RANBP6 | 0.958333 |
| RAPGEF1 | 0.958333 |
| RAPSN | 0.958333 |
| RARRES2 | 0.958333 |
| RASL11B | 0.958333 |
| RBM12B | 0.958333 |
| RBM27 | 0.958333 |
| RBMS3 | 0.958333 |
| RBMXL3 | 0.958333 |
| RBP5 | 0.958333 |
| RCBTB1 | 0.958333 |
| RCCD1 | 0.958333 |
| RCHY1 | 0.958333 |
| RDH16 | 0.958333 |
| REC114 | 0.958333 |
| REEP1 | 0.958333 |
| RELT | 0.958333 |
| REN | 0.958333 |
| RESP18 | 0.958333 |
| RETNLB | 0.958333 |
| RFPL3 | 0.958333 |
| RGL2 | 0.958333 |
| RGS17 | 0.958333 |
| RGS21 | 0.958333 |
| RGS6 | 0.958333 |
| RGS9 | 0.958333 |
| RHD | 0.958333 |
| RHEBL1 | 0.958333 |
| RHOBTB1 | 0.958333 |
| RHOQ | 0.958333 |
| RHOU | 0.958333 |
| RIIAD1 | 0.958333 |
| RIMS3 | 0.958333 |
| RIPK4 | 0.958333 |
| RNASE11 | 0.958333 |
| RNF44 | 0.958333 |
| RNF7 | 0.958333 |
| RNLS | 0.958333 |
| RNU5F.1 | 0.958333 |
| ROCK2 | 0.958333 |
| RPL11 | 0.958333 |
| RPL18A | 0.958333 |
| RPL23P8 | 0.958333 |
| RPL35A | 0.958333 |
| RPL37 | 0.958333 |
| RPL41 | 0.958333 |
| RPS20P27 | 0.958333 |
| RTCA | 0.958333 |
| RXFP4 | 0.958333 |
| S100A12 | 0.958333 |
| S100A7A | 0.958333 |
| SAA2 | 0.958333 |
| SAMD12 | 0.958333 |
| SAP30 | 0.958333 |
| SAT2 | 0.958333 |
| SCAI | 0.958333 |
| SCARNA1 | 0.958333 |
| SCG2 | 0.958333 |
| SCGB1A1 | 0.958333 |
| SCMH1 | 0.958333 |
| SCN4B | 0.958333 |
| SDC1 | 0.958333 |
| SEC24B | 0.958333 |
| SEC24C | 0.958333 |
| SEH1L | 0.958333 |
| SEMA3D | 0.958333 |
| SEMG1 | 0.958333 |
| SEPT5.GP1BB | 0.958333 |
| SERPINA7 | 0.958333 |
| SERTAD2 | 0.958333 |
| SESN2 | 0.958333 |
| SFRP1 | 0.958333 |
| SGK2 | 0.958333 |
| SGPP2 | 0.958333 |
| SGTA | 0.958333 |
| SH3YL1 | 0.958333 |
| SHC4 | 0.958333 |
| SHH | 0.958333 |
| SIGLEC1 | 0.958333 |
| SIGLEC14 | 0.958333 |
| SIGLEC16 | 0.958333 |
| SIGLEC9 | 0.958333 |
| SIGMAR1 | 0.958333 |
| SIPA1L3 | 0.958333 |
| SIRT3 | 0.958333 |
| SIX2 | 0.958333 |
| SIX5 | 0.958333 |
| SKA1 | 0.958333 |
| SKP1 | 0.958333 |
| SLAIN1 | 0.958333 |
| SLC10A2 | 0.958333 |
| SLC10A3 | 0.958333 |
| SLC10A6 | 0.958333 |
| SLC11A1 | 0.958333 |
| SLC16A1 | 0.958333 |
| SLC16A12 | 0.958333 |
| SLC16A8 | 0.958333 |
| SLC22A1 | 0.958333 |
| SLC25A33 | 0.958333 |
| SLC27A3 | 0.958333 |
| SLC29A3 | 0.958333 |
| SLC2A5 | 0.958333 |
| SLC2A7 | 0.958333 |
| SLC30A5 | 0.958333 |
| SLC36A3 | 0.958333 |
| SLC38A11 | 0.958333 |
| SLC38A4 | 0.958333 |
| SLC39A2 | 0.958333 |
| SLC39A9 | 0.958333 |
| SLC46A2 | 0.958333 |
| SLC5A12 | 0.958333 |
| SLC6A1 | 0.958333 |
| SLC6A17 | 0.958333 |
| SLC6A9 | 0.958333 |
| SLC7A14 | 0.958333 |
| SLC8A1 | 0.958333 |
| SLCO1B1 | 0.958333 |
| SLCO4A1.AS1 | 0.958333 |
| SLCO4A1 | 0.958333 |
| SLCO5A1 | 0.958333 |
| SLFN14 | 0.958333 |
| SLITRK6 | 0.958333 |
| SMC2 | 0.958333 |
| SMCP | 0.958333 |
| SMR3A | 0.958333 |
| SMR3B | 0.958333 |
| SMS | 0.958333 |
| SMU1 | 0.958333 |
| SNAI3.AS1 | 0.958333 |
| SNAP91 | 0.958333 |
| SNORA14B | 0.958333 |
| SNORA38 | 0.958333 |
| SNORA44 | 0.958333 |
| SNORD104 | 0.958333 |
| SNORD109B | 0.958333 |
| SNORD115.25 | 0.958333 |
| SNORD115.34 | 0.958333 |
| SNORD115.40 | 0.958333 |
| SNORD13 | 0.958333 |
| SNORD13P3 | 0.958333 |
| SNORD28 | 0.958333 |
| SNORD35A | 0.958333 |
| SNORD38A | 0.958333 |
| SNORD42B | 0.958333 |
| SNORD63 | 0.958333 |
| SNORD76 | 0.958333 |
| SNORD95 | 0.958333 |
| SOCS4 | 0.958333 |
| SOST | 0.958333 |
| SOX4 | 0.958333 |
| SPACA4 | 0.958333 |
| SPATA31E1 | 0.958333 |
| SPATA41 | 0.958333 |
| SPATA5L1 | 0.958333 |
| SPATA6L | 0.958333 |
| SPDL1 | 0.958333 |
| SPEF1 | 0.958333 |
| SPIN2A | 0.958333 |
| SPRYD4 | 0.958333 |
| SRA1 | 0.958333 |
| SRGAP1 | 0.958333 |
| SRP72 | 0.958333 |
| SRRM3 | 0.958333 |
| SRSF2 | 0.958333 |
| SRSF8 | 0.958333 |
| SRY | 0.958333 |
| SSMEM1 | 0.958333 |
| SSU72 | 0.958333 |
| ST3GAL4 | 0.958333 |
| STAG3L4 | 0.958333 |
| STARD5 | 0.958333 |
| STEAP1 | 0.958333 |
| STEAP1B | 0.958333 |
| STH | 0.958333 |
| STKLD1 | 0.958333 |
| STXBP2 | 0.958333 |
| SUPT6H | 0.958333 |
| SV2B | 0.958333 |
| SVIL | 0.958333 |
| SYDE2 | 0.958333 |
| TAAR1 | 0.958333 |
| TACC3 | 0.958333 |
| TADA1 | 0.958333 |
| TAF1L | 0.958333 |
| TAPBPL | 0.958333 |
| TARP | 0.958333 |
| TAS2R40 | 0.958333 |
| TBC1D19 | 0.958333 |
| TBC1D8B | 0.958333 |
| TBCE | 0.958333 |
| TBRG1 | 0.958333 |
| TCEA3 | 0.958333 |
| TCEAL5 | 0.958333 |
| TCEANC2 | 0.958333 |
| TCF7L2 | 0.958333 |
| TCHH | 0.958333 |
| TDRD10 | 0.958333 |
| TEX37 | 0.958333 |
| TFAM | 0.958333 |
| TFAP2A | 0.958333 |
| TFPI | 0.958333 |
| TFRC | 0.958333 |
| TGFBRAP1 | 0.958333 |
| THADA | 0.958333 |
| THEM6 | 0.958333 |
| THG1L | 0.958333 |
| THSD7B | 0.958333 |
| THUMPD1 | 0.958333 |
| TIMM10 | 0.958333 |
| TIMM13 | 0.958333 |
| TLL1 | 0.958333 |
| TMEM125 | 0.958333 |
| TMEM129 | 0.958333 |
| TMEM141 | 0.958333 |
| TMEM14A | 0.958333 |
| TMEM14E | 0.958333 |
| TMEM170A | 0.958333 |
| TMEM186 | 0.958333 |
| TMEM202 | 0.958333 |
| TMEM217 | 0.958333 |
| TMEM230 | 0.958333 |
| TMEM262 | 0.958333 |
| TMEM27 | 0.958333 |
| TMEM44 | 0.958333 |
| TMEM45B | 0.958333 |
| TMEM51 | 0.958333 |
| TMEM92 | 0.958333 |
| TNFRSF17 | 0.958333 |
| TNK2 | 0.958333 |
| TNNC1 | 0.958333 |
| TOMM40L | 0.958333 |
| TPCN2 | 0.958333 |
| TPGS1 | 0.958333 |
| TPH2 | 0.958333 |
| TRAK2 | 0.958333 |
| TREM1 | 0.958333 |
| TRIM25 | 0.958333 |
| TRIM54 | 0.958333 |
| TRIM55 | 0.958333 |
| TRIM62 | 0.958333 |
| TRPM3 | 0.958333 |
| TRPM8 | 0.958333 |
| TSHZ3 | 0.958333 |
| TSPAN13 | 0.958333 |
| TSPAN32 | 0.958333 |
| TSPYL1 | 0.958333 |
| TSSC1 | 0.958333 |
| TSTD1 | 0.958333 |
| TTC22 | 0.958333 |
| TTC4 | 0.958333 |
| TTPA | 0.958333 |
| TUBA4B | 0.958333 |
| TXNRD3NB | 0.958333 |
| U2AF2 | 0.958333 |
| UBE2E4P | 0.958333 |
| UBQLN1 | 0.958333 |
| UBR4 | 0.958333 |
| UBR5 | 0.958333 |
| UGT2B17 | 0.958333 |
| UHRF2 | 0.958333 |
| ULBP2 | 0.958333 |
| UNC13A | 0.958333 |
| UNC50 | 0.958333 |
| UNC80 | 0.958333 |
| URAD | 0.958333 |
| USP37 | 0.958333 |
| USP51 | 0.958333 |
| USPL1 | 0.958333 |
| UTS2 | 0.958333 |
| VAMP4 | 0.958333 |
| VAV2 | 0.958333 |
| VBP1 | 0.958333 |
| VDAC2 | 0.958333 |
| VIMP | 0.958333 |
| VMO1 | 0.958333 |
| VPS28 | 0.958333 |
| VPS33B | 0.958333 |
| VSIG1 | 0.958333 |
| WDR12 | 0.958333 |
| WDR16 | 0.958333 |
| WDR3 | 0.958333 |
| WDR38 | 0.958333 |
| WDR64 | 0.958333 |
| WDR66 | 0.958333 |
| WDR72 | 0.958333 |
| WDR89 | 0.958333 |
| WDR90 | 0.958333 |
| WFIKKN2 | 0.958333 |
| WIF1 | 0.958333 |
| WWC2 | 0.958333 |
| XKR8 | 0.958333 |
| YAF2 | 0.958333 |
| ZAN | 0.958333 |
| ZBTB32 | 0.958333 |
| ZBTB37 | 0.958333 |
| ZBTB9 | 0.958333 |
| ZC3HAV1L | 0.958333 |
| ZCWPW2 | 0.958333 |
| ZDHHC6 | 0.958333 |
| ZFAT | 0.958333 |
| ZFP92 | 0.958333 |
| ZMAT5 | 0.958333 |
| ZMYND8 | 0.958333 |
| ZNF114 | 0.958333 |
| ZNF131 | 0.958333 |
| ZNF141 | 0.958333 |
| ZNF148 | 0.958333 |
| ZNF182 | 0.958333 |
| ZNF189 | 0.958333 |
| ZNF214 | 0.958333 |
| ZNF224 | 0.958333 |
| ZNF264 | 0.958333 |
| ZNF266 | 0.958333 |
| ZNF281 | 0.958333 |
| ZNF292 | 0.958333 |
| ZNF29P | 0.958333 |
| ZNF345 | 0.958333 |
| ZNF382 | 0.958333 |
| ZNF43 | 0.958333 |
| ZNF442 | 0.958333 |
| ZNF479 | 0.958333 |
| ZNF484 | 0.958333 |
| ZNF490 | 0.958333 |
| ZNF513 | 0.958333 |
| ZNF530 | 0.958333 |
| ZNF536 | 0.958333 |
| ZNF547 | 0.958333 |
| ZNF552 | 0.958333 |
| ZNF571 | 0.958333 |
| ZNF599 | 0.958333 |
| ZNF611 | 0.958333 |
| ZNF630 | 0.958333 |
| ZNF652 | 0.958333 |
| ZNF676 | 0.958333 |
| ZNF683 | 0.958333 |
| ZNF70 | 0.958333 |
| ZNF718 | 0.958333 |
| ZNF805 | 0.958333 |
| ZNF841 | 0.958333 |
| ZNF93 | 0.958333 |
| ZNRD1.AS1 | 0.958333 |
| ZSWIM3 | 0.958333 |
| ZSWIM8 | 0.958333 |
| AADAC | 0.916667 |
| AADACL2 | 0.916667 |
| AASS | 0.916667 |
| ABCA2 | 0.916667 |
| ABCA3 | 0.916667 |
| ABCA9 | 0.916667 |
| ACCSL | 0.916667 |
| ACKR4 | 0.916667 |
| ACMSD | 0.916667 |
| ACOX3 | 0.916667 |
| ACP5 | 0.916667 |
| ACP6 | 0.916667 |
| ACSM2A | 0.916667 |
| ACTL6A | 0.916667 |
| ACTRT2 | 0.916667 |
| ACY1 | 0.916667 |
| ACY3 | 0.916667 |
| ADAL | 0.916667 |
| ADAM11 | 0.916667 |
| ADAMTS19 | 0.916667 |
| ADAMTS7 | 0.916667 |
| ADAMTS8 | 0.916667 |
| ADAMTSL4 | 0.916667 |
| ADAT1 | 0.916667 |
| ADRA2C | 0.916667 |
| ADRB1 | 0.916667 |
| AHCYL2 | 0.916667 |
| AK9 | 0.916667 |
| AKAP3 | 0.916667 |
| AKAP5 | 0.916667 |
| AKR1C1 | 0.916667 |
| AKR1D1 | 0.916667 |
| ALAD | 0.916667 |
| ALDH16A1 | 0.916667 |
| ALDOB | 0.916667 |
| ALMS1P | 0.916667 |
| ALX4 | 0.916667 |
| AMPD3 | 0.916667 |
| ANK2 | 0.916667 |
| ANKRD32 | 0.916667 |
| ANKRD42 | 0.916667 |
| ANO3 | 0.916667 |
| ANTXRL | 0.916667 |
| AP3B1 | 0.916667 |
| APC2 | 0.916667 |
| APOBEC3B | 0.916667 |
| APOO | 0.916667 |
| ARGFX | 0.916667 |
| ARHGAP23 | 0.916667 |
| ARHGAP36 | 0.916667 |
| ARMC5 | 0.916667 |
| ARMCX5 | 0.916667 |
| ARRB2 | 0.916667 |
| ART3 | 0.916667 |
| ASB4 | 0.916667 |
| ASCL2 | 0.916667 |
| ASMT | 0.916667 |
| ASTE1 | 0.916667 |
| ATAD3A | 0.916667 |
| ATG2B | 0.916667 |
| ATG7 | 0.916667 |
| ATP10A | 0.916667 |
| ATP13A4 | 0.916667 |
| ATP1A2 | 0.916667 |
| ATP5I | 0.916667 |
| ATP6V0A1 | 0.916667 |
| ATP6V0E2.AS1 | 0.916667 |
| ATP6V1A | 0.916667 |
| ATP8A1 | 0.916667 |
| ATP9B | 0.916667 |
| AUNIP | 0.916667 |
| B3GNT7 | 0.916667 |
| BAHD1 | 0.916667 |
| BAIAP2.AS1 | 0.916667 |
| BATF2 | 0.916667 |
| BCKDHA | 0.916667 |
| BCL11A | 0.916667 |
| BMP5 | 0.916667 |
| BOD1L2 | 0.916667 |
| BPI | 0.916667 |
| BPTF | 0.916667 |
| BPY2B | 0.916667 |
| BRD9 | 0.916667 |
| BSDC1 | 0.916667 |
| BSPRY | 0.916667 |
| BTNL2 | 0.916667 |
| BTNL3 | 0.916667 |
| BUB1B | 0.916667 |
| C10orf88 | 0.916667 |
| C11orf49 | 0.916667 |
| C12orf29 | 0.916667 |
| C12orf60 | 0.916667 |
| C14orf80 | 0.916667 |
| C15orf56 | 0.916667 |
| C16orf87 | 0.916667 |
| C1orf210 | 0.916667 |
| C20orf85 | 0.916667 |
| C2orf80 | 0.916667 |
| C2orf82 | 0.916667 |
| C3orf49 | 0.916667 |
| C4orf29 | 0.916667 |
| C4orf50 | 0.916667 |
| C6orf106 | 0.916667 |
| C6orf201 | 0.916667 |
| C7orf49 | 0.916667 |
| C7orf55.LUC7L2 | 0.916667 |
| C8orf33 | 0.916667 |
| C9 | 0.916667 |
| C9orf131 | 0.916667 |
| C9orf139 | 0.916667 |
| C9orf142 | 0.916667 |
| C9orf170 | 0.916667 |
| C9orf171 | 0.916667 |
| C9orf62 | 0.916667 |
| CA3 | 0.916667 |
| CA5B | 0.916667 |
| CAAP1 | 0.916667 |
| CABP7 | 0.916667 |
| CADM3 | 0.916667 |
| CALB2 | 0.916667 |
| CALCOCO2 | 0.916667 |
| CALML6 | 0.916667 |
| CAMK1 | 0.916667 |
| CAMKK2 | 0.916667 |
| CAPN15 | 0.916667 |
| CAPNS2 | 0.916667 |
| CAPSL | 0.916667 |
| CATSPER4 | 0.916667 |
| CATSPERB | 0.916667 |
| CBR3 | 0.916667 |
| CBR4 | 0.916667 |
| CCDC102A | 0.916667 |
| CCDC103 | 0.916667 |
| CCDC126 | 0.916667 |
| CCDC146 | 0.916667 |
| CCDC185 | 0.916667 |
| CCDC42B | 0.916667 |
| CCDC64B | 0.916667 |
| CCDC7 | 0.916667 |
| CCDC8 | 0.916667 |
| CCDC81 | 0.916667 |
| CCDC96 | 0.916667 |
| CCIN | 0.916667 |
| CCL23 | 0.916667 |
| CCNO | 0.916667 |
| CCR7 | 0.916667 |
| CCT8L2 | 0.916667 |
| CD1C | 0.916667 |
| CD22 | 0.916667 |
| CDH11 | 0.916667 |
| CDK10 | 0.916667 |
| CDK11A | 0.916667 |
| CDK2AP2 | 0.916667 |
| CDKN1C | 0.916667 |
| CEACAM19 | 0.916667 |
| CEACAM4 | 0.916667 |
| CEBPE | 0.916667 |
| CELP | 0.916667 |
| CELSR2 | 0.916667 |
| CENPI | 0.916667 |
| CENPP | 0.916667 |
| CEP112 | 0.916667 |
| CEP131 | 0.916667 |
| CEP170 | 0.916667 |
| CEP192 | 0.916667 |
| CEP57L1 | 0.916667 |
| CEP63 | 0.916667 |
| CEP85L | 0.916667 |
| CEPT1 | 0.916667 |
| CFHR2 | 0.916667 |
| CFHR5 | 0.916667 |
| CGGBP1 | 0.916667 |
| CHCHD4 | 0.916667 |
| CHD1 | 0.916667 |
| CHN1 | 0.916667 |
| CHST1 | 0.916667 |
| CHST2 | 0.916667 |
| CHSY3 | 0.916667 |
| CIAPIN1 | 0.916667 |
| CIART | 0.916667 |
| CIB3 | 0.916667 |
| CIDEB | 0.916667 |
| CLDN4 | 0.916667 |
| CLEC12A | 0.916667 |
| CLLU1OS | 0.916667 |
| CLNS1A | 0.916667 |
| CLRN1 | 0.916667 |
| CNKSR1 | 0.916667 |
| CNNM3 | 0.916667 |
| CNTN1 | 0.916667 |
| COBL | 0.916667 |
| COBLL1 | 0.916667 |
| COG3 | 0.916667 |
| COL4A3 | 0.916667 |
| COL5A2 | 0.916667 |
| COL6A1 | 0.916667 |
| COQ5 | 0.916667 |
| COX18 | 0.916667 |
| COX5B | 0.916667 |
| CRHR1.IT1 | 0.916667 |
| CRP | 0.916667 |
| CRYGS | 0.916667 |
| CSPG4 | 0.916667 |
| CUL2 | 0.916667 |
| CWC15 | 0.916667 |
| CXorf58 | 0.916667 |
| CYGB | 0.916667 |
| DAND5 | 0.916667 |
| DAPK1 | 0.916667 |
| DCAF13 | 0.916667 |
| DCUN1D2 | 0.916667 |
| DDX26B | 0.916667 |
| DEFB124 | 0.916667 |
| DEFB125 | 0.916667 |
| DEFB136 | 0.916667 |
| DENND4A | 0.916667 |
| DENND5A | 0.916667 |
| DEXI | 0.916667 |
| DGCR14 | 0.916667 |
| DGKI | 0.916667 |
| DGUOK | 0.916667 |
| DHX33 | 0.916667 |
| DHX57 | 0.916667 |
| DIXDC1 | 0.916667 |
| DKFZP434L187 | 0.916667 |
| DKK3 | 0.916667 |
| DMBT1P1 | 0.916667 |
| DMPK | 0.916667 |
| DMXL2 | 0.916667 |
| DNAH8 | 0.916667 |
| DNAJB1 | 0.916667 |
| DNAJB3 | 0.916667 |
| DNAJC22 | 0.916667 |
| DNAL1 | 0.916667 |
| DNALI1 | 0.916667 |
| DNASE2B | 0.916667 |
| DNM1P46 | 0.916667 |
| DNM2 | 0.916667 |
| DPCR1 | 0.916667 |
| DPF2 | 0.916667 |
| DRD3 | 0.916667 |
| DRP2 | 0.916667 |
| DTD2 | 0.916667 |
| DTWD1 | 0.916667 |
| DUOX1 | 0.916667 |
| DUSP12 | 0.916667 |
| DUSP26 | 0.916667 |
| E2F4 | 0.916667 |
| EBI3 | 0.916667 |
| ECH1 | 0.916667 |
| EDC3 | 0.916667 |
| EFCAB7 | 0.916667 |
| EFR3A | 0.916667 |
| ELMOD3 | 0.916667 |
| ELMSAN1 | 0.916667 |
| ENPP6 | 0.916667 |
| EOGT | 0.916667 |
| EP400 | 0.916667 |
| EP400NL | 0.916667 |
| EPC2 | 0.916667 |
| EPCAM | 0.916667 |
| EPHA6 | 0.916667 |
| ERCC6 | 0.916667 |
| ERCC6L2 | 0.916667 |
| ERV3.1 | 0.916667 |
| ESPNL | 0.916667 |
| ETV6 | 0.916667 |
| EVA1B | 0.916667 |
| EVI5L | 0.916667 |
| FABP7 | 0.916667 |
| FAM160B2 | 0.916667 |
| FAM161A | 0.916667 |
| FAM166A | 0.916667 |
| FAM169B | 0.916667 |
| FAM174A | 0.916667 |
| FAM199X | 0.916667 |
| FAM206A | 0.916667 |
| FAM216A | 0.916667 |
| FAM46B | 0.916667 |
| FAM71B | 0.916667 |
| FAM8A1 | 0.916667 |
| FAP | 0.916667 |
| FAR1 | 0.916667 |
| FBN3 | 0.916667 |
| FBXL14 | 0.916667 |
| FBXO28 | 0.916667 |
| FBXW11 | 0.916667 |
| FCN2 | 0.916667 |
| FCRLB | 0.916667 |
| FGF4 | 0.916667 |
| FHDC1 | 0.916667 |
| FKBP9 | 0.916667 |
| FLG | 0.916667 |
| FLI1 | 0.916667 |
| FLJ25694 | 0.916667 |
| FLJ31713 | 0.916667 |
| FLJ31715 | 0.916667 |
| FLJ33360 | 0.916667 |
| FLJ35934 | 0.916667 |
| FLJ43763 | 0.916667 |
| FNDC3A | 0.916667 |
| FOLR1 | 0.916667 |
| FOXA2 | 0.916667 |
| FOXD3 | 0.916667 |
| FOXE1 | 0.916667 |
| FOXN1 | 0.916667 |
| FRA10AC1 | 0.916667 |
| FSCN2 | 0.916667 |
| FTH1 | 0.916667 |
| FUT7 | 0.916667 |
| FZD5 | 0.916667 |
| FZD8 | 0.916667 |
| GAB1 | 0.916667 |
| GABRA2 | 0.916667 |
| GABRG1 | 0.916667 |
| GABRQ | 0.916667 |
| GAK | 0.916667 |
| GALC | 0.916667 |
| GALK1 | 0.916667 |
| GALNT9 | 0.916667 |
| GBX1 | 0.916667 |
| GDNF | 0.916667 |
| GJA10 | 0.916667 |
| GJB1 | 0.916667 |
| GLMN | 0.916667 |
| GLS2 | 0.916667 |
| GMEB1 | 0.916667 |
| GNPDA1 | 0.916667 |
| GNRHR2 | 0.916667 |
| GOLGA2P5 | 0.916667 |
| GOLM1 | 0.916667 |
| GORAB | 0.916667 |
| GP2 | 0.916667 |
| GPR115 | 0.916667 |
| GPR132 | 0.916667 |
| GPR137C | 0.916667 |
| GPR146 | 0.916667 |
| GPR15 | 0.916667 |
| GPR155 | 0.916667 |
| GPR158 | 0.916667 |
| GPR171 | 0.916667 |
| GPR25 | 0.916667 |
| GPR78 | 0.916667 |
| GPRIN2 | 0.916667 |
| GPX2 | 0.916667 |
| GPX6 | 0.916667 |
| GRIA1 | 0.916667 |
| GRIN2B | 0.916667 |
| GRM1 | 0.916667 |
| GSE1 | 0.916667 |
| GSG1L | 0.916667 |
| GSTA5 | 0.916667 |
| GTF3A | 0.916667 |
| GTF3C2 | 0.916667 |
| GTPBP4 | 0.916667 |
| GUCY2D | 0.916667 |
| GZMB | 0.916667 |
| HAS1 | 0.916667 |
| HAUS6 | 0.916667 |
| HCAR3 | 0.916667 |
| HCFC2 | 0.916667 |
| HCN4 | 0.916667 |
| HEATR5A | 0.916667 |
| HELB | 0.916667 |
| HELZ | 0.916667 |
| HES3 | 0.916667 |
| HEXDC | 0.916667 |
| HHLA3 | 0.916667 |
| HILPDA | 0.916667 |
| HINT2 | 0.916667 |
| HIPK1 | 0.916667 |
| HIRIP3 | 0.916667 |
| HIST1H2BF | 0.916667 |
| HIST1H2BK | 0.916667 |
| HIST1H4E | 0.916667 |
| HMHA1 | 0.916667 |
| HMMR | 0.916667 |
| HOXB8 | 0.916667 |
| HOXD12 | 0.916667 |
| HPN.AS1 | 0.916667 |
| HPS1 | 0.916667 |
| HRK | 0.916667 |
| HSDL1 | 0.916667 |
| HSH2D | 0.916667 |
| HSPB6 | 0.916667 |
| HTR6 | 0.916667 |
| HVCN1 | 0.916667 |
| ICAM3 | 0.916667 |
| IDI2 | 0.916667 |
| IFFO2 | 0.916667 |
| IFI6 | 0.916667 |
| IGFN1 | 0.916667 |
| IGSF21 | 0.916667 |
| IL1F10 | 0.916667 |
| IL1RL1 | 0.916667 |
| IL2RB | 0.916667 |
| IMPA1 | 0.916667 |
| IMPACT | 0.916667 |
| INCA1 | 0.916667 |
| INPP5K | 0.916667 |
| INTS5 | 0.916667 |
| INTS7 | 0.916667 |
| INTS8 | 0.916667 |
| IQGAP3 | 0.916667 |
| IRF3 | 0.916667 |
| IRF4 | 0.916667 |
| IRF5 | 0.916667 |
| ISL1 | 0.916667 |
| ISL2 | 0.916667 |
| ITGAV | 0.916667 |
| ITIH2 | 0.916667 |
| ITIH5 | 0.916667 |
| IZUMO1 | 0.916667 |
| JADE2 | 0.916667 |
| KATNA1 | 0.916667 |
| KBTBD3 | 0.916667 |
| KCNE4 | 0.916667 |
| KCNF1 | 0.916667 |
| KCNJ13 | 0.916667 |
| KCNJ16 | 0.916667 |
| KCNJ8 | 0.916667 |
| KCNJ9 | 0.916667 |
| KDM5A | 0.916667 |
| KIAA0513 | 0.916667 |
| KIAA1377 | 0.916667 |
| KIAA1524 | 0.916667 |
| KIAA1683 | 0.916667 |
| KIAA1751 | 0.916667 |
| KIAA2018 | 0.916667 |
| KIAA2026 | 0.916667 |
| KIRREL2 | 0.916667 |
| KLF15 | 0.916667 |
| KLHDC4 | 0.916667 |
| KLHDC7B | 0.916667 |
| KLHL14 | 0.916667 |
| KLHL15 | 0.916667 |
| KLHL30 | 0.916667 |
| KRT17 | 0.916667 |
| KRT71 | 0.916667 |
| KRT73 | 0.916667 |
| KRT75 | 0.916667 |
| KRT78 | 0.916667 |
| KRT82 | 0.916667 |
| KRTAP13.1 | 0.916667 |
| KRTAP19.5 | 0.916667 |
| KRTAP20.4 | 0.916667 |
| KRTAP3.3 | 0.916667 |
| KRTAP4.1 | 0.916667 |
| KRTAP6.1 | 0.916667 |
| KRTAP7.1 | 0.916667 |
| KYNU | 0.916667 |
| L3MBTL2 | 0.916667 |
| LACC1 | 0.916667 |
| LAMTOR1 | 0.916667 |
| LARS2 | 0.916667 |
| LATS1 | 0.916667 |
| LCA10 | 0.916667 |
| LCE2B | 0.916667 |
| LCE2D | 0.916667 |
| LCN1 | 0.916667 |
| LCN12 | 0.916667 |
| LGALS2 | 0.916667 |
| LGI2 | 0.916667 |
| LGR6 | 0.916667 |
| LHX6 | 0.916667 |
| LIMS2 | 0.916667 |
| LINC00238 | 0.916667 |
| LINC00242 | 0.916667 |
| LINC00282 | 0.916667 |
| LINC00301 | 0.916667 |
| LINC00310 | 0.916667 |
| LINC00518 | 0.916667 |
| LINC00597 | 0.916667 |
| LINC00885 | 0.916667 |
| LINC01123 | 0.916667 |
| LINC01140 | 0.916667 |
| LINS | 0.916667 |
| LIPJ | 0.916667 |
| LOC100128644 | 0.916667 |
| LOC100129307 | 0.916667 |
| LOC100129395 | 0.916667 |
| LOC100129455 | 0.916667 |
| LOC100129476 | 0.916667 |
| LOC100129648 | 0.916667 |
| LOC100130071 | 0.916667 |
| LOC100130433 | 0.916667 |
| LOC100134317 | 0.916667 |
| LOC100288160 | 0.916667 |
| LOC101927181 | 0.916667 |
| LOC283693 | 0.916667 |
| LOC375196 | 0.916667 |
| LOC401010 | 0.916667 |
| LOC401127 | 0.916667 |
| LOC403323 | 0.916667 |
| LOC645188 | 0.916667 |
| LOC646976 | 0.916667 |
| LOC728690 | 0.916667 |
| LOC93463 | 0.916667 |
| LOXL4 | 0.916667 |
| LPAR2 | 0.916667 |
| LPAR3 | 0.916667 |
| LPPR1 | 0.916667 |
| LRIF1 | 0.916667 |
| LRIT3 | 0.916667 |
| LRP12 | 0.916667 |
| LRP5 | 0.916667 |
| LRPAP1 | 0.916667 |
| LRRC3B | 0.916667 |
| LRRC48 | 0.916667 |
| LRRN3 | 0.916667 |
| LSP1 | 0.916667 |
| LUZP2 | 0.916667 |
| LUZP4 | 0.916667 |
| LY96 | 0.916667 |
| LYRM4 | 0.916667 |
| M1AP | 0.916667 |
| MACROD2 | 0.916667 |
| MAGEA4 | 0.916667 |
| MAGEB5 | 0.916667 |
| MAGEE2 | 0.916667 |
| MAMDC2 | 0.916667 |
| MAML2 | 0.916667 |
| MANF | 0.916667 |
| MAP7D1 | 0.916667 |
| 11-Mar | 0.916667 |
| MARK1 | 0.916667 |
| MARVELD2 | 0.916667 |
| MAST1 | 0.916667 |
| MAST2 | 0.916667 |
| MATK | 0.916667 |
| MATN1 | 0.916667 |
| MBOAT2 | 0.916667 |
| MBP | 0.916667 |
| MED11 | 0.916667 |
| MED13L | 0.916667 |
| MED15 | 0.916667 |
| MEF2D | 0.916667 |
| MEGF10 | 0.916667 |
| MEGF9 | 0.916667 |
| MEIG1 | 0.916667 |
| MESDC1 | 0.916667 |
| METTL6 | 0.916667 |
| METTL7A | 0.916667 |
| MFNG | 0.916667 |
| MFSD4 | 0.916667 |
| MFSD6L | 0.916667 |
| MGP | 0.916667 |
| MIR107 | 0.916667 |
| MIR124.3 | 0.916667 |
| MIR125B1 | 0.916667 |
| MIR15B | 0.916667 |
| MIR365A | 0.916667 |
| MIR375 | 0.916667 |
| MIR503HG | 0.916667 |
| MIS18BP1 | 0.916667 |
| MIXL1 | 0.916667 |
| MKNK1 | 0.916667 |
| MLLT11 | 0.916667 |
| MLXIPL | 0.916667 |
| MMP16 | 0.916667 |
| MRE11A | 0.916667 |
| MSGN1 | 0.916667 |
| MSH5 | 0.916667 |
| MSL1 | 0.916667 |
| MT1L | 0.916667 |
| MTMR14 | 0.916667 |
| MTMR7 | 0.916667 |
| MTMR9LP | 0.916667 |
| MTSS1 | 0.916667 |
| MUC19 | 0.916667 |
| MUT | 0.916667 |
| MYH15 | 0.916667 |
| MYO15B | 0.916667 |
| MYOM1 | 0.916667 |
| MYOZ1 | 0.916667 |
| N4BP1 | 0.916667 |
| NAA40 | 0.916667 |
| NAALAD2 | 0.916667 |
| NAF1 | 0.916667 |
| NARF | 0.916667 |
| NASP | 0.916667 |
| NCAPG2 | 0.916667 |
| NCDN | 0.916667 |
| NCF2 | 0.916667 |
| NCOA3 | 0.916667 |
| NCR1 | 0.916667 |
| NCR2 | 0.916667 |
| NCS1 | 0.916667 |
| NDC1 | 0.916667 |
| NDFIP1 | 0.916667 |
| NDNL2 | 0.916667 |
| NDST4 | 0.916667 |
| NDUFA13 | 0.916667 |
| NEBL | 0.916667 |
| NECAP1 | 0.916667 |
| NENF | 0.916667 |
| NFX1 | 0.916667 |
| NFXL1 | 0.916667 |
| NKTR | 0.916667 |
| NLRP6 | 0.916667 |
| NLRP9 | 0.916667 |
| NMNAT1 | 0.916667 |
| NMNAT2 | 0.916667 |
| NOP10 | 0.916667 |
| NOTCH1 | 0.916667 |
| NOXRED1 | 0.916667 |
| NPTXR | 0.916667 |
| NR2E1 | 0.916667 |
| NRTN | 0.916667 |
| NSAP11 | 0.916667 |
| NUDCD3 | 0.916667 |
| NUDT1 | 0.916667 |
| NUDT11 | 0.916667 |
| NUDT9 | 0.916667 |
| NXF1 | 0.916667 |
| NXPE2 | 0.916667 |
| OBSL1 | 0.916667 |
| ODF2L | 0.916667 |
| OPN1SW | 0.916667 |
| OPN5 | 0.916667 |
| OR11H4 | 0.916667 |
| OR14J1 | 0.916667 |
| OR1A2 | 0.916667 |
| OR1B1 | 0.916667 |
| OR1K1 | 0.916667 |
| OR2B2 | 0.916667 |
| OR2T1 | 0.916667 |
| OR2V1 | 0.916667 |
| OR4A15 | 0.916667 |
| OR4D2 | 0.916667 |
| OR4D9 | 0.916667 |
| OR4S1 | 0.916667 |
| OR52A4 | 0.916667 |
| OR52D1 | 0.916667 |
| OR56A3 | 0.916667 |
| OR5AP2 | 0.916667 |
| OR5M10 | 0.916667 |
| OR6K2 | 0.916667 |
| OR6P1 | 0.916667 |
| OR8H1 | 0.916667 |
| ORC3 | 0.916667 |
| OSGIN1 | 0.916667 |
| OSTF1 | 0.916667 |
| OTUB2 | 0.916667 |
| OVCH1 | 0.916667 |
| OVOL3 | 0.916667 |
| PABPC1 | 0.916667 |
| PACRG | 0.916667 |
| PADI6 | 0.916667 |
| PAG1 | 0.916667 |
| PAPOLB | 0.916667 |
| PAQR7 | 0.916667 |
| PASK | 0.916667 |
| PCAT4 | 0.916667 |
| PCBP4 | 0.916667 |
| PCDH8 | 0.916667 |
| PCGF3 | 0.916667 |
| PCGF5 | 0.916667 |
| PCID2 | 0.916667 |
| PCSK5 | 0.916667 |
| PDCL | 0.916667 |
| PDF | 0.916667 |
| PDHA2 | 0.916667 |
| PDIK1L | 0.916667 |
| PEX5 | 0.916667 |
| PHOSPHO1 | 0.916667 |
| PHTF2 | 0.916667 |
| PI3 | 0.916667 |
| PIGA | 0.916667 |
| PIH1D2 | 0.916667 |
| PIK3R6 | 0.916667 |
| PITPNM1 | 0.916667 |
| PKDCC | 0.916667 |
| PLAC8 | 0.916667 |
| PLEKHG7 | 0.916667 |
| PLS3 | 0.916667 |
| PNPLA5 | 0.916667 |
| POLR2H | 0.916667 |
| POU6F2 | 0.916667 |
| PPA2 | 0.916667 |
| PPIEL | 0.916667 |
| PPM1A | 0.916667 |
| PPM1B | 0.916667 |
| PPM1G | 0.916667 |
| PPM1J | 0.916667 |
| PPP1R16B | 0.916667 |
| PPP3R1 | 0.916667 |
| PPP4R4 | 0.916667 |
| PPY2 | 0.916667 |
| PRIMA1 | 0.916667 |
| PRLH | 0.916667 |
| PRLHR | 0.916667 |
| PRMT7 | 0.916667 |
| PRND | 0.916667 |
| PROSC | 0.916667 |
| PROZ | 0.916667 |
| PRR34 | 0.916667 |
| PRRT2 | 0.916667 |
| PSMA2 | 0.916667 |
| PTCD2 | 0.916667 |
| PTGIR | 0.916667 |
| PTRHD1 | 0.916667 |
| PUS7 | 0.916667 |
| PVRL2 | 0.916667 |
| PYGO1 | 0.916667 |
| RAB19 | 0.916667 |
| RAB29 | 0.916667 |
| RAB3IP | 0.916667 |
| RAB40C | 0.916667 |
| RADIL | 0.916667 |
| RAET1G | 0.916667 |
| RAP1GAP | 0.916667 |
| RBCK1 | 0.916667 |
| RBFA | 0.916667 |
| RBM10 | 0.916667 |
| RBM19 | 0.916667 |
| RBM24 | 0.916667 |
| RBM28 | 0.916667 |
| RBM41 | 0.916667 |
| RBM43 | 0.916667 |
| RBMY1A3P | 0.916667 |
| RBMY1J | 0.916667 |
| RCC1 | 0.916667 |
| RCOR3 | 0.916667 |
| RCVRN | 0.916667 |
| RDH12 | 0.916667 |
| RELN | 0.916667 |
| RETN | 0.916667 |
| RFX3 | 0.916667 |
| RFX4 | 0.916667 |
| RGS19 | 0.916667 |
| RHBDL2 | 0.916667 |
| RHBDL3 | 0.916667 |
| RHCG | 0.916667 |
| RHO | 0.916667 |
| RHOBTB3 | 0.916667 |
| RIMKLB | 0.916667 |
| RIMS1 | 0.916667 |
| RING1 | 0.916667 |
| RIPK2 | 0.916667 |
| RLBP1 | 0.916667 |
| RMND5B | 0.916667 |
| RNASEH2A | 0.916667 |
| RNASEH2B | 0.916667 |
| RNF133 | 0.916667 |
| RNF141 | 0.916667 |
| RNF146 | 0.916667 |
| RNF19B | 0.916667 |
| RNGTT | 0.916667 |
| RNPS1 | 0.916667 |
| RNU5E.1 | 0.916667 |
| RNVU1.10 | 0.916667 |
| RNVU1.8 | 0.916667 |
| ROGDI | 0.916667 |
| RPAP1 | 0.916667 |
| RPL13AP17 | 0.916667 |
| RPL22 | 0.916667 |
| RPP14 | 0.916667 |
| RPS14 | 0.916667 |
| RPS2P32 | 0.916667 |
| RRN3 | 0.916667 |
| RSG1 | 0.916667 |
| RSPH9 | 0.916667 |
| RTN4RL1 | 0.916667 |
| RUNDC1 | 0.916667 |
| RXFP2 | 0.916667 |
| S100A1 | 0.916667 |
| SALL4 | 0.916667 |
| SBNO2 | 0.916667 |
| SCARNA5 | 0.916667 |
| SCARNA9 | 0.916667 |
| SCGB1D2 | 0.916667 |
| SCML4 | 0.916667 |
| SCN1B | 0.916667 |
| SCYL3 | 0.916667 |
| SEC16B | 0.916667 |
| SEMA4G | 0.916667 |
| 5-Sep | 0.916667 |
| SEZ6 | 0.916667 |
| SFT2D2 | 0.916667 |
| SFXN5 | 0.916667 |
| SH3BGRL3 | 0.916667 |
| SH3BP2 | 0.916667 |
| SHANK1 | 0.916667 |
| SHISA9 | 0.916667 |
| SHQ1 | 0.916667 |
| SIGLEC7 | 0.916667 |
| SIM2 | 0.916667 |
| SIRT6 | 0.916667 |
| SKAP2 | 0.916667 |
| SKIDA1 | 0.916667 |
| SKIV2L | 0.916667 |
| SLC17A9 | 0.916667 |
| SLC1A6 | 0.916667 |
| SLC22A14 | 0.916667 |
| SLC22A25 | 0.916667 |
| SLC22A4 | 0.916667 |
| SLC25A17 | 0.916667 |
| SLC25A32 | 0.916667 |
| SLC25A34 | 0.916667 |
| SLC25A53 | 0.916667 |
| SLC26A3 | 0.916667 |
| SLC29A2 | 0.916667 |
| SLC2A10 | 0.916667 |
| SLC2A3 | 0.916667 |
| SLC2A4 | 0.916667 |
| SLC35A3 | 0.916667 |
| SLC39A6 | 0.916667 |
| SLC44A2 | 0.916667 |
| SLC47A1 | 0.916667 |
| SLC4A4 | 0.916667 |
| SLC9A3R2 | 0.916667 |
| SLC9A4 | 0.916667 |
| SLC9A8 | 0.916667 |
| SLFN12 | 0.916667 |
| SLFNL1 | 0.916667 |
| SMAD5 | 0.916667 |
| SMARCA4 | 0.916667 |
| SMCHD1 | 0.916667 |
| SMTNL2 | 0.916667 |
| SMUG1 | 0.916667 |
| SMURF1 | 0.916667 |
| SNAP47 | 0.916667 |
| SNORA23 | 0.916667 |
| SNORA28 | 0.916667 |
| SNORA29 | 0.916667 |
| SNORA61 | 0.916667 |
| SNORA70C | 0.916667 |
| SNORA71A | 0.916667 |
| SNORA74A | 0.916667 |
| SNORA7B | 0.916667 |
| SNORA80A | 0.916667 |
| SNORD102 | 0.916667 |
| SNORD115.31 | 0.916667 |
| SNORD115.33 | 0.916667 |
| SNORD115.38 | 0.916667 |
| SNORD116.24 | 0.916667 |
| SNORD1B | 0.916667 |
| SNORD22 | 0.916667 |
| SNORD36C | 0.916667 |
| SNORD41 | 0.916667 |
| SNORD4B | 0.916667 |
| SNORD58A | 0.916667 |
| SNORD59B | 0.916667 |
| SNRPA | 0.916667 |
| SNX21 | 0.916667 |
| SNX29P2 | 0.916667 |
| SOCS3 | 0.916667 |
| SORBS2 | 0.916667 |
| SORL1 | 0.916667 |
| SOS1 | 0.916667 |
| SOS2 | 0.916667 |
| SOWAHC | 0.916667 |
| SOX15 | 0.916667 |
| SOX7 | 0.916667 |
| SPANXN2 | 0.916667 |
| SPANXN4 | 0.916667 |
| SPATA33 | 0.916667 |
| SPATC1L | 0.916667 |
| SPATS2L | 0.916667 |
| SPG11 | 0.916667 |
| SPHK1 | 0.916667 |
| SPINT3 | 0.916667 |
| SPIRE2 | 0.916667 |
| SPPL2B | 0.916667 |
| SPRR2B | 0.916667 |
| SREK1 | 0.916667 |
| SRRD | 0.916667 |
| SRRM4 | 0.916667 |
| SSH3 | 0.916667 |
| SSTR5.AS1 | 0.916667 |
| ST14 | 0.916667 |
| STK24 | 0.916667 |
| STX10 | 0.916667 |
| SUFU | 0.916667 |
| SUMF2 | 0.916667 |
| SUPT20H | 0.916667 |
| SWT1 | 0.916667 |
| SYCE1 | 0.916667 |
| SYCE2 | 0.916667 |
| TAF1C | 0.916667 |
| TAPBP | 0.916667 |
| TBC1D10C | 0.916667 |
| TBC1D25 | 0.916667 |
| TBCEL | 0.916667 |
| TCEAL3 | 0.916667 |
| TCEANC | 0.916667 |
| TEAD2 | 0.916667 |
| TECRL | 0.916667 |
| TEN1 | 0.916667 |
| TET1 | 0.916667 |
| TEX19 | 0.916667 |
| TFEB | 0.916667 |
| TGFBR2 | 0.916667 |
| THAP1 | 0.916667 |
| THOC1 | 0.916667 |
| TIGD4 | 0.916667 |
| TJAP1 | 0.916667 |
| TLE2 | 0.916667 |
| TLL2 | 0.916667 |
| TM4SF19 | 0.916667 |
| TM9SF2 | 0.916667 |
| TMA7 | 0.916667 |
| TMC4 | 0.916667 |
| TMCC1 | 0.916667 |
| TMED6 | 0.916667 |
| TMEM145 | 0.916667 |
| TMEM183B | 0.916667 |
| TMEM201 | 0.916667 |
| TMEM203 | 0.916667 |
| TMEM229A | 0.916667 |
| TMEM231 | 0.916667 |
| TMEM234 | 0.916667 |
| TMEM31 | 0.916667 |
| TMEM40 | 0.916667 |
| TMEM42 | 0.916667 |
| TMEM87B | 0.916667 |
| TMEM91 | 0.916667 |
| TMEM98 | 0.916667 |
| TMTC4 | 0.916667 |
| TOMM20 | 0.916667 |
| TOPBP1 | 0.916667 |
| TOR3A | 0.916667 |
| TOR4A | 0.916667 |
| TPPP2 | 0.916667 |
| TRAF3IP3 | 0.916667 |
| TRAF4 | 0.916667 |
| TRAPPC1 | 0.916667 |
| TRERF1 | 0.916667 |
| TRIM65 | 0.916667 |
| TRIM69 | 0.916667 |
| TRMT10A | 0.916667 |
| TRPC3 | 0.916667 |
| TSNARE1 | 0.916667 |
| TTC18 | 0.916667 |
| TXNL4A | 0.916667 |
| TXNL4B | 0.916667 |
| TYMP | 0.916667 |
| TYW1B | 0.916667 |
| UBD | 0.916667 |
| UBE2D1 | 0.916667 |
| UBE2D2 | 0.916667 |
| UBE2O | 0.916667 |
| UBN1 | 0.916667 |
| UBR7 | 0.916667 |
| UG0898H09 | 0.916667 |
| UGGT1 | 0.916667 |
| ULK1 | 0.916667 |
| UNC13D | 0.916667 |
| UNC5A | 0.916667 |
| UNC5D | 0.916667 |
| UNCX | 0.916667 |
| UQCRH | 0.916667 |
| USHBP1 | 0.916667 |
| USP40 | 0.916667 |
| USP54 | 0.916667 |
| USP8 | 0.916667 |
| UTRN | 0.916667 |
| VAT1L | 0.916667 |
| VIP | 0.916667 |
| VN1R4 | 0.916667 |
| VPS26B | 0.916667 |
| VPS37B | 0.916667 |
| VPS53 | 0.916667 |
| VRK1 | 0.916667 |
| WASF1 | 0.916667 |
| WBSCR28 | 0.916667 |
| WDR20 | 0.916667 |
| WDR53 | 0.916667 |
| WDR93 | 0.916667 |
| WNT2B | 0.916667 |
| WRAP73 | 0.916667 |
| WSCD1 | 0.916667 |
| XPO1 | 0.916667 |
| XRRA1 | 0.916667 |
| YEATS2 | 0.916667 |
| YEATS4 | 0.916667 |
| YY2 | 0.916667 |
| ZAP70 | 0.916667 |
| ZBTB16 | 0.916667 |
| ZBTB39 | 0.916667 |
| ZCCHC24 | 0.916667 |
| ZCCHC4 | 0.916667 |
| ZER1 | 0.916667 |
| ZFAND2A | 0.916667 |
| ZFP37 | 0.916667 |
| ZFYVE27 | 0.916667 |
| ZMYM1 | 0.916667 |
| ZMYM3 | 0.916667 |
| ZMYND15 | 0.916667 |
| ZNF136 | 0.916667 |
| ZNF160 | 0.916667 |
| ZNF181 | 0.916667 |
| ZNF207 | 0.916667 |
| ZNF222 | 0.916667 |
| ZNF300 | 0.916667 |
| ZNF300P1 | 0.916667 |
| ZNF33B | 0.916667 |
| ZNF41 | 0.916667 |
| ZNF418 | 0.916667 |
| ZNF443 | 0.916667 |
| ZNF45 | 0.916667 |
| ZNF469 | 0.916667 |
| ZNF503 | 0.916667 |
| ZNF525 | 0.916667 |
| ZNF529 | 0.916667 |
| ZNF550 | 0.916667 |
| ZNF559 | 0.916667 |
| ZNF560 | 0.916667 |
| ZNF566 | 0.916667 |
| ZNF575 | 0.916667 |
| ZNF655 | 0.916667 |
| ZNF675 | 0.916667 |
| ZNF681 | 0.916667 |
| ZNF687 | 0.916667 |
| ZNF706 | 0.916667 |
| ZNF738 | 0.916667 |
| ZNF831 | 0.916667 |
| ZNF860 | 0.916667 |
| ZNRF3 | 0.916667 |
| ZSCAN22 | 0.916667 |
| ZSCAN5A | 0.916667 |
| ZXDC | 0.916667 |
| AANAT | 0.875 |
| AAR2 | 0.875 |
| ACSF2 | 0.875 |
| ACTR1B | 0.875 |
| ADAM21 | 0.875 |
| ADAM30 | 0.875 |
| ADCY4 | 0.875 |
| AGGF1 | 0.875 |
| AJAP1 | 0.875 |
| AKAP1 | 0.875 |
| AKAP9 | 0.875 |
| ALKBH1 | 0.875 |
| ANAPC16 | 0.875 |
| ANGEL2 | 0.875 |
| ANGPTL1 | 0.875 |
| ANKRD24 | 0.875 |
| ANKRD30A | 0.875 |
| ANKRD30BL | 0.875 |
| ANKRD35 | 0.875 |
| AP2A2 | 0.875 |
| AP3S1 | 0.875 |
| APBA1 | 0.875 |
| APC | 0.875 |
| APLP1 | 0.875 |
| APOBEC3G | 0.875 |
| ARSG | 0.875 |
| ARSI | 0.875 |
| ART5 | 0.875 |
| AS3MT | 0.875 |
| ATP13A5 | 0.875 |
| ATP5G2 | 0.875 |
| AVPR2 | 0.875 |
| BAG5 | 0.875 |
| BAIAP2L1 | 0.875 |
| BATF3 | 0.875 |
| BCL2L10 | 0.875 |
| BECN1 | 0.875 |
| BICC1 | 0.875 |
| BMP6 | 0.875 |
| BPESC1 | 0.875 |
| BPIFB1 | 0.875 |
| BRAF | 0.875 |
| BRPF1 | 0.875 |
| BTN1A1 | 0.875 |
| C11orf54 | 0.875 |
| C14orf37 | 0.875 |
| C16orf58 | 0.875 |
| C16orf92 | 0.875 |
| C17orf105 | 0.875 |
| C17orf75 | 0.875 |
| C1orf127 | 0.875 |
| C1orf27 | 0.875 |
| C1orf54 | 0.875 |
| C1QL1 | 0.875 |
| C20orf195 | 0.875 |
| C21orf58 | 0.875 |
| C3orf80 | 0.875 |
| C5orf28 | 0.875 |
| C5orf51 | 0.875 |
| C5orf60 | 0.875 |
| CACNG2 | 0.875 |
| CAMLG | 0.875 |
| CAPN6 | 0.875 |
| CBLN3 | 0.875 |
| CCDC125 | 0.875 |
| CCL3L3 | 0.875 |
| CCRN4L | 0.875 |
| CD1D | 0.875 |
| CDC34 | 0.875 |
| CDK5RAP3 | 0.875 |
| CDNF | 0.875 |
| CEACAM16 | 0.875 |
| CEACAM3 | 0.875 |
| CENPO | 0.875 |
| CHAC2 | 0.875 |
| CHL1 | 0.875 |
| CHMP4C | 0.875 |
| CHRAC1 | 0.875 |
| CISD2 | 0.875 |
| CITED4 | 0.875 |
| CLASRP | 0.875 |
| CLK2 | 0.875 |
| CLYBL | 0.875 |
| CNN3 | 0.875 |
| CNNM4 | 0.875 |
| CNOT4 | 0.875 |
| COL23A1 | 0.875 |
| COL4A1 | 0.875 |
| CPSF4L | 0.875 |
| CPT1C | 0.875 |
| CRYBB1 | 0.875 |
| CSF3R | 0.875 |
| CSRNP2 | 0.875 |
| CST6 | 0.875 |
| CTBP1.AS2 | 0.875 |
| CTDP1 | 0.875 |
| CTDSPL2 | 0.875 |
| CTSF | 0.875 |
| CUL4A | 0.875 |
| CUL4B | 0.875 |
| CYB5A | 0.875 |
| CYP2S1 | 0.875 |
| CYP39A1 | 0.875 |
| DAAM2 | 0.875 |
| DCP1B | 0.875 |
| DDN | 0.875 |
| DDO | 0.875 |
| DDX50 | 0.875 |
| DEFB126 | 0.875 |
| DHX15 | 0.875 |
| DIS3L | 0.875 |
| DNAAF3 | 0.875 |
| DNAI2 | 0.875 |
| DNASE2 | 0.875 |
| DOCK11 | 0.875 |
| DOPEY1 | 0.875 |
| DPY19L2P2 | 0.875 |
| DRD5 | 0.875 |
| DTHD1 | 0.875 |
| DYX1C1 | 0.875 |
| DZIP1L | 0.875 |
| EIF2B5 | 0.875 |
| ELMOD1 | 0.875 |
| ENSA | 0.875 |
| ENTPD2 | 0.875 |
| ENTPD4 | 0.875 |
| EOMES | 0.875 |
| ERBB4 | 0.875 |
| ERCC5 | 0.875 |
| ERVFC1.1 | 0.875 |
| EXOSC9 | 0.875 |
| EXTL2 | 0.875 |
| F7 | 0.875 |
| FAM151B | 0.875 |
| FAM154A | 0.875 |
| FAM160A2 | 0.875 |
| FAM214A | 0.875 |
| FAM222B | 0.875 |
| FAM63A | 0.875 |
| FAM84A | 0.875 |
| FBXL8 | 0.875 |
| FBXO25 | 0.875 |
| FBXO47 | 0.875 |
| FBXW2 | 0.875 |
| FDCSP | 0.875 |
| FECH | 0.875 |
| FLII | 0.875 |
| FLJ11710 | 0.875 |
| FLJ22184 | 0.875 |
| FLRT3 | 0.875 |
| FOXD2 | 0.875 |
| FOXO4 | 0.875 |
| FOXQ1 | 0.875 |
| G0S2 | 0.875 |
| GALNT18 | 0.875 |
| GGPS1 | 0.875 |
| GJC3 | 0.875 |
| GLIS1 | 0.875 |
| GNA14 | 0.875 |
| GOLGB1 | 0.875 |
| GP1BB | 0.875 |
| GPR152 | 0.875 |
| GPR97 | 0.875 |
| GRHL2 | 0.875 |
| GRID1 | 0.875 |
| GSTA3 | 0.875 |
| GSTA4 | 0.875 |
| GTPBP8 | 0.875 |
| GYPC | 0.875 |
| HBQ1 | 0.875 |
| HDAC7 | 0.875 |
| HELQ | 0.875 |
| HES4 | 0.875 |
| HES7 | 0.875 |
| HILS1 | 0.875 |
| HIST3H2BB | 0.875 |
| HLTF | 0.875 |
| HNF1A | 0.875 |
| HOMER2 | 0.875 |
| HOXA1 | 0.875 |
| HOXB9 | 0.875 |
| HOXC12 | 0.875 |
| HOXD10 | 0.875 |
| HPCAL4 | 0.875 |
| HSD3BP4 | 0.875 |
| IDUA | 0.875 |
| IFNGR1 | 0.875 |
| IGBP1 | 0.875 |
| IL36RN | 0.875 |
| IL6 | 0.875 |
| INGX | 0.875 |
| INHBE | 0.875 |
| INO80 | 0.875 |
| INTS6 | 0.875 |
| IP6K1 | 0.875 |
| IPO8 | 0.875 |
| IRAK4 | 0.875 |
| ISG20L2 | 0.875 |
| ITGB8 | 0.875 |
| JAKMIP2 | 0.875 |
| KANK1 | 0.875 |
| KCNK12 | 0.875 |
| KCTD21 | 0.875 |
| KDM6B | 0.875 |
| KIF2C | 0.875 |
| KIF9 | 0.875 |
| KLF1 | 0.875 |
| KLHDC7A | 0.875 |
| KLHL25 | 0.875 |
| KRT16P2 | 0.875 |
| KRTAP10.7 | 0.875 |
| KRTAP5.5 | 0.875 |
| KRTAP6.3 | 0.875 |
| LGALS13 | 0.875 |
| LHCGR | 0.875 |
| LHX1 | 0.875 |
| LINC00268 | 0.875 |
| LINC00598 | 0.875 |
| LINC00652 | 0.875 |
| LINC00851 | 0.875 |
| LINC01126 | 0.875 |
| LMAN1L | 0.875 |
| LOC100130744 | 0.875 |
| LOC100288208 | 0.875 |
| LOC100653515 | 0.875 |
| LOC202181 | 0.875 |
| LOC284023 | 0.875 |
| LOC401433 | 0.875 |
| LOC650157 | 0.875 |
| LRG1 | 0.875 |
| LRR1 | 0.875 |
| LRRC24 | 0.875 |
| LRRC37A6P | 0.875 |
| LRRC58 | 0.875 |
| LRRFIP1 | 0.875 |
| LSM10 | 0.875 |
| LSM6 | 0.875 |
| LY6G6C | 0.875 |
| LY6G6D | 0.875 |
| MACF1 | 0.875 |
| MAK16 | 0.875 |
| MAP1LC3A | 0.875 |
| 1-Mar | 0.875 |
| MAT2B | 0.875 |
| MATN4 | 0.875 |
| MBD3 | 0.875 |
| MBD5 | 0.875 |
| MCEE | 0.875 |
| MEG3 | 0.875 |
| METTL7B | 0.875 |
| MFHAS1 | 0.875 |
| MFSD2B | 0.875 |
| MIR126 | 0.875 |
| MIR128.2 | 0.875 |
| MIR132 | 0.875 |
| MIR223 | 0.875 |
| MIR495 | 0.875 |
| MLNR | 0.875 |
| MMP28 | 0.875 |
| MON2 | 0.875 |
| MTL5 | 0.875 |
| MTMR2 | 0.875 |
| MUC16 | 0.875 |
| MVK | 0.875 |
| MYOM3 | 0.875 |
| NAALADL2 | 0.875 |
| NAGS | 0.875 |
| NCF1 | 0.875 |
| NEDD4 | 0.875 |
| NEK11 | 0.875 |
| NEXN | 0.875 |
| NFE2 | 0.875 |
| NGEF | 0.875 |
| NKAIN4 | 0.875 |
| NPPC | 0.875 |
| NR1D2 | 0.875 |
| NRGN | 0.875 |
| NRK | 0.875 |
| NSA2 | 0.875 |
| NT5C | 0.875 |
| NTN1 | 0.875 |
| NTNG1 | 0.875 |
| NTSR1 | 0.875 |
| NUP133 | 0.875 |
| OARD1 | 0.875 |
| OFCC1 | 0.875 |
| OLAH | 0.875 |
| OR10V1 | 0.875 |
| OR10Z1 | 0.875 |
| OR2AE1 | 0.875 |
| OR2L2 | 0.875 |
| OR4F13P | 0.875 |
| OR51A7 | 0.875 |
| OR52K1 | 0.875 |
| OR5M8 | 0.875 |
| OR8H2 | 0.875 |
| OR8I2 | 0.875 |
| OTC | 0.875 |
| P2RY4 | 0.875 |
| PABPC3 | 0.875 |
| PAN3 | 0.875 |
| PCCA | 0.875 |
| PCSK9 | 0.875 |
| PCTP | 0.875 |
| PDZK1IP1 | 0.875 |
| PEAR1 | 0.875 |
| PHTF1 | 0.875 |
| PIAS3 | 0.875 |
| PIGO | 0.875 |
| PIGZ | 0.875 |
| PKD1L3 | 0.875 |
| PKP2 | 0.875 |
| PLAC9 | 0.875 |
| PLCH2 | 0.875 |
| PLEK2 | 0.875 |
| PLEKHA7 | 0.875 |
| PLK4 | 0.875 |
| PMAIP1 | 0.875 |
| PNO1 | 0.875 |
| POLN | 0.875 |
| POT1 | 0.875 |
| PPAPDC2 | 0.875 |
| PPFIA3 | 0.875 |
| PPP3R2 | 0.875 |
| PPP6R1 | 0.875 |
| PRKAA2 | 0.875 |
| PRR18 | 0.875 |
| PRRC1 | 0.875 |
| PSMB8 | 0.875 |
| PSMD11 | 0.875 |
| PTCHD1 | 0.875 |
| PTMA | 0.875 |
| PTPN14 | 0.875 |
| PURB | 0.875 |
| PYGL | 0.875 |
| PYGM | 0.875 |
| R3HCC1L | 0.875 |
| RAB3GAP2 | 0.875 |
| RBBP5 | 0.875 |
| RBBP7 | 0.875 |
| RET | 0.875 |
| RIN3 | 0.875 |
| RNASEL | 0.875 |
| RNF157 | 0.875 |
| RNF182 | 0.875 |
| RPS10 | 0.875 |
| RTP5 | 0.875 |
| RUNDC3A | 0.875 |
| S100A9 | 0.875 |
| SALL2 | 0.875 |
| SAPCD1 | 0.875 |
| SASH1 | 0.875 |
| SCUBE2 | 0.875 |
| SEC61A2 | 0.875 |
| SENP8 | 0.875 |
| SERF1B | 0.875 |
| SERPINA13P | 0.875 |
| SERPINB3 | 0.875 |
| SLAIN2 | 0.875 |
| SLC15A1 | 0.875 |
| SLC18A3 | 0.875 |
| SLC22A31 | 0.875 |
| SLC26A1 | 0.875 |
| SLC26A10 | 0.875 |
| SLC34A3 | 0.875 |
| SLC35A2 | 0.875 |
| SLC35E3 | 0.875 |
| SLU7 | 0.875 |
| SMARCD3 | 0.875 |
| SMIM11 | 0.875 |
| SMYD4 | 0.875 |
| SNORA30 | 0.875 |
| SNORA41 | 0.875 |
| SNORD115.21 | 0.875 |
| SNORD115.44 | 0.875 |
| SNORD27 | 0.875 |
| SNORD29 | 0.875 |
| SNORD44 | 0.875 |
| SNORD45B | 0.875 |
| SNORD47 | 0.875 |
| SNORD96A | 0.875 |
| SNTB1 | 0.875 |
| SNX13 | 0.875 |
| SNX17 | 0.875 |
| SP2 | 0.875 |
| SPATS2 | 0.875 |
| SPINT1 | 0.875 |
| SPNS3 | 0.875 |
| SPPL2A | 0.875 |
| SPRTN | 0.875 |
| SQLE | 0.875 |
| ST20 | 0.875 |
| STRN | 0.875 |
| STX3 | 0.875 |
| STXBP1 | 0.875 |
| SUGT1P3 | 0.875 |
| SYCP3 | 0.875 |
| SYT1 | 0.875 |
| TBATA | 0.875 |
| TCAIM | 0.875 |
| TCEAL7 | 0.875 |
| TCL1B | 0.875 |
| TCN1 | 0.875 |
| TECTB | 0.875 |
| TEDDM1 | 0.875 |
| TEX261 | 0.875 |
| TGM7 | 0.875 |
| TMCC2 | 0.875 |
| TMEM17 | 0.875 |
| TMEM190 | 0.875 |
| TMEM25 | 0.875 |
| TMEM59L | 0.875 |
| TMEM63C | 0.875 |
| TMEM64 | 0.875 |
| TMEM89 | 0.875 |
| TNFRSF11A | 0.875 |
| TNKS1BP1 | 0.875 |
| TNP1 | 0.875 |
| TOP3B | 0.875 |
| TRAPPC2 | 0.875 |
| TREML3P | 0.875 |
| TRHDE | 0.875 |
| TRIM13 | 0.875 |
| TRIO | 0.875 |
| TRIP10 | 0.875 |
| TRMT5 | 0.875 |
| TSNAXIP1 | 0.875 |
| TUBA1B | 0.875 |
| TYRP1 | 0.875 |
| U2SURP | 0.875 |
| UBE3D | 0.875 |
| UBQLNL | 0.875 |
| UBXN2B | 0.875 |
| UCN3 | 0.875 |
| USP21 | 0.875 |
| USP27X.AS1 | 0.875 |
| VN1R10P | 0.875 |
| VPS51 | 0.875 |
| VTRNA1.2 | 0.875 |
| VWA2 | 0.875 |
| VWA5A | 0.875 |
| VWA8 | 0.875 |
| WISP3 | 0.875 |
| XCL2 | 0.875 |
| XPNPEP3 | 0.875 |
| XYLB | 0.875 |
| YARS | 0.875 |
| YLPM1 | 0.875 |
| ZBTB1 | 0.875 |
| ZBTB10 | 0.875 |
| ZBTB11.AS1 | 0.875 |
| ZBTB33 | 0.875 |
| ZBTB43 | 0.875 |
| ZC3H12D | 0.875 |
| ZCCHC12 | 0.875 |
| ZFX | 0.875 |
| ZMYM4 | 0.875 |
| ZMYND11 | 0.875 |
| ZNF121 | 0.875 |
| ZNF132 | 0.875 |
| ZNF143 | 0.875 |
| ZNF568 | 0.875 |
| ZNF572 | 0.875 |
| ZNF816.ZNF321P | 0.875 |
| ZNF830 | 0.875 |
| ZNF852 | 0.875 |
| ZNF876P | 0.875 |
| ZNHIT6 | 0.875 |
| ZSCAN23 | 0.875 |
| ZSCAN5B | 0.875 |
| ZUFSP | 0.875 |
| ABCA8 | 0.833333 |
| ABCF1 | 0.833333 |
| ACBD5 | 0.833333 |
| ACSS2 | 0.833333 |
| ADAM20P1 | 0.833333 |
| ADCY8 | 0.833333 |
| ADORA2B | 0.833333 |
| AFF4 | 0.833333 |
| AIF1L | 0.833333 |
| AKIP1 | 0.833333 |
| AKT3 | 0.833333 |
| ALDH3A2 | 0.833333 |
| ALLC | 0.833333 |
| ALOXE3 | 0.833333 |
| ANKRD45 | 0.833333 |
| APOL2 | 0.833333 |
| AQP9 | 0.833333 |
| ARHGAP12 | 0.833333 |
| ARHGEF18 | 0.833333 |
| ARL14EP | 0.833333 |
| ARMC8 | 0.833333 |
| ARNT2 | 0.833333 |
| ARPC5L | 0.833333 |
| ASCC2 | 0.833333 |
| ASCL4 | 0.833333 |
| ASNS | 0.833333 |
| ASPM | 0.833333 |
| ATF7 | 0.833333 |
| ATPIF1 | 0.833333 |
| BAMBI | 0.833333 |
| BASP1 | 0.833333 |
| BDKRB2 | 0.833333 |
| BHMT2 | 0.833333 |
| BLID | 0.833333 |
| BNC2 | 0.833333 |
| BRPF3 | 0.833333 |
| C14orf178 | 0.833333 |
| C21orf62 | 0.833333 |
| C5orf15 | 0.833333 |
| C6orf57 | 0.833333 |
| C7orf62 | 0.833333 |
| C8orf12 | 0.833333 |
| CACNA1C | 0.833333 |
| CAMK2D | 0.833333 |
| CAT | 0.833333 |
| CCDC105 | 0.833333 |
| CCDC112 | 0.833333 |
| CCDC15 | 0.833333 |
| CCDC19 | 0.833333 |
| CCDC62 | 0.833333 |
| CCDC68 | 0.833333 |
| CD24 | 0.833333 |
| CD248 | 0.833333 |
| CDCA8 | 0.833333 |
| CDH18 | 0.833333 |
| CDK4 | 0.833333 |
| CENPV | 0.833333 |
| CGREF1 | 0.833333 |
| CHRD | 0.833333 |
| CHRNA3 | 0.833333 |
| CHST11 | 0.833333 |
| CKAP5 | 0.833333 |
| CLIP2 | 0.833333 |
| CLU | 0.833333 |
| COL6A2 | 0.833333 |
| COQ6 | 0.833333 |
| CPEB1 | 0.833333 |
| CPSF6 | 0.833333 |
| CRELD2 | 0.833333 |
| CRLF3 | 0.833333 |
| CSN1S1 | 0.833333 |
| CTBP1 | 0.833333 |
| CTSD | 0.833333 |
| CWC27 | 0.833333 |
| CXADR | 0.833333 |
| CXCL6 | 0.833333 |
| CYC1 | 0.833333 |
| CYFIP2 | 0.833333 |
| DCAF15 | 0.833333 |
| DCAF8L1 | 0.833333 |
| DCLK3 | 0.833333 |
| DDX39A | 0.833333 |
| DDX3Y | 0.833333 |
| DEFB119 | 0.833333 |
| DEGS2 | 0.833333 |
| DIAPH2 | 0.833333 |
| DLK2 | 0.833333 |
| DLX3 | 0.833333 |
| DNAJC3 | 0.833333 |
| DSCAML1 | 0.833333 |
| DTNA | 0.833333 |
| DUOX2 | 0.833333 |
| DUSP1 | 0.833333 |
| DYNC1H1 | 0.833333 |
| EFTUD1 | 0.833333 |
| EIF2S1 | 0.833333 |
| EIF5B | 0.833333 |
| ELOVL4 | 0.833333 |
| EMC1 | 0.833333 |
| EMP2 | 0.833333 |
| ENTPD5 | 0.833333 |
| EPHA4 | 0.833333 |
| ETNK1 | 0.833333 |
| EXOC6 | 0.833333 |
| F2RL3 | 0.833333 |
| FAM155B | 0.833333 |
| FAM208B | 0.833333 |
| FAM69A | 0.833333 |
| FAM81A | 0.833333 |
| FAT4 | 0.833333 |
| FBLN7 | 0.833333 |
| FBXO38 | 0.833333 |
| FBXO4 | 0.833333 |
| FIGN | 0.833333 |
| FLJ25758 | 0.833333 |
| FLJ37035 | 0.833333 |
| FLVCR1 | 0.833333 |
| FRMD6 | 0.833333 |
| FSD2 | 0.833333 |
| FTL | 0.833333 |
| GABRR3 | 0.833333 |
| GADD45B | 0.833333 |
| GALR2 | 0.833333 |
| GAP43 | 0.833333 |
| GBP7 | 0.833333 |
| GFRA1 | 0.833333 |
| GLIS3 | 0.833333 |
| GLTSCR2 | 0.833333 |
| GLYATL2 | 0.833333 |
| GNL1 | 0.833333 |
| GP9 | 0.833333 |
| GPC2 | 0.833333 |
| GPR150 | 0.833333 |
| GRIK4 | 0.833333 |
| GRIP2 | 0.833333 |
| GSAP | 0.833333 |
| GSDMB | 0.833333 |
| GSN | 0.833333 |
| GTF2A1 | 0.833333 |
| GTPBP2 | 0.833333 |
| GUCD1 | 0.833333 |
| GUSB | 0.833333 |
| H2AFV | 0.833333 |
| H6PD | 0.833333 |
| HBB | 0.833333 |
| HDGFL1 | 0.833333 |
| HEATR1 | 0.833333 |
| HEXB | 0.833333 |
| HID1 | 0.833333 |
| HIST1H2BJ | 0.833333 |
| HLA.DQB1 | 0.833333 |
| HMGCR | 0.833333 |
| HRH1 | 0.833333 |
| HSPB8 | 0.833333 |
| HUNK | 0.833333 |
| ID3 | 0.833333 |
| IFIT2 | 0.833333 |
| IGF2R | 0.833333 |
| IL1R1 | 0.833333 |
| IL7 | 0.833333 |
| INHBA | 0.833333 |
| INTS10 | 0.833333 |
| IPMK | 0.833333 |
| IPO4 | 0.833333 |
| ITGA7 | 0.833333 |
| ITPA | 0.833333 |
| JTB | 0.833333 |
| KCNA7 | 0.833333 |
| KCNH3 | 0.833333 |
| KCNT2 | 0.833333 |
| KDM5D | 0.833333 |
| KIAA0408 | 0.833333 |
| KIAA1107 | 0.833333 |
| KIAA1671 | 0.833333 |
| KIF18B | 0.833333 |
| KIF26B | 0.833333 |
| KLHDC8A | 0.833333 |
| KLHL24 | 0.833333 |
| KLHL28 | 0.833333 |
| KLK13 | 0.833333 |
| KRTAP20.3 | 0.833333 |
| LACTB2 | 0.833333 |
| LAP3 | 0.833333 |
| LARP1 | 0.833333 |
| LCE1A | 0.833333 |
| LIN52 | 0.833333 |
| LINGO2 | 0.833333 |
| LNX1 | 0.833333 |
| LOC100128035 | 0.833333 |
| LOC100132014 | 0.833333 |
| LOC100133299 | 0.833333 |
| LOC339803 | 0.833333 |
| LOC642980 | 0.833333 |
| LRIG1 | 0.833333 |
| LRRC16B | 0.833333 |
| LRRC47 | 0.833333 |
| LRRC6 | 0.833333 |
| LRRC61 | 0.833333 |
| LRRC7 | 0.833333 |
| LRRN1 | 0.833333 |
| LRRN2 | 0.833333 |
| MAF1 | 0.833333 |
| MAGEA3 | 0.833333 |
| MAGED1 | 0.833333 |
| MAGOH | 0.833333 |
| MAMDC4 | 0.833333 |
| MANEAL | 0.833333 |
| MAP2 | 0.833333 |
| MAP3K5 | 0.833333 |
| MAPK9 | 0.833333 |
| MBTPS2 | 0.833333 |
| MCOLN1 | 0.833333 |
| MCU | 0.833333 |
| MED1 | 0.833333 |
| MEST | 0.833333 |
| METTL25 | 0.833333 |
| MFI2 | 0.833333 |
| MFN2 | 0.833333 |
| MICAL2 | 0.833333 |
| MICALCL | 0.833333 |
| MICU1 | 0.833333 |
| MIR105.1 | 0.833333 |
| MIR517C | 0.833333 |
| MLF1 | 0.833333 |
| MLPH | 0.833333 |
| MOB1A | 0.833333 |
| MRFAP1L1 | 0.833333 |
| MRPL20 | 0.833333 |
| MRRF | 0.833333 |
| MSMO1 | 0.833333 |
| MTFMT | 0.833333 |
| MTHFD1 | 0.833333 |
| MTM1 | 0.833333 |
| MTUS2.AS1 | 0.833333 |
| MYADM | 0.833333 |
| MYBL2 | 0.833333 |
| MYLK | 0.833333 |
| N4BP2L2 | 0.833333 |
| NACC1 | 0.833333 |
| NAP1L1 | 0.833333 |
| NAPRT | 0.833333 |
| NCLN | 0.833333 |
| NCOA4 | 0.833333 |
| NDUFA2 | 0.833333 |
| NEFH | 0.833333 |
| NEK2 | 0.833333 |
| NEU1 | 0.833333 |
| NFIX | 0.833333 |
| NID2 | 0.833333 |
| NINL | 0.833333 |
| NLRP13 | 0.833333 |
| NOC2L | 0.833333 |
| NPC2 | 0.833333 |
| NR0B2 | 0.833333 |
| NTN3 | 0.833333 |
| NUDCD1 | 0.833333 |
| NUP160 | 0.833333 |
| NYX | 0.833333 |
| OK | 0.833333 |
| OLFML3 | 0.833333 |
| OR10A6 | 0.833333 |
| OR10P1 | 0.833333 |
| OR4A47 | 0.833333 |
| OR4D6 | 0.833333 |
| OR7D4 | 0.833333 |
| PABPC1L2A | 0.833333 |
| PAGR1 | 0.833333 |
| PARN | 0.833333 |
| PASD1 | 0.833333 |
| PCDHB12 | 0.833333 |
| PCDHB15 | 0.833333 |
| PCMTD2 | 0.833333 |
| PCSK1 | 0.833333 |
| PDK2 | 0.833333 |
| PDRG1 | 0.833333 |
| PDXK | 0.833333 |
| PEMT | 0.833333 |
| PGBD1 | 0.833333 |
| PHC2 | 0.833333 |
| PHGDH | 0.833333 |
| PKN1 | 0.833333 |
| PLAU | 0.833333 |
| PLCB4 | 0.833333 |
| PLEKHA2 | 0.833333 |
| POLG2 | 0.833333 |
| POU5F1P3 | 0.833333 |
| PPFIBP2 | 0.833333 |
| PPP1R14A | 0.833333 |
| PPP1R18 | 0.833333 |
| PPP1R26 | 0.833333 |
| PRAP1 | 0.833333 |
| PRELP | 0.833333 |
| PRMT5 | 0.833333 |
| PRR13 | 0.833333 |
| PRSS41 | 0.833333 |
| PSMA7 | 0.833333 |
| PTH | 0.833333 |
| PTPRN2 | 0.833333 |
| PTX4 | 0.833333 |
| RAB15 | 0.833333 |
| RAB22A | 0.833333 |
| RABGAP1L | 0.833333 |
| RAC3 | 0.833333 |
| RAD54L | 0.833333 |
| RASA2 | 0.833333 |
| RB1CC1 | 0.833333 |
| RBBP9 | 0.833333 |
| RBM18 | 0.833333 |
| RBM48 | 0.833333 |
| RBM8A | 0.833333 |
| RBP4 | 0.833333 |
| RDH10 | 0.833333 |
| REEP4 | 0.833333 |
| REG1P | 0.833333 |
| REM1 | 0.833333 |
| RFC5 | 0.833333 |
| RGL1 | 0.833333 |
| RHBDL1 | 0.833333 |
| RILPL2 | 0.833333 |
| RNF114 | 0.833333 |
| RNF13 | 0.833333 |
| RNF34 | 0.833333 |
| RNU11 | 0.833333 |
| RP2 | 0.833333 |
| RPN1 | 0.833333 |
| RRBP1 | 0.833333 |
| RSBN1 | 0.833333 |
| RUFY3 | 0.833333 |
| SACM1L | 0.833333 |
| SCAMP1 | 0.833333 |
| SCRG1 | 0.833333 |
| SGPP1 | 0.833333 |
| SIL1 | 0.833333 |
| SIN3B | 0.833333 |
| SIRT2 | 0.833333 |
| SLC13A5 | 0.833333 |
| SLC25A5 | 0.833333 |
| SLC25A6 | 0.833333 |
| SLC2A4RG | 0.833333 |
| SLC35F6 | 0.833333 |
| SLC39A4 | 0.833333 |
| SLC52A2 | 0.833333 |
| SLC5A9 | 0.833333 |
| SLFN5 | 0.833333 |
| SMOC1 | 0.833333 |
| SNAI1 | 0.833333 |
| SNORA64 | 0.833333 |
| SNORD115.41 | 0.833333 |
| SNORD116.12 | 0.833333 |
| SNORD116.16 | 0.833333 |
| SNORD45C | 0.833333 |
| SNORD51 | 0.833333 |
| SNORD54 | 0.833333 |
| SNRPB | 0.833333 |
| SNX2 | 0.833333 |
| SOCS2 | 0.833333 |
| SORCS2 | 0.833333 |
| SP100 | 0.833333 |
| SP4 | 0.833333 |
| SPAG7 | 0.833333 |
| SPATA12 | 0.833333 |
| SPATS1 | 0.833333 |
| SPG21 | 0.833333 |
| SPRY3 | 0.833333 |
| SRD5A3 | 0.833333 |
| SST | 0.833333 |
| ST3GAL1 | 0.833333 |
| ST6GAL1 | 0.833333 |
| STAT6 | 0.833333 |
| SUV39H1 | 0.833333 |
| SYF2 | 0.833333 |
| TAAR6 | 0.833333 |
| TADA2A | 0.833333 |
| TANC2 | 0.833333 |
| TANGO2 | 0.833333 |
| TANGO6 | 0.833333 |
| TAOK3 | 0.833333 |
| TARS | 0.833333 |
| TCTEX1D4 | 0.833333 |
| TDP2 | 0.833333 |
| TEX30 | 0.833333 |
| TEX38 | 0.833333 |
| THNSL2 | 0.833333 |
| THOP1 | 0.833333 |
| THTPA | 0.833333 |
| TICRR | 0.833333 |
| TMC3 | 0.833333 |
| TMEM107 | 0.833333 |
| TMEM192 | 0.833333 |
| TMEM245 | 0.833333 |
| TNFRSF11B | 0.833333 |
| TNFRSF19 | 0.833333 |
| TNNT2 | 0.833333 |
| TP53BP2 | 0.833333 |
| TP53TG1 | 0.833333 |
| TPRX1 | 0.833333 |
| TPST2 | 0.833333 |
| TRAFD1 | 0.833333 |
| TRIM22 | 0.833333 |
| TRIM68 | 0.833333 |
| TSC22D1 | 0.833333 |
| TSSK3 | 0.833333 |
| TTC28 | 0.833333 |
| TWIST1 | 0.833333 |
| UBA1 | 0.833333 |
| UBE2L6 | 0.833333 |
| UEVLD | 0.833333 |
| ULBP1 | 0.833333 |
| UNK | 0.833333 |
| UQCR11 | 0.833333 |
| USP9Y | 0.833333 |
| VCAM1 | 0.833333 |
| WAC | 0.833333 |
| WDR55 | 0.833333 |
| WISP1 | 0.833333 |
| XPO7 | 0.833333 |
| ZDHHC14 | 0.833333 |
| ZDHHC2 | 0.833333 |
| ZFYVE1 | 0.833333 |
| ZFYVE19 | 0.833333 |
| ZGRF1 | 0.833333 |
| ZNF140 | 0.833333 |
| ZNF175 | 0.833333 |
| ZNF24 | 0.833333 |
| ZNF37BP | 0.833333 |
| ZNF396 | 0.833333 |
| ZNF454 | 0.833333 |
| ZNF510 | 0.833333 |
| ZNF584 | 0.833333 |
| ZNF597 | 0.833333 |
| ZNF645 | 0.833333 |
| ZNF662 | 0.833333 |
| ZNF695 | 0.833333 |
| ZNF826P | 0.833333 |
| ZNF90 | 0.833333 |
| ZNFX1 | 0.833333 |
| A4GALT | 0.791667 |
| AMBP | 0.791667 |
| ARR3 | 0.791667 |
| BHLHA9 | 0.791667 |
| BMX | 0.791667 |
| BPIFA3 | 0.791667 |
| C11orf16 | 0.791667 |
| C12orf76 | 0.791667 |
| CLCA2 | 0.791667 |
| COL4A6 | 0.791667 |
| CPD | 0.791667 |
| DCLRE1B | 0.791667 |
| EXOC3L1 | 0.791667 |
| FBXL16 | 0.791667 |
| HRH4 | 0.791667 |
| JPX | 0.791667 |
| KCNK16 | 0.791667 |
| LANCL3 | 0.791667 |
| LCE1C | 0.791667 |
| LMO1 | 0.791667 |
| LOC100129540 | 0.791667 |
| LOC399898 | 0.791667 |
| LOC401410 | 0.791667 |
| LRRC10 | 0.791667 |
| MIRLET7G | 0.791667 |
| OR10D3 | 0.791667 |
| OR13G1 | 0.791667 |
| OR1A1 | 0.791667 |
| OR52B6 | 0.791667 |
| PP2672 | 0.791667 |
| RBAK | 0.791667 |
| RIMKLA | 0.791667 |
| SCN3B | 0.791667 |
| SLC22A13 | 0.791667 |
| SLC25A36 | 0.791667 |
| SLC41A1 | 0.791667 |
| SPATA8 | 0.791667 |
| TRPC4 | 0.791667 |
| VCX3B | 0.791667 |
| CAMK2N1 | 0.75 |
| DPH7 | 0.75 |
| EYA2 | 0.75 |
| IRF7 | 0.75 |
| IVL | 0.75 |
| LRRC26 | 0.75 |
| MT1G | 0.75 |
| OR1N1 | 0.75 |
| OR6B1 | 0.75 |
| PAX8.AS1 | 0.75 |
| SLED1 | 0.75 |
| TOM1L1 | 0.75 |
| UBLCP1 | 0.75 |
| USP11 | 0.75 |
| ADORA2A.AS1 | 0.708333 |
| CCDC78 | 0.708333 |
| CLDN10 | 0.708333 |
| KIF21B | 0.708333 |
| OR5C1 | 0.708333 |
