# Supplementary material for: KIF21B Expression in Osteosarcoma and Its Regulatory Effect on Osteosarcoma Cell Proliferation and Apoptosis Through the PI3K/AKT Pathway
Source: Front Oncol. 2021 Jan 28;10:606765. doi: 10.3389/fonc.2020.606765 (PMC7879035; doi:10.3389/fonc.2020.606765)
Supplement: Supplementary file 3 [file Table_2.docx]

Supplementary Table 2 Kaplan-Meier survival analysis for differentially expressed genes

| Symbol | Pvalue |
| --- | --- |
| CLUAP1 | 0.00001 |
| GRWD1 | 0.00005 |
| NUBP1 | 0.00007 |
| ALDH1A1 | 0.00009 |
| RHNO1 | 0.0001 |
| ADAT1 | 0.0001 |
| DUSP18 | 0.00011 |
| AKNA | 0.00021 |
| PIGV | 0.00022 |
| MAGEB2 | 0.00025 |
| SLC38A7 | 0.00025 |
| APPBP2 | 0.00025 |
| HGF | 0.00026 |
| CALD1 | 0.00028 |
| IL2RA | 0.00028 |
| PML | 0.00029 |
| PLA2G15 | 0.00029 |
| JAG2 | 0.00031 |
| FCER1G | 0.00032 |
| EHMT2 | 0.00032 |
| CREBL2 | 0.00032 |
| PLXND1 | 0.00033 |
| GLB1 | 0.00036 |
| BST1 | 0.00036 |
| SLCO2B1 | 0.00036 |
| TC2N | 0.00037 |
| TRIM68 | 0.00043 |
| LYRM1 | 0.00044 |
| LBX2 | 0.00045 |
| GNGT1 | 0.00046 |
| USP10 | 0.00054 |
| SLC35F6 | 0.00057 |
| LURAP1L | 0.00058 |
| MAP3K5 | 0.0006 |
| HARBI1 | 0.00061 |
| NBR1 | 0.00062 |
| DEF6 | 0.00063 |
| MOB1A | 0.00064 |
| DCAKD | 0.00064 |
| PTPRO | 0.00066 |
| YLPM1 | 0.00066 |
| GNAI3 | 0.00067 |
| CGREF1 | 0.00067 |
| FLYWCH2 | 0.00067 |
| NLRP10 | 0.00068 |
| HAVCR2 | 0.00072 |
| CYP2C8 | 0.00072 |
| CFL1 | 0.00073 |
| RAB3IP | 0.00074 |
| ZNF101 | 0.00075 |
| IGBP1 | 0.00076 |
| SPG11 | 0.00076 |
| THAP10 | 0.00078 |
| CCDC102B | 0.00078 |
| TADA3 | 0.00084 |
| CSF1R | 0.00084 |
| DUSP3 | 0.00084 |
| NIPA2 | 0.00085 |
| PIP5K1C | 0.00086 |
| AP2S1 | 0.00086 |
| ZNF583 | 0.00086 |
| ZNF214 | 0.00088 |
| VASP | 0.00088 |
| CARD6 | 0.00089 |
| MAGEE1 | 0.0009 |
| ASIC5 | 0.00094 |
| TIMM50 | 0.00094 |
| MARVELD3 | 0.00095 |
| KCNJ5 | 0.001 |
| TDP2 | 0.00102 |
| ABHD14B | 0.00103 |
| RTN4 | 0.00106 |
| GZMB | 0.00106 |
| ATP6V0E1 | 0.0011 |
| TOX | 0.0011 |
| PKIA | 0.0011 |
| FERMT3 | 0.00111 |
| RAB8B | 0.00111 |
| DPH3 | 0.00117 |
| VIPAS39 | 0.0012 |
| RHEBL1 | 0.00121 |
| HYAL2 | 0.00125 |
| CLIP2 | 0.00127 |
| AMBRA1 | 0.00128 |
| TNFRSF1A | 0.00128 |
| NRBP1 | 0.0013 |
| APOL2 | 0.0013 |
| PCED1B | 0.00132 |
| BCAR1 | 0.00133 |
| EXOC6B | 0.00136 |
| ACTB | 0.00136 |
| FOLR2 | 0.00136 |
| TMEM125 | 0.0014 |
| AKIP1 | 0.0014 |
| TMUB2 | 0.00141 |
| EMILIN2 | 0.00141 |
| ICAM3 | 0.00144 |
| CD180 | 0.00147 |
| DOCK8 | 0.00147 |
| CD53 | 0.00151 |
| SIPA1L1 | 0.00152 |
| GBP4 | 0.00155 |
| RAP1B | 0.00159 |
| NCEH1 | 0.0016 |
| KIF25 | 0.0016 |
| PARP3 | 0.00164 |
| MS4A4A | 0.00164 |
| ABCD3 | 0.00167 |
| TWF2 | 0.00167 |
| PARVG | 0.00168 |
| RASGRP2 | 0.00171 |
| IRF5 | 0.00172 |
| NPC2 | 0.00172 |
| ERCC4 | 0.00174 |
| CCDC71L | 0.00175 |
| IFT140 | 0.00175 |
| RNF185 | 0.00175 |
| ADD1 | 0.00181 |
| ANKRD24 | 0.00183 |
| HSDL1 | 0.00185 |
| BLVRB | 0.00185 |
| CCDC96 | 0.00189 |
| TTC8 | 0.00189 |
| PIAS1 | 0.00191 |
| SNAP23 | 0.00195 |
| ZNF584 | 0.00196 |
| WFS1 | 0.00198 |
| EXD2 | 0.00202 |
| CEACAM16 | 0.00207 |
| MAMLD1 | 0.00209 |
| RABIF | 0.0021 |
| SF3B3 | 0.00212 |
| ZNF561 | 0.00212 |
| NDUFB2 | 0.00213 |
| SNTB2 | 0.00213 |
| G6PD | 0.00217 |
| TLR1 | 0.00221 |
| IL13RA2 | 0.00221 |
| TMEM51 | 0.00222 |
| C1QC | 0.00229 |
| LIMA1 | 0.00229 |
| ETV1 | 0.0023 |
| GJA4 | 0.00231 |
| TPM4 | 0.00231 |
| RNASE6 | 0.00233 |
| USP11 | 0.00236 |
| C8orf48 | 0.0024 |
| RAB5C | 0.00243 |
| EHD2 | 0.00243 |
| FIP1L1 | 0.00244 |
| RFWD3 | 0.00244 |
| DCP1B | 0.00245 |
| PIGQ | 0.00249 |
| FAM174B | 0.0025 |
| PSMB8 | 0.00257 |
| RAET1G | 0.00257 |
| NUAK2 | 0.00259 |
| SCFD2 | 0.00259 |
| SOCS5 | 0.00261 |
| MAGEA4 | 0.00263 |
| MYL9 | 0.00265 |
| TELO2 | 0.00266 |
| ARL8B | 0.00268 |
| HCLS1 | 0.00269 |
| GLCE | 0.00271 |
| UBE2L6 | 0.00274 |
| SLC11A1 | 0.00275 |
| EIF4E3 | 0.00277 |
| CES2 | 0.00282 |
| NDUFA3 | 0.00283 |
| ACSL5 | 0.00285 |
| CLPTM1 | 0.00286 |
| TNFSF10 | 0.00291 |
| ITGAM | 0.00293 |
| TCTA | 0.00296 |
| ACSS3 | 0.00296 |
| WNT5A | 0.00298 |
| XAB2 | 0.00299 |
| SLC16A2 | 0.00303 |
| CASS4 | 0.00304 |
| S100A2 | 0.00308 |
| GALNT14 | 0.00309 |
| HTR2B | 0.00309 |
| BBS2 | 0.0031 |
| SDF2 | 0.00313 |
| SUPT20H | 0.00314 |
| RNASE2 | 0.00315 |
| RIN3 | 0.00318 |
| SELL | 0.00319 |
| LRMP | 0.0032 |
| DDAH2 | 0.00321 |
| CHCHD4 | 0.00327 |
| GLIPR1 | 0.00331 |
| RASGRP4 | 0.00331 |
| BMP8A | 0.00332 |
| TYROBP | 0.00333 |
| SRP72 | 0.00334 |
| SPI1 | 0.00334 |
| CD48 | 0.00336 |
| CD163 | 0.00337 |
| BCL2 | 0.00338 |
| PLB1 | 0.00343 |
| MSRB1 | 0.00345 |
| GABPB1 | 0.0035 |
| SH2B3 | 0.00351 |
| DIAPH1 | 0.00353 |
| CITED4 | 0.00355 |
| CRNKL1 | 0.00358 |
| LRRC8D | 0.00359 |
| FNBP1L | 0.00365 |
| BBOX1 | 0.00365 |
| ISL1 | 0.00366 |
| PEPD | 0.00367 |
| ANXA1 | 0.00372 |
| LEPR | 0.00374 |
| ANGPTL1 | 0.00375 |
| C3orf14 | 0.00378 |
| CD200R1 | 0.00378 |
| TCEANC | 0.00378 |
| STX7 | 0.00379 |
| NCKAP1L | 0.0038 |
| CLTC | 0.0038 |
| HOXA1 | 0.00381 |
| ARF5 | 0.00384 |
| NPRL3 | 0.00384 |
| RAG2 | 0.00385 |
| SLC9A6 | 0.00388 |
| URM1 | 0.00388 |
| ACTA2 | 0.00391 |
| MYOF | 0.00391 |
| TFF3 | 0.00393 |
| FGFRL1 | 0.00397 |
| ITGAL | 0.00397 |
| VPS18 | 0.00401 |
| TFIP11 | 0.00401 |
| SNED1 | 0.00402 |
| ZDHHC7 | 0.00404 |
| GRPEL1 | 0.00405 |
| CDC42SE2 | 0.00405 |
| TOMM40 | 0.00409 |
| TUBB6 | 0.0041 |
| FAM192A | 0.00413 |
| TRAPPC1 | 0.00413 |
| S100A11 | 0.00419 |
| ANO3 | 0.00419 |
| RBM10 | 0.0042 |
| PGPEP1 | 0.00421 |
| LARP6 | 0.00422 |
| ILF2 | 0.00431 |
| SESN2 | 0.00433 |
| MRPL10 | 0.00433 |
| ITPKC | 0.00433 |
| DSE | 0.00435 |
| CYFIP1 | 0.00436 |
| KCTD21 | 0.0044 |
| ARHGDIB | 0.00443 |
| TGFBRAP1 | 0.00444 |
| CMKLR1 | 0.00444 |
| MBTPS1 | 0.00446 |
| BPHL | 0.00447 |
| PRNP | 0.00449 |
| ADAMTS1 | 0.00454 |
| TMEM43 | 0.00462 |
| CYBB | 0.00462 |
| LY96 | 0.00462 |
| APOC1 | 0.00464 |
| LGALS1 | 0.00466 |
| POLR3H | 0.00468 |
| M6PR | 0.00473 |
| TTLL1 | 0.00476 |
| MDFIC | 0.00477 |
| C14orf180 | 0.00478 |
| ZNF597 | 0.0048 |
| DNAJA2 | 0.00483 |
| SUPT4H1 | 0.00491 |
| IL1RN | 0.00492 |
| TGFBR2 | 0.00492 |
| CSTA | 0.00492 |
| CD2 | 0.00496 |
| TLN2 | 0.00496 |
| CSF1 | 0.00499 |
| FNBP1 | 0.00499 |
| LPXN | 0.005 |
| TGFBR3 | 0.00505 |
| CITED2 | 0.00506 |
| HERC5 | 0.00508 |
| SLC48A1 | 0.0051 |
| CAT | 0.00512 |
| ZNF624 | 0.00512 |
| MAGEA3 | 0.00516 |
| CAPZA2 | 0.00517 |
| TAGLN | 0.00518 |
| GYPC | 0.00522 |
| GRAMD1B | 0.00524 |
| PSEN1 | 0.00525 |
| ZNF844 | 0.00526 |
| TXLNB | 0.00531 |
| FCGR2A | 0.00534 |
| MAB21L3 | 0.00537 |
| FMN2 | 0.00547 |
| VAMP8 | 0.00547 |
| MON1B | 0.00555 |
| SNX9 | 0.00559 |
| C1QTNF7 | 0.0056 |
| C4BPB | 0.00571 |
| UBIAD1 | 0.00575 |
| ROCK2 | 0.00575 |
| PSMC4 | 0.00575 |
| ADORA2B | 0.00576 |
| AKR1A1 | 0.0058 |
| EPYC | 0.0058 |
| MAML2 | 0.00583 |
| CACFD1 | 0.00585 |
| CDK5 | 0.0059 |
| TES | 0.00595 |
| MAGEC2 | 0.00596 |
| GRIK2 | 0.00597 |
| GNG12 | 0.006 |
| UBASH3B | 0.00603 |
| TOX3 | 0.00608 |
| NREP | 0.00613 |
| ZYX | 0.00617 |
| HDAC8 | 0.00619 |
| TRIM69 | 0.00621 |
| ITGA11 | 0.00625 |
| SQSTM1 | 0.0063 |
| PDLIM7 | 0.00635 |
| TMEM127 | 0.00639 |
| MAPK14 | 0.0064 |
| ZC2HC1A | 0.0064 |
| PNMA1 | 0.00641 |
| ABCA8 | 0.00644 |
| DDAH1 | 0.00645 |
| CD38 | 0.00645 |
| GBP5 | 0.00648 |
| COG4 | 0.00649 |
| KLHL30 | 0.0065 |
| SPATA20 | 0.00654 |
| TMEM53 | 0.00655 |
| TMEM18 | 0.00655 |
| PARN | 0.00655 |
| SLC12A6 | 0.0066 |
| TRIM62 | 0.00664 |
| SH3BGRL3 | 0.00666 |
| PIK3R3 | 0.00666 |
| PIK3CG | 0.00667 |
| NKAIN2 | 0.00669 |
| GOSR1 | 0.00672 |
| BFAR | 0.00673 |
| RGL2 | 0.00676 |
| TAPBP | 0.00676 |
| FCGR1A | 0.00687 |
| KIAA1549L | 0.0069 |
| ARPC4 | 0.00695 |
| ENG | 0.00697 |
| REM1 | 0.00705 |
| C3orf80 | 0.00706 |
| CCSER1 | 0.00707 |
| CRYM | 0.00707 |
| OLFML3 | 0.00711 |
| SHKBP1 | 0.00712 |
| CIITA | 0.00714 |
| NUDT16 | 0.00716 |
| UBXN2A | 0.00721 |
| ECE1 | 0.00724 |
| C1QA | 0.00725 |
| NTN4 | 0.00725 |
| EDNRA | 0.00728 |
| FHL5 | 0.00729 |
| TLR7 | 0.00729 |
| CCDC126 | 0.00732 |
| EDIL3 | 0.00734 |
| BLCAP | 0.00737 |
| NUTF2 | 0.00738 |
| TMEM184C | 0.00739 |
| TTC4 | 0.00748 |
| TMEM107 | 0.00748 |
| TUBA1A | 0.00749 |
| DOCK7 | 0.00754 |
| FAM49B | 0.00755 |
| SH3BP4 | 0.00756 |
| SPG21 | 0.00761 |
| COL15A1 | 0.00763 |
| ZNF93 | 0.00765 |
| ZNF175 | 0.00766 |
| GDPD1 | 0.00769 |
| CNTN1 | 0.00772 |
| SUPT6H | 0.00773 |
| WNT9B | 0.00773 |
| VAV1 | 0.00773 |
| FCGR2B | 0.00777 |
| TMEM9B | 0.00782 |
| PIEZO2 | 0.00782 |
| ARHGAP28 | 0.00788 |
| EVI2B | 0.00789 |
| MMP2 | 0.00791 |
| LRRC7 | 0.00792 |
| CARM1 | 0.00792 |
| 2-Mar | 0.00797 |
| LIMS1 | 0.00815 |
| UQCRC1 | 0.00815 |
| SEL1L3 | 0.00816 |
| RAB11A | 0.00817 |
| LSM10 | 0.00818 |
| SLC7A7 | 0.00819 |
| F13A1 | 0.0082 |
| RBCK1 | 0.00822 |
| ATP6V1H | 0.00823 |
| ROR2 | 0.00833 |
| IFNL1 | 0.00833 |
| SLC30A4 | 0.00834 |
| PDE12 | 0.0084 |
| SIGLEC14 | 0.00841 |
| MX2 | 0.00843 |
| ISLR | 0.00846 |
| TRAF3IP2 | 0.00849 |
| NKX6-2 | 0.0085 |
| GABRA3 | 0.00851 |
| KIF3B | 0.00855 |
| SDC1 | 0.00858 |
| TMX3 | 0.0086 |
| SUPT5H | 0.00863 |
| PLEKHB2 | 0.00864 |
| NKG7 | 0.00864 |
| SSX2IP | 0.00871 |
| CTSL | 0.00875 |
| IFITM2 | 0.00876 |
| FLOT1 | 0.00877 |
| IFI44 | 0.00883 |
| CD300C | 0.00883 |
| KLHL26 | 0.00884 |
| FOXO4 | 0.00892 |
| BTBD10 | 0.00893 |
| REPS2 | 0.00897 |
| LHPP | 0.00901 |
| B3GNT7 | 0.00902 |
| SNX12 | 0.00909 |
| KANK2 | 0.00916 |
| CRAT | 0.00918 |
| AKT2 | 0.00918 |
| EHD4 | 0.00919 |
| FIGN | 0.00926 |
| PIK3R5 | 0.00929 |
| ZBED5 | 0.0093 |
| PTPN6 | 0.00932 |
| MRPL28 | 0.00935 |
| TBX15 | 0.00938 |
| SCOC | 0.00939 |
| PRKCH | 0.00942 |
| POU2F2 | 0.00948 |
| CNN2 | 0.0095 |
| MED27 | 0.00953 |
| KBTBD8 | 0.00961 |
| CAPZB | 0.00962 |
| TLR2 | 0.00965 |
| CYSLTR1 | 0.00965 |
| P2RY10 | 0.00967 |
| FOXJ2 | 0.00969 |
| PARP14 | 0.0097 |
| VCX3B | 0.00973 |
| FBLN1 | 0.00975 |
| DR1 | 0.00976 |
| SLC45A4 | 0.00976 |
| ATP6V1E1 | 0.00976 |
| SRGAP3 | 0.00977 |
| CORO1A | 0.00978 |
| PITPNC1 | 0.00979 |
| KCNJ16 | 0.00979 |
| TRIM44 | 0.0098 |
| LY86 | 0.00994 |
| CACNA2D3 | 0.00997 |
| MVP | 0.01002 |
| RAB32 | 0.01004 |
| ACP2 | 0.01006 |
| CTF1 | 0.01011 |
| EFEMP1 | 0.01021 |
| GLIPR2 | 0.01024 |
| SYNE1 | 0.01026 |
| GALC | 0.01026 |
| CYP2S1 | 0.01037 |
| ITGB5 | 0.01038 |
| ARHGAP9 | 0.0104 |
| CYYR1 | 0.01041 |
| CD14 | 0.01045 |
| PEX26 | 0.01048 |
| SYPL1 | 0.01052 |
| MTO1 | 0.01053 |
| TRAPPC3 | 0.01054 |
| ZSCAN31 | 0.01055 |
| MSN | 0.01055 |
| TNFSF8 | 0.01055 |
| CHMP2B | 0.01056 |
| IDUA | 0.01057 |
| FAAH | 0.01061 |
| FOPNL | 0.01062 |
| NEGR1 | 0.01063 |
| LYVE1 | 0.01063 |
| CDK6 | 0.0107 |
| CD3EAP | 0.01073 |
| TNFSF4 | 0.01079 |
| OGFOD1 | 0.0108 |
| NTAN1 | 0.01082 |
| VPS25 | 0.01084 |
| HDAC7 | 0.01086 |
| CNEP1R1 | 0.01088 |
| GPR158 | 0.0109 |
| EIF2AK4 | 0.01092 |
| STXBP6 | 0.01095 |
| ACTG1 | 0.01095 |
| RFT1 | 0.01098 |
| SSH1 | 0.01104 |
| TADA1 | 0.01105 |
| PTPRC | 0.01106 |
| FLRT3 | 0.01108 |
| PDZD11 | 0.0111 |
| BRK1 | 0.01115 |
| CREM | 0.01117 |
| SAMD3 | 0.01122 |
| TPM1 | 0.01124 |
| STOM | 0.01126 |
| LACTB | 0.01126 |
| GBP2 | 0.01129 |
| BMP8B | 0.01132 |
| SKIV2L | 0.01138 |
| ALPK2 | 0.0114 |
| TEK | 0.01141 |
| TRAPPC2 | 0.01142 |
| AP1G1 | 0.01142 |
| PARVA | 0.01146 |
| GJA5 | 0.01148 |
| MLLT11 | 0.01153 |
| GNB5 | 0.01155 |
| ZC3H18 | 0.01156 |
| OPN3 | 0.01158 |
| PLEK | 0.0116 |
| GIT2 | 0.01161 |
| SLC27A4 | 0.01163 |
| SLFN5 | 0.01163 |
| GNAI2 | 0.01171 |
| CAV2 | 0.01171 |
| ST6GAL1 | 0.01172 |
| PSMD12 | 0.01179 |
| TNKS1BP1 | 0.0118 |
| SH3GL1 | 0.01183 |
| WDR82 | 0.01186 |
| FABP6 | 0.01186 |
| TK2 | 0.01196 |
| CD33 | 0.01197 |
| MORC2 | 0.01206 |
| C1QB | 0.01208 |
| SFMBT2 | 0.01208 |
| SUOX | 0.01214 |
| SPTLC3 | 0.01221 |
| CLDN9 | 0.01224 |
| GOT2 | 0.01232 |
| BBS4 | 0.01233 |
| CD4 | 0.01236 |
| PHKA2 | 0.01245 |
| NEU3 | 0.01252 |
| EXTL2 | 0.01253 |
| SLBP | 0.01257 |
| LCMT1 | 0.0126 |
| HSBP1 | 0.01263 |
| PLXNC1 | 0.01265 |
| ELMO2 | 0.01265 |
| LSM2 | 0.01266 |
| NOXO1 | 0.01279 |
| NDRG4 | 0.0128 |
| C3AR1 | 0.01282 |
| PSMF1 | 0.01284 |
| GATA3 | 0.01288 |
| HSCB | 0.01289 |
| KCNIP4 | 0.01291 |
| UTRN | 0.01298 |
| AP1S1 | 0.01301 |
| CD59 | 0.01301 |
| MRC1 | 0.01303 |
| KLRC3 | 0.01305 |
| GPX4 | 0.0131 |
| C11orf45 | 0.01314 |
| HCK | 0.01315 |
| COMMD9 | 0.01316 |
| TMEM64 | 0.01319 |
| BNIP2 | 0.01321 |
| ANPEP | 0.01322 |
| PHKB | 0.01323 |
| KIF21B | 0.01334 |
| RCN2 | 0.01334 |
| IFNGR1 | 0.01336 |
| PRSS48 | 0.01338 |
| CLCN7 | 0.01343 |
| CISH | 0.01354 |
| TMEM176A | 0.01354 |
| VGLL3 | 0.01359 |
| NFAM1 | 0.01364 |
| ANAPC13 | 0.01368 |
| HINT2 | 0.01368 |
| PPP4R1 | 0.01368 |
| ACOT13 | 0.01377 |
| KRT7 | 0.01378 |
| TBCB | 0.01379 |
| HSPBP1 | 0.01395 |
| DAD1 | 0.01398 |
| PHOSPHO1 | 0.01409 |
| CDA | 0.01411 |
| TMSB10 | 0.01412 |
| 1-Mar | 0.01414 |
| PI4K2B | 0.01421 |
| IL18R1 | 0.01422 |
| ADCK1 | 0.01425 |
| ABAT | 0.01426 |
| PEX12 | 0.01429 |
| FMO1 | 0.01433 |
| NAGA | 0.01437 |
| ZMPSTE24 | 0.0144 |
| PGD | 0.01443 |
| EIF4G2 | 0.01451 |
| CTTNBP2NL | 0.01452 |
| GAK | 0.01459 |
| ZNF107 | 0.01466 |
| FEM1B | 0.01476 |
| RABEPK | 0.01477 |
| SLC35F5 | 0.01484 |
| TOR3A | 0.01485 |
| PRKACG | 0.01485 |
| SLC38A5 | 0.01491 |
| WAS | 0.01494 |
| TGM2 | 0.01496 |
| MIEF1 | 0.01497 |
| THUMPD3 | 0.01498 |
| CYTIP | 0.015 |
| LMF1 | 0.01502 |
| CCL2 | 0.01504 |
| PRAF2 | 0.01507 |
| PLXNA2 | 0.01514 |
| TIGD6 | 0.01514 |
| C9orf66 | 0.01514 |
| C6orf89 | 0.01515 |
| DIS3L | 0.01515 |
| ABI3 | 0.01519 |
| IGFL4 | 0.01522 |
| SOS1 | 0.01525 |
| GNPDA2 | 0.01526 |
| SMPD1 | 0.0153 |
| ADRB2 | 0.01532 |
| BTLA | 0.01539 |
| VCAN | 0.01541 |
| LGR6 | 0.01542 |
| FBLN5 | 0.01547 |
| CCDC146 | 0.01549 |
| CEP89 | 0.01551 |
| C11orf68 | 0.01556 |
| PXDC1 | 0.01558 |
| PPP2R5E | 0.01558 |
| COL8A1 | 0.01559 |
| DYNLL2 | 0.01566 |
| MAGED1 | 0.01568 |
| OSBPL3 | 0.0157 |
| AIM2 | 0.01588 |
| HEBP2 | 0.01593 |
| MAGEL2 | 0.01599 |
| KCNC2 | 0.01602 |
| FAM124B | 0.01607 |
| NEU1 | 0.01608 |
| SOD2 | 0.01608 |
| COPS8 | 0.0161 |
| TNFRSF21 | 0.01614 |
| TTC28 | 0.01625 |
| NAGPA | 0.01636 |
| AGFG1 | 0.0164 |
| KCNJ8 | 0.0164 |
| TLL1 | 0.01641 |
| RTCB | 0.01642 |
| ATP6V0D1 | 0.01643 |
| TLN1 | 0.01649 |
| NLRX1 | 0.01654 |
| SDCBP | 0.01655 |
| DDA1 | 0.01663 |
| LARS2 | 0.01667 |
| TNFRSF11A | 0.0167 |
| ACVRL1 | 0.01671 |
| SNRPB | 0.01672 |
| FPR1 | 0.01679 |
| FFAR4 | 0.0168 |
| ARG2 | 0.0168 |
| C15orf39 | 0.01683 |
| CSF3R | 0.01684 |
| GIPC2 | 0.01684 |
| NDST3 | 0.01687 |
| FAM129A | 0.0169 |
| VPS35 | 0.01692 |
| CD52 | 0.01694 |
| EFNB1 | 0.01695 |
| TAF13 | 0.01701 |
| EPHA4 | 0.01701 |
| THRB | 0.01701 |
| CPE | 0.01701 |
| SHISA5 | 0.01703 |
| IGFBP6 | 0.0171 |
| PSMD8 | 0.01715 |
| VAV3 | 0.01719 |
| ARHGDIA | 0.0172 |
| SKAP2 | 0.01722 |
| SAE1 | 0.01723 |
| LCK | 0.01724 |
| PSMD10 | 0.01725 |
| ARPC1B | 0.0173 |
| SNX10 | 0.01731 |
| NIP7 | 0.01733 |
| IGDCC4 | 0.01735 |
| PDXK | 0.01735 |
| GNAQ | 0.01737 |
| DNAJC8 | 0.01742 |
| TBPL1 | 0.01749 |
| TRIM5 | 0.01749 |
| MAP3K7 | 0.01753 |
| TMEM176B | 0.01757 |
| VTA1 | 0.01763 |
| ARL6IP1 | 0.01763 |
| DUSP10 | 0.01767 |
| TMEM203 | 0.01775 |
| HMOX2 | 0.01776 |
| PEA15 | 0.01777 |
| HES1 | 0.01786 |
| TCTEX1D1 | 0.01789 |
| PLD1 | 0.018 |
| API5 | 0.018 |
| SDSL | 0.01803 |
| STAT4 | 0.01808 |
| BSDC1 | 0.01826 |
| PRR13 | 0.01826 |
| PIGW | 0.01826 |
| PSTPIP2 | 0.0183 |
| CC2D2A | 0.01831 |
| AP2B1 | 0.01832 |
| POTEE | 0.01834 |
| LAIR1 | 0.01841 |
| PPP6R1 | 0.01843 |
| CKMT2 | 0.0185 |
| IFIH1 | 0.01853 |
| PLBD2 | 0.01862 |
| CHP1 | 0.01863 |
| OSBPL10 | 0.01864 |
| CFD | 0.01865 |
| PLA2G4C | 0.0187 |
| PHB | 0.01874 |
| TRERF1 | 0.0188 |
| ARL14EP | 0.0188 |
| FBXL12 | 0.01882 |
| GPR183 | 0.01885 |
| SLC2A12 | 0.01886 |
| NCF4 | 0.01887 |
| MOB3A | 0.01888 |
| BPI | 0.01895 |
| TMEM88 | 0.01897 |
| PTPMT1 | 0.01902 |
| VWA5A | 0.01904 |
| AHDC1 | 0.01905 |
| APMAP | 0.01905 |
| COA3 | 0.01913 |
| MBP | 0.01914 |
| WDR24 | 0.0192 |
| PLEKHF1 | 0.01922 |
| GAB2 | 0.01925 |
| NCOA5 | 0.01927 |
| HSD3B7 | 0.01932 |
| CYTH4 | 0.01932 |
| CDIPT | 0.01935 |
| LILRB4 | 0.01938 |
| SPTY2D1 | 0.01946 |
| RLIM | 0.01948 |
| SORL1 | 0.01957 |
| TBC1D22B | 0.01958 |
| LTK | 0.01959 |
| PCDHB4 | 0.0196 |
| LAMP2 | 0.01966 |
| MTMR8 | 0.01968 |
| RGMB | 0.01971 |
| CXCL11 | 0.01974 |
| SCAP | 0.01976 |
| BET1 | 0.01981 |
| KLHL36 | 0.01981 |
| IKZF1 | 0.01986 |
| RRN3 | 0.01986 |
| UPRT | 0.01992 |
| RAB40C | 0.02005 |
| ZNF823 | 0.02005 |
| RCOR2 | 0.02008 |
| DHDDS | 0.02011 |
| JAK3 | 0.02011 |
| KL | 0.02016 |
| NDUFA12 | 0.02019 |
| PDCD1LG2 | 0.02022 |
| METTL9 | 0.02023 |
| DCLRE1B | 0.02027 |
| GFER | 0.02034 |
| PPP1R18 | 0.02038 |
| RALY | 0.02042 |
| PTP4A2 | 0.02054 |
| ITFG1 | 0.02055 |
| CDC40 | 0.02064 |
| MOCS3 | 0.02065 |
| ZMAT5 | 0.02065 |
| C11orf42 | 0.02077 |
| HLA-DPA1 | 0.02078 |
| MSANTD3 | 0.02079 |
| RNF146 | 0.02081 |
| UQCR11 | 0.02083 |
| DDX19A | 0.02089 |
| RAB8A | 0.02095 |
| ARHGEF9 | 0.02098 |
| ABHD17B | 0.02098 |
| DCTN5 | 0.02104 |
| GNS | 0.02112 |
| RETSAT | 0.02117 |
| SSBP2 | 0.02127 |
| MCM5 | 0.02129 |
| KYNU | 0.02132 |
| KPNA2 | 0.02133 |
| CTSA | 0.0214 |
| RFTN1 | 0.02143 |
| TEAD1 | 0.02143 |
| PARM1 | 0.02147 |
| HEXIM2 | 0.02149 |
| MX1 | 0.02153 |
| TMEM8A | 0.02162 |
| CD84 | 0.02168 |
| CCR2 | 0.02174 |
| PSMA1 | 0.02174 |
| GPR107 | 0.02176 |
| ETV6 | 0.02177 |
| GTF2H5 | 0.02184 |
| SCN2A | 0.02185 |
| CCR1 | 0.02186 |
| SLC13A5 | 0.02187 |
| KLF7 | 0.02191 |
| SIRT3 | 0.02191 |
| ENDOD1 | 0.02191 |
| TNFRSF10A | 0.02193 |
| LOXL1 | 0.02193 |
| CXCL12 | 0.02195 |
| FAR2 | 0.02205 |
| TLR8 | 0.02206 |
| MAGEA1 | 0.0221 |
| GPR34 | 0.02211 |
| LPAR5 | 0.02217 |
| ZFYVE1 | 0.02218 |
| TMEM230 | 0.02218 |
| PEX13 | 0.02219 |
| TRO | 0.02221 |
| TMED10 | 0.02224 |
| SLC9A7 | 0.02228 |
| BCL2L1 | 0.02233 |
| PIK3C2B | 0.02234 |
| CCNB2 | 0.02234 |
| PHYHD1 | 0.0224 |
| HLA-DOA | 0.02242 |
| KIF3C | 0.02246 |
| SYT1 | 0.02247 |
| DHX57 | 0.02248 |
| ATP4B | 0.0225 |
| RPUSD1 | 0.02256 |
| CUBN | 0.02258 |
| APOE | 0.02262 |
| SMIM14 | 0.02263 |
| ST6GALNAC3 | 0.02267 |
| DAPK3 | 0.02267 |
| GSKIP | 0.02274 |
| PDK2 | 0.02281 |
| MNDA | 0.02284 |
| FCGR3A | 0.02287 |
| EMC3 | 0.02291 |
| VAT1 | 0.02293 |
| DTD1 | 0.02295 |
| TMC8 | 0.02297 |
| HSD11B2 | 0.02298 |
| ZNF831 | 0.02301 |
| ICE2 | 0.02307 |
| AP2A2 | 0.02312 |
| SLC6A6 | 0.02317 |
| CPA4 | 0.02327 |
| PRF1 | 0.02328 |
| ZNF134 | 0.02331 |
| CASP6 | 0.02333 |
| FBXO3 | 0.02336 |
| CYFIP2 | 0.02341 |
| LRP10 | 0.02361 |
| DOLK | 0.02366 |
| PTGIR | 0.02366 |
| CIAO1 | 0.02372 |
| TSPAN32 | 0.02373 |
| RAD51C | 0.02376 |
| RBP7 | 0.02383 |
| DALRD3 | 0.02384 |
| CCL13 | 0.02389 |
| DPYD | 0.02394 |
| TLDC2 | 0.02394 |
| MLH1 | 0.02397 |
| MFSD1 | 0.02399 |
| S100A10 | 0.02404 |
| ACAA1 | 0.02404 |
| KRT2 | 0.02407 |
| METTL2A | 0.02414 |
| TMBIM4 | 0.02415 |
| PIGK | 0.02421 |
| MLX | 0.02422 |
| NECAP2 | 0.02427 |
| SLC7A14 | 0.02429 |
| COQ7 | 0.02432 |
| TSC2 | 0.02437 |
| ABHD17C | 0.02441 |
| EIF6 | 0.02441 |
| TMEM60 | 0.02443 |
| ZNF189 | 0.02445 |
| HPGDS | 0.02447 |
| PTPN9 | 0.02448 |
| NSMAF | 0.02451 |
| ARPP19 | 0.02453 |
| NKAP | 0.02454 |
| DOK1 | 0.02455 |
| DNAJC19 | 0.02461 |
| RHBDL2 | 0.0247 |
| PYGM | 0.02476 |
| APOBEC3B | 0.02483 |
| ARHGAP30 | 0.02485 |
| DDO | 0.02488 |
| PIK3R2 | 0.02489 |
| TBC1D5 | 0.02498 |
| ALKBH7 | 0.02498 |
| NCR3 | 0.02502 |
| TNS4 | 0.02503 |
| IRF8 | 0.02504 |
| POSTN | 0.02509 |
| EFCAB11 | 0.02514 |
| MMGT1 | 0.02517 |
| MSRB2 | 0.02521 |
| HAUS2 | 0.02521 |
| AP5S1 | 0.02525 |
| MAN2B2 | 0.02526 |
| BACH2 | 0.02531 |
| SLC38A2 | 0.02544 |
| RPAP1 | 0.02546 |
| GEMIN7 | 0.02546 |
| PTGDR | 0.02547 |
| CHMP6 | 0.02549 |
| DNAJC30 | 0.0255 |
| HFE | 0.02551 |
| SCML4 | 0.02557 |
| DNM1L | 0.02557 |
| CEPT1 | 0.02558 |
| LAMC1 | 0.02558 |
| GGNBP2 | 0.02561 |
| LPAR3 | 0.02563 |
| UGP2 | 0.02563 |
| PALLD | 0.02563 |
| NF2 | 0.02566 |
| ARAP3 | 0.02569 |
| CCDC170 | 0.02569 |
| CD28 | 0.02572 |
| NMNAT1 | 0.02578 |
| ADH5 | 0.0258 |
| IKZF3 | 0.02596 |
| TCEAL8 | 0.02599 |
| ANKRD55 | 0.02607 |
| IBTK | 0.0261 |
| TACC3 | 0.02613 |
| PSMD2 | 0.02615 |
| SATB2 | 0.02621 |
| TCEAL4 | 0.02624 |
| ATG7 | 0.02626 |
| PRKD1 | 0.02631 |
| DENR | 0.02635 |
| MAN1A1 | 0.02638 |
| DUSP6 | 0.02638 |
| E2F4 | 0.02638 |
| VASN | 0.02643 |
| COL11A2 | 0.02645 |
| SNCAIP | 0.02648 |
| PAK2 | 0.02653 |
| CTR9 | 0.02655 |
| ZNF442 | 0.02658 |
| DECR2 | 0.0266 |
| RASSF4 | 0.02667 |
| MYOM2 | 0.02671 |
| ACTN1 | 0.02672 |
| ZNFX1 | 0.02674 |
| ZBTB3 | 0.02675 |
| RBM15B | 0.02686 |
| STX1A | 0.02686 |
| SLC35A4 | 0.02697 |
| DOCK11 | 0.02698 |
| JDP2 | 0.027 |
| BAK1 | 0.02708 |
| NDUFAF3 | 0.0271 |
| PPP1R12A | 0.02713 |
| ARHGAP25 | 0.02715 |
| TRAK1 | 0.02719 |
| TYMP | 0.02721 |
| PRDM4 | 0.02723 |
| FGD6 | 0.02724 |
| CRYBB1 | 0.02737 |
| LYPLA2 | 0.02739 |
| POLR3K | 0.0274 |
| RPAP2 | 0.02746 |
| VPS39 | 0.02752 |
| PTPRU | 0.02756 |
| GAB3 | 0.02762 |
| ARHGAP26 | 0.02763 |
| METTL15 | 0.02764 |
| SLC51A | 0.02773 |
| ARMCX6 | 0.02775 |
| IGSF10 | 0.02779 |
| CETP | 0.02782 |
| ZNF276 | 0.02786 |
| ZBTB47 | 0.0279 |
| ADAL | 0.02794 |
| B3GNT9 | 0.02796 |
| MEGF10 | 0.028 |
| DOK3 | 0.02808 |
| KPNA3 | 0.02818 |
| SCYL2 | 0.02822 |
| LRRC25 | 0.02824 |
| PTPN11 | 0.02828 |
| PTPN7 | 0.02829 |
| LRIG2 | 0.0283 |
| NAT1 | 0.02843 |
| ITGA3 | 0.02843 |
| CIDEB | 0.02846 |
| SMG8 | 0.02848 |
| CYSTM1 | 0.02851 |
| AXIN2 | 0.02857 |
| STT3B | 0.0286 |
| CDK2AP1 | 0.02863 |
| RSU1 | 0.02866 |
| ZNF669 | 0.0287 |
| FBXW5 | 0.02875 |
| CCDC43 | 0.02881 |
| S100A6 | 0.02882 |
| NFKBIB | 0.02882 |
| LSM6 | 0.02884 |
| S100A3 | 0.02901 |
| LTB4R | 0.02901 |
| CD8A | 0.02903 |
| NAPRT | 0.02908 |
| HIST1H2BC | 0.02915 |
| RFK | 0.02916 |
| TOB2 | 0.02921 |
| COL18A1 | 0.02928 |
| GLRX5 | 0.0293 |
| KLRG1 | 0.02938 |
| DLG1 | 0.02945 |
| ST8SIA4 | 0.02945 |
| RASA1 | 0.02948 |
| WDFY4 | 0.0297 |
| PAM | 0.02972 |
| FAM86C1 | 0.02973 |
| ANKRD6 | 0.02976 |
| CATSPERB | 0.02982 |
| SESTD1 | 0.03002 |
| POLM | 0.03003 |
| SNX1 | 0.03006 |
| SLC44A4 | 0.03007 |
| HEBP1 | 0.03013 |
| GPD1L | 0.03018 |
| SLC10A7 | 0.03024 |
| ZNF582 | 0.03026 |
| IFI44L | 0.03028 |
| PFDN1 | 0.03029 |
| PDP1 | 0.03035 |
| RPL26L1 | 0.03036 |
| HTR1E | 0.03039 |
| DGCR6L | 0.03043 |
| ANXA11 | 0.03045 |
| BCL7C | 0.03049 |
| SLC35B4 | 0.03051 |
| AGPAT1 | 0.03057 |
| EFTUD2 | 0.03057 |
| STX12 | 0.03061 |
| MYO7A | 0.03063 |
| ALKBH4 | 0.03064 |
| CPPED1 | 0.03069 |
| CAPN7 | 0.03071 |
| STEAP1 | 0.03076 |
| GDE1 | 0.03078 |
| POC1A | 0.03083 |
| ZNF574 | 0.03084 |
| TBC1D10A | 0.03085 |
| TAOK3 | 0.03088 |
| STARD5 | 0.03088 |
| IGFBP4 | 0.03105 |
| IFI16 | 0.03118 |
| TFEB | 0.03122 |
| DNAJB5 | 0.03123 |
| COPS7A | 0.03127 |
| SLC35E1 | 0.03128 |
| SCAMP2 | 0.03135 |
| LASP1 | 0.03137 |
| SLC9A2 | 0.0314 |
| S1PR1 | 0.03145 |
| FER | 0.03151 |
| TMIGD2 | 0.03155 |
| ZNF571 | 0.03165 |
| CSRP2 | 0.0317 |
| ALDH9A1 | 0.03171 |
| DDX20 | 0.03172 |
| PAGE2 | 0.03172 |
| CASC4 | 0.03173 |
| TWIST1 | 0.03174 |
| PINK1 | 0.03178 |
| SAC3D1 | 0.03184 |
| WDR53 | 0.03191 |
| ZBTB45 | 0.03194 |
| COL5A2 | 0.032 |
| CLEC1A | 0.03201 |
| BAX | 0.03202 |
| SMYD5 | 0.03213 |
| TSPAN1 | 0.03216 |
| NCF1 | 0.03219 |
| MYL12A | 0.03221 |
| SERPINB1 | 0.03222 |
| WDR72 | 0.03227 |
| AUTS2 | 0.03229 |
| KDM1B | 0.03236 |
| COPRS | 0.03236 |
| TDO2 | 0.03237 |
| IPO8 | 0.03238 |
| IFI27 | 0.03245 |
| RALB | 0.0325 |
| PPP1R37 | 0.03251 |
| DDX24 | 0.03253 |
| HLA-DMB | 0.03254 |
| TMEM33 | 0.03262 |
| RMI2 | 0.03262 |
| C22orf39 | 0.03275 |
| RAB3GAP1 | 0.03278 |
| USP53 | 0.0328 |
| LYPD6 | 0.03281 |
| SPCS1 | 0.03284 |
| MGRN1 | 0.03286 |
| CMC2 | 0.03287 |
| CR1 | 0.0329 |
| FAM174A | 0.03291 |
| SEC61B | 0.03291 |
| TRMT5 | 0.03299 |
| PPHLN1 | 0.03303 |
| SLC35C1 | 0.03307 |
| TBC1D10B | 0.0331 |
| VPS4A | 0.03311 |
| WARS | 0.03315 |
| REST | 0.03317 |
| TSG101 | 0.03318 |
| MYL12B | 0.03321 |
| APBA1 | 0.03322 |
| SIGLEC1 | 0.03325 |
| EIF2B2 | 0.03332 |
| VPS28 | 0.03336 |
| SLC39A1 | 0.03337 |
| SLC40A1 | 0.03339 |
| KLHL6 | 0.03343 |
| AKT1S1 | 0.03346 |
| NOA1 | 0.03349 |
| TKTL1 | 0.03356 |
| RNF14 | 0.03357 |
| EML1 | 0.03364 |
| CASP10 | 0.03366 |
| TMPRSS11A | 0.03369 |
| MAVS | 0.03371 |
| SNRK | 0.03373 |
| XKR8 | 0.03383 |
| SIRPG | 0.03384 |
| CRLF3 | 0.03385 |
| UBAC1 | 0.03387 |
| CLIC1 | 0.03391 |
| METTL7A | 0.03393 |
| UBTD2 | 0.03396 |
| ZNF230 | 0.03396 |
| CLIP3 | 0.03401 |
| SMS | 0.03412 |
| COG7 | 0.03414 |
| GCSAM | 0.03423 |
| UCP1 | 0.03432 |
| AGAP2 | 0.03435 |
| F2R | 0.03444 |
| CCRL2 | 0.03445 |
| ROCK1 | 0.03449 |
| PRKX | 0.03452 |
| DMD | 0.03452 |
| RGS19 | 0.03455 |
| CPSF3 | 0.03458 |
| LRRC6 | 0.03459 |
| RAB14 | 0.03464 |
| THSD1 | 0.03464 |
| FGD5 | 0.03465 |
| TERF2IP | 0.03468 |
| COMMD2 | 0.0347 |
| HK3 | 0.03477 |
| MOSPD3 | 0.03477 |
| S1PR4 | 0.03477 |
| PIGB | 0.03479 |
| NEFH | 0.03489 |
| MED12 | 0.03493 |
| TMEM160 | 0.03499 |
| TMEM251 | 0.03503 |
| SAR1B | 0.03506 |
| HINT3 | 0.03511 |
| NSMCE1 | 0.03511 |
| CYP39A1 | 0.03515 |
| TM9SF4 | 0.03518 |
| TMEM108 | 0.03525 |
| TUFT1 | 0.03531 |
| AKTIP | 0.03531 |
| NUDC | 0.03534 |
| TBCK | 0.03543 |
| PRKAR1A | 0.03546 |
| CDC42 | 0.03547 |
| ARHGAP35 | 0.03549 |
| GSK3A | 0.0355 |
| EPS8 | 0.03552 |
| CALCOCO2 | 0.03557 |
| MOB2 | 0.03562 |
| KLF3 | 0.03563 |
| TLCD1 | 0.03566 |
| CTSD | 0.03567 |
| PRR11 | 0.03571 |
| LTB4R2 | 0.03572 |
| AP1M1 | 0.03573 |
| AXL | 0.03574 |
| ADCY7 | 0.03576 |
| DNAJC13 | 0.03587 |
| ETV5 | 0.03589 |
| CENPBD1 | 0.03589 |
| TXN2 | 0.0359 |
| DSEL | 0.03597 |
| SVIL | 0.03608 |
| B3GNT8 | 0.03609 |
| ABCF2 | 0.0361 |
| FZD2 | 0.03613 |
| EBI3 | 0.03613 |
| AHR | 0.03615 |
| SLITRK6 | 0.03615 |
| HADHB | 0.03616 |
| PNRC2 | 0.03621 |
| UNC45A | 0.03621 |
| ZNF519 | 0.03623 |
| HSPH1 | 0.03625 |
| STAB1 | 0.03629 |
| RPUSD2 | 0.03631 |
| ZNF549 | 0.03635 |
| RHOG | 0.03646 |
| RSL1D1 | 0.03665 |
| RNF149 | 0.03667 |
| GNB2 | 0.03671 |
| TRIM56 | 0.0368 |
| C16orf72 | 0.03682 |
| CSRP1 | 0.03685 |
| TFDP2 | 0.03689 |
| SH3BP2 | 0.03694 |
| BTBD2 | 0.03697 |
| TMEM109 | 0.037 |
| RASGRP1 | 0.037 |
| PPL | 0.037 |
| AMER1 | 0.03707 |
| CCDC68 | 0.03709 |
| GOPC | 0.0371 |
| CNP | 0.0371 |
| NDUFAB1 | 0.03711 |
| RORB | 0.03717 |
| ZNF559 | 0.0372 |
| KRT75 | 0.03723 |
| PSMG2 | 0.03726 |
| TMEM186 | 0.03737 |
| TREM2 | 0.03743 |
| IFITM5 | 0.03747 |
| CNTNAP5 | 0.03749 |
| MVK | 0.03754 |
| MT1G | 0.03757 |
| PHLDA3 | 0.03758 |
| MRPS11 | 0.03758 |
| CCDC115 | 0.03761 |
| ITGBL1 | 0.03762 |
| CDKN1A | 0.03763 |
| HOXA6 | 0.03764 |
| CIB2 | 0.03764 |
| TUBG1 | 0.03764 |
| MSR1 | 0.03766 |
| MASP1 | 0.03769 |
| THOC6 | 0.03773 |
| ERAL1 | 0.03775 |
| DRAM1 | 0.03779 |
| PDE9A | 0.03783 |
| PRPF31 | 0.03788 |
| RPS27L | 0.03789 |
| C7orf50 | 0.03794 |
| CRYBA2 | 0.03799 |
| AP3B2 | 0.0381 |
| PKD1L3 | 0.03814 |
| CCM2 | 0.03823 |
| DCBLD2 | 0.03839 |
| PYCARD | 0.0384 |
| COL13A1 | 0.03843 |
| TRIM32 | 0.03851 |
| TFE3 | 0.03856 |
| ZMYM3 | 0.03856 |
| BCL9L | 0.03866 |
| CMTM6 | 0.03872 |
| SLC9A9 | 0.03876 |
| TNPO1 | 0.03882 |
| CEP41 | 0.03883 |
| CFHR4 | 0.03884 |
| JOSD2 | 0.03887 |
| CHRNA1 | 0.03891 |
| MAP1A | 0.03902 |
| FTL | 0.03903 |
| SCN5A | 0.0391 |
| TM9SF2 | 0.03918 |
| ARFIP1 | 0.03932 |
| CALM3 | 0.03933 |
| EPG5 | 0.03942 |
| RP2 | 0.0395 |
| CCDC92 | 0.0395 |
| OAF | 0.03951 |
| CXCL10 | 0.03954 |
| CCDC174 | 0.03956 |
| EMC10 | 0.03958 |
| ARHGAP44 | 0.03959 |
| PYGB | 0.03959 |
| PTRH2 | 0.03966 |
| PSMB6 | 0.03967 |
| LRP11 | 0.03971 |
| GABARAPL2 | 0.03974 |
| C15orf61 | 0.03978 |
| PANK2 | 0.03983 |
| 2-Sep | 0.03984 |
| FAM222B | 0.03984 |
| CCDC117 | 0.03999 |
| PRDM16 | 0.04011 |
| NFE2L2 | 0.04013 |
| YKT6 | 0.04016 |
| COIL | 0.0402 |
| ABCC12 | 0.04021 |
| HIF1A | 0.04026 |
| NUP98 | 0.04029 |
| GLP2R | 0.04031 |
| TFAP4 | 0.04034 |
| SGCB | 0.04036 |
| INTS5 | 0.04037 |
| UCK1 | 0.04045 |
| CXCR6 | 0.04049 |
| OGDH | 0.04051 |
| ENPEP | 0.04053 |
| NSDHL | 0.04055 |
| ATG2B | 0.04058 |
| CYBA | 0.04059 |
| ZNF780B | 0.04071 |
| NSFL1C | 0.04077 |
| ARSJ | 0.04078 |
| HIST1H4I | 0.04078 |
| MC1R | 0.04078 |
| PNPO | 0.04087 |
| LZIC | 0.04092 |
| OGG1 | 0.04093 |
| DCTN2 | 0.04094 |
| KLRD1 | 0.04116 |
| PIK3AP1 | 0.04121 |
| SPR | 0.04125 |
| CETN2 | 0.04136 |
| THEMIS2 | 0.04137 |
| SMARCAL1 | 0.04143 |
| LAMTOR1 | 0.04143 |
| ZNF215 | 0.04149 |
| RBFA | 0.04153 |
| MORC4 | 0.04155 |
| NFATC2 | 0.04158 |
| BAG6 | 0.0416 |
| UBN1 | 0.04162 |
| SEC13 | 0.04172 |
| ELP5 | 0.04176 |
| THY1 | 0.04181 |
| LSP1 | 0.04184 |
| PON1 | 0.04189 |
| POLR2L | 0.04208 |
| MON1A | 0.04212 |
| TSPO2 | 0.04222 |
| RPUSD3 | 0.04229 |
| ASB6 | 0.04229 |
| RBPJ | 0.0423 |
| SWAP70 | 0.04232 |
| RNF34 | 0.04235 |
| TCTEX1D2 | 0.04241 |
| MPV17L2 | 0.04243 |
| ATP6V0A2 | 0.04248 |
| TSN | 0.04249 |
| ANKRD29 | 0.04251 |
| SYCE1 | 0.04253 |
| AGFG2 | 0.04261 |
| DKKL1 | 0.04265 |
| DNASE1L1 | 0.04273 |
| ZNF841 | 0.04274 |
| ACSF2 | 0.04288 |
| ZWILCH | 0.04293 |
| MYH13 | 0.04296 |
| PNPLA2 | 0.04302 |
| KLRB1 | 0.0431 |
| MPV17 | 0.04316 |
| NEK11 | 0.0432 |
| KCNK9 | 0.04322 |
| BNC2 | 0.04323 |
| FAM214B | 0.04326 |
| LIN7A | 0.04326 |
| MYH9 | 0.04327 |
| WFDC10B | 0.04331 |
| PAG1 | 0.04334 |
| FMNL1 | 0.04334 |
| ZBTB7A | 0.04338 |
| GNPTG | 0.0434 |
| TULP3 | 0.04343 |
| LRIF1 | 0.04346 |
| ACSL4 | 0.04346 |
| TSHZ3 | 0.04346 |
| VMP1 | 0.04357 |
| DNAJC14 | 0.04366 |
| PDCL | 0.04373 |
| VCAM1 | 0.04374 |
| ACTR3 | 0.04379 |
| TTLL5 | 0.04391 |
| VPS52 | 0.04397 |
| PLIN3 | 0.04404 |
| ANXA6 | 0.04408 |
| LOXL4 | 0.04415 |
| ZNF513 | 0.04416 |
| THTPA | 0.04418 |
| DLX3 | 0.04426 |
| CPT2 | 0.04438 |
| KLHL18 | 0.0445 |
| GPSM1 | 0.04457 |
| COPS4 | 0.0446 |
| LYZ | 0.04468 |
| SFXN5 | 0.04481 |
| FCRLB | 0.0449 |
| TFPI | 0.0449 |
| CAPSL | 0.04492 |
| NDUFA6 | 0.04496 |
| CIAPIN1 | 0.04498 |
| PDSS2 | 0.04502 |
| LIN52 | 0.04503 |
| PPEF1 | 0.04504 |
| MYL6 | 0.04516 |
| TBXAS1 | 0.04523 |
| IL7 | 0.04525 |
| PANX3 | 0.04527 |
| RHOBTB2 | 0.04529 |
| BCL10 | 0.04532 |
| WDR1 | 0.04533 |
| THADA | 0.04536 |
| RPS6KA2 | 0.04538 |
| DNTTIP1 | 0.04541 |
| SPATA12 | 0.04542 |
| FBXL5 | 0.04542 |
| AKAP11 | 0.04547 |
| POLR2I | 0.04564 |
| CLEC7A | 0.04565 |
| XYLT2 | 0.0457 |
| RNPC3 | 0.04573 |
| GMPR | 0.04574 |
| ABHD5 | 0.04587 |
| SPRY3 | 0.04588 |
| MTMR14 | 0.04593 |
| ARL2BP | 0.04601 |
| ASNA1 | 0.04601 |
| KANSL1 | 0.04607 |
| KCNMB1 | 0.04611 |
| IL2RG | 0.04615 |
| GNPDA1 | 0.04618 |
| OCIAD2 | 0.04621 |
| TBC1D2 | 0.04623 |
| CALCRL | 0.04624 |
| COA7 | 0.04627 |
| YY1 | 0.0463 |
| SRP14 | 0.0463 |
| MICAL1 | 0.04643 |
| TEX2 | 0.04646 |
| CHCHD2 | 0.04648 |
| ZNF492 | 0.0465 |
| MAATS1 | 0.04652 |
| DENND4A | 0.04654 |
| IFI6 | 0.04656 |
| SCUBE2 | 0.04662 |
| GNA15 | 0.04662 |
| NDUFB10 | 0.04666 |
| SLC39A3 | 0.04666 |
| SPARCL1 | 0.04669 |
| MALSU1 | 0.04672 |
| NAPA | 0.04675 |
| MGST1 | 0.04676 |
| LCP1 | 0.04678 |
| TSGA13 | 0.0468 |
| L3MBTL2 | 0.04686 |
| ZNF253 | 0.04688 |
| TMCO4 | 0.04692 |
| ARHGAP1 | 0.047 |
| PIP4K2B | 0.047 |
| SEMA6A | 0.04701 |
| MAFK | 0.04702 |
| IL10RA | 0.04702 |
| SYN3 | 0.04703 |
| HTRA1 | 0.04704 |
| GNG2 | 0.04705 |
| ZNF784 | 0.04717 |
| RAB35 | 0.0472 |
| NMT1 | 0.04724 |
| LAMB1 | 0.04728 |
| QDPR | 0.04734 |
| SLCO4C1 | 0.04735 |
| SOX4 | 0.04741 |
| MOCS2 | 0.04742 |
| MLPH | 0.04745 |
| GALNT10 | 0.04753 |
| RNASEH2A | 0.04765 |
| TSPAN3 | 0.04766 |
| STXBP5 | 0.0478 |
| ITGA9 | 0.04782 |
| ZFP36L1 | 0.04784 |
| FTO | 0.04784 |
| GPR174 | 0.04803 |
| DHX8 | 0.04808 |
| RIPPLY2 | 0.04813 |
| ABHD12 | 0.04813 |
| CTSS | 0.04817 |
| BAIAP2L1 | 0.0482 |
| SNRNP25 | 0.04826 |
| TBL3 | 0.04837 |
| PHLDA2 | 0.04842 |
| LAPTM5 | 0.04849 |
| ZNF575 | 0.04854 |
| PODN | 0.04855 |
| ZNF136 | 0.04856 |
| CTC1 | 0.04857 |
| SF3B5 | 0.04859 |
| RUNX1T1 | 0.04861 |
| POFUT1 | 0.04867 |
| LETM1 | 0.04883 |
| TMC6 | 0.04885 |
| KLHL12 | 0.04893 |
| RGS5 | 0.04895 |
| POLR3F | 0.049 |
| GTF2H1 | 0.04908 |
| ZNF280A | 0.04909 |
| CDKL5 | 0.04911 |
| MAP2 | 0.04914 |
| SLC25A30 | 0.04919 |
| JADE2 | 0.04929 |
| PAF1 | 0.04931 |
| ITGB4 | 0.04933 |
| AAR2 | 0.04936 |
| HESX1 | 0.04947 |
| MTFMT | 0.0496 |
| MCF2L2 | 0.04968 |
| ALDH16A1 | 0.04981 |
| PLCD3 | 0.04989 |
| LRRC8A | 0.0499 |
| AKAP6 | 0.04997 |

Symbol: gene symbol

Pvalue: P value
