# Supplementary material for: KIF21B Expression in Osteosarcoma and Its Regulatory Effect on Osteosarcoma Cell Proliferation and Apoptosis Through the PI3K/AKT Pathway
Source: Front Oncol. 2021 Jan 28;10:606765. doi: 10.3389/fonc.2020.606765 (PMC7879035; doi:10.3389/fonc.2020.606765)
Supplement: Supplementary file 4 [file Table_3.docx]

Supplementary Table 3 Univariable Cox regression analysis for overall survival

| Symbol | HR | Z | Pvalue |
| --- | --- | --- | --- |
| NMNAT1 | 0.527165 | -3.4322 | 0.000599 |
| PGD | 0.515368 | -3.10927 | 0.001875 |
| UBIAD1 | 0.518317 | -2.78597 | 0.005337 |
| MFN2 | 0.571511 | -2.58894 | 0.009627 |
| TMEM51 | 0.613068 | -2.68986 | 0.007148 |
| EFHD2 | 0.539429 | -2.78885 | 0.00529 |
| SZRD1 | 0.535848 | -2.60177 | 0.009275 |
| CAPZB | 0.541728 | -2.94708 | 0.003208 |
| TMCO4 | 0.596415 | -2.862 | 0.00421 |
| ECE1 | 0.562744 | -2.79213 | 0.005236 |
| HMGCL | 0.61207 | -2.59672 | 0.009412 |
| SH3BGRL3 | 0.584051 | -3.16147 | 0.00157 |
| PIGV | 0.564834 | -2.70019 | 0.00693 |
| PTP4A2 | 0.577529 | -2.59752 | 0.00939 |
| BSDC1 | 0.488151 | -2.63176 | 0.008494 |
| TRIM62 | 0.539933 | -3.43102 | 0.000601 |
| GJA4 | 0.715046 | -2.65502 | 0.00793 |
| PSMB2 | 0.503878 | -2.95976 | 0.003079 |
| C1orf216 | 0.525547 | -3.01654 | 0.002557 |
| TMEM53 | 0.579091 | -3.30391 | 0.000953 |
| MMACHC | 0.603592 | -2.68591 | 0.007233 |
| PODN | 0.703017 | -3.52639 | 0.000421 |
| TCEANC2 | 0.565324 | -2.58702 | 0.009681 |
| GNG12 | 0.69239 | -2.97034 | 0.002975 |
| PIGK | 0.609788 | -2.62908 | 0.008562 |
| IFI44 | 0.706778 | -2.89916 | 0.003742 |
| BCL10 | 0.496729 | -3.20333 | 0.001358 |
| ZNHIT6 | 0.585815 | -2.60109 | 0.009293 |
| GBP3 | 0.670662 | -3.28699 | 0.001013 |
| GBP2 | 0.724437 | -3.11094 | 0.001865 |
| GBP5 | 0.778262 | -2.87836 | 0.003997 |
| RPAP2 | 0.550282 | -2.87283 | 0.004068 |
| FNBP1L | 0.638648 | -2.73656 | 0.006209 |
| CNN3 | 0.630038 | -2.84522 | 0.004438 |
| VCAM1 | 0.714865 | -3.28343 | 0.001026 |
| EXTL2 | 0.597248 | -2.59955 | 0.009335 |
| DPH5 | 0.628447 | -2.613 | 0.008975 |
| KCNA3 | 0.668345 | -2.96798 | 0.002998 |
| CTTNBP2NL | 0.643499 | -2.73525 | 0.006233 |
| OLFML3 | 0.72171 | -2.99058 | 0.002784 |
| TBX15 | 0.690509 | -2.5924 | 0.009531 |
| GJA5 | 0.688458 | -4.05275 | 5.06E-05 |
| MLLT11 | 0.651764 | -2.89324 | 0.003813 |
| S100A11 | 0.578898 | -2.93806 | 0.003303 |
| S100A6 | 0.636229 | -2.80387 | 0.005049 |
| S100A3 | 0.674819 | -3.20353 | 0.001358 |
| FCER1G | 0.73769 | -2.90716 | 0.003647 |
| FCGR2B | 0.733877 | -2.90785 | 0.003639 |
| NMNAT2 | 0.662712 | -3.57761 | 0.000347 |
| KIF21B | 0.635736 | -3.02584 | 0.002479 |
| PHLDA3 | 0.679966 | -2.75813 | 0.005813 |
| CSRP1 | 0.574917 | -2.58853 | 0.009639 |
| NUAK2 | 0.664324 | -2.64827 | 0.00809 |
| OR2M5 | 1.505045 | 2.58542 | 0.009726 |
| KIF3C | 0.609354 | -2.59709 | 0.009402 |
| SLC35F6 | 0.53054 | -2.98471 | 0.002838 |
| CGREF1 | 1.415333 | 3.122193 | 0.001795 |
| NRBP1 | 0.59673 | -2.76513 | 0.00569 |
| PLB1 | 0.571957 | -3.20652 | 0.001343 |
| RTN4 | 0.610878 | -2.65608 | 0.007906 |
| ARHGAP25 | 0.704938 | -2.96597 | 0.003017 |
| EXOC6B | 0.49274 | -3.51058 | 0.000447 |
| MOB1A | 0.577692 | -2.6287 | 0.008571 |
| LBX2 | 0.675912 | -3.23574 | 0.001213 |
| DOK1 | 0.624255 | -2.59685 | 0.009408 |
| TMEM127 | 0.621333 | -2.77222 | 0.005567 |
| CIAO1 | 0.55744 | -2.73963 | 0.006151 |
| ARID5A | 0.60176 | -2.68356 | 0.007284 |
| GYPC | 0.668933 | -2.85714 | 0.004275 |
| PTPN18 | 0.564406 | -2.85676 | 0.00428 |
| POTEE | 0.651935 | -2.60679 | 0.00914 |
| FAP | 0.735801 | -3.10629 | 0.001895 |
| CASP10 | 0.676566 | -2.67071 | 0.007569 |
| ARMC9 | 0.573861 | -2.65661 | 0.007893 |
| B3GNT7 | 0.724187 | -2.59977 | 0.009329 |
| SNED1 | 0.669125 | -2.59484 | 0.009464 |
| SETMAR | 0.578678 | -2.81476 | 0.004881 |
| LMCD1 | 0.630547 | -2.90903 | 0.003625 |
| THUMPD3 | 0.566663 | -2.71294 | 0.006669 |
| TADA3 | 0.593315 | -2.86264 | 0.004201 |
| VHL | 0.551326 | -2.75854 | 0.005806 |
| ATG7 | 0.534357 | -2.98175 | 0.002866 |
| TMEM43 | 0.523973 | -3.34231 | 0.000831 |
| DPH3 | 0.536088 | -3.14516 | 0.00166 |
| GLB1 | 0.597293 | -2.97386 | 0.002941 |
| TRAK1 | 0.544119 | -3.18807 | 0.001432 |
| SCAP | 0.526197 | -2.9343 | 0.003343 |
| SHISA5 | 0.503943 | -3.57441 | 0.000351 |
| UQCRC1 | 0.547175 | -2.80232 | 0.005074 |
| MON1A | 0.57423 | -2.72082 | 0.006512 |
| GNAI2 | 0.567116 | -2.82051 | 0.004795 |
| HYAL2 | 0.620947 | -2.63912 | 0.008312 |
| CISH | 0.578301 | -3.28522 | 0.001019 |
| MAPKAPK3 | 0.624694 | -2.7737 | 0.005542 |
| ABHD14B | 0.603838 | -2.69733 | 0.00699 |
| TWF2 | 0.608434 | -2.81509 | 0.004876 |
| WNT5A | 0.611001 | -3.20163 | 0.001367 |
| ABHD6 | 0.640757 | -2.6728 | 0.007522 |
| C3orf14 | 0.621263 | -3.80051 | 0.000144 |
| KBTBD8 | 0.703785 | -2.71064 | 0.006715 |
| EIF4E3 | 0.606465 | -2.91063 | 0.003607 |
| DPPA2 | 1.352867 | 2.823509 | 0.00475 |
| ITGB5 | 0.67877 | -2.75319 | 0.005902 |
| NUDT16 | 0.48416 | -3.48591 | 0.00049 |
| TM4SF1 | 0.646991 | -2.83146 | 0.004634 |
| NCEH1 | 0.646462 | -2.74867 | 0.005984 |
| TPRG1 | 0.700653 | -2.70768 | 0.006775 |
| TCTEX1D2 | 0.639443 | -2.76709 | 0.005656 |
| WDR53 | 0.514712 | -3.06649 | 0.002166 |
| FGFRL1 | 0.620249 | -3.0012 | 0.002689 |
| TMEM129 | 0.546772 | -2.60922 | 0.009075 |
| SH3BP2 | 0.484482 | -3.29025 | 0.001001 |
| ADD1 | 0.485189 | -3.2605 | 0.001112 |
| WFS1 | 0.547724 | -2.82602 | 0.004713 |
| CCDC96 | 0.553618 | -3.59357 | 0.000326 |
| GRPEL1 | 0.515159 | -2.93357 | 0.003351 |
| ACOX3 | 0.541846 | -2.92624 | 0.003431 |
| WDR1 | 0.547682 | -3.11642 | 0.001831 |
| FBXL5 | 0.488107 | -3.55538 | 0.000377 |
| BST1 | 0.651578 | -2.82262 | 0.004763 |
| LGI2 | 0.733806 | -2.57851 | 0.009923 |
| RBPJ | 0.572593 | -2.94742 | 0.003204 |
| TBC1D19 | 0.603594 | -2.655 | 0.007931 |
| SMIM14 | 0.621298 | -2.73197 | 0.006296 |
| RBM47 | 0.691337 | -2.71887 | 0.006551 |
| GNPDA2 | 0.560064 | -2.79491 | 0.005191 |
| SCFD2 | 0.66787 | -2.61424 | 0.008943 |
| CXCL11 | 0.815641 | -2.59649 | 0.009418 |
| FRAS1 | 0.817231 | -2.71199 | 0.006688 |
| SPARCL1 | 0.667236 | -2.7445 | 0.00606 |
| HERC5 | 0.723464 | -2.66984 | 0.007589 |
| ADH5 | 0.625796 | -2.59699 | 0.009404 |
| PAPSS1 | 0.599073 | -2.61472 | 0.00893 |
| CASP6 | 0.591004 | -2.92132 | 0.003486 |
| ARSJ | 0.658234 | -3.69233 | 0.000222 |
| NUDT6 | 0.541561 | -2.71005 | 0.006727 |
| PCDH10 | 0.792545 | -2.91971 | 0.003504 |
| SCOC | 0.599508 | -2.60071 | 0.009303 |
| TLR2 | 0.733375 | -2.68253 | 0.007307 |
| ASIC5 | 0.668692 | -3.24755 | 0.001164 |
| TDO2 | 0.747268 | -3.77874 | 0.000158 |
| TLL1 | 0.756008 | -2.89557 | 0.003785 |
| PALLD | 0.700137 | -2.66513 | 0.007696 |
| GALNT7 | 0.703329 | -2.59649 | 0.009418 |
| ZFP42 | 1.257444 | 2.713755 | 0.006653 |
| CARD6 | 0.713488 | -2.61642 | 0.008886 |
| MOCS2 | 0.582672 | -2.58213 | 0.009819 |
| CD180 | 0.676703 | -3.1176 | 0.001823 |
| CKMT2 | 1.261625 | 2.689282 | 0.007161 |
| EDIL3 | 0.583552 | -4.23958 | 2.24E-05 |
| RGMB | 0.567567 | -2.77969 | 0.005441 |
| EFNA5 | 0.790167 | -2.61013 | 0.009051 |
| NREP | 0.573395 | -3.35758 | 0.000786 |
| KCNN2 | 0.668482 | -3.079 | 0.002077 |
| SEMA6A | 0.602225 | -2.59889 | 0.009353 |
| SNX2 | 0.570058 | -2.87442 | 0.004048 |
| ALDH7A1 | 0.57108 | -2.8705 | 0.004098 |
| SLC22A4 | 0.621141 | -2.61937 | 0.008809 |
| CTNNA1 | 0.548263 | -2.59124 | 0.009563 |
| SLC35A4 | 0.473318 | -3.03536 | 0.002403 |
| DPYSL3 | 0.647639 | -2.58654 | 0.009694 |
| ADRB2 | 0.663189 | -2.9844 | 0.002841 |
| ABLIM3 | 0.656593 | -2.91631 | 0.003542 |
| TIGD6 | 0.57847 | -2.95014 | 0.003176 |
| ZNF300 | 0.693689 | -3.17044 | 0.001522 |
| ANXA6 | 0.57 | -2.94738 | 0.003205 |
| GM2A | 0.631035 | -2.66279 | 0.00775 |
| CCNJL | 0.675037 | -2.75217 | 0.00592 |
| KCNMB1 | 0.70762 | -2.79507 | 0.005189 |
| PDLIM7 | 0.574142 | -2.6703 | 0.007578 |
| DOK3 | 0.636297 | -3.18446 | 0.00145 |
| RPP40 | 0.605507 | -2.5762 | 0.009989 |
| F13A1 | 0.729826 | -3.70415 | 0.000212 |
| C6orf52 | 0.64262 | -2.76459 | 0.005699 |
| HIVEP1 | 0.599068 | -2.80707 | 0.004999 |
| TPMT | 0.501641 | -3.21888 | 0.001287 |
| ACOT13 | 0.504603 | -2.97282 | 0.002951 |
| PPP1R18 | 0.457253 | -3.87542 | 0.000106 |
| CLIC1 | 0.55317 | -2.60869 | 0.009089 |
| EHMT2 | 0.51166 | -3.29721 | 0.000977 |
| BAK1 | 0.561156 | -2.75606 | 0.00585 |
| DEF6 | 0.686967 | -2.6346 | 0.008424 |
| TREM2 | 0.753398 | -2.58473 | 0.009745 |
| TNFRSF21 | 0.666497 | -3.39229 | 0.000693 |
| KHDC1L | 0.613824 | -2.73308 | 0.006274 |
| AKIRIN2 | 0.557087 | -2.68037 | 0.007354 |
| GRIK2 | 0.825719 | -2.94048 | 0.003277 |
| MICAL1 | 0.613289 | -2.64465 | 0.008178 |
| DDO | 0.778325 | -2.80066 | 0.0051 |
| TSPYL4 | 0.596539 | -2.79902 | 0.005126 |
| DSE | 0.698268 | -2.58001 | 0.00988 |
| NKAIN2 | 0.778057 | -2.67643 | 0.007441 |
| CENPW | 0.616888 | -2.68074 | 0.007346 |
| THEMIS | 0.720175 | -2.71167 | 0.006694 |
| MAP3K5 | 0.562884 | -3.49539 | 0.000473 |
| PEX7 | 0.579636 | -2.67823 | 0.007401 |
| IFNGR1 | 0.574334 | -3.05388 | 0.002259 |
| VTA1 | 0.54484 | -2.58113 | 0.009848 |
| PEX3 | 0.577402 | -2.73227 | 0.00629 |
| LRP11 | 0.628172 | -2.84812 | 0.004398 |
| RAET1G | 0.745152 | -2.74875 | 0.005982 |
| SNX9 | 0.623471 | -2.64669 | 0.008128 |
| GTF2H5 | 0.596949 | -2.763 | 0.005727 |
| KIF25 | 1.398833 | 2.698671 | 0.006962 |
| CARD11 | 0.730723 | -2.63029 | 0.008531 |
| ACTB | 0.530935 | -3.6023 | 0.000315 |
| AHR | 0.656366 | -2.63338 | 0.008454 |
| KLHL7 | 0.524319 | -3.06419 | 0.002183 |
| CCDC126 | 0.567025 | -2.99156 | 0.002776 |
| SKAP2 | 0.695555 | -2.60738 | 0.009124 |
| GPR141 | 0.666497 | -3.00786 | 0.002631 |
| NUDCD3 | 0.56451 | -2.76408 | 0.005708 |
| OGDH | 0.487622 | -3.26548 | 0.001093 |
| EGFR | 0.773591 | -2.78645 | 0.005329 |
| DNAJC30 | 0.612628 | -2.64666 | 0.008129 |
| LIMK1 | 0.569727 | -3.41052 | 0.000648 |
| CLIP2 | 0.538245 | -3.79466 | 0.000148 |
| RHBDD2 | 0.528043 | -2.82942 | 0.004663 |
| HGF | 0.729546 | -3.39382 | 0.000689 |
| MTERF1 | 0.553777 | -2.79754 | 0.005149 |
| CDK6 | 0.616085 | -4.08781 | 4.35E-05 |
| GNGT1 | 0.765214 | -2.81544 | 0.004871 |
| BRI3 | 0.610285 | -2.7341 | 0.006255 |
| AGFG2 | 0.523926 | -3.32129 | 0.000896 |
| MOSPD3 | 0.539658 | -2.76965 | 0.005612 |
| LAMB1 | 0.536242 | -2.99962 | 0.002703 |
| TES | 0.598436 | -3.43553 | 0.000591 |
| CAV2 | 0.667771 | -2.65942 | 0.007828 |
| CPA4 | 0.806195 | -2.62835 | 0.00858 |
| SLC35B4 | 0.551085 | -2.65983 | 0.007818 |
| CALD1 | 0.665581 | -3.03582 | 0.002399 |
| ZYX | 0.415261 | -4.28902 | 1.79E-05 |
| REPS2 | 0.689338 | -2.67625 | 0.007445 |
| YY2 | 0.616139 | -2.58325 | 0.009787 |
| ACOT9 | 0.587243 | -2.82388 | 0.004745 |
| APOO | 0.561357 | -2.76892 | 0.005624 |
| MAGEB6 | 0.62433 | -2.72843 | 0.006364 |
| DCAF8L1 | 0.549209 | -2.70787 | 0.006772 |
| MAGEB2 | 0.801443 | -2.90982 | 0.003616 |
| RP2 | 0.493342 | -3.29425 | 0.000987 |
| UBA1 | 0.487832 | -3.0034 | 0.00267 |
| USP11 | 0.478479 | -3.85299 | 0.000117 |
| WAS | 0.687079 | -3.01058 | 0.002607 |
| SUV39H1 | 0.590605 | -2.67372 | 0.007501 |
| TFE3 | 0.591528 | -2.68654 | 0.00722 |
| PRAF2 | 0.637281 | -2.67705 | 0.007427 |
| BMP15 | 1.794248 | 3.279573 | 0.00104 |
| PAGE2 | 0.832116 | -2.72068 | 0.006515 |
| MSN | 0.540789 | -3.05805 | 0.002228 |
| EFNB1 | 0.630736 | -2.93772 | 0.003306 |
| IGBP1 | 0.598697 | -2.82641 | 0.004707 |
| PDZD11 | 0.565902 | -3.08668 | 0.002024 |
| IL2RG | 0.732259 | -2.70639 | 0.006802 |
| MED12 | 0.592255 | -2.63914 | 0.008312 |
| UPRT | 0.475473 | -3.33474 | 0.000854 |
| MAGEE1 | 0.721173 | -3.40584 | 0.00066 |
| CYSLTR1 | 0.688917 | -2.74853 | 0.005986 |
| GPR174 | 0.721198 | -2.63911 | 0.008312 |
| NXF3 | 0.737532 | -2.88018 | 0.003974 |
| SLC25A53 | 0.551586 | -2.61097 | 0.009028 |
| MORC4 | 0.645801 | -3.63206 | 0.000281 |
| PRPS1 | 0.549117 | -2.65052 | 0.008037 |
| PSMD10 | 0.551283 | -3.16284 | 0.001562 |
| IL13RA2 | 0.794954 | -2.96113 | 0.003065 |
| SLC9A6 | 0.615651 | -2.71598 | 0.006608 |
| MAGEC2 | 0.852055 | -2.74946 | 0.005969 |
| MAMLD1 | 0.571267 | -3.04896 | 0.002296 |
| MTM1 | 0.622523 | -2.77151 | 0.00558 |
| GABRA3 | 0.834786 | -3.0423 | 0.002348 |
| MAGEA3 | 0.886062 | -2.95085 | 0.003169 |
| MAGEA1 | 0.837915 | -2.73054 | 0.006323 |
| G6PD | 0.528769 | -3.46056 | 0.000539 |
| C8orf48 | 0.707203 | -3.1237 | 0.001786 |
| NAT1 | 0.658995 | -2.74211 | 0.006105 |
| DOK2 | 0.698573 | -2.69984 | 0.006937 |
| RHOBTB2 | 0.537875 | -3.19779 | 0.001385 |
| TTI2 | 0.557477 | -2.67188 | 0.007543 |
| SNAI2 | 0.622374 | -2.93251 | 0.003362 |
| SDCBP | 0.646657 | -2.70464 | 0.006838 |
| TOX | 0.823524 | -2.59233 | 0.009533 |
| LY96 | 0.722923 | -2.68387 | 0.007277 |
| IL7 | 0.712954 | -3.16969 | 0.001526 |
| LRRC6 | 0.709708 | -2.79448 | 0.005198 |
| DOCK8 | 0.705607 | -2.58538 | 0.009727 |
| LURAP1L | 0.637118 | -3.80536 | 0.000142 |
| BNC2 | 0.746801 | -2.59868 | 0.009358 |
| DNAJB5 | 0.615765 | -3.04268 | 0.002345 |
| GLIPR2 | 0.580354 | -3.48943 | 0.000484 |
| ALDH1A1 | 0.756881 | -2.76063 | 0.005769 |
| ANXA1 | 0.631201 | -2.62355 | 0.008702 |
| TBC1D2 | 0.602118 | -2.698 | 0.006976 |
| TNFSF8 | 0.756378 | -2.84072 | 0.004501 |
| TRIM32 | 0.543763 | -2.63084 | 0.008518 |
| STOM | 0.548411 | -2.98276 | 0.002857 |
| MAPKAP1 | 0.456713 | -3.10638 | 0.001894 |
| CDK9 | 0.470503 | -2.89136 | 0.003836 |
| FPGS | 0.525182 | -2.6061 | 0.009158 |
| ENG | 0.539959 | -2.95287 | 0.003148 |
| TRUB2 | 0.485078 | -2.73409 | 0.006255 |
| SLC27A4 | 0.441848 | -3.54229 | 0.000397 |
| URM1 | 0.414333 | -3.20375 | 0.001356 |
| GLE1 | 0.517638 | -2.83748 | 0.004547 |
| TBC1D13 | 0.42026 | -3.29379 | 0.000988 |
| LRRC8A | 0.41428 | -3.16377 | 0.001557 |
| DOLK | 0.561162 | -2.68414 | 0.007272 |
| NUP188 | 0.519432 | -2.61531 | 0.008915 |
| DOLPP1 | 0.481057 | -2.90443 | 0.003679 |
| CRAT | 0.449137 | -3.79101 | 0.00015 |
| TOR1B | 0.444487 | -3.32197 | 0.000894 |
| TOR1A | 0.429231 | -3.18699 | 0.001438 |
| FNBP1 | 0.640415 | -2.59705 | 0.009403 |
| MED27 | 0.479188 | -3.6151 | 0.0003 |
| CACFD1 | 0.581491 | -3.09202 | 0.001988 |
| UBAC1 | 0.499132 | -2.61883 | 0.008823 |
| AP2A2 | 0.597133 | -2.68296 | 0.007297 |
| RHOG | 0.621839 | -2.59716 | 0.0094 |
| OR52B4 | 5.575825 | 3.054302 | 0.002256 |
| TRIM68 | 0.550383 | -3.47589 | 0.000509 |
| TRIM5 | 0.612291 | -2.59324 | 0.009508 |
| DCHS1 | 0.653231 | -2.90069 | 0.003723 |
| AKIP1 | 0.60769 | -2.67465 | 0.007481 |
| LYVE1 | 0.749251 | -2.61139 | 0.009017 |
| PARVA | 0.587466 | -2.84236 | 0.004478 |
| BTBD10 | 0.535477 | -3.00295 | 0.002674 |
| BBOX1 | 0.759814 | -3.12521 | 0.001777 |
| ARL14EP | 0.522631 | -2.80834 | 0.00498 |
| CAT | 0.576742 | -3.11807 | 0.00182 |
| COMMD9 | 0.605392 | -2.857 | 0.004277 |
| SLC35C1 | 0.611481 | -2.84864 | 0.004391 |
| AMBRA1 | 0.497927 | -2.79669 | 0.005163 |
| HARBI1 | 0.499565 | -3.27107 | 0.001071 |
| ARHGAP1 | 0.528293 | -3.313 | 0.000923 |
| ACP2 | 0.517077 | -3.10003 | 0.001935 |
| PTPMT1 | 0.589757 | -2.59496 | 0.00946 |
| UBE2L6 | 0.715035 | -2.80505 | 0.005031 |
| OR1S2 | 1.854543 | 2.621125 | 0.008764 |
| LPXN | 0.640261 | -2.84172 | 0.004487 |
| MS4A4A | 0.726453 | -2.94498 | 0.00323 |
| GAL | 1.251906 | 2.598573 | 0.009361 |
| FAM86C1 | 0.605492 | -2.58409 | 0.009764 |
| FOLR2 | 0.784772 | -2.69435 | 0.007053 |
| TAGLN | 0.631392 | -3.53838 | 0.000403 |
| OAF | 0.567268 | -2.93762 | 0.003307 |
| KCNJ5 | 0.689495 | -3.15891 | 0.001584 |
| C11orf45 | 0.721591 | -2.63353 | 0.00845 |
| IL2RA | 0.755294 | -3.0272 | 0.002468 |
| GATA3 | 0.728099 | -2.77468 | 0.005526 |
| MRC1 | 0.739879 | -2.62647 | 0.008627 |
| PLXDC2 | 0.695814 | -2.87234 | 0.004074 |
| GPR158 | 0.787724 | -3.21319 | 0.001313 |
| CXCL12 | 0.692929 | -3.53033 | 0.000415 |
| COL13A1 | 1.529775 | 3.116261 | 0.001832 |
| PRF1 | 0.75908 | -2.61235 | 0.008992 |
| ACTA2 | 0.647266 | -4.79763 | 1.61E-06 |
| FAS | 0.689291 | -3.07551 | 0.002101 |
| TLL2 | 0.694976 | -2.83449 | 0.00459 |
| PI4K2A | 0.597627 | -2.6688 | 0.007612 |
| LHPP | 0.583414 | -3.03592 | 0.002398 |
| DCP1B | 0.45787 | -3.27138 | 0.00107 |
| RHNO1 | 0.505631 | -3.70477 | 0.000212 |
| TULP3 | 0.518333 | -3.24399 | 0.001179 |
| TNFRSF1A | 0.509707 | -3.89716 | 9.73E-05 |
| LPAR5 | 0.748945 | -2.8619 | 0.004211 |
| C1R | 0.737381 | -2.82053 | 0.004794 |
| CD163 | 0.76144 | -2.7069 | 0.006792 |
| FOXJ2 | 0.612576 | -2.75304 | 0.005904 |
| C3AR1 | 0.746304 | -2.66085 | 0.007794 |
| KLRB1 | 0.734378 | -2.66507 | 0.007697 |
| ETV6 | 0.565494 | -2.93981 | 0.003284 |
| CREBL2 | 0.580884 | -3.04303 | 0.002342 |
| C12orf60 | 0.664339 | -2.71001 | 0.006728 |
| ARHGDIB | 0.683071 | -2.92046 | 0.003495 |
| EPS8 | 0.539951 | -3.87044 | 0.000109 |
| SLC38A4 | 0.824628 | -3.00378 | 0.002666 |
| PCED1B | 0.680628 | -3.13376 | 0.001726 |
| HDAC7 | 0.567772 | -3.09278 | 0.001983 |
| TUBA1A | 0.558437 | -3.71906 | 0.0002 |
| METTL7A | 0.627683 | -2.93207 | 0.003367 |
| ITGA5 | 0.635271 | -2.76383 | 0.005713 |
| NCKAP1L | 0.752712 | -2.58194 | 0.009825 |
| SUOX | 0.546329 | -2.86152 | 0.004216 |
| RAB3IP | 0.665331 | -2.99609 | 0.002735 |
| GLIPR1 | 0.646487 | -3.3725 | 0.000745 |
| PHLDA1 | 0.639128 | -2.67728 | 0.007422 |
| PAWR | 0.770342 | -3.04237 | 0.002347 |
| LIN7A | 0.666712 | -2.95989 | 0.003078 |
| EPYC | 0.832417 | -2.86922 | 0.004115 |
| NTN4 | 0.768019 | -2.76486 | 0.005695 |
| DRAM1 | 0.603086 | -2.86526 | 0.004167 |
| CMKLR1 | 0.708674 | -2.70687 | 0.006792 |
| CORO1C | 0.605953 | -2.65186 | 0.008005 |
| C12orf76 | 0.546999 | -2.66287 | 0.007748 |
| SH2B3 | 0.613394 | -2.65565 | 0.007916 |
| PEBP1 | 0.545725 | -2.68195 | 0.007319 |
| PXN | 0.543379 | -3.07222 | 0.002125 |
| RNF34 | 0.544924 | -3.15085 | 0.001628 |
| VPS37B | 0.585339 | -2.59191 | 0.009545 |
| CCDC92 | 0.568755 | -2.89534 | 0.003787 |
| CDK8 | 0.52558 | -2.72934 | 0.006346 |
| ALOX5AP | 0.749332 | -2.66177 | 0.007773 |
| HSPH1 | 0.584798 | -2.58925 | 0.009619 |
| POSTN | 0.774047 | -2.68703 | 0.007209 |
| GAS6 | 0.673297 | -2.7333 | 0.00627 |
| RNASE1 | 0.730842 | -2.59517 | 0.009454 |
| RNASE2 | 0.779398 | -2.59823 | 0.009371 |
| SLC7A7 | 0.675979 | -3.19306 | 0.001408 |
| LTB4R | 0.638106 | -2.67469 | 0.00748 |
| GZMB | 0.750768 | -3.13392 | 0.001725 |
| STXBP6 | 0.812926 | -2.6694 | 0.007599 |
| PRKD1 | 0.733789 | -2.63047 | 0.008527 |
| PTGDR | 0.734515 | -2.85741 | 0.004271 |
| ACTN1 | 0.58992 | -3.32899 | 0.000872 |
| SYNJ2BP | 0.570643 | -2.61993 | 0.008795 |
| PNMA1 | 0.589246 | -2.70821 | 0.006765 |
| NPC2 | 0.677571 | -2.86724 | 0.004141 |
| TMED10 | 0.558216 | -2.72505 | 0.006429 |
| VASH1 | 0.592212 | -3.0631 | 0.002191 |
| ALKBH1 | 0.484486 | -3.1359 | 0.001713 |
| ADCK1 | 0.60452 | -2.78348 | 0.005378 |
| GPR65 | 0.742565 | -2.60164 | 0.009278 |
| TTC8 | 0.549903 | -3.29818 | 0.000973 |
| EFCAB11 | 0.502707 | -2.96813 | 0.002996 |
| RIN3 | 0.612254 | -3.09004 | 0.002001 |
| TMEM251 | 0.57197 | -2.71189 | 0.00669 |
| DDX24 | 0.565046 | -2.67728 | 0.007422 |
| GSKIP | 0.522631 | -2.93625 | 0.003322 |
| CYFIP1 | 0.560057 | -3.02439 | 0.002491 |
| RASGRP1 | 0.586125 | -3.04631 | 0.002317 |
| VPS18 | 0.590714 | -2.98613 | 0.002825 |
| RPAP1 | 0.544464 | -2.68624 | 0.007226 |
| VPS39 | 0.58391 | -2.58163 | 0.009833 |
| SLC30A4 | 0.561109 | -2.78642 | 0.005329 |
| DTWD1 | 0.543326 | -2.78849 | 0.005295 |
| ANXA2 | 0.606679 | -2.74155 | 0.006115 |
| TLN2 | 0.668775 | -2.75704 | 0.005833 |
| TPM1 | 0.545309 | -3.44306 | 0.000575 |
| LACTB | 0.592908 | -2.85952 | 0.004243 |
| RAB8B | 0.63493 | -2.71877 | 0.006553 |
| SNX1 | 0.546898 | -2.99193 | 0.002772 |
| DIS3L | 0.537067 | -2.68351 | 0.007285 |
| ITGA11 | 0.779777 | -2.83326 | 0.004608 |
| GLCE | 0.460873 | -3.49382 | 0.000476 |
| THAP10 | 0.581865 | -3.25742 | 0.001124 |
| BBS4 | 0.531936 | -3.01098 | 0.002604 |
| LOXL1 | 0.719093 | -3.43595 | 0.00059 |
| PML | 0.493651 | -3.46642 | 0.000527 |
| SNX33 | 0.546456 | -2.61253 | 0.008988 |
| RCN2 | 0.507263 | -2.6463 | 0.008138 |
| ST20 | 0.603699 | -2.80524 | 0.005028 |
| STARD5 | 0.608436 | -2.71531 | 0.006621 |
| POLR3K | 0.609725 | -2.58511 | 0.009735 |
| SNRNP25 | 0.580812 | -2.80235 | 0.005073 |
| NPRL3 | 0.506379 | -3.39676 | 0.000682 |
| MRPL28 | 0.560956 | -2.75052 | 0.00595 |
| TMEM8A | 0.512726 | -2.87936 | 0.003985 |
| DECR2 | 0.552831 | -2.72765 | 0.006379 |
| PIGQ | 0.517773 | -2.80331 | 0.005058 |
| RAB40C | 0.411594 | -3.39677 | 0.000682 |
| WDR24 | 0.503909 | -3.06279 | 0.002193 |
| IFT140 | 0.55288 | -2.7707 | 0.005594 |
| FAHD1 | 0.575341 | -2.64895 | 0.008074 |
| MSRB1 | 0.564204 | -2.97174 | 0.002961 |
| NDUFB10 | 0.572891 | -2.80041 | 0.005104 |
| NOXO1 | 0.511257 | -2.84026 | 0.004508 |
| GFER | 0.470947 | -3.00206 | 0.002682 |
| FLYWCH2 | 0.551675 | -2.75602 | 0.005851 |
| TNFRSF12A | 0.600223 | -3.13076 | 0.001744 |
| ZNF597 | 0.639962 | -3.82942 | 0.000128 |
| CLUAP1 | 0.346558 | -4.13786 | 3.51E-05 |
| HMOX2 | 0.554125 | -2.78657 | 0.005327 |
| MGRN1 | 0.529062 | -2.89719 | 0.003765 |
| PPL | 0.786462 | -2.61475 | 0.008929 |
| NAGPA | 0.617725 | -2.6235 | 0.008703 |
| ABAT | 0.651881 | -3.07804 | 0.002084 |
| EMP2 | 0.618151 | -2.66333 | 0.007737 |
| NUBP1 | 0.489424 | -3.72556 | 0.000195 |
| RMI2 | 0.578834 | -3.03355 | 0.002417 |
| ERCC4 | 0.524403 | -3.50585 | 0.000455 |
| PARN | 0.471063 | -3.37661 | 0.000734 |
| BFAR | 0.491179 | -3.26652 | 0.001089 |
| NTAN1 | 0.480402 | -3.22839 | 0.001245 |
| NDE1 | 0.538549 | -2.74118 | 0.006122 |
| COQ7 | 0.521862 | -2.77131 | 0.005583 |
| GDE1 | 0.53025 | -2.82492 | 0.004729 |
| THUMPD1 | 0.592473 | -2.57694 | 0.009968 |
| LYRM1 | 0.463437 | -3.73557 | 0.000187 |
| METTL9 | 0.510799 | -3.06527 | 0.002175 |
| COG7 | 0.535711 | -2.72885 | 0.006356 |
| UBFD1 | 0.489445 | -2.89379 | 0.003806 |
| DCTN5 | 0.417301 | -3.58272 | 0.00034 |
| ARHGAP17 | 0.593116 | -2.59172 | 0.00955 |
| CORO1A | 0.706576 | -2.76218 | 0.005742 |
| TBC1D10B | 0.541086 | -2.82616 | 0.004711 |
| ITGAM | 0.706906 | -2.92126 | 0.003486 |
| CNEP1R1 | 0.591313 | -2.61456 | 0.008934 |
| FTO | 0.528713 | -2.93617 | 0.003323 |
| MMP2 | 0.713132 | -2.62715 | 0.00861 |
| OGFOD1 | 0.515907 | -2.90943 | 0.003621 |
| BBS2 | 0.53524 | -2.92725 | 0.00342 |
| SLC38A7 | 0.572968 | -2.97214 | 0.002957 |
| GOT2 | 0.527319 | -2.83571 | 0.004572 |
| CDH11 | 0.636011 | -2.7279 | 0.006374 |
| ATP6V0D1 | 0.57414 | -3.19074 | 0.001419 |
| TANGO6 | 0.519943 | -2.73884 | 0.006166 |
| CHTF8 | 0.548771 | -2.59198 | 0.009542 |
| TMED6 | 0.68174 | -2.67482 | 0.007477 |
| DDX19A | 0.532174 | -2.80755 | 0.004992 |
| SF3B3 | 0.540823 | -2.9779 | 0.002902 |
| TXNL4B | 0.5391 | -2.83278 | 0.004615 |
| BCAR1 | 0.5781 | -3.08241 | 0.002053 |
| MON1B | 0.605283 | -2.64137 | 0.008257 |
| MAF | 0.661922 | -2.7437 | 0.006075 |
| HSBP1 | 0.639287 | -2.68527 | 0.007247 |
| CHMP1A | 0.607911 | -2.74886 | 0.00598 |
| SPATA33 | 0.590833 | -2.9181 | 0.003522 |
| MC1R | 0.650755 | -2.7549 | 0.005871 |
| SRR | 0.599902 | -2.59155 | 0.009554 |
| TRAPPC1 | 0.672934 | -2.65824 | 0.007855 |
| PIK3R5 | 0.712198 | -2.67505 | 0.007472 |
| LLGL1 | 0.61342 | -2.7691 | 0.005621 |
| FLII | 0.598087 | -2.75619 | 0.005848 |
| TNFAIP1 | 0.580506 | -2.81688 | 0.004849 |
| FAM222B | 0.592316 | -2.60048 | 0.009309 |
| ERAL1 | 0.592848 | -2.70113 | 0.00691 |
| EVI2B | 0.753149 | -2.77647 | 0.005495 |
| CCL2 | 0.687003 | -3.61933 | 0.000295 |
| CCL8 | 0.770264 | -3.08303 | 0.002049 |
| SLFN5 | 0.631829 | -2.81212 | 0.004922 |
| ZNHIT3 | 0.520704 | -2.76282 | 0.00573 |
| LASP1 | 0.605077 | -2.8542 | 0.004315 |
| CNP | 0.472789 | -3.20072 | 0.001371 |
| RAB5C | 0.50758 | -3.16171 | 0.001568 |
| TUBG1 | 0.55219 | -2.84421 | 0.004452 |
| VPS25 | 0.580433 | -2.65998 | 0.007815 |
| DHX8 | 0.520171 | -2.66427 | 0.007716 |
| DUSP3 | 0.527209 | -3.09559 | 0.001964 |
| TMUB2 | 0.535707 | -2.7719 | 0.005573 |
| EFTUD2 | 0.489979 | -2.99132 | 0.002778 |
| DCAKD | 0.587562 | -2.73274 | 0.006281 |
| PLCD3 | 0.55882 | -3.29557 | 0.000982 |
| FMNL1 | 0.643392 | -2.81224 | 0.00492 |
| MRPL10 | 0.493918 | -3.21322 | 0.001313 |
| CALCOCO2 | 0.41135 | -3.31988 | 0.000901 |
| UBE2Z | 0.528864 | -2.62276 | 0.008722 |
| SNF8 | 0.542107 | -2.60599 | 0.009161 |
| PHB | 0.592135 | -2.60502 | 0.009187 |
| TAC4 | 1.391745 | 3.908932 | 9.27E-05 |
| ITGA3 | 0.646771 | -3.12282 | 0.001791 |
| PDK2 | 0.509448 | -3.31945 | 0.000902 |
| SPATA20 | 0.566885 | -3.25473 | 0.001135 |
| COIL | 0.516251 | -2.64783 | 0.008101 |
| MRPS23 | 0.500857 | -3.13135 | 0.00174 |
| DYNLL2 | 0.505692 | -3.38323 | 0.000716 |
| SUPT4H1 | 0.518729 | -3.23026 | 0.001237 |
| RAD51C | 0.493146 | -3.26747 | 0.001085 |
| CLTC | 0.537877 | -3.00515 | 0.002655 |
| VMP1 | 0.501652 | -3.21456 | 0.001306 |
| APPBP2 | 0.547819 | -2.9877 | 0.002811 |
| METTL2A | 0.54865 | -2.70903 | 0.006748 |
| PSMD12 | 0.535007 | -2.66622 | 0.007671 |
| WIPI1 | 0.637739 | -2.7111 | 0.006706 |
| UNC13D | 0.602898 | -2.87681 | 0.004017 |
| ACTG1 | 0.50529 | -2.8695 | 0.004111 |
| ARHGAP28 | 0.711917 | -3.43232 | 0.000598 |
| TUBB6 | 0.512705 | -3.05521 | 0.002249 |
| DSEL | 0.70844 | -3.50172 | 0.000462 |
| CCDC102B | 0.625915 | -2.79832 | 0.005137 |
| MBP | 0.72411 | -2.63761 | 0.008349 |
| SIRPG | 0.733911 | -3.16754 | 0.001537 |
| AP5S1 | 0.590517 | -2.61775 | 0.008851 |
| PRNP | 0.611309 | -2.81941 | 0.004811 |
| TMX4 | 0.585343 | -2.77454 | 0.005528 |
| SPTLC3 | 0.683409 | -2.6065 | 0.009147 |
| FLRT3 | 0.792003 | -3.16336 | 0.00156 |
| TM9SF4 | 0.560163 | -2.61421 | 0.008943 |
| KIF3B | 0.42019 | -3.36532 | 0.000765 |
| RALY | 0.504092 | -2.96315 | 0.003045 |
| DYNLRB1 | 0.573142 | -2.78076 | 0.005423 |
| PIGU | 0.553079 | -2.91998 | 0.003501 |
| ACSS2 | 0.460684 | -3.55152 | 0.000383 |
| AAR2 | 0.482808 | -2.97176 | 0.002961 |
| MYL9 | 0.693719 | -2.8521 | 0.004343 |
| TLDC2 | 0.698058 | -2.63415 | 0.008435 |
| BLCAP | 0.519057 | -3.08359 | 0.002045 |
| BPI | 0.765311 | -2.81724 | 0.004844 |
| EYA2 | 0.716683 | -3.03854 | 0.002377 |
| ZNFX1 | 0.573091 | -2.72702 | 0.006391 |
| CASS4 | 0.703277 | -3.07249 | 0.002123 |
| CNN2 | 0.683734 | -2.85513 | 0.004302 |
| GPX4 | 0.610231 | -2.59088 | 0.009573 |
| MOB3A | 0.589759 | -3.29217 | 0.000994 |
| S1PR4 | 0.707098 | -2.978 | 0.002901 |
| PIP5K1C | 0.426423 | -3.57641 | 0.000348 |
| DAPK3 | 0.547727 | -2.80814 | 0.004983 |
| ANKRD24 | 0.661754 | -2.64202 | 0.008241 |
| SH3GL1 | 0.495348 | -2.86926 | 0.004114 |
| DPP9 | 0.520445 | -2.69058 | 0.007133 |
| PLIN3 | 0.579833 | -2.98057 | 0.002877 |
| VAV1 | 0.743171 | -2.58832 | 0.009645 |
| PNPLA6 | 0.534207 | -2.92576 | 0.003436 |
| ZNF560 | 0.84052 | -2.61303 | 0.008974 |
| ZNF561 | 0.57119 | -2.72801 | 0.006372 |
| ICAM3 | 0.720036 | -2.59822 | 0.009371 |
| KANK2 | 0.606256 | -2.73196 | 0.006296 |
| TPM4 | 0.566545 | -3.19691 | 0.001389 |
| RAB8A | 0.585219 | -2.78337 | 0.00538 |
| AP1M1 | 0.530679 | -3.08336 | 0.002047 |
| DDA1 | 0.541451 | -2.75575 | 0.005856 |
| MAP1S | 0.500943 | -3.14208 | 0.001678 |
| PIK3R2 | 0.65468 | -2.79346 | 0.005215 |
| MPV17L2 | 0.597867 | -2.84166 | 0.004488 |
| ZNF101 | 0.454193 | -3.69229 | 0.000222 |
| ZNF93 | 0.637336 | -3.16073 | 0.001574 |
| PLEKHF1 | 0.649902 | -3.134 | 0.001724 |
| TSHZ3 | 0.569036 | -3.77776 | 0.000158 |
| RASGRP4 | 0.696072 | -2.92403 | 0.003455 |
| TIMM50 | 0.475734 | -2.95067 | 0.003171 |
| PSMC4 | 0.482575 | -3.4232 | 0.000619 |
| ZNF780B | 0.564581 | -3.67001 | 0.000243 |
| AKT2 | 0.549485 | -2.69265 | 0.007089 |
| BLVRB | 0.628675 | -3.26479 | 0.001095 |
| SHKBP1 | 0.578881 | -2.97083 | 0.00297 |
| ITPKC | 0.506077 | -3.15063 | 0.001629 |
| CYP2S1 | 0.709397 | -2.91526 | 0.003554 |
| HNRNPUL1 | 0.50826 | -2.72696 | 0.006392 |
| ZNF574 | 0.564462 | -2.70125 | 0.006908 |
| ZNF45 | 0.573339 | -2.78424 | 0.005365 |
| ZNF224 | 0.591607 | -2.85966 | 0.004241 |
| CLPTM1 | 0.503346 | -2.95349 | 0.003142 |
| PPP1R37 | 0.588679 | -2.63497 | 0.008414 |
| ERCC2 | 0.519646 | -2.81789 | 0.004834 |
| VASP | 0.496278 | -3.20281 | 0.001361 |
| AP2S1 | 0.591688 | -2.86351 | 0.00419 |
| EHD2 | 0.574894 | -3.5083 | 0.000451 |
| GRWD1 | 0.547216 | -2.57619 | 0.00999 |
| FTL | 0.636339 | -2.75135 | 0.005935 |
| CD37 | 0.701009 | -2.67348 | 0.007507 |
| AP2A1 | 0.552702 | -2.68975 | 0.007151 |
| ZNF175 | 0.554903 | -2.89232 | 0.003824 |
| SIGLEC14 | 0.811126 | -2.73609 | 0.006217 |
| FPR1 | 0.770468 | -2.8813 | 0.00396 |
| ZNF649 | 0.535724 | -2.96061 | 0.00307 |
| ZNF468 | 0.62311 | -2.60126 | 0.009288 |
| LAIR1 | 0.696472 | -2.92429 | 0.003452 |
| ZNF583 | 0.674008 | -3.18724 | 0.001436 |
| ZNF584 | 0.549696 | -2.76276 | 0.005731 |
| HSFY1 | 8.705388 | 3.423399 | 0.000618 |
| ATP6V1E1 | 0.648769 | -2.63619 | 0.008384 |
| PEX26 | 0.508842 | -3.20534 | 0.001349 |
| DGCR6L | 0.571726 | -2.66761 | 0.007639 |
| MED15 | 0.575596 | -2.7321 | 0.006293 |
| MAPK1 | 0.624154 | -2.62298 | 0.008716 |
| VPREB1 | 2.034151 | 2.773763 | 0.005541 |
| ZNF280A | 0.763695 | -2.61907 | 0.008817 |
| CRYBB1 | 0.671349 | -2.91351 | 0.003574 |
| TBC1D10A | 0.63066 | -2.76424 | 0.005706 |
| DUSP18 | 0.492019 | -3.58879 | 0.000332 |
| RNF185 | 0.5835 | -2.87276 | 0.004069 |
| YWHAH | 0.580526 | -2.91584 | 0.003547 |
| RTCB | 0.615055 | -2.65325 | 0.007972 |
| FBXO7 | 0.59262 | -2.59146 | 0.009557 |
| SYN3 | 0.709499 | -2.72631 | 0.006405 |
| HMOX1 | 0.749197 | -2.57735 | 0.009956 |
| APOL2 | 0.605273 | -3.06527 | 0.002175 |
| MYH9 | 0.60017 | -2.99319 | 0.002761 |
| TXN2 | 0.580943 | -2.79612 | 0.005172 |
| MFNG | 0.6715 | -2.57957 | 0.009892 |
| LGALS1 | 0.649247 | -3.14996 | 0.001633 |
| ANKRD54 | 0.543846 | -2.76353 | 0.005718 |
| APOBEC3B | 0.777405 | -3.31648 | 0.000912 |
| NAGA | 0.602865 | -2.8886 | 0.00387 |
| CYB5R3 | 0.60806 | -2.5972 | 0.009399 |
| PACSIN2 | 0.554185 | -2.63815 | 0.008336 |
| TTLL1 | 0.490068 | -3.74999 | 0.000177 |
| PARVG | 0.725142 | -2.61179 | 0.009007 |
| FBLN1 | 0.792973 | -2.68945 | 0.007157 |
| KRTAP13-3 | 1.71918 | 2.660815 | 0.007795 |
| KRTAP19-4 | 1.770355 | 2.697133 | 0.006994 |
| IFNAR2 | 0.590242 | -2.62099 | 0.008768 |
| WRB | 0.587314 | -2.69137 | 0.007116 |
| AGPAT3 | 0.583143 | -3.3324 | 0.000861 |

Symbol: gene symbol; HR: Hazard ratio;

Z: Z-score; Pvalue: P value
