# Supplementary material for: KIF21B Expression in Osteosarcoma and Its Regulatory Effect on Osteosarcoma Cell Proliferation and Apoptosis Through the PI3K/AKT Pathway
Source: Front Oncol. 2021 Jan 28;10:606765. doi: 10.3389/fonc.2020.606765 (PMC7879035; doi:10.3389/fonc.2020.606765)
Supplement: Supplementary file 5 [file Table_4.doc]

Supplementary Table 4. The top 50 most important upregulated genes after KIF21B silencing.

| sequence | Gene Symbol | Fold Change | logFC | P-value | FDR |
| --- | --- | --- | --- | --- | --- |
| 1 | HMOX1 | 4.080568045 | 2.02877 | 7.12799E-24 | 2.8023E-19 |
| 2 | TFRC | 2.582496988 | 1.368766667 | 6.79505E-22 | 3.33926E-18 |
| 3 | ARL2BP | 3.058356672 | 1.612756667 | 3.61953E-20 | 7.95998E-17 |
| 4 | GABARAP | 1.967531409 | 0.976386667 | 3.64449E-20 | 7.95998E-17 |
| 5 | SEPT2 | 2.322319818 | 1.215566667 | 8.56075E-20 | 1.56629E-16 |
| 6 | MANF | 1.807847936 | 0.854273333 | 2.40877E-19 | 3.64224E-16 |
| 7 | SDF2L1 | 1.774927381 | 0.82776 | 5.27715E-19 | 7.15399E-16 |
| 8 | ALG5 | 3.176049358 | 1.667233333 | 6.00766E-19 | 7.87283E-16 |
| 9 | HERPUD1 | 2.099093844 | 1.069766667 | 6.76895E-19 | 8.31607E-16 |
| 10 | KRTAP2-3 | 2.219631219 | 1.15032 | 9.37453E-19 | 1.08397E-15 |
| 11 | XIST | 14.59120035 | 3.867026667 | 1.87789E-18 | 1.71692E-15 |
| 12 | CRELD2 | 1.801584933 | 0.849266667 | 2.40954E-18 | 2.10508E-15 |
| 13 | CTSB | 1.881913085 | 0.9122 | 4.1965E-18 | 3.05521E-15 |
| 14 | COL6A3 | 2.136259143 | 1.095086667 | 5.94837E-18 | 4.15214E-15 |
| 15 | IGFBP7 | 2.675787103 | 1.419963333 | 6.69624E-18 | 4.53889E-15 |
| 16 | GPC6 | 2.318443657 | 1.213156667 | 7.99674E-18 | 5.04401E-15 |
| 17 | PDIA6 | 1.528249399 | 0.61188 | 1.26918E-17 | 7.33773E-15 |
| 18 | GRB10 | 2.466479563 | 1.302453333 | 1.36376E-17 | 7.6647E-15 |
| 19 | NNMT | 1.612404074 | 0.689213333 | 1.66358E-17 | 9.08363E-15 |
| 20 | MAP3K7CL | 2.340169994 | 1.226613333 | 2.36298E-17 | 1.16123E-14 |
| 21 | CXCL14 | 2.36416927 | 1.241333333 | 2.65213E-17 | 1.23231E-14 |
| 22 | MAPK9 | 2.951871664 | 1.56163 | 3.71138E-17 | 1.62121E-14 |
| 23 | RALB | 1.917628603 | 0.939323333 | 5.40176E-17 | 2.08416E-14 |
| 24 | HSP90B1 | 1.676377039 | 0.745346667 | 8.36771E-17 | 3.08698E-14 |
| 25 | GSKIP | 2.285267951 | 1.192363333 | 1.09394E-16 | 3.80593E-14 |
| 26 | SERPINA1 | 2.316826485 | 1.21215 | 1.18321E-16 | 4.01006E-14 |
| 27 | DNAJB9 | 2.02563841 | 1.018376667 | 1.24909E-16 | 4.19716E-14 |
| 28 | AXL | 1.550012661 | 0.63228 | 1.32787E-16 | 4.38687E-14 |
| 29 | SCARNA20 | 2.483187729 | 1.312193333 | 1.54032E-16 | 5.00463E-14 |
| 30 | LAMB3 | 2.002353463 | 1.001696667 | 1.74759E-16 | 5.63152E-14 |
| 31 | SERP1 | 2.024145971 | 1.017313333 | 2.02425E-16 | 6.31841E-14 |
| 32 | FXN | 2.035044018 | 1.02506 | 2.3419E-16 | 7.08225E-14 |
| 33 | BTF3L4 | 1.979949088 | 0.985463333 | 3.2752E-16 | 9.33054E-14 |
| 34 | HSPA5 | 2.396878313 | 1.261156667 | 4.07204E-16 | 1.1195E-13 |
| 35 | TGFBR2 | 1.580210406 | 0.660116667 | 4.63257E-16 | 1.23192E-13 |
| 36 | NEK6 | 1.959736925 | 0.97066 | 5.01488E-16 | 1.30566E-13 |
| 37 | METTL21A | 2.093025662 | 1.06559 | 6.40759E-16 | 1.49945E-13 |
| 38 | MRPL33 | 1.794833479 | 0.84385 | 6.83642E-16 | 1.55357E-13 |
| 39 | MCM6 | 1.692379756 | 0.759053333 | 7.32849E-16 | 1.637E-13 |
| 40 | FN1 | 1.641430122 | 0.714953333 | 1.0262E-15 | 2.1346E-13 |
| 41 | AP3S1 | 1.500170696 | 0.585126667 | 1.29141E-15 | 2.60211E-13 |
| 42 | MZT1 | 2.185898885 | 1.128226667 | 1.92904E-15 | 3.56048E-13 |
| 43 | METTL9 | 1.762720007 | 0.817803333 | 2.08492E-15 | 3.79475E-13 |
| 44 | LIPA | 1.975046564 | 0.981886667 | 2.3172E-15 | 4.0669E-13 |
| 45 | RBM15B | 1.875952545 | 0.907623333 | 2.41117E-15 | 4.17987E-13 |
| 46 | FBXO16 | 1.734640461 | 0.794636667 | 2.41346E-15 | 4.17987E-13 |
| 47 | ABL2 | 1.60916991 | 0.686316667 | 2.6091E-15 | 4.4213E-13 |
| 48 | CBFB | 2.046799804 | 1.03337 | 3.00311E-15 | 4.82642E-13 |
| 49 | RCHY1 | 2.05561013 | 1.039566667 | 3.00777E-15 | 4.82642E-13 |
| 50 | SERINC1 | 1.613924772 | 0.690573333 | 3.4585E-15 | 5.48256E-13 |
