# Supplementary material for: KIF21B Expression in Osteosarcoma and Its Regulatory Effect on Osteosarcoma Cell Proliferation and Apoptosis Through the PI3K/AKT Pathway
Source: Front Oncol. 2021 Jan 28;10:606765. doi: 10.3389/fonc.2020.606765 (PMC7879035; doi:10.3389/fonc.2020.606765)
Supplement: Supplementary file 6 [file Table_5.doc]

Supplementary Table 5. The top 50 most important downregulated genes after KIF21B silencing.

| sequence | Gene Symbol | Fold Change | logFC | P-value | FDR |
| --- | --- | --- | --- | --- | --- |
| 1 | DDIT4 | -2.718165563 | -1.442633333 | 1.61E-21 | 7.0484E-18 |
| 2 | TMEM123 | -2.081793062 | -1.057826667 | 5.06008E-20 | 1.04701E-16 |
| 3 | SNAPC1 | -5.525736114 | -2.466166667 | 4.94392E-19 | 6.94162E-16 |
| 4 | G3BP1 | -2.050733471 | -1.03614 | 1.1804E-18 | 1.28465E-15 |
| 5 | SPX | -3.914035027 | -1.968656667 | 1.66256E-18 | 1.61864E-15 |
| 6 | COX7A2 | -4.203021005 | -2.071426667 | 2.4028E-18 | 2.10508E-15 |
| 7 | TMEM30A | -4.203021005 | -2.071426667 | 2.4028E-18 | 2.10508E-15 |
| 8 | MESDC1 | -2.430449795 | -1.281223333 | 2.80877E-18 | 2.28133E-15 |
| 9 | EXOSC1 | -2.288131553 | -1.19417 | 4.56174E-18 | 3.26074E-15 |
| 10 | CSTA | -2.128966625 | -1.090153333 | 8.08295E-18 | 5.04401E-15 |
| 11 | PPP1CA | -1.737119095 | -0.796696667 | 8.40705E-18 | 5.07502E-15 |
| 12 | BEX1 | -1.659026196 | -0.730336667 | 8.51989E-18 | 5.07502E-15 |
| 13 | MSMO1 | -1.817560432 | -0.862003333 | 2.18814E-17 | 1.11957E-14 |
| 14 | DAZAP2 | -2.270966881 | -1.183306667 | 2.32168E-17 | 1.16123E-14 |
| 15 | GHITM | -1.645227015 | -0.718286667 | 2.35367E-17 | 1.16123E-14 |
| 16 | NUP54 | -1.965005477 | -0.974533333 | 2.5783E-17 | 1.22124E-14 |
| 17 | HNRNPH3 | -2.134931824 | -1.09419 | 2.66435E-17 | 1.23231E-14 |
| 18 | ASS1 | -1.954523142 | -0.966816667 | 3.29313E-17 | 1.48811E-14 |
| 19 | FKBP9 | -2.180370541 | -1.124573333 | 3.54731E-17 | 1.56696E-14 |
| 20 | FKBP9P1 | -2.180370541 | -1.124573333 | 3.54731E-17 | 1.56696E-14 |
| 21 | ASNS | -1.55408635 | -0.636066667 | 4.0106E-17 | 1.73267E-14 |
| 22 | GNPNAT1 | -1.965118983 | -0.974616667 | 5.68766E-17 | 2.17092E-14 |
| 23 | MAT2B | -2.476255192 | -1.30816 | 8.54125E-17 | 3.10917E-14 |
| 24 | PTP4A1 | -1.53913168 | -0.622116667 | 9.33325E-17 | 3.36631E-14 |
| 25 | UBE2D3 | -1.729761741 | -0.790573333 | 9.99064E-17 | 3.5487E-14 |
| 26 | EMC10 | -1.691238354 | -0.75808 | 1.00195E-16 | 3.5487E-14 |
| 27 | PPP1CC | -1.672999097 | -0.742436667 | 1.06322E-16 | 3.7321E-14 |
| 28 | ARL6IP6 | -2.079826716 | -1.056463333 | 1.13175E-16 | 3.90294E-14 |
| 29 | PPP1CB | -1.80300492 | -0.850403333 | 2.46619E-16 | 7.40122E-14 |
| 30 | TMEM189 | -1.784343452 | -0.835393333 | 2.99277E-16 | 8.71539E-14 |
| 31 | YIPF5 | -1.680588674 | -0.748966667 | 3.19571E-16 | 9.23795E-14 |
| 32 | SLC39A14 | -1.568983304 | -0.64983 | 3.24538E-16 | 9.31306E-14 |
| 33 | ICMT | -2.792124618 | -1.481363333 | 3.51093E-16 | 9.93012E-14 |
| 34 | MAGEA4 | -1.611338949 | -0.68826 | 3.54251E-16 | 9.94787E-14 |
| 35 | MRPL16 | -1.691984869 | -0.758716667 | 4.75509E-16 | 1.24628E-13 |
| 36 | KLHDC3 | -1.692063057 | -0.758783333 | 5.32304E-16 | 1.34147E-13 |
| 37 | ANKH | -1.841745009 | -0.881073333 | 5.45266E-16 | 1.36539E-13 |
| 38 | TSPAN3 | -1.841745009 | -0.881073333 | 5.45266E-16 | 1.36539E-13 |
| 39 | C8orf76 | -1.911577025 | -0.934763333 | 6.21618E-16 | 1.48158E-13 |
| 40 | TPM1 | -1.831505458 | -0.87303 | 6.21815E-16 | 1.48158E-13 |
| 41 | XRN2 | -1.655132444 | -0.726946667 | 8.54993E-16 | 1.85708E-13 |
| 42 | RRM1 | -1.628461938 | -0.70351 | 8.70081E-16 | 1.87947E-13 |
| 43 | LACTB | -1.906658764 | -0.931046667 | 9.11938E-16 | 1.94848E-13 |
| 44 | CDKN1A | -1.683429534 | -0.751403333 | 9.68827E-16 | 2.05884E-13 |
| 45 | NEK7 | -1.882983033 | -0.91302 | 9.81664E-16 | 2.0638E-13 |
| 46 | C8orf33 | -1.816452114 | -0.861123333 | 1.0772E-15 | 2.21722E-13 |
| 47 | PCK2 | -2.127329242 | -1.089043333 | 1.26339E-15 | 2.57352E-13 |
| 48 | MAP2K3 | -2.157623789 | -1.109443333 | 1.29728E-15 | 2.60211E-13 |
| 49 | PSAT1 | -1.611186314 | -0.688123333 | 1.49041E-15 | 2.91027E-13 |
| 50 | CERS2 | -1.618289816 | -0.69447 | 1.57562E-15 | 3.01489E-13 |
